# Genome-wide profiling of DNA methylation and gene expression in esophageal squamous cell carcinoma

**DOI:** 10.18632/oncotarget.6607

**Published:** 2015-12-14

**Authors:** Chen Chen, Hao Peng, Xiaojie Huang, Ming Zhao, Zhi Li, Ni Yin, Xiang Wang, Fenglei Yu, Bangliang Yin, Yunchang Yuan, Qianjin Lu

**Affiliations:** ^1^ Department of Thoracic Surgery, The Second Xiangya Hospital, Central South University, Changsha, P.R. China; ^2^ Department of Thoracic and Cardiovascular Surgery, Tongji Hospital, Tongji University School of Medicine, Shanghai, P.R. China; ^3^ Department of Cardiovascular Surgery, The Second Xiangya Hospital, Central South University, Changsha, P.R. China; ^4^ Hunan Key Laboratory of Medical Epigenomics, Department of Dermatology, The Second Xiangya Hospital, Central South University, Changsha, P.R. China; ^5^ Beijing Genomics Institute at Shenzhen, Shenzhen, P.R. China

**Keywords:** esophageal squamous cell carcinoma, MeDIP-Seq, DNA methylation, RNA-Seq, gene expression

## Abstract

Esophageal squamous cell carcinoma (ESCC) is the leading cause of cancer-related death worldwide. Previous studies have suggested that DNA methylation involved in the development of ESCC. However, the precise mechanisms underlying the regulation and maintenance of the methylome as well as their relationship with ESCC remain poorly understood. Herein, we used methylated DNA immunoprecipitation sequencing (MeDIP-Seq) and RNA-Seq to investigate whole-genome DNA methylation patterns and the genome expression profiles in ESCC samples. The results of MeDIP-Seq analyses identified differentially methylated regions (DMRs) covering almost the entire genome with sufficient depth and high resolution. The gene ontology (GO) analysis showed that the DMRs related genes belonged to several different ontological domains, such as cell cycle, adhesion, proliferation and apoptosis. The RNA-Seq analysis identified a total of 6150 differentially expressed genes (3423 up-regulated and 2727 down-regulated). The significant GO terms showed that these genes belonged to several molecular functions and biological pathways. Moreover, the bisulfite-sequencing of genes MLH1, CDH5, TWIST1 and CDX1 confirmed the methylation status identified by MeDIP-Seq. And the mRNA expression levels of MLH1, TWIST1 and CDX1 were consistent with their DNA methylation profiles. The DMR region of MLH1 was found to correlate with survival. The identification of whole-genome DNA methylation patterns and gene expression profiles in ESCC provides new insight into the carcinogenesis of ESCC and represents a promising avenue through which to investigate novel therapeutic targets.

## INTRODUCTION

Esophageal cancer, mainly including squamous cell carcinoma and adenocarcinoma, is the sixth leading cause of cancer-related death and the eighth most common cancer worldwide [1, 2]. It is considered as a serious malignancy with respect to its extremely aggressive histopathological features and poor survival rate [3]. Esophageal squamous cell carcinoma (ESCC), which mainly occurs in an area referred to the “esophageal cancer belt” that extends from northeast China to the Middle East [4], constitutes the vast majority of cases (more than 90%) [5, 6]. Dietary and environmental factors, such as smoking, alcohol consumption, obesity, chronic irritation and high levels of nitrates in the soil and drinking water, are strongly believed to be associated with the occurrence of ESCC [1, 7]. Currently, surgical removal remains the most commonly employed treatment for patients with ESCC. However, the success of surgery strongly depends on early diagnosis. Current reliable diagnostic biomarkers are very limited in clinic [8]. With the increasing understanding of genetic and epigenetic mechanisms of the carcinogenesis, many studies indicated that highly sensitive and specific molecular biomarkers would help to optimize the clinical management of esophageal carcinomas and improve patient outcomes.

DNA methylation, as a gene silencing mechanism, plays essential roles in several developmental and cellular processes such as transcription, embryonic development, X-chromosome inactivation and genomic imprinting[9-11]. In mammals, DNA methylation occurs almost exclusively at the 5′-carbon position of cytosine residues within CpG pairs, and has a profound effect on gene expression[12]. The methylation of gene promoter region inhibits the binding of some transcription factors, which usually contain a methylated-DNA binding domain, resulting in transcriptional repression. Many tumor suppressor genes (TSGs), such as CDKN2A, FHIT, MGMT, RASSF1A, CDH1 and APC, have been reported to be silenced by promoter hypermethylation in the development of breast cancer [13], lung cancer[14, 15], thymic epithelial tumors[16], colorectal cancer[17, 18] and ESCC [19]. On the other hand, some of the oncogenes, such as GADD45A, were abnormally activated by hypomethylated changes, contributing to the occurrence of ESCC [20, 21].

Although previous studies have provided many clues to understand the relationship between DNA methylation and gene regulation in the development of ESCC, the information is still very limited. In order to acquire quantitative and qualitative information on DNA methylation, a wide range of approaches have been developed. The high-throughput sequencing (or next-generation sequencing) technologies has dramatically improved sequencing capabilities through the massive parallelization of reactions on millions of DNA fragments at once[22]. Methylated DNA immunoprecipitation (MeDIP) is a large-scale purification technique used for enrichment of methylated DNA sequences via an antibody against 5-methylcytosine (5-mC). Therefore, a novel method termed methylated DNA immunoprecipitation sequencing (MeDIP-Seq) has emerged as an advantageous tool for identifying methylated CpG-rich sequences much faster than ever before[23].

To investigate the genome-wide profiling of DNA methylation and gene expression in ESCC, MeDIP-Seq and quantitative measurements of transcriptomes (RNA-Seq) were performed in this study. Owing to the tremendous progress in next-generation sequencing technology, MeDIP-Seq and RNA-Seq can offer higher resolution, less noise and greater coverage results [23-25]. The combination of MeDIP-Seq and RNA-Seq may provide more information about genome-wide epigenetic regulation in gene expression and will bring new insight on the DNA methylation in the development of ESCC.

## RESULTS

### High-throughput MeDIP sequencing analysis

We isolated genomic DNA from 4 pairs of ESCC and NE tissue samples, and then put equal amounts of genomic DNA of 4 individuals into one pool for each group (the ESCC group and the NE group). The MeDIP-Seq was conducted on the two groups using the Hiseq 2000 sequencing system, which provided high accuracy and unprecedented output.

**Figure 1:**
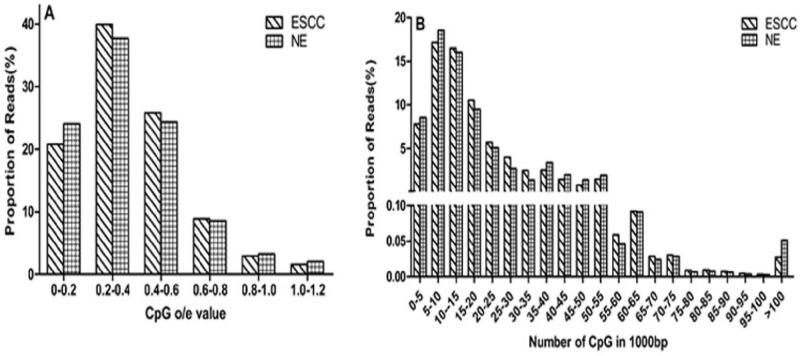
Distribution of reads varies CpG density **A.** Distribution of reads varies CpG o/e value. The x axis indicates the range of CpG o/e values and the y axis indicates the proportion of reads in a specific range. CpG o/e value can reflect the CpG density of a specific region. DNA hypermethylation may have lower CpG o/e value, while hypomethylation maintain high o/e value. **B.** Overall distribution of reads at 1kb resolution level varies CpG density. The x axis indicates the CpG density and the y axis indicates the proportion of reads.

In total, we obtained about 86 million clean 49-bp reads after sequencing. And the unique mapping rates satisfied the requirements. In the ESCC group, 90.89 % of the reads could be mapped to human genome and 73.26% were uniquely mapped to human genome. While in NE group, 88.85% of the reads could be mapped to the human genome and 68.43% could be uniquely mapped to the human genome (Table 1 and Supplementary Fig. S1). To understand the global DNA methylation status in ESCC, we directly compared the overall distribution of reads at 1kb resolution level. The CpG o/e values were calculated to reflect the CpG density of specific regions[26]. As expected, both hypermethylated and hypomethylated changes were found in ESCC samples compared to NE (Figure 1). Model-based analysis of ChIP-Seq (MACS)[27] identified 286,272 and 260,746 regions (peaks) from the ESCC and NE MeDIP-Seq data, respectively. The average length of peaks varied from 1,095 bp (ESCC) to1, 124 bp (NE). Peak-based differential analysis was conducted for gene elements that were covered by two groups and exhibited a greater than 2-fold change in methylation and a p-value of less than 0.01 (Table 2 and Supplementary Fig. S2).

**Table 1:** Number of reads generated by MeDIP-seq for each group

Sample group	Total number of reads	Total number of mapped reads	Mapping rate(%)	Total number of unique mapped reads	Unique mapping rate (%)
ESCC	85,714,286	77,904,067	90.89	62,791,726	73.26
NE	85,714,286	76,156,660	88.85	58,652,189	68.43

**Table 2:** Number of peak-related differences in gene methylation identified between groups

# of genes	NE vs ESCC (up-regulated)	NE vs ESCC (down-regulated)
Upstream 2K	960	52
5′UTR	322	28
CDS	2071	193
Intron	6702	1455
3′UTR	635	40
Downstream 2K	864	42

The main goal of this study was to identify local differentially methylated regions (DMRs) between ESCC and NE groups in a genome-wide level. In total we identified 26,081 DMRs, 87.6% of them were hypermethylated and 12.4% of them were hypomethylated relative to NE group. The distribution of the DMRs showed that most of the DMRs were located at gene body (Table 3). Gene ontology (GO) analysis of genes with DMRs was also conducted, for both hypermethylated and hypomethylated genes (Supplementary Fig. S3).

**Table 3:** Number of DMRs in six characteristic genomic areas

Genomic areas	Hypermethylated	Hypomethylated
Upstream 2K	1094	64
5′UTR	378	32
CDS	2792	224
Intron	16932	2824
3′UTR	727	47
Downstream 2K	915	52

### Validation of the MeDIP-Seq results

Four genes were selected to validate the results of the MeDIP-Seq: MLH1, CDH5, TWIST1 and CDX1. We mainly focus on the DMRs located at promoter region, which may contribute the regulation of gene expression. The starting and ending point of the DMRs of these four genes, the CG sites and the primers are showed in Supplementary Table S1.

**Figure 2:**
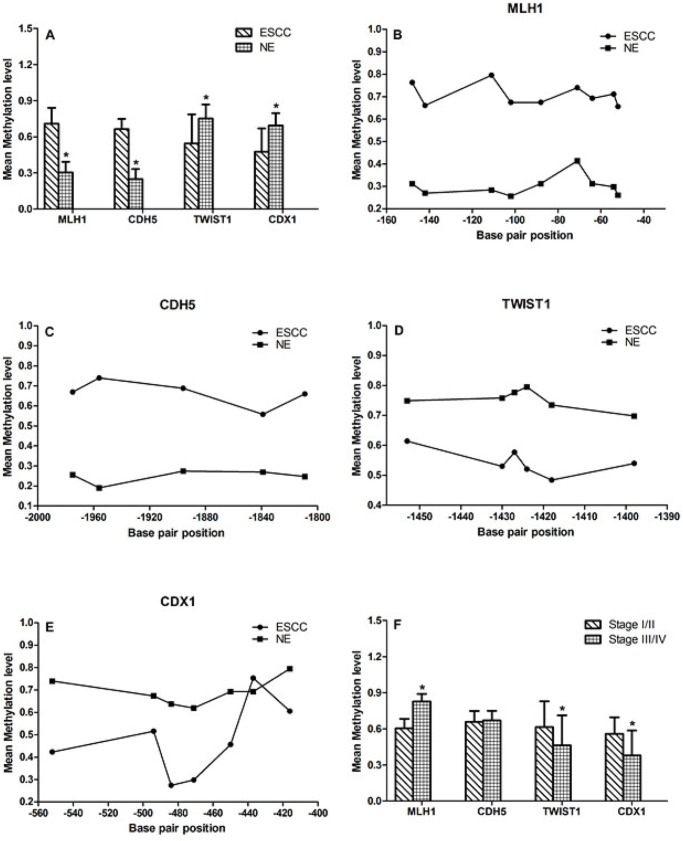
DNA methylation at genes locus **A, B** and **C.** Compare to NE group, the MLH1 and CDH5 were significantly hypermethylated in ESCC samples (0.707±0.133 vs 0.302±0.09, p<0.001; 0.663±0.086 vs 0.247±0.084, p<0.001). **A, D** and **E.** Compare to NE group, TWIST1 and CDX1 were significantly hypomethylated in ESCC samples (0.544±0.241 vs 0.752±0.117, p<0.001; 0.475±0.194 vs 0.693±0.102, p<0.001). **F.** DNA methylation level of MLH1, TWIST1 and CDX1 were related to TNM stage.

Of the18 CpG sites from MLH1 DMR region, 9 continuous clustered CpG loci (−52, −54, −64, −71, −88, −102, −111, −142, −148) were identified to be significantly hypermethylated in ESCC samples than that in NE samples (0.707±0.133 vs 0.302±0.09, p<0.001; Fig. 2A and 2B). Compared to NE, CDH5 DMR region were also significantly hypermethylated in ESCC samples (0.663±0.086 vs 0.247±0.084, p<0.001; Fig. 2A and 2C).

There were no significant differences in the DNA methylation status of all the 13 CpG sites of the TWIST1 DMR region (−1500bp∼-1320), however, 6 continuous clustered CpG loci (−1398, −1418, −1424, −1427, −1430, −1453) showed hypomethylated changes in ESCC samples (0.544±0.241 vs 0.752±0.117, p<0.001; Fig. 2A and 2D). Moreover, significant hypomethylation of CDX1 DMR region were also found in the ESCC group compared to NE group (0.475±0.194 vs 0.693±0.102, p<0.001; Fig. 2A and 2E).

The DNA methylation level of MLH1, TWIST1 and CDX1 were related to TNM stage (MLH1: p<0.001; CDH5: p=0.636; TWIST1: p=0.038; CDX1: p=0.002; Fig. 2F). There was no significant correlation between DNA methylation levels and tumor histological differentiation.

### Identification of gene expression by RNA-Seq

RNA-Seq was performed to investigate the genome-wide expression changes in ESCC and adjacent NE. The sequencing provided us about 26.5 million clean 49-bp reads. In ESCC, 88.28% of the reads were mapped to the human genomic sequence with no more than two mismatches and 82.66 % were uniquely mapped to the human genome, while in NE library, 88.78 % of the reads were mapped to the human genomic sequence and 83.92 % could be uniquely mapped (Table 4). For genes with multiple transcripts, we chose the longest transcript for further analysis.

**Table 4:** Number of reads generated by RNA-seq for each group

Sample group	Total number of reads	Total number of mapped reads	Mapping rate(%)	Total number of unique mapped reads	Unique mapping rate (%)
ESCC	26,708,458	23,578,386	88.28	22,077,327	82.66
NE	26,552,622	23,573,912	88.78	22,283,765	83.92

**Figure 3:**
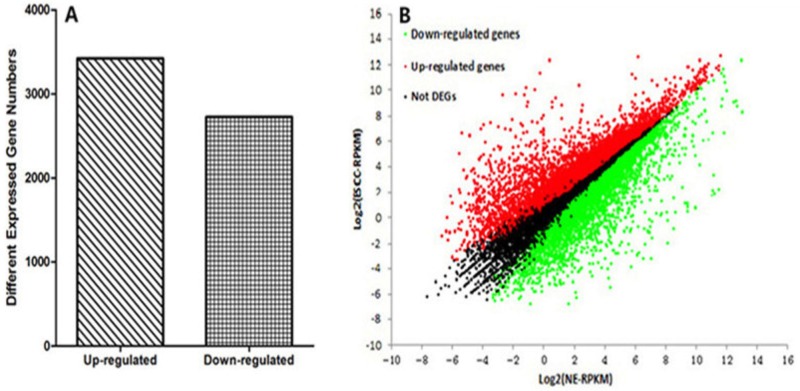
Whole genome expression profiles **A.** Differentially expressed gene numbers between ESCC and NE. The y axis indicates the gene numbers. **B.** Comparison of gene expression level between ESCC and NE. Red dots represent transcripts more prevalent in ESCC, green dots show those down-regulated genes in ESCC and black dots indicate gene expression did not change significantly.

**Figure 4:**
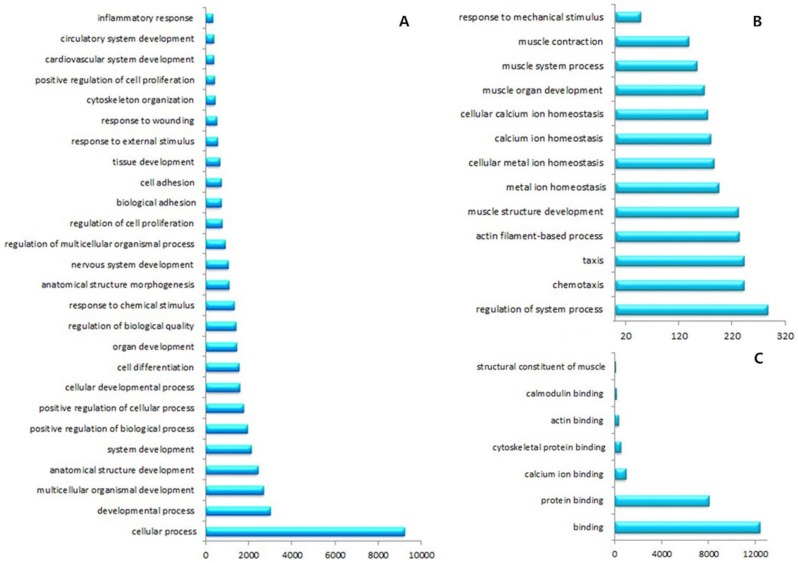
GO analysis showed that these DEGs related to many biological processes and molecular functions **A.** and **B.** DEGs involved in many biological processes. **C.** DEGs correlated with molecular functions.

**Figure 5:**
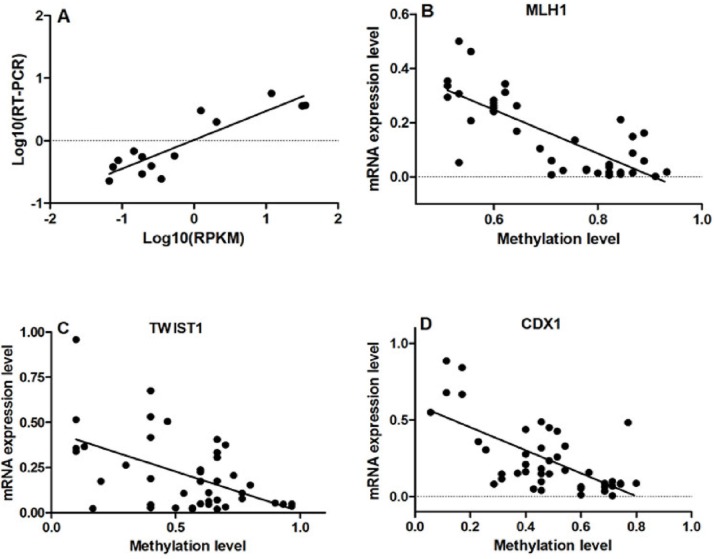
Results of correlation analysis **A.** High level of concordance between qRT-PCR results and RNA-Seq data confirmed the RNA-Seq results were acceptable (r=0.889, p<0.001). **B, C** and **D.** Correlation analyses showed that the DNA methylation levels of MLH1, TWIST1 and CDX1 were negatively correlated with their mRNA expression (MLH1: r=−0.776, p<0.001; TWIST1: r=−0.515, p<0.001; CDX1: r=−0.649, p<0.001).

Gene expression was initially estimated by calculating the density of uniquely mapped reads as “reads per kilo base of exon model per million mapped reads” (RPKM). Differentially expressed genes (DEGs) were identified by using a threshold of 0.1 % false discovery rate (FDR) and |log2ratio|≥1 (ratio = treated/control RPKM). A total of 6150 genes (3423 up-regulated and 2727 down-regulated) were differentially expressed between ESCC and NE groups (Fig. 3 and Supplementary Fig. 2). To study biological functions, GO analysis was performed on the differentially expressed genes with p values < 0.005[28]. The significant GO terms showed that these DEGs were related to many biological processes and molecular functions (Fig. 4 and Supplementary Fig. 3). Most of these terms were closely related to the cell differentiation, adhesion, cell proliferation, response to DNA damage, or DNA replication. Among these DEGs, we identified many previously described tumor-related genes, such as MLH1, CDKN2A, DAPK1, CDX1, TWIST1, CDH5 and CXCL5 [29-33]. The KEGG pathway analysis showed that these DEGs were involved in 197 pathways. Among these pathways, 33 showed significant (Q<0.05) enrichment of DEGs in ESCC (Table 5). Furthermore, the quantitative real time RT-PCR (qRT-PCR) was performed to validate the results of RNA-Seq. In total the expression of 14 genes were investigated in ESCC and NE samples. A high level of concordance between qRT-PCR results and RNA-Seq data confirmed the RNA-Seq results were acceptable (r=0.889, p<0.001; Fig. 5A).

**Table 5:** Significantly enriched pathways for DEGs

Pathway	DEGs with pathway annotation	All genes with pathway annotation	Q value	Pathway ID
Dilated cardiomyopathy	66	108	2.611394e-09	ko05414
Hypertrophic cardiomyopathy (HCM)	61	101	1.971738e-08	ko05410
Cell adhesion molecules (CAMs)	74	134	1.292208e-07	ko04514
Calcium signaling pathway	99	193	1.515844e-07	ko04020
Arrhythmogenic right ventricular cardiomyopathy (ARVC)	48	80	8.359827e-07	ko05412
ECM-receptor interaction	46	84	4.016593e-05	ko04512
Vascular smooth muscle contraction	62	129	0.0003407075	ko04270
Focal adhesion	93	208	0.0003635684	ko04510
Long-term potentiation	37	70	0.00056412	ko04720
Viral myocarditis	38	73	0.0007063471	ko05416
Axon guidance	60	129	0.001193845	ko04360
Melanogenesis	50	104	0.001217776	ko04916
Cytokine-cytokine receptor interaction	115	277	0.002342581	ko04060
Glycine, serine and threonine metabolism	18	31	0.003986321	ko00260
Hematopoietic cell lineage	46	99	0.004332282	ko04640
Type I diabetes mellitus	23	43	0.004977168	ko04940
Phosphatidylinositol signaling system	36	76	0.00748928	ko04070
Steroid biosynthesis	11	17	0.007951011	ko00100
Regulation of actin cytoskeleton	94	230	0.009258026	ko04810
Prion diseases	22	43	0.01161543	ko05020
Leukocyte transendothelial migration	51	118	0.0152988	ko04670
Bladder cancer	23	47	0.01890428	ko05219
p53 signaling pathway	34	75	0.01990306	ko04115
Cardiac muscle contraction	42	96	0.02082785	ko04260
Pathways in cancer	135	351	0.02250529	ko05200
Renin-angiotensin system	10	17	0.02715161	ko04614
DNA replication	18	36	0.02807702	ko03030
Arginine and proline metabolism	25	54	0.03205055	ko00330
Cell cycle	61	150	0.03473822	ko04110
Basal cell carcinoma	25	55	0.04061275	ko05217
Primary immunodeficiency	17	35	0.04385614	ko05340
PPAR signaling pathway	31	71	0.04405247	ko03320
N-Glycan biosynthesis	22	48	0.04797281	ko00510

### Association of DNA methylation changes, differential gene expression and clinical pathological features

The qRT-PCR results showed that the mRNA expression of MLH1, CDH5, ITIH5, CRABP1, CDKN2A, CDO1 and FHIT were significantly down-regulated in ESCC samples compared to NE samples. In contrast, the mRNA expression of TWIST1, CXCL5, GADD45A, WNT3A and CDX1 was significantly up-regulated in ESCC samples than that in NE (Supplementary Fig. S4). Correlation analyses showed that the DNA methylation levels of MLH1, TWIST1 and CDX1 were negatively correlated with their mRNA expression (MLH1: r=−0.776, p<0.001; TWIST1: r=−0.515, p<0.001; CDX1: r=−0.649, p<0.001; Fig. 5B, 5C and 5D).

**Figure 6:**
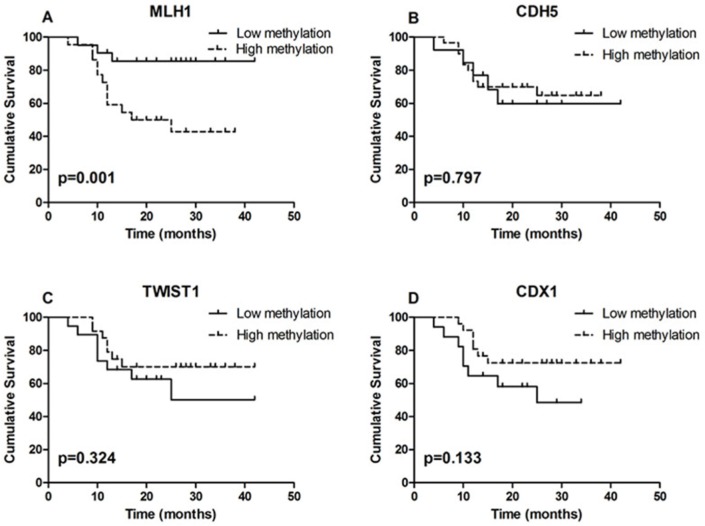
Results of survival analysis **A.** Survival analysis showed that the aberrant methylation of MLH1 significantly related to patient survival (p=0.001). **B, C.** and **D.** DNA methylation level of CDH5, TWIST1 and CDX1 did not correlated with patient survival (p=0.797; p=0.324 and p=0.133).

For survival analysis, patients were grouped into low methylation or high methylation groups according to their methylation Z scores. Survival analysis showed that the aberrant methylation of MLH1 significantly related to patients’ survival (p=0.001; Fig. 6).

## DISCUSSION

In this study, we used the high-throughput MeDIP-Seq and RNA-Seq to examine whole-genome DNA methylation patterns and messenger RNA transcriptome in a total of 4 pairs of ESCC samples and NE samples. Our findings provided a comprehensive, detailed picture of DNA methylation patterns and gene expression levels in patients with ESCC. The DMRs identified by MeDIP-Seq spanned almost the entire genome with sufficient depth and high resolution, and the number of DMRs was much greater than that detected by traditional approaches, indicating that this method represents an effective approach for DNA methylome studies [22, 23]. Moreover, the bisulfite sequencing analysis of MLH1, CDH5, TWIST1 and CDX1 were in accordance with the MeDIP-Seq results. It also proved the high accuracy of the high-throughput sequencing technique.

With the increasing availability and applications of high-throughput sequencing methods, more and more studies have reported the methylation of tissue-specific genes mainly occurred within the gene body instead of the 5′promoters [34-36]. Our study also found that most of the DMRs occurred within the gene body, only small proportion of DMRs located at the 5′promoter region. Moreover, the great number of uniquely mapped reads located at the intronic region, indicating the DNA methylation in introns may have important regulatory functions.

To understand the global DNA methylation pattern in ESCC, we compared the overall distribution of reads between ESCC and NE groups. As expected, both hypermethylated and hypomethylated changes were observed in ESCC samples compared to NE (Fig. 1). The similar results have been shown in previous studies [37, 38].

In total of 6150 differentially expressed genes were identified by RNA-Seq in our study. The results of RNA-Seq were confirmed by qRT-PCR from validation of a number of randomly selected genes. GO analysis revealed these DEGs were significantly enriched in many cell related process and molecular functions, such as cell differentiation, apoptosis, adhesion and proliferation. Aberrant expressions of some of these genes were previously reported to be important in the development of ESCC [28, 39, 40]. Pathway analysis highlighted many pathways which were closely related to the carcinogenesis of ESCC, such as cell adhesion molecules, p53 and PPAR signaling pathways, providing new clues for understanding the molecular mechanisms of ESCC pathogenesis.

In light of previously described effects of DNA methylation on promoters[41], we selected DMRs from upstream 2kbp of transcription start site to validate the MeDIP-Seq results. According to GO analysis and pathway analysis, DMRs were identified in genes associated with cell cycle, adhesion, apoptosis and many biological pathways, which were closely correlated with carcinogenesis. As such, we selected four genes for further study: TWIST1, CDX1, MLH1 and CDH5.

TWIST1 is one of the epithelial-to-mesenchymal transition (EMT) inducer prototypes, which lead to epithelial cells lose their adhesion properties and acquire mesenchymal features allowing their migration and invasion [42]. The overexpression of TWIST1 has been reported in previous studies showing that it could be a biomarker for tumor progression and metastasis [43-46]. Interestingly, promoter hypermethylation of TWIST1 was also observed in the development of colorectal cancer, vulvar cancer and tonsillar squamous cell carcinoma [47-49]. Recently, Galvan et al. reported strong inverse correlations between TWIST1 methylation and stromal expression of TWIST1 in colon cancer [50]. Wong and colleagues reported promoter-specific hypomethylation of TWIST1 was strongly associated with gene overexpression [51], indicating the promoter methylation may regulate the TWIST1 expression. Our MeDIP-Seq results, for the first time, identified a DMR (from −1010 to −1715) in upstream 2K of TWIST1. Significant hypomethylated changes of 6 continuous clustered CpG loci (−1398, −1418, −1424, −1427, −1430, −1453) were identified in this region. Overexpression of TWIST1 was detected in both RNA-Seq data and qRT-PCR validation test, with adverse correlation with DNA hypomethylation. Nevertheless, survival analysis did not found significant correlation between overexpression or methylation of TWIST1 and patients’ survival. Our results indicated that the DNA hypomethylation, to some degree, has regulatory function for the expression of TWIST1 in ESCC.

CDX1 was previously described as an oncogene since it regulated Ras, Wnt/β-catenin and PI3 kinase pathways leading to transformation and tumorigenesis of intestinal epithelial cells [52]. Recent studies showed that CDX1 expression was down-regulated by promoter hypermethylation in colon cancer [31, 53], whereas a subset of colon cancers may express increased levels of CDX1 mRNA and protein [54]. Our results showed that the hypomethylation of CDX1 was related to the gene overexpression in ESCC samples, which indicated that the CDX1 might have oncogenic potential in the development of ESCC [55].

The silencing and promoter hypermethylation of tumor suppressor gene MLH1 and CDH5 have been reported in various cancers [56-59]. Deng and colleagues reported that MLH1 silencing by methylation is region specific. The loss of expression of MLH1 was correlated with the proximal region (from −322 to +56), but not the distal region (from −796 to −547) [60]. In our study, the identified MLH1 DMR (from −250 to −10) also located at proximal region. In validation test, hypermethylation in this region was detected and related to gene silencing. Moreover, the survival analysis showed that the methylation of MLH1 significantly correlated with patient survival, indicating the MLH1 might play important role in the carcinogenesis of ESCC. Together, our results show that genome-wide aberrant DNA methylation of cancer-associated genes may be involved in the pathogenesis of ESCC.

## MATERIALS AND METHODS

### Tissue samples

Fresh frozen ESCC samples and paired adjacent normal esophageal (NE) tissue samples (healthy surrounding esophageal tissue more than 5 cm away from tumor) were obtained from 47 ESCC patients at the Second Xiangya Hospital of Central South University, Changsha, China. The 25 male and 22 female patients ranged between 45 and 71 years of age (mean 58.9 ± 6.9 years) and all had undergone ESCC surgery between April 2010 and August 2013. None of the patients had received preoperative treatments, such as chemotherapy or radiotherapy. Control samples were histologically and pathologically confirmed as healthy esophageal tissues, while all tumor samples were confirmed as ESCC, and staged according to the TNM system. Only samples with a tumor cell content of more than 80% were included in this study. A summary of clinical and pathological characteristics of patients included in this study is presented in Table 6.

**Table 6:** Characteristics of 47 patients with ESCC

Patient No.	Gender/age (years)	Alcohol History	TNM Stage	Histological Differentiation
1	F/60	No	IIb	Moderate
2	M/56	Yes	IIb	Moderate
3	M/56	Yes	IIIa	Moderate
4	F/48	No	IIIa	Moderate
5	F/45	Yes	Ia	Low
6	M/56	Yes	Ib	Moderate
7	F/67	No	Ib	High
8	M/67	Yes	Ib	Moderate
9	M/59	Yes	Ib	High
10	M/65	Yes	Ib	Moderate
11	M/49	No	Ib	High
12	F/52	Yes	Ib	Moderate
13	M/53	No	IIb	Moderate
14	F/65	No	IIb	Low
15	M/51	Yes	IIb	Moderate
16	F/51	No	IIb	Moderate
17	F/69	Yes	IIb	Moderate
18	M/68	Yes	IIIb	Moderate
19	F/59	No	IIIb	High
20	F/48	No	Ia	Low
21	F/71	No	IIa	Moderate
22	F/64	No	IIa	Moderate
23	M/71	Yes	IIa	Moderate
24	M/64	Yes	IIb	High
25	F/59	Yes	IIb	High
26	M/71	Yes	IIb	High
27	M/61	Yes	IIb	Moderate
28	F/60	No	IIb	Low
29	M/65	Yes	IIb	High
30	F/58	No	IIb	Moderate
31	M/58	No	IIb	High
32	M/59	Yes	IIIa	Moderate
33	M/59	No	IIIa	Low
34	F/71	Yes	IIIa	Moderate
35	M/52	Yes	IIIa	Moderate
36	F/48	No	IIIa	Low
37	F/57	Yes	IIIa	High
38	F/62	No	IIIa	High
39	M/62	Yes	IIIa	Low
40	F/50	Yes	IIIb	High
41	F/62	No	IIIb	High
42	M/48	Yes	IIIb	High
43	M/55	Yes	IIIc	High
44	M/61	Yes	IIIc	Low
45	F/60	No	IIIc	High
46	M/62	Yes	IIIc	Moderate
47	M/65	Yes	IV	High

This study was approved by the Human Ethics Committee of the Second Xiangya Hospital, Central South University. Written informed consents were obtained from all participants.

### Genomic DNA and total RNA isolation and pooling

Genomic DNA was isolated from ESCC and NE tissue samples using Qiagen DNeasy Kits (Qiagen, Germany) according to the manufacturer's instructions. For MeDIP-Seq, an equal quantity of DNA of four parallel individuals (Patient No.1, 2, 3 and 4) from ESCC and NE groups was then pooled respectively.

Total RNA was isolated from each sample with TRIzol Reagent (Invitrogen, USA) according to the manufacturer's instructions, then treated with RNase-free DNase I for 30 min at 37°C(New England BioLabs) to remove residual DNA. The integrity of total RNA was checked using an Agilent Technologies 2100 Bioanalyzer, and all samples had an RNA Integrity Number (RIN) value greater than eight. Pooling of the RNA samples was the same as the DNA pooling, using for RNA-Seq.

### MeDIP-Seq, RNA-Seq and data analysis

The detection and analysis of MeDIP-Seq and RNA-Seq were performed as described previously [61, 62]. Pathway enrichment analysis was based on KEGG database (http://www.genome.jp/kegg/), and Q value was used for determining the threshold of significance in multiple test and analysis. Pathways with a Q value <0.05 are considered significantly enriched in differentially expressed or modified genes (Supplementary Fig. S5).

### Bisulfite conversion and sequencing

Bisulfite conversion was performed using the EpiTect Bisulfite Kit (Qiagen, Germany), according to manufacturer's instruction. Fragments about 250 bp flanking each side of the loci of MLH1, CDH5, TWIST1 and CDX1 were amplified by PCR. The fragments were cloned into pGEM-T vectors (Promega, USA) and independent clones were sequenced for each of the amplified fragments[63]. The primers designed for the BSP were showed in Supplementary Table S1.

### Real-time quantitative RT-PCR

Real-time quantitative RT-PCR was performed using a Prism 7500 Sequence Detection System (ABI, USA) and mRNA levels were quantified using the SYBR®Premix Ex Taq™(Takara Bio Inc., Dalian, China). A dilution series of sample RNA was also included to generate a standard curve used to calculate the relative concentrations of transcript present in each sample. Negative controls (in which water was substituted for RNA) were run for each sample. β-actin was also amplified and used as a loading control. Specific primers used for amplification were showed in Supplementary Table S2.

### Statistical analysis

To evaluate the methylation level of individual genes, methylation for each gene among the patients was standardized by the Z score method[64]. Data was analyzed using SPSS 20.0 software (SPSS Inc., Chicago, USA) and are presented as the mean ± standard deviation (SD). Student's t-test was used to compare continuous variables and Pearson's correlation test was used for correlation analyses. Overall survival was calculated by Log-rank tests and the Kaplan-Meier test was used to generate survival curves. P values of less than 0.05 were considered significant.

## SUPPLEMENTARY FIGURES AND TABLES




