# Supplementary material for: Genome-wide profiling of DNA methylation and gene expression in esophageal squamous cell carcinoma
Source: Oncotarget. 2015 Dec 14;7(4):4507–21. doi: 10.18632/oncotarget.6607 (PMC4826222; doi:10.18632/oncotarget.6607)
Supplement: Supplementary file 1 [file oncotarget-07-4507-s001.pdf]

SUPPLEMENTARY FIGURES AND TABLES

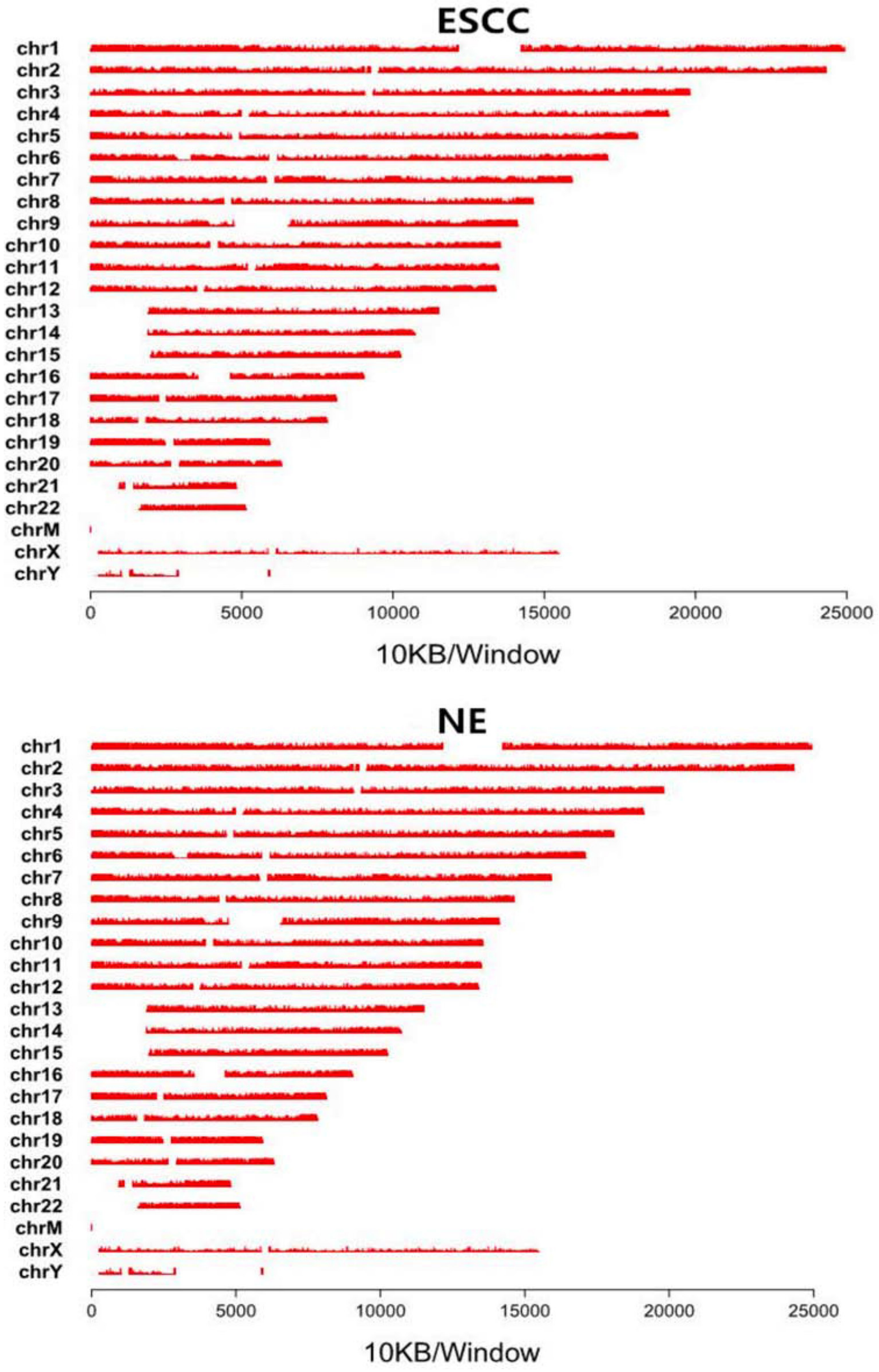

Supplementary Figure S1: Distribution of MeDIP-Seq reads on each chromosome. The X axis indicates the number of windows. The y axis indicates the normalized read count of each window. This figure shows the distribution of reads on each chromosome.

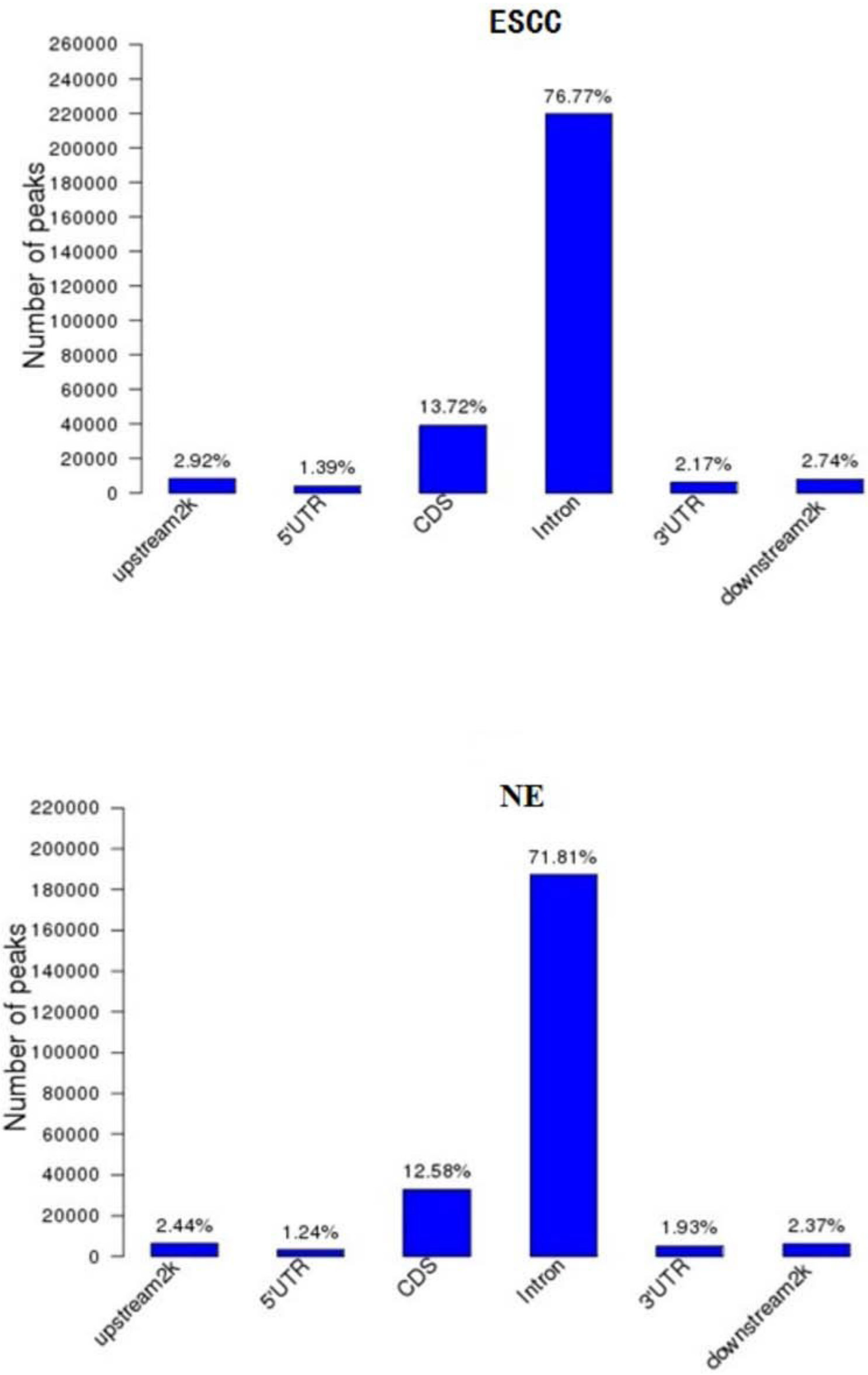

Supplementary Figure S2: Count of peaks on each gene element. The X axis indicates different gene elements and the y axis indicates the number of peak in a specific gene element. Peaks are concentrated in intron because the total length of intron is much larger than other elements.

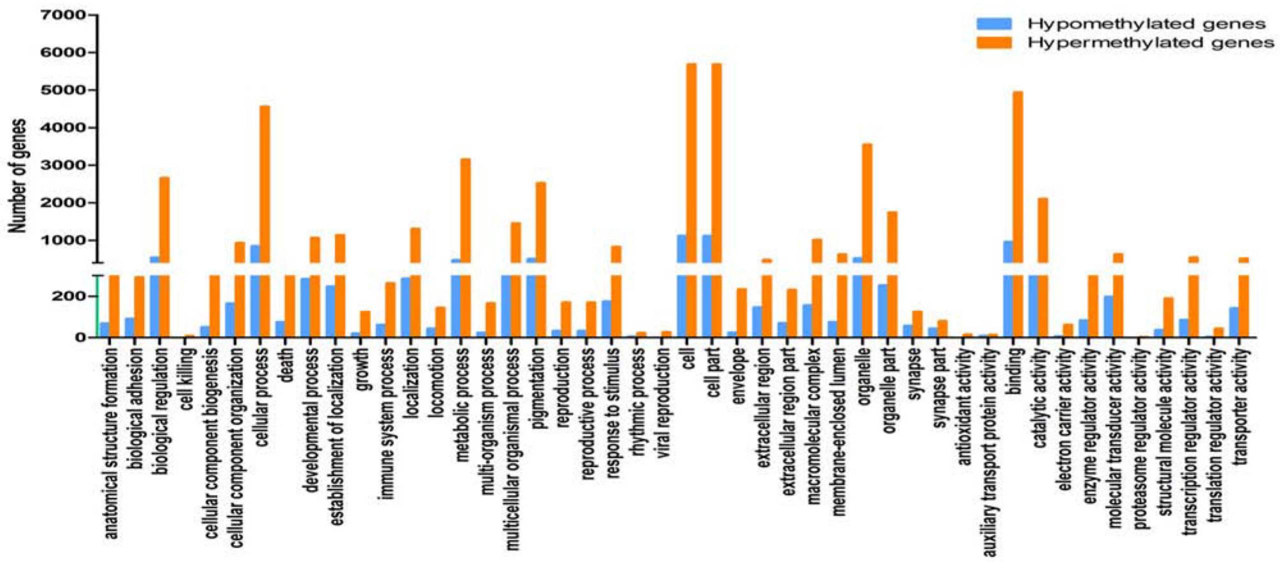

Supplementary Figure S3: Go enrichment analysis for DMR related genes. The horizontal axis indicates Go items (biological process, cellular component and molecular function), and the left Vertical axis indicates the number of genes involved.

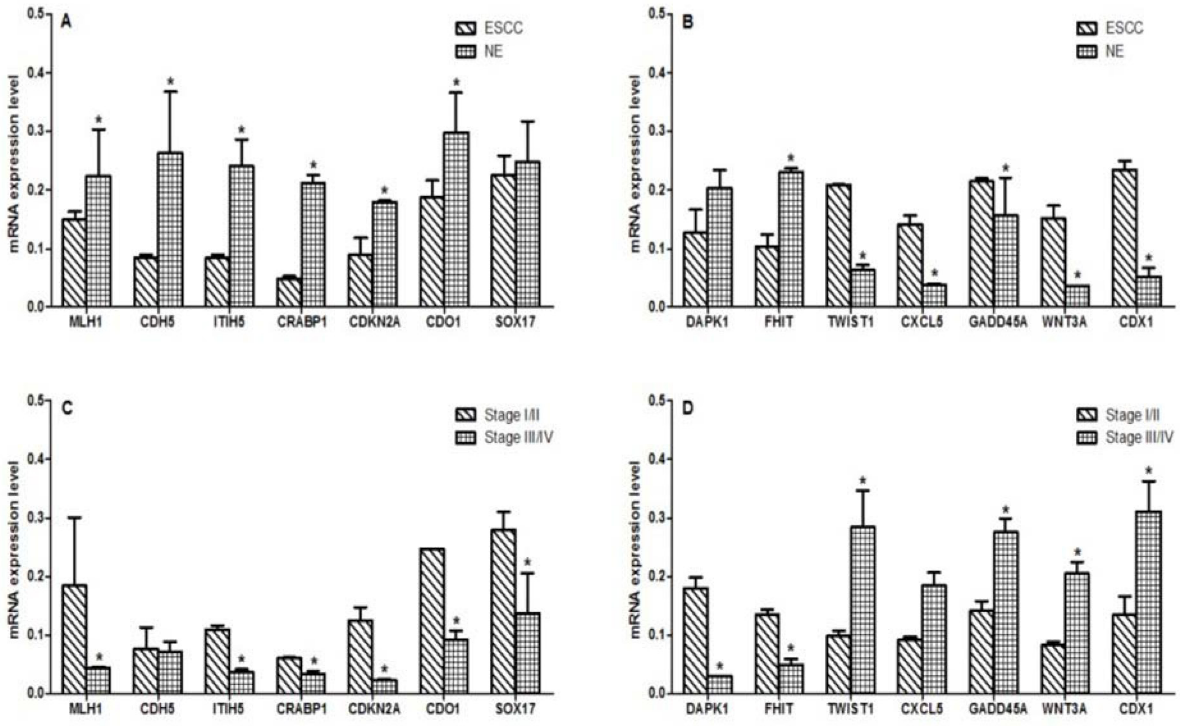

Supplementary Figure S4: Expression level of 14 cancer-related genes between ESCC group and NE group. A and B. The expression of MLH1, CDH5, ITIH5, CRABP1, CDKN2A, CDO1 and FHIT were significantly down-regulated in ESCC samples compared to NE samples. In contrast, the mRNA expression of TWIST1, CXCL5, GADD45A, WNT3A and CDX1 in patients with ESCC were significantly up-regulated than that in NE samples. **C and D.** gene expression in ESCC samples grouped according to TNM stages. mRNA levels were determined by real-time RT-PCR and normalized to β-actin. Data are presented as mean±SD; *, $p<0.05$.

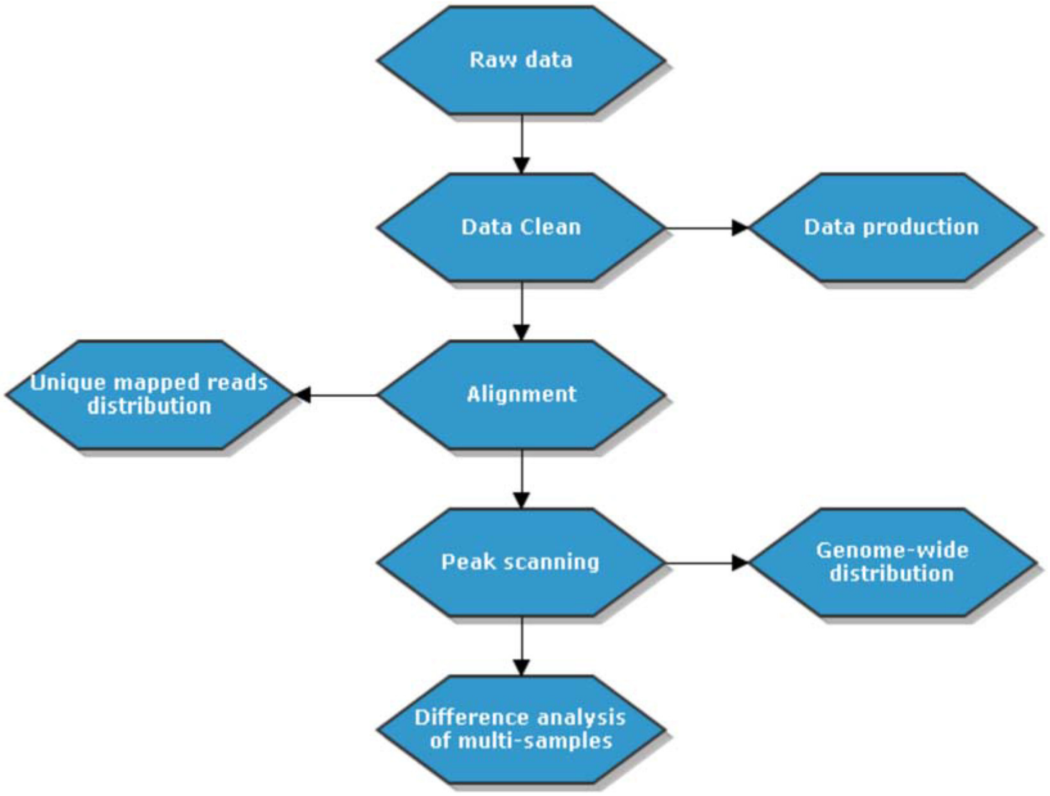

Supplementary Figure S5: Pipeline of Bioinformatics. Sequence data was filtered firstly to get clean data. Then the production of data was estimated. All of the clean data was mapped to reference genome. Only the uniquely mapped reads were used for further analysis. The distribution of uniquely mapped reads was analyzed. Meanwhile, read rich region (Peak) was scanned from the whole genome. After peak scanning, the distribution of peak was analyzed. Finally, the difference of multi-samples was analyzed.

Supplementary Table S1: DMRs CpG sites and primers for bisulfite sequencing

Gene DMRs	Starting	Ending	Primer	CG sites
MLH1	37,034,328	37,034,839	F: GGGAGGTTATAAGAGTAGGGTTAA	-1, -16, -27, -46, -52, -54, -64, -71, -88, -102, -111, -142, -148, -180, -187, -203, -231, -243
			R:TCTCAACTCTATAAATTACTAAATCTCTTC	
CDH5	66,398,524	66,399,564	F: ATAGGTTAGTGTATTTTTATGGGTA	-1809, -1839, -1896, -1956, -1975
			R: TCTAAATAAAATCCAACACATTCAAC	
CDX1	149,544,760	149,546,056	F: GGTAGTTTTTTAATTGTAAGGAAGATT	-416, -437, -450, -471, -484, -494, -552
			R: TCTAAACCAACTTCACACAATAACC	
TWIST1	19,158,305	19,159,010	F: GTTTGGGTTTTTGTTTTTAGTTTGA	-1343, -1353, -1356, -1370, -1372, -1398, -1418, -1424, -1427, -1430, -1453, -1480, -1482
			R: TTCAAATCCATCCCTTACATAACA	

Supplementary Table S2: Primers for qRT-PCR validation

Gene	Forward	Reverse
MLH1	TGGGACGAAGAAAAGGAATG	TCCAGGAGTTTGGAATGGAG
CDH5	GTTGGAAAAACAATTCCTGTAACC	TTGTCATGCACCAGTTTGGC
ITIH5	ACTGTCGCTGGAGAACTGTG	GTAGTTGGGGAACAGGGTCT
CRABP1	CACTGCACGCAAACTCTTCT	GAAAGTAGGAGCAAGCCAGC
CDKN2A	GCTGCCCAACGCACCGAATA	ACCACCAGCGTGTCCAGGAA
CDO1	TCATGGAAGCCTACGAGAGC	GTCCTTCACCCCAACAGAGA
SOX17	GAATCCAGACCTGCACAACG	CCGGTACTTGTAGTTGGGGT
DAPK1	TAACCAACCCAACAAGCACG	GCCCTTATCCTGGACATCGA
TWIST1	GCCACTGAAAGGAAAGGCAT	TTTGCAGGCCAGTTTGATCC
CXCL5	GTCTTGATCCAGAAGCCCCT	CCTTCTTGTCTTCCCTGGGT
GADD45A	CTGAACGGTGATGGCATCTG	TTGAACTCACTCAGCCCCTT
WNT3A	CTTTGCAGTGACACGCTCAT	ACCATCCCACCAAACTCGAT
CDX1	TCGGACCAAGGACAAGTACC	AGATCTTCACCTGCCGTTCA
FHIT	GATGAAGTGGCCGATTTGTT	CTTCCTGGGAAGAACATGGA
β-actin	CGCGAGAAGATGACCCAGAT	ATCACGATGCCAGTGGTACG

Supplementary 2: Differentially expressed gene identified by RNA-Seq

See Supplemenatry Table 2

Supplementary 3: DEGs related to many biological process and molecular functions

Biological Process		
**Gene Ontology term**	**Cluster frequency**	**Genome frequency of use**
anatomical structure development	983 out of 4631 genes, 21.2%	2398 out of 14370 genes, 16.7%
system development	875 out of 4631 genes, 18.9%	2104 out of 14370 genes, 14.6%
developmental process	1149 out of 4631 genes, 24.8%	2975 out of 14370 genes, 20.7%
organ development	597 out of 4631 genes, 12.9%	1417 out of 14370 genes, 9.9%
cell adhesion	323 out of 4631 genes, 7.0%	690 out of 14370 genes, 4.8%
biological adhesion	323 out of 4631 genes, 7.0%	691 out of 14370 genes, 4.8%
muscle contraction	91 out of 4631 genes, 2.0%	138 out of 14370 genes, 1.0%
muscle system process	98 out of 4631 genes, 2.1%	153 out of 14370 genes, 1.1%
multicellular organismal development	1041 out of 4631 genes, 22.5%	2679 out of 14370 genes, 18.6%
anatomical structure morphogenesis	450 out of 4631 genes, 9.7%	1065 out of 14370 genes, 7.4%
tissue development	290 out of 4631 genes, 6.3%	638 out of 14370 genes, 4.4%
response to chemical stimulus	532 out of 4631 genes, 11.5%	1295 out of 14370 genes, 9.0%
muscle structure development	126 out of 4631 genes, 2.7%	231 out of 14370 genes, 1.6%
regulation of multicellular organismal process	380 out of 4631 genes, 8.2%	891 out of 14370 genes, 6.2%
regulation of cell proliferation	322 out of 4631 genes, 7.0%	739 out of 14370 genes, 5.1%
muscle organ development	94 out of 4631 genes, 2.0%	166 out of 14370 genes, 1.2%
cell differentiation	601 out of 4631 genes, 13.0%	1540 out of 14370 genes, 10.7%
nervous system development	420 out of 4631 genes, 9.1%	1031 out of 14370 genes, 7.2%
cellular developmental process	613 out of 4631 genes, 13.2%	1575 out of 14370 genes, 11.0%
response to external stimulus	237 out of 4631 genes, 5.1%	545 out of 14370 genes, 3.8%
positive regulation of biological process	729 out of 4631 genes, 15.7%	1929 out of 14370 genes, 13.4%
metal ion homeostasis	99 out of 4631 genes, 2.1%	194 out of 14370 genes, 1.4%
regulation of biological quality	542 out of 4631 genes, 11.7%	1400 out of 14370 genes, 9.7%
cytoskeleton organization	184 out of 4631 genes, 4.0%	411 out of 14370 genes, 2.9%
response to mechanical stimulus	33 out of 4631 genes, 0.7%	47 out of 14370 genes, 0.3%
cellular metal ion homeostasis	94 out of 4631 genes, 2.0%	185 out of 14370 genes, 1.3%
response to wounding	214 out of 4631 genes, 4.6%	495 out of 14370 genes, 3.4%
positive regulation of cellular process	661 out of 4631 genes, 14.3%	1754 out of 14370 genes, 12.2%
calcium ion homeostasis	91 out of 4631 genes, 2.0%	179 out of 14370 genes, 1.2%
regulation of system process	134 out of 4631 genes, 2.9%	287 out of 14370 genes, 2.0%
cellular process	3102 out of 4631 genes, 67.0%	9208 out of 14370 genes, 64.1%
chemotaxis	115 out of 4631 genes, 2.5%	241 out of 14370 genes, 1.7%
taxis	115 out of 4631 genes, 2.5%	241 out of 14370 genes, 1.7%
cardiovascular system development	162 out of 4631 genes, 3.5%	364 out of 14370 genes, 2.5%

(*Continued*)

Biological Process		
**Gene Ontology term**	**Cluster frequency**	**Genome frequency of use**
circulatory system development	162 out of 4631 genes, 3.5%	364 out of 14370 genes, 2.5%
cellular calcium ion homeostasis	87 out of 4631 genes, 1.9%	173 out of 14370 genes, 1.2%
actin filament-based process	110 out of 4631 genes, 2.4%	232 out of 14370 genes, 1.6%
inflammatory response	139 out of 4631 genes, 3.0%	307 out of 14370 genes, 2.1%
positive regulation of cell proliferation	171 out of 4631 genes, 3.7%	392 out of 14370 genes, 2.7%
**Molecular Function**		
**Gene Ontology term**	**Cluster frequency**	**Genome frequency of use**
protein binding	2811 out of 4911 genes, 57.2%	8022 out of 15375 genes, 52.2%
calcium ion binding	399 out of 4911 genes, 8.1%	915 out of 15375 genes, 6.0%
binding	4128 out of 4911 genes, 84.1%	12393 out of 15375 genes, 80.6%
cytoskeletal protein binding	236 out of 4911 genes, 4.8%	494 out of 15375 genes, 3.2%
structural constituent of muscle	36 out of 4911 genes, 0.7%	42 out of 15375 genes, 0.3%
actin binding	163 out of 4911 genes, 3.3%	322 out of 15375 genes, 2.1%
calmodulin binding	76 out of 4911 genes, 1.5%	140 out of 15375 genes, 0.9%
