# Supplementary material for: Genome-wide profiling of DNA methylation and gene expression in esophageal squamous cell carcinoma
Source: Oncotarget. 2015 Dec 14;7(4):4507–21. doi: 10.18632/oncotarget.6607 (PMC4826222; doi:10.18632/oncotarget.6607)
Supplement: Supplementary file 2 [file oncotarget-07-4507-s002.docx]

| **Supplementary 2. Differentially expressed gene identified by RNA-Seq.** | | | | | |
| --- | --- | --- | --- | --- | --- |
| **geneID** | **NE-RPKM** | **ESCC-RPKM** | **log2 Ratio(ESCC/NE)** | **Up/Down-Regulation(ESCC/NE)** | **P-value** |
| 8581 | 0 | 263.4694572 | 18.0072762 | Up | 0 |
| 374918 | 0 | 129.4734704 | 16.98229699 | Up | 0 |
| 3963 | 0 | 123.7916756 | 16.91755478 | Up | 6.64E-241 |
| 2877 | 0 | 111.0186445 | 16.76044246 | Up | 0 |
| 1308 | 0 | 94.55959851 | 16.52893629 | Up | 0 |
| 8277 | 0 | 70.90845846 | 16.11367011 | Up | 0 |
| 3854 | 0 | 57.01367161 | 15.79902029 | Up | 0 |
| 2709 | 0 | 48.25064821 | 15.5582607 | Up | 1.76E-243 |
| 5268 | 0 | 45.48237493 | 15.47301997 | Up | 0 |
| 2172 | 0 | 45.42015446 | 15.47104499 | Up | 2.92E-133 |
| 11247 | 0 | 41.27493946 | 15.33297848 | Up | 5.23E-293 |
| 10804 | 0 | 37.29552933 | 15.18671508 | Up | 0 |
| 2706 | 0 | 37.007117 | 15.17551513 | Up | 0 |
| 4321 | 0 | 36.3641121 | 15.15022773 | Up | 3.13E-261 |
| 338442 | 0 | 35.89693152 | 15.13157291 | Up | 7.30E-292 |
| 171177 | 0 | 34.00587607 | 15.05349644 | Up | 2.62E-228 |
| 4102 | 0 | 33.94960404 | 15.05110713 | Up | 1.34E-230 |
| 158511 | 0 | 31.8208473 | 14.95768463 | Up | 4.51E-110 |
| 4605 | 0 | 30.62749567 | 14.90253979 | Up | 0 |
| 4105 | 0 | 26.35056659 | 14.68554636 | Up | 4.48E-183 |
| 120071 | 0 | 23.81548374 | 14.53961223 | Up | 3.50E-237 |
| 30848 | 0 | 23.35103869 | 14.5111991 | Up | 2.56E-90 |
| 26499 | 0 | 22.16595993 | 14.43605822 | Up | 8.02E-128 |
| 53836 | 0 | 21.59290975 | 14.39827005 | Up | 1.54E-129 |
| 1178 | 0 | 21.28794322 | 14.37774895 | Up | 2.18E-53 |
| 8843 | 0 | 21.21496366 | 14.37279459 | Up | 8.82E-179 |
| 1515 | 0 | 21.11072426 | 14.36568845 | Up | 1.13E-124 |
| 653499 | 0 | 20.56963839 | 14.32822881 | Up | 3.22E-40 |
| 4103 | 0 | 17.47287754 | 14.0928296 | Up | 3.11E-119 |
| 116211 | 0 | 16.94078107 | 14.04821277 | Up | 7.57E-69 |
| 10563 | 0 | 16.8156701 | 14.03751865 | Up | 2.68E-80 |
| 84648 | 0 | 16.72840869 | 14.03001259 | Up | 8.61E-41 |
| 153571 | 0 | 16.62503089 | 14.0210694 | Up | 7.92E-59 |
| 79412 | 0 | 15.82671745 | 13.95007444 | Up | 2.15E-128 |
| 10911 | 0 | 15.31455425 | 13.90261576 | Up | 8.69E-39 |
| 10053 | 0 | 14.99411653 | 13.8721089 | Up | 8.96E-104 |
| 8038 | 0 | 12.59912026 | 13.62103538 | Up | 6.97E-302 |
| 332 | 0 | 11.74307737 | 13.51952291 | Up | 3.03E-125 |
| 3237 | 0 | 11.50864207 | 13.49043 | Up | 7.64E-67 |
| 4316 | 0 | 11.39893801 | 13.4766118 | Up | 8.22E-51 |
| 23624 | 0 | 11.0936554 | 13.4374472 | Up | 5.42E-70 |
| 55165 | 0 | 10.94149898 | 13.41752278 | Up | 4.43E-114 |
| 113828 | 0 | 10.87513884 | 13.4087462 | Up | 3.57E-89 |
| 7476 | 0 | 10.37613081 | 13.34098095 | Up | 1.45E-70 |
| 389493 | 0 | 10.099682 | 13.30202225 | Up | 1.79E-24 |
| 2161 | 0 | 10.09274055 | 13.30103035 | Up | 5.13E-82 |
| 244 | 0 | 9.627403553 | 13.23293105 | Up | 1.95E-77 |
| 83959 | 0 | 8.669298143 | 13.08169948 | Up | 4.59E-106 |
| 8318 | 0 | 8.624451842 | 13.07421705 | Up | 2.06E-65 |
| 1907 | 0 | 8.288078524 | 13.01682196 | Up | 4.45E-41 |
| 81607 | 0 | 8.137988002 | 12.99045644 | Up | 1.20E-112 |
| 4110 | 0 | 8.004153717 | 12.96653316 | Up | 5.67E-60 |
| 222 | 0 | 7.673875676 | 12.90573968 | Up | 1.38E-80 |
| 51361 | 0 | 7.578766001 | 12.88774725 | Up | 4.65E-175 |
| 8339 | 0 | 7.223141721 | 12.81841076 | Up | 2.62E-13 |
| 11202 | 0 | 7.17452222 | 12.80866704 | Up | 4.62E-33 |
| 401138 | 0 | 7.098536592 | 12.79330592 | Up | 1.76E-28 |
| 83540 | 0 | 7.0725977 | 12.78802449 | Up | 5.78E-56 |
| 128218 | 0 | 7.070898454 | 12.78767783 | Up | 3.13E-46 |
| 7272 | 0 | 7.023580527 | 12.77799097 | Up | 1.37E-82 |
| 3226 | 0 | 6.994873146 | 12.77208218 | Up | 1.56E-54 |
| 9833 | 0 | 6.813644588 | 12.73421098 | Up | 5.52E-66 |
| 3161 | 0 | 6.710624786 | 12.71223138 | Up | 1.90E-83 |
| 90737 | 0 | 6.697822323 | 12.70947639 | Up | 3.53E-20 |
| 3225 | 0 | 6.621887932 | 12.69302688 | Up | 8.61E-41 |
| 22943 | 0 | 6.582135641 | 12.68434004 | Up | 2.24E-47 |
| 440585 | 0 | 6.485205139 | 12.66293649 | Up | 7.01E-14 |
| 165545 | 0 | 6.336889604 | 12.62955917 | Up | 2.85E-66 |
| 6999 | 0 | 6.199575646 | 12.59795375 | Up | 6.16E-42 |
| 8345 | 0 | 6.174047982 | 12.59200098 | Up | 5.11E-11 |
| 728643 | 0 | 6.051592233 | 12.56309906 | Up | 1.35E-13 |
| 4111 | 0 | 5.868107045 | 12.51867947 | Up | 8.69E-39 |
| 128876 | 0 | 5.755532727 | 12.49073375 | Up | 2.78E-72 |
| 7012 | 0 | 5.656501831 | 12.4656944 | Up | 9.88E-11 |
| 6861 | 0 | 5.431658651 | 12.4071771 | Up | 1.20E-39 |
| 126353 | 0 | 5.368815838 | 12.3903882 | Up | 7.85E-61 |
| 5624 | 0 | 5.357584372 | 12.38736695 | Up | 1.21E-37 |
| 4312 | 0.1520245 | 798.0799918 | 12.35801377 | Up | 0 |
| 93273 | 0 | 5.239706959 | 12.35527041 | Up | 9.61E-17 |
| 973 | 0 | 5.214567552 | 12.3483319 | Up | 1.77E-26 |
| 2352 | 0 | 5.175484008 | 12.33747807 | Up | 6.88E-18 |
| 127534 | 0 | 5.066068911 | 12.30665099 | Up | 1.29E-23 |
| 642273 | 0 | 5.055383491 | 12.30360482 | Up | 6.82E-20 |
| 154197 | 0 | 5.011054569 | 12.29089853 | Up | 1.68E-38 |
| 6036 | 0 | 4.85920443 | 12.24650441 | Up | 9.70E-15 |
| 388555 | 0 | 4.734421235 | 12.20897236 | Up | 5.06E-13 |
| 4100 | 0 | 4.691449567 | 12.19581804 | Up | 3.34E-32 |
| 79369 | 0 | 4.652429774 | 12.18376866 | Up | 3.40E-28 |
| 221150 | 0 | 4.643838029 | 12.18110194 | Up | 2.18E-53 |
| 389336 | 0 | 4.572314132 | 12.15870881 | Up | 1.91E-10 |
| 58495 | 0 | 4.546715399 | 12.15060899 | Up | 1.76E-28 |
| 995 | 0 | 4.445655117 | 12.11818032 | Up | 1.21E-37 |
| 57549 | 0 | 4.379772539 | 12.09664023 | Up | 2.80E-70 |
| 90226 | 0 | 4.3683757 | 12.09288122 | Up | 3.43E-26 |
| 9407 | 0 | 4.347617331 | 12.08600925 | Up | 3.10E-48 |
| 554202 | 0 | 4.343422954 | 12.08461673 | Up | 2.35E-37 |
| 9914 | 0 | 4.241934739 | 12.05050671 | Up | 4.14E-57 |
| 112714 | 0 | 4.212362282 | 12.0404138 | Up | 3.43E-26 |
| 9787 | 0 | 4.209628757 | 12.03947729 | Up | 1.14E-51 |
| 113115 | 0 | 4.187100304 | 12.03173576 | Up | 3.37E-30 |
| 346689 | 0 | 4.154242129 | 12.02036959 | Up | 6.63E-26 |
| 1300 | 0.02373939 | 97.78964295 | 12.00818319 | Up | 0 |
| 79977 | 0 | 4.112063416 | 12.0056468 | Up | 3.64E-85 |
| 3229 | 0 | 4.015536215 | 11.97137694 | Up | 1.68E-38 |
| 3861 | 1.29055684 | 5128.572078 | 11.9563478 | Up | 0 |
| 4109 | 0 | 3.843438532 | 11.90818188 | Up | 9.26E-25 |
| 8347 | 0 | 3.827455544 | 11.90216991 | Up | 2.69E-07 |
| 1828 | 0 | 3.81437733 | 11.89723185 | Up | 2.65E-82 |
| 90527 | 0 | 3.801506393 | 11.8923555 | Up | 9.10E-29 |
| 130576 | 0 | 3.753295221 | 11.87394206 | Up | 6.69E-24 |
| 5275 | 0 | 3.714333679 | 11.85888771 | Up | 4.33E-47 |
| 64711 | 0 | 3.63754004 | 11.82874741 | Up | 6.94E-16 |
| 50964 | 0 | 3.612712859 | 11.81886688 | Up | 2.39E-33 |
| 54857 | 0 | 3.600657022 | 11.81404447 | Up | 2.41E-31 |
| 701 | 0 | 3.584020371 | 11.80736312 | Up | 2.18E-53 |
| 84141 | 0 | 3.536348238 | 11.78804463 | Up | 1.77E-26 |
| 5017 | 0 | 3.531162591 | 11.78592754 | Up | 3.19E-42 |
| 89780 | 0 | 3.530049603 | 11.78547274 | Up | 2.30E-41 |
| 8193 | 0 | 3.44846766 | 11.75173972 | Up | 3.34E-32 |
| 55872 | 0 | 3.302371943 | 11.6892869 | Up | 9.26E-25 |
| 9156 | 0 | 3.184759024 | 11.6369685 | Up | 4.45E-41 |
| 352909 | 0 | 3.160275944 | 11.62583482 | Up | 1.27E-27 |
| 3868 | 0.27292692 | 854.7049097 | 11.61269597 | Up | 0 |
| 26476 | 0 | 3.103230244 | 11.59955503 | Up | 1.89E-12 |
| 3972 | 0 | 3.066037212 | 11.58215949 | Up | 5.21E-07 |
| 3852 | 0.8478353 | 2571.43275 | 11.56650076 | Up | 0 |
| 89858 | 0 | 2.982719558 | 11.54241262 | Up | 1.28E-25 |
| 3206 | 0 | 2.97675884 | 11.53952663 | Up | 9.01E-31 |
| 374897 | 0 | 2.976580724 | 11.53944031 | Up | 7.07E-12 |
| 11012 | 0 | 2.915522658 | 11.50953882 | Up | 2.60E-15 |
| 100131726 | 0 | 2.895380878 | 11.49953743 | Up | 7.01E-14 |
| 50604 | 0 | 2.894258717 | 11.49897817 | Up | 9.70E-15 |
| 388611 | 0 | 2.889000685 | 11.49635483 | Up | 3.56E-18 |
| 260436 | 0 | 2.845073226 | 11.47425007 | Up | 1.01E-06 |
| 55635 | 0 | 2.829144901 | 11.46615035 | Up | 5.67E-60 |
| 81623 | 0 | 2.823750758 | 11.46339704 | Up | 2.72E-05 |
| 6349 | 0 | 2.796217383 | 11.44926081 | Up | 2.67E-09 |
| 1852 | 0 | 2.786896658 | 11.44444379 | Up | 9.18E-27 |
| 134285 | 0 | 2.763528586 | 11.43229582 | Up | 1.88E-14 |
| 29986 | 0 | 2.741708674 | 11.42085957 | Up | 6.94E-16 |
| 118932 | 0 | 2.708369027 | 11.40320861 | Up | 4.41E-43 |
| 3872 | 0.44658405 | 1194.827122 | 11.38558255 | Up | 0 |
| 3552 | 0 | 2.663600789 | 11.37916216 | Up | 2.41E-31 |
| 10874 | 0 | 2.612798427 | 11.35138011 | Up | 5.16E-09 |
| 286887 | 0.03412083 | 88.17258891 | 11.33546161 | Up | 0 |
| 59082 | 0 | 2.572519992 | 11.32896657 | Up | 5.25E-05 |
| 5003 | 0 | 2.545896241 | 11.31395791 | Up | 3.59E-16 |
| 4113 | 0 | 2.535237094 | 11.30790496 | Up | 9.61E-17 |
| 4751 | 0 | 2.532261464 | 11.30621066 | Up | 6.76E-22 |
| 2150 | 0 | 2.503788542 | 11.28989701 | Up | 4.71E-29 |
| 3848 | 0.0637312 | 158.3256082 | 11.27860713 | Up | 0 |
| 123099 | 0 | 2.438248486 | 11.25162945 | Up | 3.63E-14 |
| 115749 | 0 | 2.434195036 | 11.24922905 | Up | 6.94E-16 |
| 3046 | 0 | 2.411737493 | 11.23585717 | Up | 1.93E-08 |
| 8294 | 0 | 2.36393729 | 11.20697605 | Up | 0.0003796 |
| 55040 | 0 | 2.301533038 | 11.16837944 | Up | 3.28E-36 |
| 6783 | 0 | 2.293107709 | 11.16308841 | Up | 9.61E-17 |
| 56729 | 0 | 2.28728242 | 11.1594188 | Up | 5.25E-05 |
| 25818 | 0 | 2.285038552 | 11.15800279 | Up | 9.70E-15 |
| 3227 | 0 | 2.254270096 | 11.13844467 | Up | 9.52E-19 |
| 8739 | 0 | 2.196053652 | 11.10069759 | Up | 1.01E-06 |
| 1833 | 0 | 2.181425541 | 11.09105552 | Up | 7.01E-14 |
| 283869 | 0 | 2.152206687 | 11.07160092 | Up | 2.67E-09 |
| 9635 | 0.05822758 | 124.2212624 | 11.05892196 | Up | 0 |
| 606 | 0 | 2.128118729 | 11.05536293 | Up | 2.72E-05 |
| 80117 | 0 | 2.097090559 | 11.03417345 | Up | 2.64E-11 |
| 11227 | 0 | 2.095153619 | 11.03284031 | Up | 1.77E-26 |
| 2792 | 0 | 2.089148402 | 11.02869926 | Up | 7.27E-06 |
| 8601 | 0 | 2.045859144 | 10.9984911 | Up | 1.33E-17 |
| 55879 | 0 | 2.040865861 | 10.99496565 | Up | 9.61E-17 |
| 162461 | 0 | 2.03597951 | 10.99150733 | Up | 3.49E-22 |
| 641 | 0 | 2.028245665 | 10.98601669 | Up | 8.77E-37 |
| 2520 | 0 | 2.010705281 | 10.97348592 | Up | 0.0003796 |
| 50487 | 0 | 1.981423166 | 10.95232131 | Up | 9.44E-21 |
| 642587 | 0.08775572 | 173.6210122 | 10.95016082 | Up | 0 |
| 8456 | 0 | 1.972869429 | 10.94607976 | Up | 1.31E-21 |
| 55388 | 0 | 1.966027881 | 10.94106807 | Up | 1.22E-35 |
| 54830 | 0 | 1.945337378 | 10.92580467 | Up | 3.63E-14 |
| 6706 | 0 | 1.90924057 | 10.89878318 | Up | 1.41E-05 |
| 1469 | 0.41106625 | 779.8064057 | 10.88952935 | Up | 0 |
| 10017 | 0 | 1.889994959 | 10.88416667 | Up | 2.69E-07 |
| 3007 | 0 | 1.876140707 | 10.87355232 | Up | 1.95E-06 |
| 387695 | 0 | 1.848610381 | 10.85222547 | Up | 1.01E-06 |
| 29851 | 0 | 1.793913033 | 10.80889424 | Up | 2.55E-19 |
| 245 | 0 | 1.790602789 | 10.80622962 | Up | 3.53E-20 |
| 84667 | 0 | 1.78196377 | 10.79925229 | Up | 1.38E-09 |
| 158228 | 0 | 1.774665966 | 10.79333179 | Up | 9.79E-13 |
| 3904 | 0 | 1.772671144 | 10.79170921 | Up | 1.41E-05 |
| 100129128 | 0 | 1.727205366 | 10.75422392 | Up | 5.25E-05 |
| 153328 | 0 | 1.704984011 | 10.73554249 | Up | 9.97E-09 |
| 27299 | 0 | 1.704109839 | 10.73480261 | Up | 4.97E-17 |
| 124056 | 0 | 1.691699155 | 10.72425731 | Up | 9.88E-11 |
| 81832 | 0 | 1.682255414 | 10.71618105 | Up | 2.57E-17 |
| 1437 | 0 | 1.679878612 | 10.71414127 | Up | 7.27E-06 |
| 353145 | 0 | 1.670102996 | 10.70572136 | Up | 0.00010157 |
| 652995 | 0 | 1.64951777 | 10.6878286 | Up | 7.14E-10 |
| 147920 | 0 | 1.623342237 | 10.66475147 | Up | 1.95E-06 |
| 340206 | 0 | 1.605464019 | 10.64877462 | Up | 7.20E-08 |
| 731220 | 0 | 1.601935526 | 10.64560037 | Up | 1.37E-11 |
| 124739 | 0 | 1.576733001 | 10.62272266 | Up | 6.63E-26 |
| 3853 | 0.66945076 | 1048.993152 | 10.6137397 | Up | 0 |
| 729533 | 0 | 1.56160828 | 10.60881689 | Up | 3.66E-12 |
| 1301 | 0.02131912 | 32.61907601 | 10.57935246 | Up | 0 |
| 79025 | 0 | 1.508933869 | 10.55931386 | Up | 2.69E-07 |
| 56286 | 0 | 1.501655886 | 10.55233853 | Up | 0.00019636 |
| 3381 | 0 | 1.466007622 | 10.51767689 | Up | 7.14E-10 |
| 3850 | 0 | 1.451450674 | 10.50327983 | Up | 7.01E-14 |
| 157313 | 0 | 1.449923903 | 10.50176147 | Up | 6.76E-22 |
| 145873 | 0 | 1.447807651 | 10.49965423 | Up | 7.14E-10 |
| 7546 | 0 | 1.440368204 | 10.49222194 | Up | 6.94E-16 |
| 256764 | 0 | 1.436021558 | 10.48786169 | Up | 6.16E-42 |
| 145957 | 0 | 1.424879495 | 10.4766242 | Up | 0.00019636 |
| 145200 | 0 | 1.366651246 | 10.41642942 | Up | 0.0003796 |
| 221393 | 0 | 1.36002401 | 10.40941641 | Up | 1.33E-17 |
| 56300 | 0 | 1.358930053 | 10.40825548 | Up | 5.21E-07 |
| 5148 | 0 | 1.353737305 | 10.40273209 | Up | 3.76E-06 |
| 6538 | 0 | 1.317915817 | 10.3640425 | Up | 5.11E-11 |
| 23596 | 0 | 1.301572615 | 10.34604009 | Up | 3.63E-14 |
| 57822 | 0 | 1.301572615 | 10.34604009 | Up | 5.02E-15 |
| 6702 | 0 | 1.290054273 | 10.33321605 | Up | 0.0003796 |
| 284415 | 0 | 1.283742853 | 10.32614053 | Up | 7.27E-06 |
| 57115 | 0 | 1.272459425 | 10.31340394 | Up | 7.14E-10 |
| 266695 | 0 | 1.268997893 | 10.30947396 | Up | 1.29E-23 |
| 200844 | 0 | 1.257183428 | 10.29597944 | Up | 2.62E-13 |
| 84634 | 0 | 1.255917453 | 10.29452593 | Up | 9.97E-09 |
| 348378 | 0 | 1.253089968 | 10.29127428 | Up | 0.0003796 |
| 93429 | 0 | 1.250530552 | 10.28832459 | Up | 9.97E-09 |
| 121268 | 0 | 1.238539787 | 10.2744245 | Up | 1.95E-06 |
| 79413 | 0 | 1.23313144 | 10.26811087 | Up | 2.64E-11 |
| 2016 | 0 | 1.199255207 | 10.22792299 | Up | 5.11E-11 |
| 1044 | 0 | 1.19286339 | 10.22021312 | Up | 5.16E-09 |
| 128710 | 0 | 1.191489359 | 10.21855035 | Up | 2.69E-07 |
| 146909 | 0 | 1.174292566 | 10.19757617 | Up | 5.06E-13 |
| 124359 | 0 | 1.17403597 | 10.19726089 | Up | 5.11E-11 |
| 390539 | 0 | 1.173091198 | 10.19609946 | Up | 5.25E-05 |
| 26257 | 0 | 1.172353814 | 10.19519232 | Up | 5.16E-09 |
| 23532 | 0 | 1.162404829 | 10.18289689 | Up | 2.62E-13 |
| 157570 | 0 | 1.150407243 | 10.16792895 | Up | 6.94E-16 |
| 9622 | 0 | 1.136339566 | 10.1501783 | Up | 1.01E-06 |
| 2810 | 0.47514883 | 539.7042609 | 10.1495739 | Up | 0 |
| 51438 | 0 | 1.134302239 | 10.14758939 | Up | 1.38E-09 |
| 284422 | 0 | 1.123824813 | 10.13420144 | Up | 0.00010157 |
| 3009 | 0 | 1.107160503 | 10.11264867 | Up | 0.0003796 |
| 3914 | 0.12693711 | 138.6176346 | 10.09278123 | Up | 0 |
| 51806 | 0 | 1.081672219 | 10.07904767 | Up | 0.00019636 |
| 340393 | 0 | 1.044702165 | 10.02887599 | Up | 0.00019636 |
| 131540 | 0 | 1.035806479 | 10.01653877 | Up | 3.76E-06 |
| 221188 | 0 | 1.027411575 | 10.00479852 | Up | 6.94E-16 |
| 135886 | 0 | 1.024188287 | 10.00026525 | Up | 0.0003796 |
| 10633 | 0 | 1.019698747 | 9.99392728 | Up | 7.20E-08 |
| 149708 | 0 | 1.012334256 | 9.983470008 | Up | 0.00010157 |
| 219790 | 0 | 1.009742827 | 9.979772182 | Up | 4.75E-27 |
| 388585 | 0 | 1.006123617 | 9.974591856 | Up | 7.27E-06 |
| 9721 | 0 | 0.999562074 | 9.965152353 | Up | 7.20E-08 |
| 134121 | 0 | 0.994574006 | 9.957934916 | Up | 0.00010157 |
| 10309 | 0 | 0.967090267 | 9.917506745 | Up | 3.76E-06 |
| 5313 | 0 | 0.958108005 | 9.904044486 | Up | 3.66E-12 |
| 85411 | 0 | 0.954657059 | 9.898838757 | Up | 1.95E-06 |
| 147111 | 0 | 0.953429827 | 9.896982949 | Up | 5.16E-09 |
| 6092 | 0 | 0.946598266 | 9.886608469 | Up | 6.69E-24 |
| 8710 | 0 | 0.945751198 | 9.885316888 | Up | 5.16E-09 |
| 1232 | 0 | 0.936864607 | 9.871696759 | Up | 7.20E-08 |
| 100137047 | 0 | 0.922633753 | 9.849614261 | Up | 7.27E-06 |
| 50805 | 0.45715157 | 419.7053048 | 9.842488394 | Up | 0 |
| 389816 | 0 | 0.914912968 | 9.837490702 | Up | 5.25E-05 |
| 124590 | 0 | 0.900610762 | 9.814759907 | Up | 2.62E-13 |
| 57082 | 0 | 0.891539134 | 9.800154316 | Up | 1.27E-27 |
| 338376 | 0 | 0.891294291 | 9.799758055 | Up | 7.27E-06 |
| 144455 | 0 | 0.888724029 | 9.795591686 | Up | 9.44E-21 |
| 91450 | 0 | 0.881710481 | 9.7841612 | Up | 0.0003796 |
| 810 | 0.23994651 | 209.931067 | 9.772987152 | Up | 0 |
| 133690 | 0 | 0.872040675 | 9.768251619 | Up | 0.0003796 |
| 84779 | 0 | 0.865425591 | 9.757265972 | Up | 7.27E-06 |
| 84830 | 0 | 0.863974926 | 9.754845633 | Up | 5.21E-07 |
| 10683 | 0 | 0.856427134 | 9.742186695 | Up | 9.97E-09 |
| 2707 | 0.07045791 | 58.32360392 | 9.693102566 | Up | 0 |
| 3821 | 0 | 0.792836111 | 9.630878863 | Up | 5.25E-05 |
| 122618 | 0 | 0.791442293 | 9.628340353 | Up | 1.01E-06 |
| 220359 | 0 | 0.778560222 | 9.604664826 | Up | 1.01E-06 |
| 1800 | 0 | 0.755751841 | 9.561768778 | Up | 7.27E-06 |
| 6372 | 0 | 0.74689399 | 9.544759679 | Up | 1.41E-05 |
| 27033 | 0 | 0.74375578 | 9.538685165 | Up | 1.95E-06 |
| 375519 | 0 | 0.735274355 | 9.522138856 | Up | 2.69E-07 |
| 3857 | 0 | 0.732383368 | 9.516455218 | Up | 2.69E-07 |
| 13 | 0 | 0.726157574 | 9.504138832 | Up | 2.72E-05 |
| 4322 | 0.1726024 | 124.5406042 | 9.494947902 | Up | 0 |
| 284366 | 0 | 0.709619562 | 9.470901971 | Up | 0.00010157 |
| 84658 | 0 | 0.696887206 | 9.44478136 | Up | 5.21E-07 |
| 113220 | 0 | 0.691399532 | 9.433375817 | Up | 3.76E-06 |
| 284836 | 0 | 0.68028862 | 9.410003146 | Up | 9.97E-09 |
| 255231 | 0 | 0.670455276 | 9.388997288 | Up | 9.97E-09 |
| 55789 | 0 | 0.669766492 | 9.38751439 | Up | 2.69E-07 |
| 83401 | 0 | 0.667167656 | 9.38190554 | Up | 0.0003796 |
| 285782 | 0 | 0.664710532 | 9.376582403 | Up | 3.73E-08 |
| 27293 | 0 | 0.661557464 | 9.369722666 | Up | 1.41E-05 |
| 84072 | 0 | 0.659444987 | 9.365108499 | Up | 1.41E-05 |
| 399815 | 0 | 0.655716507 | 9.356928403 | Up | 3.76E-06 |
| 81693 | 0 | 0.655384027 | 9.356196702 | Up | 0.00010157 |
| 84815 | 0 | 0.654356706 | 9.353933489 | Up | 7.27E-06 |
| 200407 | 0 | 0.647801049 | 9.339406995 | Up | 1.38E-09 |
| 6344 | 0 | 0.639719728 | 9.321296163 | Up | 2.72E-05 |
| 9333 | 0 | 0.633809273 | 9.307904958 | Up | 3.76E-06 |
| 646600 | 0 | 0.630904852 | 9.301278636 | Up | 1.41E-05 |
| 26150 | 0 | 0.627894327 | 9.294377967 | Up | 0.0003796 |
| 7490 | 0 | 0.625648639 | 9.289208865 | Up | 3.73E-08 |
| 284217 | 0 | 0.620552174 | 9.277408702 | Up | 6.69E-24 |
| 257101 | 0 | 0.617321797 | 9.269878922 | Up | 0.00010157 |
| 55503 | 0 | 0.604462196 | 9.239508303 | Up | 1.39E-07 |
| 50852 | 0 | 0.602025328 | 9.233680373 | Up | 0.00010157 |
| 2556 | 0 | 0.601948125 | 9.233495353 | Up | 2.69E-07 |
| 7498 | 0 | 0.599219717 | 9.226941286 | Up | 3.63E-14 |
| 131873 | 0 | 0.593775276 | 9.213773212 | Up | 1.82E-20 |
| 166012 | 0 | 0.586792944 | 9.196707714 | Up | 0.00010157 |
| 6656 | 0 | 0.585517574 | 9.193568662 | Up | 3.69E-10 |
| NM_001146317:6851017-6852762 | 0 | 0.584273078 | 9.190499004 | Up | 0.0003796 |
| 127343 | 0 | 0.581284857 | 9.183101516 | Up | 2.69E-07 |
| 253430 | 0 | 0.567853606 | 9.149375238 | Up | 0.00010157 |
| 3352 | 0 | 0.56562168 | 9.143693608 | Up | 5.21E-07 |
| 4051 | 0 | 0.56105272 | 9.131992532 | Up | 2.69E-07 |
| 3898 | 0.22008479 | 122.0791673 | 9.115541897 | Up | 0 |
| 5317 | 0.2152377 | 118.8197343 | 9.108627929 | Up | 0 |
| 202020 | 0 | 0.543727233 | 9.086739278 | Up | 3.76E-06 |
| 374946 | 0 | 0.534430267 | 9.061857906 | Up | 0.00019636 |
| 4950 | 0 | 0.524573206 | 9.035000308 | Up | 3.76E-06 |
| 5540 | 0 | 0.521693727 | 9.027059276 | Up | 0.00010157 |
| 136332 | 0 | 0.512916023 | 9.002578831 | Up | 3.76E-06 |
| 131578 | 0.0263016 | 13.48779004 | 9.002287942 | Up | 0 |
| 6318 | 0 | 0.508817218 | 8.99100368 | Up | 0.0003796 |
| 54854 | 0 | 0.507425624 | 8.987052565 | Up | 0.00010157 |
| 51352 | 0 | 0.498806272 | 8.962335795 | Up | 2.72E-05 |
| 84793 | 0 | 0.494057867 | 8.948536218 | Up | 1.41E-05 |
| 2977 | 0 | 0.493487247 | 8.946868993 | Up | 1.95E-06 |
| 340152 | 0 | 0.489181654 | 8.934226489 | Up | 2.72E-05 |
| 168400 | 0 | 0.481336288 | 8.910901382 | Up | 0.00010157 |
| 6278 | 0.35582045 | 170.3488751 | 8.903127287 | Up | 2.39E-289 |
| 84951 | 0.0575412 | 27.42252402 | 8.896550353 | Up | 0 |
| 54967 | 0 | 0.47220404 | 8.883266574 | Up | 0.00010157 |
| 2569 | 0 | 0.461170936 | 8.849157784 | Up | 1.95E-06 |
| 338324 | 0 | 0.459915353 | 8.845224548 | Up | 1.93E-08 |
| 247 | 0 | 0.456389366 | 8.834121368 | Up | 1.41E-05 |
| 25975 | 0.06557732 | 29.92633459 | 8.834002926 | Up | 1.20E-275 |
| 84985 | 0.05333055 | 23.8149128 | 8.802687386 | Up | 1.18E-269 |
| 85416 | 0 | 0.443956028 | 8.79427298 | Up | 9.97E-09 |
| 80157 | 0 | 0.44264008 | 8.789990278 | Up | 5.25E-05 |
| 3239 | 0 | 0.438518664 | 8.776494436 | Up | 0.00010157 |
| 1014 | 0 | 0.437379855 | 8.772742964 | Up | 1.41E-05 |
| 57116 | 0 | 0.436978816 | 8.771419531 | Up | 1.95E-06 |
| 11023 | 0 | 0.421693308 | 8.720050318 | Up | 3.73E-08 |
| 23359 | 0 | 0.418095983 | 8.707690374 | Up | 1.93E-08 |
| 140893 | 0 | 0.417547105 | 8.705795151 | Up | 2.72E-05 |
| 3363 | 0 | 0.414136741 | 8.693963391 | Up | 3.76E-06 |
| 286676 | 0 | 0.411177509 | 8.683617543 | Up | 5.25E-05 |
| 23650 | 0.38805526 | 153.9123629 | 8.631631302 | Up | 0 |
| 56961 | 0 | 0.396130796 | 8.629833053 | Up | 0.00010157 |
| 6273 | 1.70494215 | 669.1003273 | 8.616355948 | Up | 0 |
| 83416 | 0 | 0.392162139 | 8.615306447 | Up | 5.16E-09 |
| 153572 | 0.10226198 | 39.9154616 | 8.608534001 | Up | 0 |
| 3748 | 0 | 0.390143933 | 8.607862653 | Up | 1.41E-05 |
| 54551 | 0 | 0.382074542 | 8.577710322 | Up | 0.00019636 |
| 445582 | 0 | 0.381391045 | 8.575127161 | Up | 7.27E-06 |
| 3866 | 0.12741042 | 48.23533272 | 8.564463223 | Up | 0 |
| 57016 | 1.4690142 | 535.0303963 | 8.508628706 | Up | 0 |
| 79674 | 0 | 0.359172469 | 8.488532961 | Up | 5.25E-05 |
| 2304 | 0 | 0.358017085 | 8.483884624 | Up | 1.41E-05 |
| 54456 | 0 | 0.355277902 | 8.472804147 | Up | 3.76E-06 |
| 51744 | 0 | 0.355118472 | 8.472156597 | Up | 0.0003796 |
| 54845 | 0.04104182 | 14.51633063 | 8.46636631 | Up | 6.93E-213 |
| 138009 | 0 | 0.350845085 | 8.454690342 | Up | 2.72E-05 |
| 89958 | 0.04075272 | 14.26194882 | 8.451059057 | Up | 1.32E-210 |
| 9088 | 0.03644544 | 12.72054916 | 8.447206731 | Up | 1.60E-105 |
| 5122 | 0 | 0.331702716 | 8.373747016 | Up | 2.69E-07 |
| 7643 | 0 | 0.330161772 | 8.367029276 | Up | 1.41E-05 |
| 4948 | 0 | 0.32497864 | 8.344201087 | Up | 0.00010157 |
| 10024 | 0.02983292 | 9.577347158 | 8.326577026 | Up | 5.71E-97 |
| 1159 | 0.08780505 | 28.06538815 | 8.320272449 | Up | 4.49E-192 |
| 5349 | 0.59393603 | 184.8068149 | 8.281494684 | Up | 0 |
| 57111 | 0.1439679 | 44.13590752 | 8.260061832 | Up | 4.29E-184 |
| 283748 | 0 | 0.305056082 | 8.252930683 | Up | 5.25E-05 |
| 5266 | 0.28246867 | 83.95967148 | 8.215461834 | Up | 2.14E-178 |
| 11187 | 0.13902205 | 40.89412343 | 8.200435996 | Up | 0 |
| 63970 | 0.02942825 | 8.321433359 | 8.143486255 | Up | 2.75E-85 |
| 401827 | 0 | 0.276615053 | 8.111735858 | Up | 0.0003796 |
| 999 | 0.08110341 | 22.37353317 | 8.107814731 | Up | 0 |
| 4320 | 1.45082207 | 391.3905417 | 8.075594483 | Up | 0 |
| 81831 | 0.04276079 | 11.53279562 | 8.075237954 | Up | 1.37E-161 |
| 54626 | 0.07337021 | 19.36035772 | 8.043695485 | Up | 0 |
| 9547 | 4.94320179 | 1298.293311 | 8.03695484 | Up | 0 |
| 57402 | 0.89945399 | 223.9804115 | 7.960107363 | Up | 0 |
| 9982 | 0.39645984 | 97.86240394 | 7.947436184 | Up | 0 |
| 256076 | 0 | 0.244908959 | 7.936101743 | Up | 1.38E-09 |
| 84059 | 0 | 0.241288794 | 7.914617104 | Up | 4.93E-19 |
| 6382 | 0.7352506 | 176.6534037 | 7.90846977 | Up | 0 |
| 85320 | 0 | 0.239862004 | 7.906060831 | Up | 2.72E-05 |
| 7015 | 0 | 0.23611883 | 7.883369287 | Up | 0.00019636 |
| 3575 | 0.08658824 | 20.16138867 | 7.863208277 | Up | 4.24E-139 |
| 1832 | 0.46556527 | 106.9199766 | 7.843332258 | Up | 0 |
| 259266 | 0.01434654 | 3.280230764 | 7.83695021 | Up | 1.54E-136 |
| 2196 | 0.02149218 | 4.838812887 | 7.814697404 | Up | 1.12E-267 |
| 6334 | 0 | 0.22249722 | 7.797643498 | Up | 5.21E-07 |
| 6299 | 0 | 0.222008198 | 7.79446914 | Up | 2.72E-05 |
| 728378 | 0 | 0.219267184 | 7.776546102 | Up | 0.0003796 |
| 54207 | 0 | 0.203869126 | 7.671499497 | Up | 1.01E-06 |
| 6523 | 0 | 0.202627667 | 7.662687364 | Up | 0.00010157 |
| 548596 | 0.08780505 | 17.74060302 | 7.658535486 | Up | 2.51E-120 |
| 3576 | 2.26795174 | 450.5567229 | 7.634174991 | Up | 0 |
| 4319 | 0.3584743 | 71.1735451 | 7.633327593 | Up | 0 |
| 646 | 0.05080394 | 9.687854453 | 7.575093034 | Up | 1.47E-169 |
| 8564 | 0 | 0.188942146 | 7.561800742 | Up | 0.00019636 |
| 55765 | 0.11281067 | 21.28387689 | 7.559713606 | Up | 0 |
| 5618 | 0 | 0.185497285 | 7.53525426 | Up | 2.67E-09 |
| 11130 | 0.04219481 | 7.757400914 | 7.522364091 | Up | 4.00E-55 |
| 57214 | 0.02206288 | 3.984135836 | 7.496502028 | Up | 2.97E-107 |
| 286077 | 0.25117859 | 45.29295964 | 7.494429487 | Up | 0 |
| 63967 | 0.08917857 | 15.96246991 | 7.483771182 | Up | 5.34E-264 |
| 84740 | 0 | 0.160971878 | 7.330664861 | Up | 5.25E-05 |
| 8608 | 0.09065884 | 14.50992849 | 7.322377038 | Up | 9.23E-95 |
| 146439 | 0.04312677 | 6.801812938 | 7.301191708 | Up | 3.56E-47 |
| 6768 | 0.30767686 | 48.17238574 | 7.290646608 | Up | 0 |
| 91319 | 0.14653394 | 22.51826443 | 7.263717002 | Up | 2.48E-270 |
| 4288 | 0.06250207 | 9.233499455 | 7.206829717 | Up | 0 |
| 55723 | 0.09023985 | 12.54803224 | 7.119480792 | Up | 5.33E-82 |
| 55561 | 0.01581344 | 2.169375535 | 7.099984616 | Up | 6.18E-41 |
| 80326 | 0.0657706 | 8.869326823 | 7.075237954 | Up | 1.89E-79 |
| 83715 | 0.13271468 | 17.46341099 | 7.039863624 | Up | 4.41E-230 |
| 9119 | 0.14701664 | 19.31105008 | 7.037303384 | Up | 1.61E-153 |
| 222584 | 0.04933834 | 6.400152392 | 7.019253344 | Up | 2.47E-76 |
| 7153 | 0.24676963 | 31.89572576 | 7.014054438 | Up | 0 |
| 23120 | 0 | 0.125237228 | 6.968519666 | Up | 0.00019636 |
| 79883 | 0.16413033 | 20.35001555 | 6.954044255 | Up | 4.49E-252 |
| 9212 | 0.12637959 | 15.50935395 | 6.939231261 | Up | 4.36E-72 |
| 3543 | 0.86780654 | 104.4728952 | 6.911539527 | Up | 0 |
| 22974 | 0.2132785 | 25.49689053 | 6.901438937 | Up | 0 |
| 8911 | 0 | 0.116585931 | 6.865249886 | Up | 2.72E-05 |
| 64151 | 0.03349886 | 3.892157098 | 6.860314204 | Up | 3.99E-68 |
| 3236 | 0.08658824 | 9.939281789 | 6.842826777 | Up | 2.82E-67 |
| 5652 | 0.44284973 | 49.70665563 | 6.810477999 | Up | 0 |
| 57834 | 0.04601036 | 5.088209646 | 6.78905552 | Up | 9.87E-65 |
| 1063 | 0.07585721 | 8.155308135 | 6.748309178 | Up | 0 |
| 6692 | 0.56824842 | 60.92664568 | 6.74440774 | Up | 0 |
| 2886 | 0.10474196 | 11.14337002 | 6.733202286 | Up | 9.91E-93 |
| 1366 | 0.55787563 | 57.26919507 | 6.681671985 | Up | 0 |
| 9493 | 0.12952336 | 13.01428716 | 6.650740125 | Up | 1.60E-173 |
| 3880 | 2.22998552 | 223.5696505 | 6.647546201 | Up | 0 |
| 991 | 0.65318772 | 65.00709869 | 6.6369558 | Up | 0 |
| 84958 | 0.16459976 | 16.35949226 | 6.635021892 | Up | 8.88E-115 |
| 10279 | 0.05764029 | 5.728840647 | 6.635021892 | Up | 5.94E-58 |
| 699 | 0.04463005 | 4.435759473 | 6.635021892 | Up | 5.94E-58 |
| 56666 | 0.05118125 | 4.991351864 | 6.607671404 | Up | 7.99E-57 |
| 56265 | 0.52286252 | 50.19822485 | 6.585060892 | Up | 0 |
| 204219 | 0.04011432 | 3.799763094 | 6.565648189 | Up | 3.94E-55 |
| 10112 | 0.0450159 | 4.264056913 | 6.565648189 | Up | 3.94E-55 |
| 126695 | 0.0875099 | 8.085062833 | 6.529668892 | Up | 1.01E-53 |
| 4188 | 0.67621289 | 62.43040942 | 6.52862761 | Up | 0 |
| 9834 | 0 | 0.091005806 | 6.507886678 | Up | 0.0003796 |
| 4998 | 0.04947899 | 4.455938177 | 6.492769309 | Up | 2.60E-52 |
| 2261 | 0.52759726 | 47.4544947 | 6.490963844 | Up | 0 |
| 10158 | 0.3494523 | 31.06303503 | 6.473959459 | Up | 4.36E-102 |
| 9133 | 0.20648404 | 18.25814751 | 6.46636631 | Up | 1.60E-101 |
| 983 | 0.16220683 | 14.30513454 | 6.462554696 | Up | 3.05E-101 |
| 51203 | 0.25819037 | 22.49891969 | 6.445276704 | Up | 1.10E-198 |
| 441317 | 0.03992975 | 3.428273064 | 6.423874228 | Up | 2.18E-25 |
| 159963 | 0.01249042 | 1.037428101 | 6.376045702 | Up | 1.52E-24 |
| 84290 | 0.30962374 | 25.71670134 | 6.376045702 | Up | 4.79E-95 |
| 1277 | 74.0044096 | 6118.158137 | 6.36934235 | Up | 0 |
| 7020 | 0.20510133 | 16.76731781 | 6.353171236 | Up | 9.31E-232 |
| 57451 | 0.14575911 | 11.84950629 | 6.345097114 | Up | 0 |
| 11122 | 0 | 0.080387028 | 6.328890804 | Up | 0.00010157 |
| 3664 | 0.28793581 | 22.87408906 | 6.311823259 | Up | 4.15E-180 |
| 113130 | 0.15159664 | 11.94051399 | 6.299483365 | Up | 4.95E-112 |
| 2041 | 0.02323099 | 1.821117663 | 6.292629694 | Up | 3.89E-23 |
| 51237 | 0.37776343 | 29.26098919 | 6.275351703 | Up | 2.70E-88 |
| 1258 | 0.02767142 | 2.130474927 | 6.266634486 | Up | 1.99E-44 |
| 6513 | 5.83109242 | 442.8496201 | 6.246906883 | Up | 0 |
| 255738 | 0.12888216 | 9.702371882 | 6.234213008 | Up | 6.87E-128 |
| 150468 | 0.04772538 | 3.585389153 | 6.23122915 | Up | 2.64E-43 |
| 55057 | 0.06535781 | 4.849038731 | 6.213195227 | Up | 9.64E-43 |
| 121551 | 0.08135686 | 5.795613617 | 6.154553559 | Up | 1.37E-120 |
| 65989 | 0.10090774 | 7.109882451 | 6.138717011 | Up | 1.70E-40 |
| 1001 | 0.74887889 | 51.54665878 | 6.105002691 | Up | 0 |
| 10202 | 0.09320118 | 6.394448035 | 6.100327812 | Up | 1.18E-39 |
| 3084 | 0.06621669 | 4.527209096 | 6.095283191 | Up | 2.76E-54 |
| 3833 | 0.11553637 | 7.84409295 | 6.085187614 | Up | 8.77E-77 |
| 94032 | 0.11485675 | 7.771154144 | 6.080221361 | Up | 8.20E-39 |
| 5453 | 0.21339505 | 14.36352949 | 6.07273978 | Up | 6.63E-151 |
| 4973 | 0.06208473 | 4.142684222 | 6.060183608 | Up | 2.98E-38 |
| 2921 | 0.06773858 | 4.361905104 | 6.008836728 | Up | 6.35E-19 |
| 860 | 0.05479922 | 3.426416736 | 5.966401462 | Up | 2.44E-70 |
| 64073 | 0.67767972 | 41.42445425 | 5.933735273 | Up | 1.16E-68 |
| 80215 | 0.05245305 | 3.13286518 | 5.900312271 | Up | 1.60E-17 |
| 10631 | 5.61672949 | 334.3920705 | 5.895666505 | Up | 0 |
| 114569 | 0.22156763 | 13.15603753 | 5.891834118 | Up | 7.22E-132 |
| 55143 | 0.20348047 | 11.83679412 | 5.862244231 | Up | 4.62E-97 |
| 54209 | 0.29725057 | 17.12973588 | 5.848679503 | Up | 1.82E-64 |
| 25837 | 0.04809273 | 2.737790673 | 5.831049609 | Up | 1.10E-16 |
| 29842 | 0.0336504 | 1.907776836 | 5.825124775 | Up | 2.38E-63 |
| 55655 | 0.2670174 | 15.11752489 | 5.823144411 | Up | 3.39E-187 |
| 9928 | 0.053664 | 2.984834932 | 5.797552697 | Up | 2.81E-77 |
| 53637 | 0.14722448 | 8.106307108 | 5.782955321 | Up | 2.14E-61 |
| 10993 | 0.43823148 | 23.90219636 | 5.769306259 | Up | 3.66E-135 |
| 8714 | 0.18145809 | 9.709000914 | 5.741614525 | Up | 4.91E-176 |
| 56649 | 0.13263955 | 6.952527142 | 5.711954552 | Up | 9.78E-87 |
| 5754 | 1.04774006 | 54.89334259 | 5.711278447 | Up | 0 |
| 144406 | 0.16644132 | 8.659583814 | 5.701212171 | Up | 2.76E-114 |
| 90853 | 0.08035246 | 4.143071678 | 5.688214831 | Up | 3.26E-57 |
| 990 | 0.10232897 | 5.276207889 | 5.688214831 | Up | 3.26E-57 |
| 200634 | 0.54680925 | 28.06658335 | 5.681671985 | Up | 8.72E-85 |
| 9319 | 0.26576806 | 13.61031951 | 5.678389401 | Up | 2.46E-112 |
| 1591 | 0.04782767 | 2.432577845 | 5.668496596 | Up | 9.53E-29 |
| 4314 | 1.23904544 | 62.60080106 | 5.65888012 | Up | 0 |
| 221914 | 0.0308462 | 1.55448483 | 5.655199774 | Up | 9.90E-15 |
| 84419 | 0.34068741 | 17.00986502 | 5.641779258 | Up | 2.90E-55 |
| 151648 | 0.13362291 | 6.578007708 | 5.62141146 | Up | 1.99E-54 |
| 55001 | 0.03946568 | 1.937988247 | 5.617817266 | Up | 3.99E-16 |
| 84929 | 0.04797456 | 2.350505828 | 5.614557789 | Up | 1.24E-27 |
| 79098 | 0.05678123 | 2.755492152 | 5.60075199 | Up | 1.36E-53 |
| 2978 | 0.0821268 | 3.947145554 | 5.586812799 | Up | 4.46E-27 |
| 121355 | 0.57711272 | 27.64719762 | 5.582136311 | Up | 1.15E-78 |
| 55200 | 0.10000331 | 4.759656068 | 5.572737613 | Up | 1.76E-52 |
| 58985 | 0.05582744 | 2.657106068 | 5.572737613 | Up | 8.46E-27 |
| 60675 | 0.0968414 | 4.563976883 | 5.558523754 | Up | 1.60E-26 |
| 54894 | 0.03427807 | 1.599474796 | 5.544168461 | Up | 3.04E-26 |
| 7087 | 0.0520337 | 2.427983559 | 5.544168461 | Up | 3.04E-26 |
| 5522 | 0.33437341 | 15.52032961 | 5.536555277 | Up | 1.04E-237 |
| 5744 | 2.58534996 | 119.6251555 | 5.532017411 | Up | 0 |
| 2591 | 0.04781303 | 2.119487699 | 5.47016788 | Up | 7.44E-25 |
| 119548 | 0.06767989 | 3.000158714 | 5.47016788 | Up | 7.44E-25 |
| 10045 | 0.29860803 | 13.22141222 | 5.46847953 | Up | 1.27E-107 |
| 147700 | 0.39203753 | 17.31752164 | 5.46509689 | Up | 2.40E-107 |
| 3224 | 0.13654299 | 6.020911084 | 5.462554696 | Up | 2.56E-48 |
| 2171 | 56.0918588 | 2436.670149 | 5.440975764 | Up | 0 |
| 26470 | 0.73367077 | 31.51636007 | 5.424824307 | Up | 0 |
| 253982 | 0.21137372 | 9.074021805 | 5.423874228 | Up | 6.24E-47 |
| 128178 | 0.05403154 | 2.319509552 | 5.423874228 | Up | 5.05E-24 |
| 7472 | 0.24205348 | 10.36596813 | 5.420385241 | Up | 9.61E-104 |
| 11004 | 0.37966536 | 16.24796481 | 5.419386837 | Up | 1.77E-160 |
| 1594 | 0.06280868 | 2.637686361 | 5.392165368 | Up | 1.81E-23 |
| 79852 | 0.51809345 | 21.71733987 | 5.389491233 | Up | 1.24E-134 |
| 3553 | 1.6684131 | 69.82268048 | 5.387147293 | Up | 0 |
| 55083 | 0.06430843 | 2.690666924 | 5.386812133 | Up | 7.61E-68 |
| 27076 | 1.14452796 | 47.8514856 | 5.385739099 | Up | 4.50E-278 |
| 641455 | 0.01171656 | 0.48110935 | 5.35974389 | Up | 5.95E-12 |
| 7262 | 0.59425882 | 24.40165703 | 5.35974389 | Up | 2.52E-77 |
| 768 | 0.70362692 | 28.89256688 | 5.35974389 | Up | 2.77E-153 |
| 344 | 0.3249761 | 13.24318544 | 5.348772772 | Up | 1.50E-33 |
| 78990 | 0.04033183 | 1.637299711 | 5.343255767 | Up | 1.23E-22 |
| 8857 | 0.00953052 | 0.386898217 | 5.343255767 | Up | 1.23E-22 |
| 79755 | 0.09790359 | 3.951625038 | 5.334940499 | Up | 6.91E-44 |
| 220042 | 0.08768183 | 3.539050097 | 5.334940499 | Up | 6.91E-44 |
| 2906 | 0.21469394 | 8.644098201 | 5.331362089 | Up | 5.76E-150 |
| 84561 | 0.2670174 | 10.67363708 | 5.32097432 | Up | 1.01E-127 |
| 56924 | 0.1401482 | 5.549283706 | 5.30727647 | Up | 2.77E-74 |
| 80328 | 0.40617988 | 16.02887643 | 5.302410715 | Up | 5.24E-74 |
| 11065 | 1.07727708 | 42.32534617 | 5.2960606 | Up | 7.69E-208 |
| 4070 | 5.3902848 | 211.7342046 | 5.295749036 | Up | 0 |
| 1058 | 0.10931083 | 4.284532948 | 5.292629694 | Up | 8.28E-22 |
| 79623 | 0.05759778 | 2.257594979 | 5.292629694 | Up | 8.28E-22 |
| 51659 | 0.46371342 | 17.99018434 | 5.277832692 | Up | 1.26E-72 |
| 8091 | 0.03773072 | 1.461282492 | 5.275351703 | Up | 1.56E-21 |
| 374393 | 0.13167056 | 5.038062475 | 5.257864276 | Up | 1.73E-61 |
| 196051 | 0.66754349 | 25.49405086 | 5.255154989 | Up | 2.18E-131 |
| 81930 | 0.04510689 | 1.704860925 | 5.240162274 | Up | 5.58E-21 |
| 3512 | 0.99776195 | 37.60796615 | 5.236198824 | Up | 1.04E-178 |
| 11262 | 0.04806313 | 1.79416779 | 5.222240366 | Up | 1.05E-20 |
| 115908 | 3.58503684 | 131.1387753 | 5.192962574 | Up | 0 |
| 53345 | 0.10533053 | 3.833627231 | 5.18571449 | Up | 3.75E-20 |
| 9232 | 3.18114474 | 114.7577563 | 5.172901839 | Up | 3.98E-273 |
| 1824 | 0.56585288 | 20.31694054 | 5.166112354 | Up | 0 |
| 9076 | 0.40998734 | 14.66689001 | 5.160839822 | Up | 1.61E-168 |
| 51200 | 0.27735294 | 9.913398243 | 5.159584758 | Up | 3.65E-94 |
| 57662 | 0.13063754 | 4.65020639 | 5.153653281 | Up | 5.18E-66 |
| 100144603 | 0.12456553 | 4.417458573 | 5.148239785 | Up | 2.71E-10 |
| 348825 | 0.13835711 | 4.874268394 | 5.138717011 | Up | 8.10E-38 |
| 80320 | 0.16468653 | 5.782632578 | 5.133931948 | Up | 4.08E-74 |
| 10403 | 0.17987699 | 6.311817823 | 5.132973028 | Up | 9.15E-47 |
| 3204 | 0.58140885 | 20.1482149 | 5.114955193 | Up | 9.95E-136 |
| 6624 | 5.09059001 | 176.2752086 | 5.113852899 | Up | 0 |
| 1364 | 0.4284289 | 14.83350118 | 5.113659553 | Up | 7.28E-91 |
| 115761 | 0.02371056 | 0.818718415 | 5.109765637 | Up | 5.11E-10 |
| 57126 | 0.06607664 | 2.250773626 | 5.09013683 | Up | 8.93E-19 |
| 3589 | 0.19907202 | 6.781005333 | 5.09013683 | Up | 4.70E-54 |
| 54101 | 0.10085561 | 3.407215916 | 5.078230064 | Up | 7.68E-45 |
| 9633 | 0.02154554 | 0.723853901 | 5.070237273 | Up | 9.64E-10 |
| 84069 | 0.09770966 | 3.252303215 | 5.056816757 | Up | 6.20E-27 |
| 81624 | 0.04885483 | 1.626151608 | 5.056816757 | Up | 6.20E-27 |
| 642475 | 0.72094697 | 23.66058773 | 5.036448959 | Up | 9.65E-254 |
| 53831 | 0.10441522 | 3.410537869 | 5.029595288 | Up | 5.95E-18 |
| 7784 | 0.21151683 | 6.908821465 | 5.029595288 | Up | 8.51E-35 |
| 341208 | 0.05249712 | 1.714725139 | 5.029595288 | Up | 8.51E-35 |
| 675 | 0.01371906 | 0.448108612 | 5.029595288 | Up | 5.95E-18 |
| 10653 | 2.1639248 | 69.78844507 | 5.011265917 | Up | 0 |
| 6337 | 0.26939668 | 8.673658957 | 5.008836728 | Up | 3.37E-100 |
| 1493 | 0.15802243 | 5.087786126 | 5.008836728 | Up | 3.01E-34 |
| 6374 | 0.22242397 | 7.116816903 | 4.999847945 | Up | 1.34E-58 |
| 387914 | 0.21627577 | 6.912886884 | 4.998344354 | Up | 1.05E-66 |
| 79767 | 0.46637692 | 14.82715046 | 4.990601157 | Up | 4.13E-123 |
| 430 | 0.16896179 | 5.36115578 | 4.987775113 | Up | 1.06E-33 |
| 83461 | 0.2670174 | 8.472459006 | 4.987775113 | Up | 1.06E-33 |
| 1281 | 64.0185149 | 2026.593607 | 4.984423794 | Up | 0 |
| 91683 | 0.04551433 | 1.422931367 | 4.966401462 | Up | 3.96E-17 |
| 347735 | 2.77680709 | 84.89616553 | 4.934200533 | Up | 0 |
| 114787 | 0.14848401 | 4.520861536 | 4.928218268 | Up | 3.79E-63 |
| 7804 | 0.38124183 | 11.46608367 | 4.91052247 | Up | 8.42E-169 |
| 54457 | 0.03336292 | 0.996334099 | 4.900312271 | Up | 1.21E-08 |
| 9582 | 0.40678431 | 12.14801108 | 4.900312271 | Up | 8.80E-62 |
| 218 | 0.32441366 | 9.688133443 | 4.900312271 | Up | 8.80E-62 |
| 1365 | 0.12261003 | 3.604354934 | 4.877592195 | Up | 4.92E-16 |
| 128239 | 0.12905253 | 3.769657105 | 4.868403023 | Up | 5.55E-75 |
| 5639 | 0.11213581 | 3.244120689 | 4.854508582 | Up | 9.23E-16 |
| 3918 | 2.38905303 | 68.36415836 | 4.838729373 | Up | 0 |
| 1846 | 0.11495818 | 3.261403032 | 4.82631169 | Up | 6.60E-37 |
| 84000 | 0.04606463 | 1.289673839 | 4.807202867 | Up | 3.24E-15 |
| 283358 | 0.0454349 | 1.272043045 | 4.807202867 | Up | 3.24E-15 |
| 57167 | 0.04483501 | 1.255247987 | 4.807202867 | Up | 3.24E-15 |
| 79019 | 0.33592511 | 9.4049118 | 4.807202867 | Up | 2.50E-29 |
| 348738 | 0.0821268 | 2.29930809 | 4.807202867 | Up | 3.24E-15 |
| 5603 | 1.19966899 | 33.54858567 | 4.805543643 | Up | 3.90E-204 |
| 54742 | 0.79900346 | 22.29517327 | 4.802385851 | Up | 6.60E-141 |
| 54210 | 1.61298823 | 44.12897067 | 4.773918275 | Up | 6.42E-131 |
| 81620 | 0.46715567 | 12.77925352 | 4.773756384 | Up | 1.82E-110 |
| 2644 | 0.43816319 | 11.96059435 | 4.770676991 | Up | 1.64E-28 |
| 147744 | 0.13170757 | 3.564509152 | 4.758293267 | Up | 7.99E-08 |
| 130399 | 0.0175986 | 0.476285247 | 4.758293267 | Up | 1.14E-14 |
| 53833 | 0.45942699 | 12.36238774 | 4.749977998 | Up | 1.21E-81 |
| 85409 | 0.65157888 | 17.51259439 | 4.748309178 | Up | 1.45E-101 |
| 84451 | 0.0537804 | 1.442955352 | 4.745802322 | Up | 5.72E-28 |
| 4318 | 0.53494923 | 14.29055412 | 4.73951606 | Up | 1.77E-107 |
| 283248 | 0.12038935 | 3.202019104 | 4.733202286 | Up | 1.07E-27 |
| 2146 | 0.37815033 | 10.01701919 | 4.727349559 | Up | 9.51E-87 |
| 24137 | 0.1053779 | 2.786366381 | 4.724740707 | Up | 1.14E-40 |
| 136 | 0.49720481 | 13.10825174 | 4.720491234 | Up | 9.58E-80 |
| 341 | 5.08259793 | 133.9179876 | 4.719639835 | Up | 5.29E-197 |
| 100048912 | 0.02038168 | 0.532585037 | 4.707667194 | Up | 1.50E-07 |
| 6317 | 0.17580774 | 4.593958043 | 4.707667194 | Up | 3.73E-27 |
| 245972 | 0.13181871 | 3.444499343 | 4.707667194 | Up | 3.73E-27 |
| 8626 | 0.90687462 | 23.65261558 | 4.704952809 | Up | 0 |
| 3038 | 0.59238749 | 15.4103236 | 4.701212171 | Up | 6.39E-207 |
| 7137 | 0.64930893 | 16.88025292 | 4.700287663 | Up | 2.49E-46 |
| 146722 | 0.26641019 | 6.754266533 | 4.664077925 | Up | 9.04E-39 |
| 51512 | 0.2008424 | 5.060695067 | 4.655199774 | Up | 6.63E-51 |
| 1746 | 0.06779739 | 1.708314057 | 4.655199774 | Up | 1.39E-13 |
| 63950 | 0.05035628 | 1.268844484 | 4.655199774 | Up | 1.39E-13 |
| 92359 | 0.28246867 | 7.117460377 | 4.655199774 | Up | 4.54E-26 |
| 1830 | 2.5607406 | 63.35523701 | 4.628830858 | Up | 0 |
| 220441 | 0.08654026 | 2.140203613 | 4.628232726 | Up | 2.59E-13 |
| 9022 | 0.57640287 | 14.2548617 | 4.628232726 | Up | 1.09E-37 |
| 27036 | 0.13358481 | 3.282871864 | 4.619130519 | Up | 2.69E-19 |
| 25758 | 0.04026598 | 0.989544123 | 4.619130519 | Up | 2.04E-37 |
| 79589 | 0.11000365 | 2.694805274 | 4.614557789 | Up | 2.94E-25 |
| 348 | 7.41312702 | 181.417162 | 4.6130849 | Up | 0 |
| 143686 | 0.55029504 | 13.42578376 | 4.608657188 | Up | 8.17E-85 |
| 81706 | 1.48363126 | 36.13085519 | 4.606026932 | Up | 7.49E-251 |
| 26085 | 0.06208473 | 1.506430626 | 4.60075199 | Up | 5.23E-07 |
| 4680 | 0.05937103 | 1.440585122 | 4.60075199 | Up | 4.83E-13 |
| 7421 | 1.06317659 | 25.76650391 | 4.599043647 | Up | 0 |
| 64919 | 0.07995147 | 1.930624409 | 4.593799229 | Up | 1.80E-48 |
| 760 | 3.46914257 | 82.29438955 | 4.568143039 | Up | 0 |
| 9651 | 0.12917525 | 3.058978187 | 4.565648189 | Up | 2.16E-47 |
| 27074 | 0.18701607 | 4.385064758 | 4.551363963 | Up | 7.47E-47 |
| 57468 | 0.01292875 | 0.300742971 | 4.539876142 | Up | 9.76E-07 |
| 5831 | 1.51729166 | 35.15194268 | 4.534034084 | Up | 2.75E-225 |
| 55612 | 0.36390257 | 8.405263342 | 4.529668892 | Up | 2.52E-135 |
| 9837 | 0.18997285 | 4.343588028 | 4.515022116 | Up | 1.66E-45 |
| 23072 | 0.02284035 | 0.522227702 | 4.515022116 | Up | 3.13E-12 |
| 59341 | 0.19302462 | 4.413364543 | 4.515022116 | Up | 1.66E-45 |
| 5753 | 0.84048222 | 18.94102006 | 4.494152924 | Up | 1.12E-147 |
| 891 | 0.70968397 | 15.96496147 | 4.491588546 | Up | 3.74E-104 |
| 30837 | 0.03627617 | 0.812500509 | 4.485274772 | Up | 1.82E-06 |
| 3223 | 0.0753886 | 1.688526636 | 4.485274772 | Up | 5.82E-12 |
| 6424 | 4.78125498 | 106.0100906 | 4.470668439 | Up | 0 |
| 6696 | 4.75824999 | 105.0933783 | 4.46509689 | Up | 0 |
| 79056 | 2.1055123 | 46.08335025 | 4.452002398 | Up | 2.03E-278 |
| 5881 | 0.5894535 | 12.85855512 | 4.447206731 | Up | 4.35E-43 |
| 1999 | 0.60134423 | 13.06463033 | 4.441331425 | Up | 5.01E-131 |
| 55010 | 0.09796499 | 2.125616237 | 4.439471083 | Up | 5.04E-22 |
| 3772 | 0.10709988 | 2.323822482 | 4.439471083 | Up | 5.04E-22 |
| 116372 | 0.11644068 | 2.526496526 | 4.439471083 | Up | 5.04E-22 |
| 149466 | 0.26475454 | 5.73221404 | 4.436365172 | Up | 3.64E-27 |
| 1747 | 0.1800982 | 3.86570068 | 4.423874228 | Up | 4.96E-32 |
| 29785 | 0.50657917 | 10.8456108 | 4.420179744 | Up | 4.91E-88 |
| 597 | 2.15341176 | 45.91252542 | 4.414191674 | Up | 3.49E-133 |
| 51702 | 0.34298502 | 7.293379297 | 4.410369963 | Up | 3.59E-72 |
| 4753 | 0.7167309 | 15.16780006 | 4.40343648 | Up | 3.93E-172 |
| 899 | 0.20040319 | 4.233526603 | 4.400882585 | Up | 1.45E-56 |
| 55355 | 0.12803703 | 2.700443118 | 4.398563139 | Up | 2.32E-26 |
| 8140 | 2.75069648 | 57.98315478 | 4.397764967 | Up | 0 |
| 6241 | 0.55228701 | 11.63975605 | 4.397498812 | Up | 2.28E-121 |
| 5055 | 0.21675094 | 4.551289996 | 4.392165368 | Up | 3.16E-31 |
| 55287 | 0.36539223 | 7.672428048 | 4.392165368 | Up | 3.27E-41 |
| 146802 | 0.13265832 | 2.785531202 | 4.392165368 | Up | 3.22E-21 |
| 7681 | 0.05757655 | 1.208980092 | 4.392165368 | Up | 3.73E-11 |
| 54478 | 0.10533053 | 2.211708018 | 4.392165368 | Up | 3.73E-11 |
| 338440 | 0.19016282 | 3.980322238 | 4.387578098 | Up | 4.35E-36 |
| 11009 | 0.11863684 | 2.472658457 | 4.381438961 | Up | 4.59E-16 |
| 375616 | 0.11553637 | 2.40290329 | 4.378359568 | Up | 8.06E-36 |
| 176 | 0.07352562 | 1.526721091 | 4.376045702 | Up | 1.12E-40 |
| 163782 | 0.3707627 | 7.638807768 | 4.36477948 | Up | 7.80E-128 |
| 643036 | 0.10441522 | 2.14376666 | 4.35974389 | Up | 6.30E-06 |
| 220832 | 0.09134806 | 1.875482412 | 4.35974389 | Up | 6.30E-06 |
| 23566 | 0.18152839 | 3.726990034 | 4.35974389 | Up | 1.10E-20 |
| 1116 | 1.70065516 | 34.71805016 | 4.351523383 | Up | 1.19E-193 |
| 6573 | 0.16512175 | 3.364459727 | 4.348772772 | Up | 3.71E-30 |
| 10409 | 4.04203712 | 82.3387729 | 4.348417472 | Up | 0 |
| 283208 | 0.30979431 | 6.296192607 | 4.345097114 | Up | 1.84E-44 |
| 25791 | 0.24529707 | 4.967559805 | 4.339935409 | Up | 4.81E-49 |
| 7941 | 0.63705211 | 12.88125155 | 4.337717584 | Up | 9.75E-73 |
| 2118 | 0.32651584 | 6.551393933 | 4.326577026 | Up | 1.64E-48 |
| 2358 | 0.15535075 | 3.080798433 | 4.309703208 | Up | 6.99E-20 |
| 84627 | 0.2247897 | 4.438537107 | 4.303436449 | Up | 8.72E-177 |
| 56938 | 0.28327364 | 5.589343956 | 4.302410715 | Up | 1.10E-33 |
| 1748 | 0.0777527 | 1.52379233 | 4.292629694 | Up | 2.37E-10 |
| 26579 | 0.20490404 | 3.983825232 | 4.281134055 | Up | 1.48E-28 |
| 1294 | 1.23512655 | 24.00719388 | 4.280736016 | Up | 0 |
| 10855 | 0.1742389 | 3.374071119 | 4.275351703 | Up | 2.39E-19 |
| 2491 | 0.12189245 | 2.360401684 | 4.275351703 | Up | 2.39E-19 |
| 113157 | 0.79764345 | 15.42538938 | 4.27341909 | Up | 2.15E-163 |
| 5818 | 2.10933997 | 40.68696994 | 4.269703297 | Up | 0 |
| 9066 | 0.70657681 | 13.60620128 | 4.26727412 | Up | 6.70E-185 |
| 639 | 0.71071087 | 13.57566698 | 4.255616504 | Up | 1.22E-209 |
| 140766 | 0.37071667 | 7.057701517 | 4.250809519 | Up | 8.26E-112 |
| 3909 | 0.78065414 | 14.73719687 | 4.238634805 | Up | 0 |
| 2175 | 0.25748106 | 4.845854967 | 4.234213008 | Up | 8.28E-80 |
| 5347 | 0.90021425 | 16.90304169 | 4.230870671 | Up | 5.96E-110 |
| 55630 | 1.81947358 | 34.11714931 | 4.228904097 | Up | 3.13E-235 |
| 1290 | 5.38716265 | 100.7601265 | 4.225255404 | Up | 0 |
| 3755 | 0.03518135 | 0.656649247 | 4.222240366 | Up | 2.17E-05 |
| 55 | 0.04852599 | 0.9057231 | 4.222240366 | Up | 8.10E-10 |
| 2300 | 0.04896714 | 0.913956946 | 4.222240366 | Up | 8.10E-10 |
| 1718 | 3.37120364 | 62.2082471 | 4.205770171 | Up | 0 |
| 113730 | 0.10448507 | 1.9258051 | 4.20409302 | Up | 2.77E-18 |
| 51514 | 0.17702309 | 3.254521553 | 4.200435996 | Up | 1.01E-43 |
| 23397 | 0.10425262 | 1.91341309 | 4.19799282 | Up | 1.07E-26 |
| 9447 | 0.26314888 | 4.813362879 | 4.193094021 | Up | 2.32E-22 |
| 25788 | 0.12841596 | 2.324939969 | 4.178297019 | Up | 4.26E-22 |
| 56901 | 13.7402701 | 248.3593375 | 4.175946714 | Up | 0 |
| 90141 | 0.33713347 | 6.082745114 | 4.173330766 | Up | 1.15E-13 |
| 80178 | 0.28784738 | 5.103955513 | 4.148239785 | Up | 1.23E-25 |
| 23682 | 0.99847059 | 17.65257859 | 4.144015199 | Up | 5.53E-74 |
| 7022 | 0.59974064 | 10.58339813 | 4.141320371 | Up | 6.89E-90 |
| 2919 | 3.18641566 | 55.97116254 | 4.134677334 | Up | 3.50E-181 |
| 150696 | 0.69479016 | 12.12894041 | 4.125732385 | Up | 3.01E-136 |
| 8438 | 0.49651995 | 8.618677915 | 4.117542988 | Up | 3.20E-80 |
| 2214 | 1.86119686 | 32.18064979 | 4.111890894 | Up | 8.59E-217 |
| 2125 | 0.42685489 | 7.225733626 | 4.081326452 | Up | 8.61E-139 |
| 4651 | 1.26412763 | 21.36264415 | 4.078876191 | Up | 0 |
| 3235 | 0.58007227 | 9.744187133 | 4.070237273 | Up | 2.24E-54 |
| 2672 | 0.05604778 | 0.941503549 | 4.070237273 | Up | 9.34E-09 |
| 4237 | 11.7756854 | 194.8315891 | 4.048344667 | Up | 0 |
| 909 | 0.11184142 | 1.843946311 | 4.043270225 | Up | 2.42E-12 |
| 80131 | 0.30356364 | 4.998154474 | 4.041324581 | Up | 4.61E-53 |
| 638 | 0.328853 | 5.370699633 | 4.029595288 | Up | 6.62E-16 |
| 115362 | 0.50127556 | 8.132660093 | 4.020051516 | Up | 1.59E-95 |
| 65997 | 0.31927476 | 5.177026692 | 4.019253344 | Up | 2.46E-30 |
| 114904 | 1.94192403 | 31.40442918 | 4.015409382 | Up | 3.46E-264 |
| 3149 | 1.88887024 | 30.49984333 | 4.013206328 | Up | 0 |
| 29984 | 7.58947412 | 122.3386274 | 4.010736261 | Up | 0 |
| 9700 | 0.09434104 | 1.496720072 | 3.987775113 | Up | 1.50E-29 |
| 200810 | 0.38808739 | 6.157004371 | 3.987775113 | Up | 2.22E-15 |
| 223117 | 0.04992176 | 0.792008392 | 3.987775113 | Up | 2.22E-15 |
| 1089 | 0.06481543 | 1.028296373 | 3.987775113 | Up | 0.00013621 |
| 55806 | 1.27479741 | 20.15856027 | 3.983052702 | Up | 0 |
| 9048 | 0.56275668 | 8.853105344 | 3.975600399 | Up | 3.52E-50 |
| 83541 | 1.59569065 | 25.07413882 | 3.973947259 | Up | 5.60E-130 |
| 54933 | 0.46768017 | 7.340387748 | 3.972262113 | Up | 1.30E-39 |
| 116028 | 0.43632731 | 6.820531358 | 3.966401462 | Up | 5.02E-29 |
| 3291 | 0.91299519 | 14.1748312 | 3.956580491 | Up | 9.33E-77 |
| 7477 | 1.1785301 | 18.21269589 | 3.949883974 | Up | 5.51E-202 |
| 10643 | 0.2253501 | 3.470025765 | 3.944706391 | Up | 4.33E-42 |
| 771 | 10.5512176 | 160.8855082 | 3.930552984 | Up | 0 |
| 2305 | 0.96928198 | 14.77460806 | 3.930059615 | Up | 8.35E-152 |
| 92815 | 0.47239469 | 7.200635597 | 3.930059615 | Up | 2.72E-11 |
| 9134 | 0.08595296 | 1.310167005 | 3.930059615 | Up | 2.72E-11 |
| 7058 | 5.72761277 | 86.48948908 | 3.916518951 | Up | 0 |
| 29128 | 0.23061788 | 3.47940081 | 3.915262613 | Up | 4.78E-41 |
| 9620 | 0.418321 | 6.297467503 | 3.912089439 | Up | 5.72E-202 |
| 219699 | 0.75008488 | 11.22925394 | 3.904064406 | Up | 3.61E-158 |
| 4909 | 0.15636154 | 2.334752879 | 3.900312271 | Up | 1.05E-07 |
| 117144 | 0.02983292 | 0.445458007 | 3.900312271 | Up | 0.0002502 |
| 147798 | 0.97707031 | 14.55996485 | 3.897400676 | Up | 3.97E-102 |
| 2625 | 0.33072757 | 4.890860485 | 3.88637308 | Up | 2.87E-43 |
| 3198 | 0.18398725 | 2.718636165 | 3.885205379 | Up | 1.20E-20 |
| 5655 | 0.25145714 | 3.684296984 | 3.873004925 | Up | 3.24E-33 |
| 3557 | 3.21025207 | 47.024559 | 3.872655929 | Up | 4.02E-252 |
| 202 | 0.42402094 | 6.196257768 | 3.869189754 | Up | 2.91E-131 |
| 1825 | 2.1601891 | 31.5033546 | 3.866275947 | Up | 0 |
| 54972 | 1.64192941 | 23.79215578 | 3.857021988 | Up | 4.98E-233 |
| 10733 | 0.12286721 | 1.777291605 | 3.854508582 | Up | 3.97E-20 |
| 256933 | 0.25357983 | 3.668068279 | 3.854508582 | Up | 1.93E-07 |
| 63926 | 0.12257796 | 1.773107554 | 3.854508582 | Up | 3.97E-20 |
| 2700 | 0.0300106 | 0.4341076 | 3.854508582 | Up | 1.93E-07 |
| 26191 | 0.04290172 | 0.620579528 | 3.854508582 | Up | 1.93E-07 |
| 101 | 0.88508293 | 12.74704011 | 3.848205841 | Up | 1.91E-116 |
| 1745 | 0.09836598 | 1.407578109 | 3.838911727 | Up | 1.65E-10 |
| 116238 | 0.76646308 | 10.94393932 | 3.835772019 | Up | 3.53E-32 |
| 9768 | 1.15629673 | 16.43169735 | 3.828897938 | Up | 1.07E-68 |
| 9949 | 0.99484559 | 14.0340139 | 3.818311257 | Up | 4.65E-210 |
| 27237 | 0.48410695 | 6.826981431 | 3.817850111 | Up | 6.23E-56 |
| 6491 | 0.18673661 | 2.62855948 | 3.815195658 | Up | 1.14E-37 |
| 79935 | 0.08601607 | 1.204098014 | 3.807202867 | Up | 3.51E-07 |
| 85415 | 0.13275227 | 1.858335972 | 3.807202867 | Up | 2.38E-19 |
| 10331 | 0.05742837 | 0.803912498 | 3.807202867 | Up | 3.51E-07 |
| 8000 | 0.15314233 | 2.14376666 | 3.807202867 | Up | 3.51E-07 |
| 6195 | 1.39163724 | 19.48086803 | 3.807202867 | Up | 4.06E-172 |
| 1870 | 0.25572881 | 3.565787534 | 3.801534117 | Up | 3.70E-52 |
| 58527 | 3.97445123 | 55.37767889 | 3.800477004 | Up | 1.77E-129 |
| 148113 | 0.20460311 | 2.829424823 | 3.789608807 | Up | 6.86E-34 |
| 8836 | 0.89230108 | 12.24597891 | 3.778633715 | Up | 3.99E-51 |
| 399 | 0.46884995 | 6.380896263 | 3.766560883 | Up | 4.03E-36 |
| 23616 | 1.4250084 | 19.38211394 | 3.765683598 | Up | 4.67E-137 |
| 50489 | 0.16724323 | 2.263119836 | 3.758293267 | Up | 8.95E-13 |
| 654346 | 0.09086979 | 1.22964161 | 3.758293267 | Up | 6.39E-07 |
| 114827 | 0.03068864 | 0.415275821 | 3.758293267 | Up | 6.39E-07 |
| 83648 | 0.03816398 | 0.516431451 | 3.758293267 | Up | 6.39E-07 |
| 55350 | 0.08156928 | 1.103787951 | 3.758293267 | Up | 6.39E-07 |
| 5265 | 1.41522244 | 19.04747603 | 3.750499109 | Up | 1.66E-183 |
| 144501 | 0.20057162 | 2.676680406 | 3.738255513 | Up | 1.36E-29 |
| 84842 | 0.39007952 | 5.177398727 | 3.73038727 | Up | 1.38E-26 |
| 80352 | 0.17995988 | 2.384816921 | 3.728131296 | Up | 2.72E-15 |
| 79092 | 0.09972241 | 1.321514994 | 3.728131296 | Up | 2.72E-15 |
| 993 | 0.1264478 | 1.671744643 | 3.724740707 | Up | 4.66E-18 |
| 1278 | 136.589317 | 1801.847962 | 3.721560731 | Up | 0 |
| 2210 | 0.13934449 | 1.820576147 | 3.707667194 | Up | 1.16E-06 |
| 401115 | 1.00994726 | 13.19525341 | 3.707667194 | Up | 8.44E-18 |
| 92840 | 0.51196161 | 6.68892671 | 3.707667194 | Up | 4.51E-26 |
| 54443 | 0.52395866 | 6.84567165 | 3.707667194 | Up | 4.19E-89 |
| 57864 | 0.06245709 | 0.816019936 | 3.707667194 | Up | 1.16E-06 |
| 140862 | 0.90396514 | 11.81056632 | 3.707667194 | Up | 1.24E-83 |
| 11082 | 0.22443272 | 2.897370458 | 3.690389202 | Up | 1.52E-17 |
| 2326 | 0.80821117 | 10.38809226 | 3.684054636 | Up | 1.87E-60 |
| 414236 | 0.12466495 | 1.599698186 | 3.681671985 | Up | 5.31E-12 |
| 404550 | 0.8707089 | 11.13229109 | 3.67641626 | Up | 4.71E-28 |
| 392255 | 0.06330931 | 0.807460342 | 3.672901775 | Up | 3.26E-09 |
| 56913 | 1.41052146 | 17.97755199 | 3.671896062 | Up | 3.10E-94 |
| 54809 | 0.61696018 | 7.858178024 | 3.670945578 | Up | 2.20E-144 |
| 1890 | 12.2532204 | 155.7709636 | 3.668193456 | Up | 0 |
| 10437 | 33.3752339 | 423.7678282 | 3.666424211 | Up | 0 |
| 3556 | 0.84283369 | 10.67256496 | 3.662515157 | Up | 2.03E-135 |
| 6790 | 0.67461992 | 8.527932392 | 3.660049173 | Up | 3.56E-59 |
| 6659 | 6.51655461 | 82.34648098 | 3.659525699 | Up | 0 |
| 149469 | 0.13148584 | 1.656546965 | 3.655199774 | Up | 2.10E-06 |
| 7161 | 0.10981032 | 1.383464179 | 3.655199774 | Up | 9.60E-12 |
| 3713 | 0.07316402 | 0.921769459 | 3.655199774 | Up | 2.10E-06 |
| 9001 | 0.13897258 | 1.741606062 | 3.647546201 | Up | 1.55E-19 |
| 401647 | 1.02553502 | 12.74407796 | 3.635378333 | Up | 7.29E-99 |
| 653820 | 0.13159661 | 1.6272399 | 3.628232726 | Up | 1.73E-11 |
| 10397 | 64.9208115 | 788.8383293 | 3.602976714 | Up | 0 |
| 10460 | 1.57328955 | 19.1134534 | 3.602732354 | Up | 1.14E-140 |
| 100130311 | 0.10136611 | 1.229779187 | 3.60075199 | Up | 3.12E-11 |
| 3887 | 0.08178281 | 0.992193575 | 3.60075199 | Up | 3.81E-06 |
| 9966 | 0.07837691 | 0.950872919 | 3.60075199 | Up | 3.81E-06 |
| 79919 | 0.12372687 | 1.501061171 | 3.60075199 | Up | 3.12E-11 |
| 79822 | 0.08552939 | 1.037647232 | 3.60075199 | Up | 2.91E-16 |
| 55215 | 0.57561394 | 6.952683534 | 3.594396495 | Up | 7.33E-88 |
| 25903 | 4.00399345 | 48.20761357 | 3.589749496 | Up | 0 |
| 9170 | 1.21899246 | 14.66699665 | 3.588812376 | Up | 3.03E-70 |
| 57121 | 0.43770277 | 5.233644001 | 3.579792371 | Up | 1.92E-40 |
| 353091 | 0.28221351 | 3.357986801 | 3.572737613 | Up | 5.62E-11 |
| 10615 | 0.47173307 | 5.60824671 | 3.57150717 | Up | 4.50E-57 |
| 8784 | 0.71594039 | 8.503607753 | 3.57016367 | Up | 5.39E-28 |
| 3832 | 0.38466602 | 4.566273667 | 3.569339037 | Up | 8.75E-62 |
| 84296 | 0.30516771 | 3.588393998 | 3.5556641 | Up | 3.41E-37 |
| 8534 | 0.63217695 | 7.428254013 | 3.554622819 | Up | 7.89E-54 |
| 1945 | 2.2368283 | 26.26753707 | 3.55375451 | Up | 4.83E-87 |
| 196996 | 0.2847861 | 3.344302046 | 3.55375451 | Up | 5.58E-30 |
| 6402 | 0.83429223 | 9.792275156 | 3.553019379 | Up | 2.98E-63 |
| 64063 | 0.2254043 | 2.629439627 | 3.544168461 | Up | 1.01E-10 |
| 84222 | 0.3191117 | 3.722577449 | 3.544168461 | Up | 2.97E-20 |
| 4072 | 3.82320362 | 44.59934136 | 3.544168461 | Up | 4.14E-199 |
| 92211 | 0.08801945 | 1.026785309 | 3.544168461 | Up | 1.69E-15 |
| 50619 | 1.36067227 | 15.80934542 | 3.538386109 | Up | 3.46E-95 |
| 10103 | 1.89450723 | 21.9576726 | 3.534830597 | Up | 6.94E-74 |
| 57586 | 0.07572483 | 0.87629632 | 3.532580487 | Up | 5.48E-13 |
| 160364 | 0.25113372 | 2.906147987 | 3.532580487 | Up | 5.48E-13 |
| 7070 | 31.4235789 | 360.5768997 | 3.520387567 | Up | 0 |
| 6556 | 0.97324097 | 11.16406002 | 3.519920915 | Up | 5.60E-112 |
| 11339 | 0.25275919 | 2.88957545 | 3.515022116 | Up | 1.81E-10 |
| 6713 | 2.01133756 | 22.89026569 | 3.508507078 | Up | 9.96E-177 |
| 23601 | 0.24469623 | 2.781828797 | 3.506969843 | Up | 1.78E-26 |
| 7051 | 0.16874884 | 1.916034876 | 3.50517433 | Up | 5.42E-15 |
| 11169 | 0.24747335 | 2.802202808 | 3.501216316 | Up | 2.01E-35 |
| 6715 | 1.55239243 | 17.54593472 | 3.498571595 | Up | 4.94E-103 |
| 8744 | 1.12580307 | 12.69521878 | 3.495258861 | Up | 1.65E-55 |
| 864 | 1.33170312 | 14.98068094 | 3.491758806 | Up | 2.76E-167 |
| 1164 | 2.54820842 | 28.65583037 | 3.491273513 | Up | 2.46E-46 |
| 9618 | 2.23380938 | 25.1164943 | 3.491057125 | Up | 2.69E-187 |
| 1470 | 0.56269876 | 6.301561941 | 3.485274772 | Up | 1.76E-12 |
| 23251 | 0.04633793 | 0.518930168 | 3.485274772 | Up | 3.25E-10 |
| 53904 | 0.02700643 | 0.30244011 | 3.485274772 | Up | 1.24E-05 |
| 255057 | 0.06092246 | 0.682259592 | 3.485274772 | Up | 1.24E-05 |
| 170679 | 1.45138375 | 16.25378485 | 3.485274772 | Up | 3.74E-37 |
| 80714 | 0.48882009 | 5.474208051 | 3.485274772 | Up | 1.74E-21 |
| 26298 | 0.12955917 | 1.45090981 | 3.485274772 | Up | 9.71E-15 |
| 2019 | 0.18457735 | 2.067048475 | 3.485274772 | Up | 5.42E-17 |
| 283212 | 0.26086369 | 2.921365389 | 3.485274772 | Up | 1.76E-12 |
| 2256 | 1.26575097 | 14.14807036 | 3.482539804 | Up | 2.69E-99 |
| 140707 | 0.67541546 | 7.506554014 | 3.474303654 | Up | 1.02E-25 |
| 55784 | 0.27677778 | 3.070884543 | 3.471854256 | Up | 6.15E-61 |
| 375790 | 4.95144158 | 54.86273508 | 3.469906024 | Up | 0 |
| 9401 | 0.73933557 | 8.164690063 | 3.46509689 | Up | 3.13E-80 |
| 148641 | 0.10806308 | 1.184966836 | 3.454901123 | Up | 5.83E-10 |
| 387882 | 4.92074915 | 53.82503369 | 3.45132744 | Up | 6.26E-187 |
| 9055 | 1.60570692 | 17.56056853 | 3.451059057 | Up | 1.61E-139 |
| 80329 | 0.17400322 | 1.902234707 | 3.450509354 | Up | 1.73E-16 |
| 10635 | 0.2066206 | 2.249631681 | 3.444632788 | Up | 3.10E-14 |
| 158405 | 0.08733051 | 0.950832026 | 3.444632788 | Up | 1.11E-07 |
| 11015 | 4.80983681 | 52.24001009 | 3.441095324 | Up | 1.18E-225 |
| 541471 | 3.25838864 | 35.29219905 | 3.437118734 | Up | 8.57E-72 |
| 92558 | 0.30419703 | 3.288361323 | 3.434290843 | Up | 5.96E-27 |
| 3012 | 0.27695953 | 2.972385689 | 3.423874228 | Up | 2.22E-05 |
| 255520 | 0.48435714 | 5.198218693 | 3.423874228 | Up | 3.11E-18 |
| 55150 | 0.20995319 | 2.253260119 | 3.423874228 | Up | 2.22E-05 |
| 23286 | 0.34779244 | 3.732578733 | 3.423874228 | Up | 1.31E-64 |
| 8914 | 0.6081572 | 6.498488307 | 3.417587965 | Up | 4.00E-85 |
| 100133941 | 5.2307238 | 55.50192216 | 3.407455239 | Up | 3.00E-303 |
| 669 | 8.91327683 | 94.51533867 | 3.406520661 | Up | 0 |
| 55971 | 1.93977498 | 20.52960824 | 3.403744887 | Up | 4.91E-189 |
| 202915 | 0.23644824 | 2.496962124 | 3.400577608 | Up | 1.37E-40 |
| 53905 | 0.70164383 | 7.408211087 | 3.400314438 | Up | 2.38E-106 |
| 51053 | 1.92845897 | 20.27668021 | 3.394301112 | Up | 7.34E-63 |
| 200931 | 0.21501057 | 2.257373015 | 3.392165368 | Up | 1.86E-09 |
| 124222 | 1.9728737 | 20.71299065 | 3.392165368 | Up | 3.69E-140 |
| 51599 | 3.42855962 | 35.80410288 | 3.384450403 | Up | 8.52E-204 |
| 127579 | 0.19372283 | 2.018809612 | 3.381438961 | Up | 1.76E-13 |
| 4050 | 0.87460905 | 9.059994822 | 3.372800043 | Up | 1.80E-21 |
| 55706 | 1.02236629 | 10.58192476 | 3.37161799 | Up | 1.52E-67 |
| 1468 | 1.61591562 | 16.66565339 | 3.366454112 | Up | 3.72E-79 |
| 25886 | 0.24127013 | 2.476778957 | 3.35974389 | Up | 3.11E-15 |
| 100128191 | 0.148343 | 1.522827599 | 3.35974389 | Up | 3.13E-13 |
| 5028 | 0.05025907 | 0.515938694 | 3.35974389 | Up | 3.98E-05 |
| 89872 | 0.08721674 | 0.8953308 | 3.35974389 | Up | 3.98E-05 |
| 222171 | 0.09309009 | 0.955624232 | 3.35974389 | Up | 3.98E-05 |
| 147699 | 0.59237669 | 6.081093059 | 3.35974389 | Up | 3.45E-27 |
| 81615 | 0.08256088 | 0.847535656 | 3.35974389 | Up | 3.98E-05 |
| 5050 | 2.84526733 | 29.07556749 | 3.353171236 | Up | 3.88E-80 |
| 286499 | 0.24747335 | 2.517363512 | 3.346568501 | Up | 5.69E-21 |
| 4015 | 1.10003645 | 11.18052543 | 3.345364751 | Up | 9.02E-109 |
| 6004 | 7.93229412 | 80.53052408 | 3.343725646 | Up | 0 |
| 1869 | 0.95458719 | 9.6644177 | 3.339733929 | Up | 1.48E-65 |
| 23767 | 0.11783141 | 1.191280945 | 3.337717584 | Up | 5.56E-13 |
| 10663 | 0.23994651 | 2.425870107 | 3.337717584 | Up | 5.56E-13 |
| 3655 | 4.5033334 | 45.43899771 | 3.334865817 | Up | 0 |
| 3620 | 0.49778578 | 5.017151802 | 3.333271679 | Up | 1.01E-20 |
| 1525 | 0.60030306 | 6.044520074 | 3.331864858 | Up | 4.25E-38 |
| 5427 | 0.4161772 | 4.185993159 | 3.330300112 | Up | 9.93E-19 |
| 3217 | 0.74328964 | 7.47022643 | 3.32915557 | Up | 1.93E-26 |
| 204962 | 0.20057162 | 2.002830794 | 3.319851163 | Up | 1.79E-20 |
| 4828 | 2.50231593 | 24.83855468 | 3.311245372 | Up | 8.04E-64 |
| 3728 | 16.6981018 | 164.6838126 | 3.301942738 | Up | 0 |
| 6280 | 54.4146282 | 536.4915394 | 3.301488974 | Up | 0 |
| 6474 | 0.3878465 | 3.82311708 | 3.301191708 | Up | 2.09E-31 |
| 483 | 17.9555869 | 176.9689212 | 3.300991303 | Up | 0 |
| 3627 | 1.92763835 | 18.98191348 | 3.299719119 | Up | 1.15E-55 |
| 54541 | 15.156895 | 149.1875607 | 3.299081108 | Up | 0 |
| 58498 | 0.26165021 | 2.563901835 | 3.292629694 | Up | 7.10E-05 |
| 8972 | 0.02409458 | 0.23610202 | 3.292629694 | Up | 7.10E-05 |
| 80071 | 0.20062314 | 1.965899558 | 3.292629694 | Up | 5.61E-20 |
| 4232 | 1.58809655 | 15.56170593 | 3.292629694 | Up | 3.42E-94 |
| 2563 | 0.08114555 | 0.795142543 | 3.292629694 | Up | 7.10E-05 |
| 126006 | 0.22125379 | 2.168058634 | 3.292629694 | Up | 7.10E-05 |
| 84659 | 0.10427582 | 1.021795324 | 3.292629694 | Up | 7.10E-05 |
| 1462 | 5.31177924 | 52.00763338 | 3.291456294 | Up | 0 |
| 9718 | 0.9193816 | 8.998526723 | 3.290953116 | Up | 2.09E-77 |
| 84518 | 3.84596078 | 37.41035229 | 3.282021595 | Up | 3.10E-49 |
| 10630 | 4.8605807 | 47.25282544 | 3.281200006 | Up | 0 |
| 5803 | 0.23931422 | 2.322698526 | 3.278823895 | Up | 2.95E-47 |
| 2215 | 0.68122624 | 6.611739129 | 3.278823895 | Up | 4.24E-38 |
| 10381 | 7.66875209 | 74.29385616 | 3.276179173 | Up | 1.80E-307 |
| 10232 | 0.07612338 | 0.737307031 | 3.275853981 | Up | 7.10E-05 |
| 1040 | 1.01543691 | 9.82977009 | 3.275057065 | Up | 5.34E-109 |
| 924 | 0.66962468 | 6.476414322 | 3.273770667 | Up | 1.79E-21 |
| 1241 | 1.43619567 | 13.87924227 | 3.272604584 | Up | 1.38E-139 |
| 1894 | 0.53918944 | 5.18099782 | 3.264365842 | Up | 3.16E-50 |
| 84984 | 0.79548932 | 7.626251397 | 3.261059615 | Up | 4.50E-41 |
| 633 | 90.5071169 | 866.2961075 | 3.258757088 | Up | 0 |
| 374739 | 0.19272693 | 1.843559978 | 3.257864276 | Up | 1.86E-08 |
| 357 | 0.08392488 | 0.802796702 | 3.257864276 | Up | 9.65E-16 |
| 7447 | 4.12911439 | 39.47952128 | 3.257200205 | Up | 4.04E-191 |
| 23550 | 0.85247913 | 8.143675613 | 3.25594367 | Up | 7.14E-100 |
| 6343 | 0.7597528 | 7.232084415 | 3.250809519 | Up | 3.16E-10 |
| 366 | 0.41365158 | 3.932722553 | 3.249040426 | Up | 6.34E-30 |
| 93099 | 2.82833998 | 26.80983717 | 3.244735004 | Up | 2.74E-125 |
| 125965 | 3.14664667 | 29.76007043 | 3.241490845 | Up | 1.29E-119 |
| 84817 | 2.86062492 | 26.97736628 | 3.237347259 | Up | 6.51E-135 |
| 387509 | 0.29674236 | 2.796996471 | 3.236595659 | Up | 5.46E-19 |
| 441168 | 0.84511367 | 7.952620415 | 3.234213008 | Up | 1.78E-22 |
| 27111 | 1.43258823 | 13.41893071 | 3.227573811 | Up | 1.66E-48 |
| 65243 | 0.23715361 | 2.213200398 | 3.222240366 | Up | 9.67E-12 |
| 4173 | 2.40961849 | 22.48740233 | 3.222240366 | Up | 6.48E-190 |
| 85474 | 0.10757932 | 1.003967857 | 3.222240366 | Up | 0.0001264 |
| 8673 | 18.1046431 | 168.0747732 | 3.214671561 | Up | 2.07E-296 |
| 8045 | 1.67233551 | 15.480278 | 3.210495159 | Up | 7.85E-65 |
| 440823 | 0.15401812 | 1.422977436 | 3.207740797 | Up | 1.22E-35 |
| 25923 | 2.83883521 | 26.20222602 | 3.206318371 | Up | 4.16E-141 |
| 10451 | 0.45855996 | 4.218307054 | 3.201481806 | Up | 5.18E-49 |
| 6813 | 3.14063318 | 28.80814055 | 3.197349191 | Up | 9.05E-130 |
| 283643 | 0.91401508 | 8.359310137 | 3.193094021 | Up | 3.78E-35 |
| 114784 | 0.08341747 | 0.761799291 | 3.190989432 | Up | 5.65E-25 |
| 163732 | 9.74013687 | 88.9223119 | 3.190531507 | Up | 4.43E-271 |
| 79879 | 0.24502773 | 2.229517327 | 3.18571449 | Up | 5.80E-08 |
| 283152 | 0.71373122 | 6.470481095 | 3.180420191 | Up | 5.26E-13 |
| 2769 | 3.88314932 | 34.9397767 | 3.169571298 | Up | 1.87E-175 |
| 65078 | 0.9767911 | 8.773915243 | 3.167098812 | Up | 7.32E-41 |
| 5329 | 9.0816963 | 81.26925239 | 3.161675924 | Up | 2.38E-294 |
| 55966 | 0.10688004 | 0.9475698 | 3.148239785 | Up | 1.02E-07 |
| 257358 | 0.07087349 | 0.6283454 | 3.148239785 | Up | 0.00022414 |
| 861 | 1.79311689 | 15.87224323 | 3.145964599 | Up | 5.17E-269 |
| 1062 | 0.66204005 | 5.848310952 | 3.143029613 | Up | 2.95E-118 |
| 4495 | 6.1140915 | 53.92980674 | 3.140872815 | Up | 4.91E-51 |
| 55620 | 3.65867687 | 32.24716017 | 3.139778206 | Up | 2.75E-116 |
| 1944 | 2.89268845 | 25.48219158 | 3.139006505 | Up | 1.17E-106 |
| 5228 | 3.70197239 | 32.44150935 | 3.131474902 | Up | 4.03E-131 |
| 9398 | 0.30827072 | 2.691947196 | 3.126380351 | Up | 1.64E-23 |
| 135398 | 0.13831627 | 1.204761429 | 3.122704693 | Up | 6.20E-06 |
| 25878 | 3.2858436 | 28.59552245 | 3.121453548 | Up | 0 |
| 29887 | 1.36331128 | 11.81815818 | 3.115818307 | Up | 1.33E-71 |
| 54958 | 3.69645837 | 31.93721385 | 3.111022888 | Up | 1.40E-49 |
| 79598 | 0.14165058 | 1.222787785 | 3.109765637 | Up | 8.84E-14 |
| 50943 | 0.26230928 | 2.264364785 | 3.109765637 | Up | 8.84E-14 |
| 11226 | 0.91580468 | 7.901582425 | 3.109029756 | Up | 1.75E-83 |
| 54 | 5.50692682 | 47.50460738 | 3.108748102 | Up | 4.31E-179 |
| 9935 | 9.29792717 | 79.98163721 | 3.104687783 | Up | 0 |
| 1475 | 3.58875254 | 30.84742837 | 3.10359587 | Up | 5.46E-60 |
| 80758 | 0.52915033 | 4.543158614 | 3.101946133 | Up | 8.67E-17 |
| 3159 | 24.6354157 | 210.1566529 | 3.092659405 | Up | 0 |
| 6530 | 0.39669674 | 3.375455722 | 3.088973835 | Up | 2.88E-27 |
| 10123 | 5.67062205 | 48.17377446 | 3.086669057 | Up | 0 |
| 79801 | 0.5316389 | 4.510400152 | 3.084736843 | Up | 9.99E-35 |
| 55034 | 0.37232624 | 3.153939069 | 3.082515603 | Up | 4.81E-21 |
| 4001 | 1.02980512 | 8.700032915 | 3.078649513 | Up | 1.54E-58 |
| 79865 | 0.04156604 | 0.349117934 | 3.070237273 | Up | 0.00039591 |
| 127255 | 0.05457903 | 0.458415512 | 3.070237273 | Up | 0.00039591 |
| 2847 | 0.06446767 | 0.541471397 | 3.070237273 | Up | 0.00039591 |
| 728882 | 0.04275689 | 0.359120035 | 3.070237273 | Up | 1.09E-05 |
| 30009 | 0.06073296 | 0.510103109 | 3.070237273 | Up | 0.00039591 |
| 8492 | 0.09328467 | 0.783508627 | 3.070237273 | Up | 3.16E-07 |
| 124093 | 0.09702185 | 0.814897637 | 3.070237273 | Up | 0.00039591 |
| 10110 | 0.07368169 | 0.618860942 | 3.070237273 | Up | 0.00039591 |
| 1236 | 0.14383534 | 1.208089499 | 3.070237273 | Up | 3.16E-07 |
| 9053 | 0.92000543 | 7.690695733 | 3.063399843 | Up | 5.95E-71 |
| 284114 | 0.71254262 | 5.947784439 | 3.061304148 | Up | 4.91E-28 |
| 3937 | 3.99864614 | 33.19386346 | 3.053333037 | Up | 1.47E-182 |
| 201266 | 1.29141591 | 10.71575096 | 3.052707348 | Up | 9.59E-69 |
| 4323 | 24.5036385 | 203.2954889 | 3.052510311 | Up | 0 |
| 29089 | 2.66943797 | 22.09946888 | 3.049403769 | Up | 1.10E-46 |
| 11211 | 0.16801417 | 1.388770781 | 3.04715366 | Up | 1.52E-11 |
| 51537 | 2.67245811 | 22.07217752 | 3.045989727 | Up | 1.37E-59 |
| 4085 | 0.65176012 | 5.372833828 | 3.043270225 | Up | 8.03E-19 |
| 51155 | 5.66762145 | 46.6999647 | 3.042606154 | Up | 3.72E-179 |
| 29028 | 1.0948961 | 9.012758756 | 3.041174807 | Up | 4.32E-114 |
| 5731 | 0.27481558 | 2.256914091 | 3.037815795 | Up | 1.64E-08 |
| 115004 | 0.39007952 | 3.195425777 | 3.034168018 | Up | 2.56E-14 |
| 81557 | 0.90049102 | 7.367241152 | 3.032340665 | Up | 1.78E-44 |
| 7039 | 0.31145766 | 2.54757727 | 3.032017951 | Up | 8.05E-26 |
| 29923 | 5.53134477 | 45.21950865 | 3.031243141 | Up | 2.49E-144 |
| 25790 | 0.17404476 | 1.42121578 | 3.029595288 | Up | 5.52E-07 |
| 79953 | 0.64097323 | 5.23406304 | 3.029595288 | Up | 4.66E-30 |
| 146227 | 0.11294662 | 0.92230019 | 3.029595288 | Up | 5.52E-07 |
| 7850 | 0.75098643 | 6.107379194 | 3.023694687 | Up | 2.46E-21 |
| 57718 | 0.12115189 | 0.979880935 | 3.015789489 | Up | 8.66E-10 |
| 93100 | 10.8283824 | 87.42267793 | 3.013189825 | Up | 0 |
| 8612 | 6.97648391 | 56.32019078 | 3.013080202 | Up | 6.87E-174 |
| 1 | 0.35380561 | 2.847834986 | 3.008836728 | Up | 1.43E-12 |
| 9056 | 1.27628781 | 10.25487742 | 3.00628464 | Up | 1.12E-58 |
| 5888 | 0.72349111 | 5.787318907 | 2.999847945 | Up | 1.36E-30 |
| 4430 | 3.64836428 | 29.15522658 | 2.998432844 | Up | 0 |
| 2827 | 0.333139 | 2.659896264 | 2.997173811 | Up | 7.76E-14 |
| 100128553 | 0.11896815 | 0.943714493 | 2.987775113 | Up | 9.64E-07 |
| 9595 | 1.06069608 | 8.413968739 | 2.987775113 | Up | 1.46E-42 |
| 10205 | 3.22441878 | 25.54983063 | 2.986202693 | Up | 8.63E-149 |
| 116832 | 3.44406861 | 27.26822401 | 2.98503495 | Up | 8.22E-44 |
| 163351 | 0.20861591 | 1.647357162 | 2.981232267 | Up | 4.04E-19 |
| 3775 | 0.93968871 | 7.420349199 | 2.981232267 | Up | 7.56E-37 |
| 80004 | 1.53447015 | 12.09060938 | 2.978074474 | Up | 2.08E-108 |
| 653361 | 0.44693899 | 3.493204902 | 2.966401462 | Up | 4.32E-12 |
| 3624 | 34.2574111 | 267.6583847 | 2.965904814 | Up | 0 |
| 55076 | 3.44570242 | 26.8902905 | 2.964215323 | Up | 1.41E-93 |
| 6279 | 61.8312157 | 481.9902041 | 2.96259655 | Up | 0 |
| 80346 | 4.05727731 | 31.62498751 | 2.962481093 | Up | 1.15E-118 |
| 148979 | 0.24961765 | 1.941265974 | 2.959205961 | Up | 2.34E-13 |
| 915 | 1.89722886 | 14.75466932 | 2.959205961 | Up | 2.11E-25 |
| 822 | 18.6861255 | 145.3212143 | 2.959205961 | Up | 0 |
| 24147 | 1.04718554 | 8.089625252 | 2.94955579 | Up | 7.22E-49 |
| 5111 | 9.3953667 | 72.56531487 | 2.94925875 | Up | 2.51E-215 |
| 79586 | 10.1838881 | 78.51733538 | 2.946722736 | Up | 0 |
| 84187 | 0.23884584 | 1.838919108 | 2.944706391 | Up | 1.68E-06 |
| 54869 | 0.48342028 | 3.721943819 | 2.944706391 | Up | 2.05E-22 |
| 118738 | 0.08931114 | 0.687623268 | 2.944706391 | Up | 1.68E-06 |
| 254263 | 0.234895 | 1.808500897 | 2.944706391 | Up | 1.68E-06 |
| 494143 | 0.24502773 | 1.886514661 | 2.944706391 | Up | 1.68E-06 |
| 9368 | 4.95208622 | 38.09975316 | 2.943673312 | Up | 3.25E-167 |
| 7293 | 1.0238167 | 7.848434122 | 2.9384474 | Up | 1.15E-19 |
| 688 | 3.77690128 | 28.894135 | 2.9355017 | Up | 5.25E-211 |
| 794 | 1.76533247 | 13.47929996 | 2.932733749 | Up | 3.58E-44 |
| 56169 | 1.58277168 | 12.05848999 | 2.929524193 | Up | 2.45E-72 |
| 6678 | 174.412531 | 1324.914703 | 2.925323878 | Up | 0 |
| 84262 | 10.1848476 | 77.14162076 | 2.921085065 | Up | 4.74E-232 |
| 29127 | 1.31501652 | 9.949242985 | 2.919505837 | Up | 7.09E-73 |
| 221079 | 0.21761657 | 1.645007908 | 2.918234179 | Up | 3.80E-14 |
| 55370 | 0.32569887 | 2.462023929 | 2.918234179 | Up | 3.80E-14 |
| 4283 | 2.27095934 | 17.01198879 | 2.905178027 | Up | 2.28E-94 |
| 5650 | 0.16212265 | 1.210388234 | 2.900312271 | Up | 2.91E-06 |
| 116444 | 0.0960081 | 0.716784919 | 2.900312271 | Up | 2.91E-06 |
| 83903 | 0.08380106 | 0.625648639 | 2.900312271 | Up | 5.82E-05 |
| 162514 | 0.06851104 | 0.511495203 | 2.900312271 | Up | 5.82E-05 |
| 283849 | 0.12511428 | 0.934088156 | 2.900312271 | Up | 2.91E-06 |
| 160851 | 0.09192866 | 0.686328309 | 2.900312271 | Up | 1.50E-07 |
| 2902 | 0.07139176 | 0.533002314 | 2.900312271 | Up | 2.91E-06 |
| 400954 | 0.05969625 | 0.445684992 | 2.900312271 | Up | 7.87E-09 |
| 6320 | 8.1599719 | 60.86956509 | 2.899084968 | Up | 1.48E-184 |
| 23480 | 12.9053633 | 96.19347826 | 2.897968331 | Up | 2.12E-97 |
| 4494 | 4.42758437 | 32.89053339 | 2.893080702 | Up | 1.63E-32 |
| 10644 | 2.04245396 | 15.10972993 | 2.887102414 | Up | 1.18E-119 |
| 23657 | 0.17809462 | 1.314523587 | 2.883824149 | Up | 1.60E-28 |
| 6282 | 258.238187 | 1900.611551 | 2.879689441 | Up | 0 |
| 151246 | 0.44708659 | 3.289752009 | 2.879352653 | Up | 2.69E-33 |
| 5238 | 2.03677763 | 14.98701467 | 2.879352653 | Up | 4.62E-65 |
| 64403 | 0.92325041 | 6.790303967 | 2.878682255 | Up | 8.07E-53 |
| 24141 | 1.40725384 | 10.34222565 | 2.877592195 | Up | 1.45E-40 |
| 84624 | 2.22945871 | 16.35558811 | 2.875018251 | Up | 1.83E-228 |
| 2209 | 0.4307128 | 3.148657282 | 2.869938622 | Up | 5.81E-16 |
| 9636 | 34.7122614 | 253.6854925 | 2.869523757 | Up | 0 |
| 54913 | 2.41036706 | 17.49563533 | 2.859670287 | Up | 1.19E-87 |
| 1289 | 19.511763 | 141.3707153 | 2.857067256 | Up | 0 |
| 152189 | 0.2670174 | 1.931222274 | 2.854508582 | Up | 5.05E-06 |
| 128272 | 1.10875249 | 8.013115262 | 2.853425884 | Up | 1.16E-52 |
| 25902 | 1.36755505 | 9.845358615 | 2.847844852 | Up | 5.75E-76 |
| 1949 | 0.24262997 | 1.743516967 | 2.845170717 | Up | 3.35E-13 |
| 10699 | 0.15803842 | 1.135649761 | 2.845170717 | Up | 3.35E-13 |
| 23080 | 0.92939756 | 6.659980573 | 2.841150199 | Up | 2.16E-34 |
| 80727 | 3.82252402 | 27.38970892 | 2.841036462 | Up | 9.88E-278 |
| 6096 | 0.06501325 | 0.465156917 | 2.838911727 | Up | 0.00010097 |
| 139231 | 0.54690042 | 3.912963888 | 2.838911727 | Up | 4.26E-26 |
| 84725 | 0.11821784 | 0.845825191 | 2.838911727 | Up | 0.00010097 |
| 2191 | 9.46783174 | 67.64432066 | 2.836862841 | Up | 0 |
| 145474 | 0.46666403 | 3.325691245 | 2.833198076 | Up | 3.13E-14 |
| 79868 | 3.00168199 | 21.36658466 | 2.831513274 | Up | 8.98E-61 |
| 26996 | 0.31070149 | 2.210925934 | 2.831049609 | Up | 1.14E-10 |
| 55117 | 0.12941605 | 0.920913847 | 2.831049609 | Up | 1.14E-10 |
| 116984 | 0.49875294 | 3.545370624 | 2.829539197 | Up | 5.12E-56 |
| 140576 | 39.6363322 | 281.6564141 | 2.829040953 | Up | 0 |
| 29108 | 11.5426695 | 82.00890325 | 2.828803631 | Up | 6.10E-160 |
| 22925 | 0.46922098 | 3.333640249 | 2.82875901 | Up | 1.93E-37 |
| 1829 | 2.59743262 | 18.45087973 | 2.828531377 | Up | 9.92E-219 |
| 7474 | 1.99336856 | 14.14562852 | 2.827075897 | Up | 1.79E-173 |
| 6288 | 1.52714558 | 10.7906914 | 2.820877804 | Up | 1.53E-17 |
| 55285 | 0.94042852 | 6.626187859 | 2.816788916 | Up | 2.24E-24 |
| 921 | 0.27213445 | 1.916284294 | 2.815920084 | Up | 5.37E-14 |
| 375061 | 2.60689547 | 18.34365891 | 2.814876795 | Up | 1.35E-58 |
| 3560 | 0.32840564 | 2.307611305 | 2.81284943 | Up | 7.59E-21 |
| 10089 | 0.19987866 | 1.399003195 | 2.807202867 | Up | 8.71E-06 |
| 64220 | 0.54598104 | 3.821464512 | 2.807202867 | Up | 3.83E-24 |
| 11245 | 0.11094118 | 0.77650639 | 2.807202867 | Up | 8.71E-06 |
| 4521 | 9.04241161 | 63.24384392 | 2.80614556 | Up | 2.54E-104 |
| 1663 | 0.71599011 | 4.992841832 | 2.801849633 | Up | 4.66E-42 |
| 79729 | 0.46508091 | 3.240755634 | 2.800776598 | Up | 2.47E-18 |
| 6498 | 1.91362467 | 13.33166344 | 2.800477004 | Up | 4.25E-98 |
| 2049 | 2.31346529 | 16.11482607 | 2.800261258 | Up | 9.29E-142 |
| 5054 | 8.36110831 | 57.97079844 | 2.793560259 | Up | 0 |
| 57333 | 15.6710695 | 108.3492509 | 2.789513633 | Up | 0 |
| 1953 | 1.75334484 | 12.12024979 | 2.789237764 | Up | 1.40E-186 |
| 29909 | 0.43511191 | 3.004856221 | 2.787837542 | Up | 1.69E-12 |
| 51700 | 1.91200698 | 13.19338888 | 2.78665549 | Up | 4.43E-38 |
| 56833 | 0.78102588 | 5.367212166 | 2.780730656 | Up | 7.55E-38 |
| 30001 | 2.92589756 | 20.0607337 | 2.777423205 | Up | 2.83E-137 |
| 196792 | 0.9105224 | 6.231363194 | 2.774781389 | Up | 3.10E-11 |
| 100130776 | 1.83770796 | 12.57676441 | 2.774781389 | Up | 6.72E-41 |
| 10333 | 0.08510998 | 0.582469111 | 2.774781389 | Up | 0.00017447 |
| 2303 | 0.13955197 | 0.95505507 | 2.774781389 | Up | 0.00017447 |
| 5932 | 3.58159224 | 24.46685936 | 2.77215592 | Up | 9.45E-165 |
| 3985 | 3.55196282 | 24.24551067 | 2.771029255 | Up | 1.14E-191 |
| 10799 | 2.08902522 | 14.21285851 | 2.766294923 | Up | 4.04E-35 |
| 6820 | 0.43632731 | 2.966713 | 2.765382691 | Up | 6.28E-09 |
| 116447 | 4.09342706 | 27.81054108 | 2.764250722 | Up | 1.66E-109 |
| 201799 | 0.45570392 | 3.095084832 | 2.763810272 | Up | 6.13E-21 |
| 5590 | 2.35046482 | 15.95014008 | 2.762551101 | Up | 5.02E-77 |
| 871 | 57.5138388 | 389.805644 | 2.76077394 | Up | 0 |
| 8424 | 0.16564706 | 1.120760301 | 2.758293267 | Up | 1.50E-05 |
| 941 | 0.11331533 | 0.766686227 | 2.758293267 | Up | 1.50E-05 |
| 1678 | 2.32899535 | 15.7199644 | 2.754818287 | Up | 1.50E-47 |
| 3595 | 0.27065253 | 1.822201661 | 2.751168832 | Up | 2.22E-16 |
| 3559 | 0.67738585 | 4.551553152 | 2.748309178 | Up | 9.43E-23 |
| 51303 | 6.02007779 | 40.14018264 | 2.737193146 | Up | 2.87E-81 |
| 3321 | 0.96914834 | 6.461743655 | 2.737134102 | Up | 6.39E-96 |
| 9509 | 1.11147635 | 7.340670075 | 2.723434509 | Up | 2.59E-55 |
| 91523 | 2.3336677 | 15.38552921 | 2.720903021 | Up | 1.84E-65 |
| 78995 | 0.4675052 | 3.081313696 | 2.720491234 | Up | 5.72E-18 |
| 2212 | 12.0627968 | 79.46240194 | 2.719707966 | Up | 0 |
| 9262 | 1.25117226 | 8.214674659 | 2.71492301 | Up | 1.47E-88 |
| 91862 | 1.15370878 | 7.569396337 | 2.713899147 | Up | 2.59E-35 |
| 55357 | 1.74294075 | 11.42997423 | 2.713226719 | Up | 3.54E-77 |
| 27286 | 2.85306258 | 18.70460883 | 2.712810497 | Up | 2.92E-83 |
| 201232 | 0.17882676 | 1.168211712 | 2.707667194 | Up | 2.57E-05 |
| 2015 | 0.10003534 | 0.653495312 | 2.707667194 | Up | 2.57E-05 |
| 56992 | 0.14532216 | 0.949338058 | 2.707667194 | Up | 1.54E-10 |
| 3816 | 0.6255356 | 4.086401895 | 2.707667194 | Up | 1.83E-08 |
| 85477 | 0.14436708 | 0.943098827 | 2.707667194 | Up | 2.02E-07 |
| 191585 | 0.03121294 | 0.203903073 | 2.707667194 | Up | 2.57E-05 |
| 55065 | 0.16170308 | 1.056348789 | 2.707667194 | Up | 2.26E-06 |
| 10622 | 0.90346982 | 5.879859242 | 2.702233307 | Up | 6.08E-40 |
| 51195 | 1.92794154 | 12.51633017 | 2.69867841 | Up | 4.68E-94 |
| 92312 | 0.43361986 | 2.808880418 | 2.69549248 | Up | 1.21E-35 |
| 285761 | 0.67125581 | 4.348226411 | 2.69549248 | Up | 1.56E-18 |
| 79581 | 6.24659503 | 40.43068702 | 2.694308811 | Up | 1.65E-156 |
| 1311 | 0.92372555 | 5.974918987 | 2.693382968 | Up | 1.49E-30 |
| 10360 | 6.2410744 | 40.35469433 | 2.692870192 | Up | 1.60E-71 |
| 10299 | 6.35186957 | 41.03609206 | 2.691640154 | Up | 0 |
| 5597 | 4.56707843 | 29.48998711 | 2.690881722 | Up | 2.29E-247 |
| 3120 | 0.8353218 | 5.391897964 | 2.690389202 | Up | 2.10E-13 |
| 55038 | 3.05660353 | 19.72999434 | 2.690389202 | Up | 3.37E-97 |
| 50848 | 6.41961232 | 41.42396323 | 2.689907508 | Up | 0 |
| 84885 | 6.44634742 | 41.54176507 | 2.688008668 | Up | 5.88E-96 |
| 84649 | 0.76446253 | 4.904784243 | 2.681671985 | Up | 3.14E-25 |
| 3222 | 0.38098824 | 2.444416863 | 2.681671985 | Up | 2.84E-09 |
| 2535 | 0.86649367 | 5.550225938 | 2.679285392 | Up | 3.46E-23 |
| 10866 | 3.04962839 | 19.47276745 | 2.674752571 | Up | 1.36E-94 |
| 10625 | 10.1978123 | 65.09041531 | 2.674185426 | Up | 0 |
| 2237 | 2.15311715 | 13.7090494 | 2.670629814 | Up | 4.87E-62 |
| 9143 | 0.6158903 | 3.915623087 | 2.668496596 | Up | 4.73E-17 |
| 254251 | 0.11260465 | 0.714588887 | 2.665847018 | Up | 3.85E-06 |
| 388722 | 0.66413765 | 4.214616087 | 2.665847018 | Up | 3.85E-06 |
| 146760 | 0.49369525 | 3.13298895 | 2.665847018 | Up | 6.45E-21 |
| 22983 | 0.20628477 | 1.303159254 | 2.659304172 | Up | 5.66E-14 |
| 338773 | 1.99521606 | 12.60226843 | 2.659066554 | Up | 5.26E-68 |
| 2867 | 0.95200619 | 6.005891385 | 2.657335518 | Up | 1.45E-25 |
| 7739 | 2.08297719 | 13.13405094 | 2.656593009 | Up | 6.62E-112 |
| 942 | 1.63610714 | 10.31308548 | 2.656136893 | Up | 2.77E-56 |
| 1758 | 0.1163973 | 0.733225706 | 2.655199774 | Up | 4.38E-05 |
| 54549 | 0.31325541 | 1.971601462 | 2.653956607 | Up | 9.51E-43 |
| 3577 | 0.21991879 | 1.378012519 | 2.647546201 | Up | 5.28E-08 |
| 6457 | 0.32331054 | 2.025865833 | 2.647546201 | Up | 5.28E-08 |
| 129790 | 0.71716412 | 4.493754862 | 2.647546201 | Up | 1.75E-21 |
| 23149 | 0.50857499 | 3.186734068 | 2.647546201 | Up | 1.75E-21 |
| 100133205 | 0.24006943 | 1.493607919 | 2.637277866 | Up | 0.00051401 |
| 5328 | 17.4703158 | 108.4552508 | 2.63412231 | Up | 0 |
| 80896 | 0.95615282 | 5.917459003 | 2.629664682 | Up | 3.26E-19 |
| 1513 | 37.1239682 | 229.1134874 | 2.625639555 | Up | 0 |
| 11123 | 0.4596974 | 2.831437543 | 2.622778296 | Up | 1.18E-10 |
| 3280 | 18.8134246 | 115.8784814 | 2.622778296 | Up | 0 |
| 4063 | 0.11094118 | 0.682305872 | 2.620623704 | Up | 2.22E-05 |
| 79173 | 0.38728556 | 2.379403822 | 2.619130519 | Up | 1.72E-12 |
| 84002 | 1.85642824 | 11.38489323 | 2.616519305 | Up | 1.59E-91 |
| 386757 | 0.18310062 | 1.120186486 | 2.613030319 | Up | 1.27E-09 |
| 2189 | 1.57333226 | 9.613135332 | 2.611183669 | Up | 5.76E-50 |
| 387103 | 5.54827129 | 33.85511283 | 2.609263489 | Up | 4.22E-37 |
| 259307 | 0.64101476 | 3.904155611 | 2.606581068 | Up | 9.25E-19 |
| 2717 | 6.94000431 | 42.25246165 | 2.606026932 | Up | 7.94E-116 |
| 83882 | 0.3873656 | 2.349770276 | 2.60075199 | Up | 1.38E-08 |
| 51176 | 0.91645801 | 5.559259306 | 2.60075199 | Up | 4.72E-34 |
| 144110 | 0.52330042 | 3.174354653 | 2.60075199 | Up | 3.45E-23 |
| 3694 | 0.2606678 | 1.581217962 | 2.60075199 | Up | 1.38E-08 |
| 55055 | 1.40618026 | 8.518806668 | 2.598869804 | Up | 1.06E-54 |
| 79646 | 0.72080388 | 4.361566346 | 2.597167652 | Up | 2.04E-29 |
| 890 | 0.89323904 | 5.392362314 | 2.593799229 | Up | 3.29E-30 |
| 5130 | 5.22986377 | 31.44823082 | 2.588133585 | Up | 8.27E-96 |
| 5912 | 4.45942605 | 26.7470563 | 2.584450177 | Up | 0 |
| 952 | 0.26191344 | 1.564331406 | 2.578384177 | Up | 1.11E-05 |
| 6375 | 1.15044729 | 6.859351589 | 2.575877321 | Up | 2.74E-17 |
| 25976 | 3.68523683 | 21.96522545 | 2.575392067 | Up | 4.84E-160 |
| 92659 | 0.32989478 | 1.962667048 | 2.572737613 | Up | 2.32E-08 |
| 84798 | 7.16838392 | 42.63099268 | 2.572182837 | Up | 4.41E-135 |
| 780 | 14.1600134 | 84.12049317 | 2.570634677 | Up | 0 |
| 55722 | 0.35589415 | 2.113572764 | 2.57016367 | Up | 5.22E-11 |
| 735301 | 3.68724665 | 21.89770237 | 2.57016367 | Up | 5.22E-11 |
| 84957 | 1.49382627 | 8.863921299 | 2.568932701 | Up | 6.69E-60 |
| 717 | 3.11088913 | 18.42112924 | 2.565962618 | Up | 1.54E-99 |
| 646300 | 0.1619855 | 0.954761268 | 2.559275354 | Up | 7.33E-18 |
| 971 | 0.65590029 | 3.860995768 | 2.557424558 | Up | 1.29E-12 |
| 10072 | 5.60989934 | 32.9583181 | 2.554595833 | Up | 1.35E-166 |
| 84058 | 4.88577406 | 28.5950502 | 2.549106384 | Up | 3.72E-61 |
| 254552 | 3.57205904 | 20.90238741 | 2.548839901 | Up | 4.70E-33 |
| 79075 | 0.65429858 | 3.824373857 | 2.547202521 | Up | 1.23E-17 |
| 79651 | 1.88538434 | 11.00197229 | 2.544831617 | Up | 3.03E-75 |
| 349667 | 0.24735578 | 1.44275666 | 2.544168461 | Up | 0.00012635 |
| 939 | 0.94669804 | 5.521823216 | 2.544168461 | Up | 5.11E-15 |
| 125206 | 0.1460544 | 0.851894185 | 2.544168461 | Up | 0.00012635 |
| 8549 | 0.1085135 | 0.632928677 | 2.544168461 | Up | 0.00012635 |
| 9450 | 1.86593214 | 10.86272663 | 2.54141785 | Up | 3.12E-19 |
| 10673 | 1.36225445 | 7.930512214 | 2.54141785 | Up | 3.12E-19 |
| 267004 | 0.32362951 | 1.879250332 | 2.537742192 | Up | 6.05E-09 |
| 50515 | 1.70199345 | 9.883127655 | 2.537742192 | Up | 8.13E-32 |
| 1789 | 0.66646581 | 3.866295038 | 2.536348957 | Up | 1.31E-32 |
| 55248 | 1.19422918 | 6.909877859 | 2.532580487 | Up | 1.30E-26 |
| 162979 | 0.73867517 | 4.274016507 | 2.532580487 | Up | 5.39E-14 |
| 79682 | 0.95585481 | 5.524875152 | 2.531078462 | Up | 2.09E-27 |
| 5909 | 0.46942025 | 2.711920045 | 2.530362662 | Up | 5.21E-19 |
| 54892 | 1.19179941 | 6.877281957 | 2.528697052 | Up | 1.77E-51 |
| 3687 | 3.71277092 | 21.40077666 | 2.527094948 | Up | 1.14E-184 |
| 84948 | 1.86287092 | 10.72072709 | 2.524803136 | Up | 2.73E-46 |
| 1041 | 0.18384289 | 1.058006457 | 2.524803136 | Up | 2.81E-06 |
| 4145 | 0.51142948 | 2.931886677 | 2.519222104 | Up | 5.70E-13 |
| 146956 | 0.27118954 | 1.55013683 | 2.515022116 | Up | 6.56E-08 |
| 201633 | 0.29210879 | 1.669712602 | 2.515022116 | Up | 6.56E-08 |
| 27099 | 0.7296466 | 4.1707068 | 2.515022116 | Up | 3.55E-21 |
| 10537 | 2.52708071 | 14.44495554 | 2.515022116 | Up | 9.35E-28 |
| 1535 | 48.8141176 | 278.9451729 | 2.514611228 | Up | 0 |
| 5366 | 1.0488984 | 5.98615835 | 2.512755509 | Up | 9.10E-23 |
| 57823 | 1.05822099 | 6.035142875 | 2.511746984 | Up | 6.27E-31 |
| 10721 | 0.08895511 | 0.506397728 | 2.509121514 | Up | 1.57E-09 |
| 54361 | 0.60001988 | 3.415753178 | 2.509121514 | Up | 6.03E-26 |
| 3984 | 6.28196088 | 35.74402779 | 2.508415349 | Up | 7.81E-219 |
| 2950 | 377.061034 | 2145.328611 | 2.508328677 | Up | 0 |
| 3274 | 0.52993679 | 3.014433766 | 2.507994849 | Up | 1.45E-18 |
| 93145 | 4.74239251 | 26.86792777 | 2.502198075 | Up | 1.14E-92 |
| 55916 | 1.0154497 | 5.740070612 | 2.498949709 | Up | 1.08E-29 |
| 39 | 2.76959533 | 15.64908197 | 2.498330925 | Up | 9.37E-46 |
| 79839 | 0.52678352 | 2.975551169 | 2.497874809 | Up | 9.52E-17 |
| 6932 | 1.38276866 | 7.787186587 | 2.493542388 | Up | 6.59E-48 |
| 649 | 9.82189876 | 55.22694854 | 2.49129856 | Up | 0 |
| 4313 | 107.917133 | 606.5874708 | 2.490791777 | Up | 0 |
| 2048 | 0.40101966 | 2.245473397 | 2.485274772 | Up | 2.62E-21 |
| 92565 | 0.48211474 | 2.699558017 | 2.485274772 | Up | 1.10E-07 |
| 9956 | 0.32009257 | 1.792329503 | 2.485274772 | Up | 1.10E-07 |
| 284837 | 0.06795962 | 0.380533738 | 2.485274772 | Up | 0.00021307 |
| 4049 | 0.21412636 | 1.198981216 | 2.485274772 | Up | 0.00021307 |
| 9641 | 0.4308448 | 2.41247661 | 2.485274772 | Up | 9.96E-16 |
| 3691 | 7.12542619 | 39.82429581 | 2.482600638 | Up | 0 |
| 55227 | 1.4981943 | 8.366083099 | 2.481327588 | Up | 1.28E-49 |
| 1033 | 3.68988606 | 20.57919537 | 2.479538403 | Up | 1.39E-34 |
| 9601 | 12.6731503 | 70.61648715 | 2.478229857 | Up | 0 |
| 1111 | 0.90949157 | 5.066091955 | 2.477741099 | Up | 1.19E-26 |
| 153478 | 0.18313243 | 1.019103665 | 2.476341647 | Up | 1.12E-22 |
| 58489 | 1.52233858 | 8.462428054 | 2.474782398 | Up | 1.57E-37 |
| 84879 | 0.78102588 | 4.340153048 | 2.474303654 | Up | 1.06E-18 |
| 1606 | 7.21293744 | 40.04636551 | 2.473012498 | Up | 8.27E-201 |
| 441282 | 1.23190202 | 6.821280667 | 2.469155107 | Up | 4.16E-13 |
| 146330 | 0.61786401 | 3.416961803 | 2.467352864 | Up | 1.85E-22 |
| 79815 | 0.45025882 | 2.488860806 | 2.466659094 | Up | 1.68E-11 |
| 64581 | 0.74868279 | 4.13628598 | 2.465909447 | Up | 7.22E-21 |
| 1687 | 3.91295532 | 21.58889022 | 2.463958541 | Up | 5.75E-98 |
| 6446 | 10.8611412 | 59.88210209 | 2.462949172 | Up | 0 |
| 533 | 41.1146697 | 226.5806136 | 2.462299284 | Up | 0 |
| 25817 | 0.76977532 | 4.227397334 | 2.457260396 | Up | 1.91E-21 |
| 155368 | 1.42921851 | 7.845862912 | 2.45670562 | Up | 1.73E-14 |
| 80154 | 1.73900294 | 9.538086821 | 2.455439543 | Up | 3.84E-45 |
| 3925 | 12.2548093 | 67.18604963 | 2.454813669 | Up | 6.91E-269 |
| 26472 | 64.4128742 | 352.9938781 | 2.45422219 | Up | 0 |
| 285966 | 0.18383259 | 1.00484557 | 2.450509354 | Up | 1.20E-06 |
| 2152 | 6.64420146 | 36.27351669 | 2.448748896 | Up | 5.85E-153 |
| 914 | 1.98954993 | 10.85465653 | 2.447800067 | Up | 4.15E-31 |
| 282969 | 4.88141176 | 26.57377423 | 2.444632788 | Up | 8.41E-38 |
| 8900 | 0.47696237 | 2.596521451 | 2.444632788 | Up | 1.77E-10 |
| 401303 | 0.16611681 | 0.904318442 | 2.444632788 | Up | 7.88E-06 |
| 339983 | 0.20009203 | 1.089275118 | 2.444632788 | Up | 7.88E-06 |
| 401541 | 0.52632861 | 2.86170551 | 2.442839506 | Up | 7.75E-19 |
| 90381 | 0.19909192 | 1.082009084 | 2.44220605 | Up | 2.87E-14 |
| 3429 | 89.6023558 | 486.4048729 | 2.440549113 | Up | 0 |
| 2922 | 3.50401222 | 19.0180197 | 2.440287317 | Up | 4.25E-30 |
| 3070 | 0.65526534 | 3.55149985 | 2.438277293 | Up | 5.25E-21 |
| 26586 | 0.64806751 | 3.511737677 | 2.437969057 | Up | 9.22E-25 |
| 54566 | 0.35004746 | 1.89472385 | 2.436365172 | Up | 3.28E-20 |
| 57524 | 0.06857119 | 0.371159601 | 2.436365172 | Up | 5.26E-05 |
| 1441 | 2.63380972 | 14.25146046 | 2.435886745 | Up | 3.24E-79 |
| 2533 | 2.37801998 | 12.86259996 | 2.435349547 | Up | 3.55E-111 |
| 6367 | 0.51276751 | 2.770451731 | 2.433744472 | Up | 1.20E-15 |
| 100170841 | 0.89495069 | 4.830612023 | 2.432325892 | Up | 4.36E-29 |
| 64065 | 40.2225605 | 216.6475335 | 2.429272981 | Up | 0 |
| 5690 | 71.355264 | 384.1071715 | 2.428417131 | Up | 0 |
| 29992 | 2.01784802 | 10.85566947 | 2.427559274 | Up | 1.05E-26 |
| 84733 | 0.37378602 | 2.005773544 | 2.423874228 | Up | 3.16E-16 |
| 388581 | 0.60310879 | 3.236342719 | 2.423874228 | Up | 3.04E-07 |
| 1143 | 0.12761861 | 0.68481435 | 2.423874228 | Up | 0.0003576 |
| 55362 | 8.69162907 | 46.54902149 | 2.421052329 | Up | 3.56E-262 |
| 113278 | 0.55063049 | 2.947977456 | 2.420569083 | Up | 1.98E-15 |
| 225689 | 0.74462213 | 3.976414642 | 2.416887797 | Up | 1.25E-14 |
| 90993 | 10.3512977 | 55.25957865 | 2.416412914 | Up | 2.67E-259 |
| 79866 | 0.39431527 | 2.102793118 | 2.414885444 | Up | 1.20E-11 |
| 29929 | 1.6454848 | 8.759545078 | 2.41234325 | Up | 3.67E-53 |
| 22801 | 3.29000721 | 17.49080772 | 2.410434263 | Up | 3.01E-154 |
| 100134229 | 1.51018035 | 8.027185124 | 2.410173317 | Up | 6.51E-35 |
| 8877 | 9.08335962 | 48.25830773 | 2.409479422 | Up | 4.31E-185 |
| 5118 | 53.0326216 | 281.5639011 | 2.408510406 | Up | 0 |
| 1978 | 26.8221927 | 142.3820437 | 2.408268128 | Up | 2.35E-214 |
| 81570 | 1.27872616 | 6.785726893 | 2.407796019 | Up | 1.95E-38 |
| 22948 | 29.354455 | 155.5645097 | 2.405861587 | Up | 0 |
| 9735 | 0.90830002 | 4.803391601 | 2.402812612 | Up | 9.24E-60 |
| 9121 | 0.85202824 | 4.505807744 | 2.402812612 | Up | 1.37E-16 |
| 5042 | 0.28938793 | 1.530379329 | 2.402812612 | Up | 7.79E-08 |
| 29968 | 0.92809291 | 4.896958761 | 2.399544898 | Up | 3.90E-20 |
| 7718 | 0.68198919 | 3.597360911 | 2.399118128 | Up | 5.77E-18 |
| 1767 | 0.08525936 | 0.449319616 | 2.397811931 | Up | 1.30E-13 |
| 387644 | 0.30205421 | 1.590651174 | 2.396738098 | Up | 1.99E-11 |
| 2539 | 9.06577016 | 47.71961929 | 2.396081043 | Up | 3.05E-199 |
| 360 | 5.10110208 | 26.84297069 | 2.395663464 | Up | 2.78E-86 |
| 5966 | 1.74766594 | 9.195369495 | 2.395478102 | Up | 6.86E-43 |
| 2710 | 1.15879211 | 6.095306339 | 2.395076963 | Up | 9.81E-41 |
| 65263 | 1.85119001 | 9.734862148 | 2.394707562 | Up | 2.07E-46 |
| 11178 | 0.50084108 | 2.630807822 | 2.393081074 | Up | 2.05E-26 |
| 55194 | 10.1518544 | 53.31755146 | 2.392867238 | Up | 9.54E-99 |
| 10848 | 4.87984771 | 25.52600341 | 2.387059643 | Up | 7.69E-139 |
| 84057 | 0.41499781 | 2.168826632 | 2.385739099 | Up | 8.77E-05 |
| 347902 | 1.29900355 | 6.781166266 | 2.384128049 | Up | 1.49E-46 |
| 5008 | 2.05312598 | 10.71423294 | 2.38363451 | Up | 5.45E-36 |
| 6173 | 707.415921 | 3690.200249 | 2.383068513 | Up | 0 |
| 51337 | 5.14014384 | 26.78572053 | 2.381583469 | Up | 7.79E-106 |
| 126789 | 3.71617154 | 19.33884989 | 2.379612996 | Up | 8.10E-43 |
| 6712 | 0.79433093 | 4.132732982 | 2.379284076 | Up | 1.84E-57 |
| 1717 | 4.3960181 | 22.86469927 | 2.378852713 | Up | 3.42E-106 |
| 4171 | 1.95597629 | 10.16697258 | 2.377929362 | Up | 1.50E-61 |
| 79980 | 2.20863877 | 11.47090882 | 2.376750315 | Up | 1.22E-49 |
| 292 | 118.978709 | 617.3914414 | 2.375482053 | Up | 0 |
| 51571 | 6.79307575 | 35.24914689 | 2.375451494 | Up | 3.47E-134 |
| 79000 | 0.68211867 | 3.536538887 | 2.37424346 | Up | 5.60E-14 |
| 7298 | 3.89772866 | 20.16738667 | 2.371318583 | Up | 3.04E-56 |
| 93594 | 0.62446387 | 3.226056625 | 2.369081755 | Up | 7.35E-21 |
| 7097 | 1.1249344 | 5.806192242 | 2.367751469 | Up | 2.41E-35 |
| 5341 | 5.86043264 | 30.23372462 | 2.367079643 | Up | 1.20E-148 |
| 57465 | 0.34263431 | 1.767085425 | 2.366630276 | Up | 1.47E-14 |
| 898 | 0.92073784 | 4.744635797 | 2.365435002 | Up | 2.57E-17 |
| 26233 | 5.41570956 | 27.90213661 | 2.365153327 | Up | 1.24E-84 |
| 10308 | 1.07612262 | 5.534672529 | 2.362655486 | Up | 2.14E-32 |
| 10553 | 8.51355227 | 43.78465603 | 2.362592252 | Up | 2.17E-127 |
| 2000 | 3.64528595 | 18.73666428 | 2.361760236 | Up | 9.19E-135 |
| 56651 | 0.28006307 | 1.437505569 | 2.35974389 | Up | 8.36E-07 |
| 7226 | 0.21266872 | 1.091584385 | 2.35974389 | Up | 2.22E-12 |
| 79644 | 2.23150252 | 11.45383901 | 2.35974389 | Up | 1.90E-53 |
| 6170 | 701.754677 | 3601.688371 | 2.359634682 | Up | 0 |
| 10535 | 5.5396757 | 28.40209244 | 2.358123794 | Up | 9.50E-57 |
| 55839 | 2.28724203 | 11.70854814 | 2.35588125 | Up | 7.02E-94 |
| 25855 | 12.1203042 | 61.88130903 | 2.352077804 | Up | 4.31E-152 |
| 2892 | 0.1356471 | 0.689215603 | 2.345097114 | Up | 2.13E-07 |
| 8851 | 0.32424531 | 1.645371505 | 2.343255767 | Up | 3.65E-12 |
| 348254 | 0.13004713 | 0.658835996 | 2.340884863 | Up | 5.46E-06 |
| 51657 | 4.90899036 | 24.86224301 | 2.340458219 | Up | 6.43E-61 |
| 11174 | 0.25790618 | 1.303721607 | 2.337717584 | Up | 1.82E-17 |
| 916 | 2.52942949 | 12.7662509 | 2.335450977 | Up | 4.64E-34 |
| 349136 | 0.49746871 | 2.499835694 | 2.32915557 | Up | 5.63E-10 |
| 3157 | 4.51704428 | 22.65552151 | 2.326411741 | Up | 1.90E-134 |
| 153769 | 1.66940751 | 8.364990198 | 2.325027685 | Up | 3.94E-44 |
| 5557 | 1.22870009 | 6.132169749 | 2.319264821 | Up | 1.85E-16 |
| 10578 | 5.29643058 | 26.41085809 | 2.318038858 | Up | 4.53E-45 |
| 8638 | 1.63071338 | 8.129822797 | 2.317720675 | Up | 3.23E-26 |
| 126567 | 0.150053 | 0.746851786 | 2.315349771 | Up | 3.60E-05 |
| 8698 | 0.29943484 | 1.49036302 | 2.315349771 | Up | 3.60E-05 |
| 57478 | 1.62074917 | 8.060065677 | 2.314130763 | Up | 1.03E-144 |
| 9824 | 0.51456477 | 2.556792929 | 2.312910725 | Up | 2.00E-25 |
| 11164 | 8.56274223 | 42.49785934 | 2.311245372 | Up | 1.78E-86 |
| 113802 | 1.66087375 | 8.21492296 | 2.306304631 | Up | 3.68E-27 |
| 57216 | 2.20645291 | 10.90934577 | 2.305763722 | Up | 5.01E-98 |
| 84790 | 57.4328112 | 283.3388649 | 2.302581422 | Up | 0 |
| 10785 | 1.57302637 | 7.749691375 | 2.300596004 | Up | 6.78E-29 |
| 51291 | 1.44346552 | 7.108503495 | 2.300009225 | Up | 7.38E-43 |
| 124045 | 1.49785786 | 7.355371089 | 2.295897408 | Up | 4.18E-28 |
| 152002 | 3.41573245 | 16.74202188 | 2.293206888 | Up | 1.81E-76 |
| 282890 | 0.23158662 | 1.134654852 | 2.292629694 | Up | 2.27E-06 |
| 50512 | 1.29511431 | 6.345391322 | 2.292629694 | Up | 3.28E-24 |
| 6242 | 6.40673654 | 31.36884356 | 2.291670774 | Up | 1.81E-136 |
| 8609 | 0.81009396 | 3.957924292 | 2.288582861 | Up | 4.16E-12 |
| 81792 | 0.34684234 | 1.691991853 | 2.286370704 | Up | 3.08E-15 |
| 1117 | 1.14550463 | 5.588085095 | 2.286370704 | Up | 3.08E-15 |
| 55615 | 2.43949581 | 11.88903017 | 2.284976122 | Up | 7.34E-41 |
| 157285 | 0.64143038 | 3.119048446 | 2.281741378 | Up | 3.71E-25 |
| 57171 | 2.47773728 | 12.03071993 | 2.279627848 | Up | 2.55E-44 |
| 284353 | 0.08333609 | 0.404415221 | 2.278823895 | Up | 0.00024058 |
| 7264 | 23.6378231 | 114.484717 | 2.275985938 | Up | 3.10E-254 |
| 10417 | 25.4425098 | 123.1366577 | 2.274947417 | Up | 0 |
| 10483 | 2.34467111 | 11.34428082 | 2.274507676 | Up | 5.98E-65 |
| 7045 | 52.486053 | 253.536137 | 2.272185376 | Up | 0 |
| 256329 | 0.22411073 | 1.080597829 | 2.269546081 | Up | 5.93E-05 |
| 440905 | 0.62205601 | 2.999376186 | 2.269546081 | Up | 1.41E-23 |
| 9699 | 0.26537002 | 1.277365971 | 2.267094602 | Up | 4.59E-13 |
| 7378 | 5.39664239 | 25.93514443 | 2.26477441 | Up | 9.33E-79 |
| 342897 | 0.83129972 | 3.989818133 | 2.262882351 | Up | 3.12E-14 |
| 54453 | 6.10981604 | 29.31666575 | 2.262520183 | Up | 2.17E-202 |
| 55157 | 1.56842348 | 7.524372094 | 2.262256045 | Up | 2.19E-40 |
| 94240 | 3.9721596 | 19.04449797 | 2.26137876 | Up | 4.99E-50 |
| 255631 | 0.276363 | 1.323198745 | 2.259393365 | Up | 2.13E-15 |
| 6319 | 3.18523723 | 15.23611192 | 2.25802206 | Up | 8.11E-136 |
| 92797 | 0.18551684 | 0.887295346 | 2.257864276 | Up | 3.71E-06 |
| 84440 | 0.29882729 | 1.428183563 | 2.256797588 | Up | 2.03E-21 |
| 57697 | 0.1862716 | 0.889627074 | 2.255792926 | Up | 1.10E-11 |
| 112703 | 0.57381493 | 2.737021271 | 2.253949226 | Up | 9.39E-07 |
| 65108 | 15.2666548 | 72.69935948 | 2.251558679 | Up | 8.06E-182 |
| 9603 | 0.69508776 | 3.302371943 | 2.248235575 | Up | 3.30E-21 |
| 10018 | 1.5164064 | 7.190174039 | 2.245370241 | Up | 2.41E-60 |
| 27165 | 0.41686475 | 1.972951856 | 2.242704469 | Up | 1.03E-09 |
| 4282 | 313.384895 | 1481.53765 | 2.241087741 | Up | 0 |
| 89790 | 0.63917113 | 3.020967907 | 2.24073671 | Up | 7.72E-20 |
| 201255 | 1.11188756 | 5.244351068 | 2.237753366 | Up | 2.56E-23 |
| 11040 | 4.61109102 | 21.64959045 | 2.231159684 | Up | 8.91E-77 |
| 10261 | 3.26693706 | 15.31500229 | 2.228935019 | Up | 1.39E-26 |
| 4233 | 1.76655045 | 8.275787054 | 2.22796157 | Up | 3.19E-89 |
| 116412 | 0.23050444 | 1.075574025 | 2.222240366 | Up | 9.72E-05 |
| 23423 | 16.1494545 | 75.35617821 | 2.222240366 | Up | 9.32E-167 |
| 8458 | 0.72406602 | 3.378618655 | 2.222240366 | Up | 1.57E-27 |
| 3112 | 0.45540868 | 2.125016515 | 2.222240366 | Up | 6.06E-06 |
| 10125 | 0.07771402 | 0.362627196 | 2.222240366 | Up | 0.0003955 |
| 84900 | 0.16170308 | 0.754534849 | 2.222240366 | Up | 9.72E-05 |
| 970 | 0.51327002 | 2.39500766 | 2.222240366 | Up | 9.72E-05 |
| 84848 | 1.59189989 | 7.428083205 | 2.222240366 | Up | 1.12E-10 |
| 23213 | 14.1010971 | 65.70892345 | 2.220281893 | Up | 0 |
| 9289 | 4.71358682 | 21.95836382 | 2.219873352 | Up | 1.22E-141 |
| 1478 | 3.02182633 | 14.0642152 | 2.218536398 | Up | 2.26E-46 |
| 4069 | 17.6709682 | 81.87887676 | 2.212110225 | Up | 1.95E-196 |
| 286262 | 1.75823362 | 8.146651977 | 2.2120805 | Up | 4.74E-34 |
| 664618 | 1.33772549 | 6.194045173 | 2.211099809 | Up | 2.63E-16 |
| 51726 | 18.3117969 | 84.75557283 | 2.210534866 | Up | 4.80E-226 |
| 2537 | 122.875351 | 567.5946291 | 2.2076654 | Up | 0 |
| 55922 | 1.89310189 | 8.742943956 | 2.20736709 | Up | 9.44E-46 |
| 182 | 5.57037 | 25.72448378 | 2.207297064 | Up | 1.32E-242 |
| 1293 | 23.1628317 | 106.9452067 | 2.206988282 | Up | 0 |
| 2357 | 5.51521694 | 25.45824645 | 2.206643511 | Up | 3.46E-54 |
| 7433 | 1.03539798 | 4.77911722 | 2.206558744 | Up | 1.75E-22 |
| 9955 | 0.55633027 | 2.567090587 | 2.206120701 | Up | 1.22E-11 |
| 7852 | 3.62692758 | 16.73156143 | 2.205752244 | Up | 2.63E-51 |
| 375444 | 0.54415738 | 2.509261304 | 2.205166853 | Up | 4.71E-11 |
| 6507 | 1.1986968 | 5.52341223 | 2.20409302 | Up | 1.12E-37 |
| 10926 | 1.19942547 | 5.522105879 | 2.202875041 | Up | 2.28E-35 |
| 57291 | 29.8708144 | 137.2512129 | 2.200010419 | Up | 1.45E-184 |
| 4610 | 0.56064807 | 2.575834485 | 2.199872553 | Up | 4.25E-16 |
| 5984 | 6.20289893 | 28.49077736 | 2.199480463 | Up | 5.00E-66 |
| 4830 | 19.0143062 | 87.16875454 | 2.196725802 | Up | 1.57E-141 |
| 1838 | 1.42062105 | 6.511019679 | 2.196361735 | Up | 1.06E-26 |
| 3364 | 1.96805402 | 9.013223336 | 2.195273319 | Up | 1.07E-31 |
| 3606 | 3.61522897 | 16.55100199 | 2.19475963 | Up | 4.04E-31 |
| 92521 | 2.0381471 | 9.325683699 | 2.193951322 | Up | 6.50E-59 |
| 50615 | 0.23237902 | 1.062634709 | 2.193094021 | Up | 6.31E-07 |
| 51148 | 17.7756727 | 81.26382418 | 2.192709097 | Up | 0 |
| 29100 | 9.04967017 | 41.30935756 | 2.190531507 | Up | 1.40E-52 |
| 7805 | 24.8952 | 113.4645927 | 2.188302656 | Up | 0 |
| 1163 | 15.5697467 | 70.91385013 | 2.18732195 | Up | 4.96E-103 |
| 5792 | 10.2211069 | 46.49642206 | 2.185568269 | Up | 0 |
| 440193 | 0.47979688 | 2.180412244 | 2.184105237 | Up | 7.23E-27 |
| 51005 | 2.25730024 | 10.25207871 | 2.183246235 | Up | 1.53E-34 |
| 165918 | 3.87815097 | 17.59258599 | 2.181526693 | Up | 1.38E-64 |
| 6348 | 5.10217913 | 23.07511652 | 2.177152477 | Up | 6.35E-30 |
| 81849 | 0.99934414 | 4.519634042 | 2.177152477 | Up | 1.10E-15 |
| 79958 | 1.06185988 | 4.798355716 | 2.175946714 | Up | 3.09E-22 |
| 10195 | 18.4800645 | 83.46273612 | 2.175162428 | Up | 1.34E-207 |
| 8459 | 4.52542469 | 20.42467796 | 2.174188237 | Up | 1.52E-64 |
| 340485 | 0.1643112 | 0.741147941 | 2.173330766 | Up | 0.00015855 |
| 8718 | 5.39945122 | 24.35495732 | 2.173330766 | Up | 2.15E-63 |
| 79149 | 0.48763323 | 2.199535824 | 2.173330766 | Up | 6.67E-08 |
| 54836 | 0.76505942 | 3.445730569 | 2.171166182 | Up | 6.16E-14 |
| 150223 | 7.12678815 | 32.05548472 | 2.169247279 | Up | 2.21E-67 |
| 2583 | 1.11575126 | 5.012568025 | 2.167534474 | Up | 1.01E-24 |
| 79899 | 0.31532713 | 1.416195084 | 2.167098812 | Up | 4.74E-10 |
| 57136 | 15.296995 | 68.68740346 | 2.166797277 | Up | 7.16E-235 |
| 503542 | 0.36922119 | 1.653936585 | 2.163346677 | Up | 1.83E-09 |
| 79703 | 2.99230751 | 13.39784949 | 2.1626711 | Up | 3.29E-49 |
| 9168 | 1188.2288 | 5319.16543 | 2.16238725 | Up | 0 |
| 7187 | 1.18659951 | 5.309726695 | 2.161804512 | Up | 2.10E-22 |
| 55320 | 1.16082976 | 5.187762737 | 2.159956088 | Up | 1.88E-39 |
| 10319 | 0.35862126 | 1.601672828 | 2.15904654 | Up | 1.96E-16 |
| 9997 | 11.6641605 | 51.99986174 | 2.156425311 | Up | 4.38E-80 |
| 6472 | 10.2894102 | 45.8551177 | 2.15592247 | Up | 1.09E-147 |
| 8323 | 4.66035689 | 20.7558186 | 2.155003487 | Up | 4.47E-120 |
| 11006 | 2.65960852 | 11.81590718 | 2.151444588 | Up | 4.82E-39 |
| 100049587 | 0.29570313 | 1.310812364 | 2.148239785 | Up | 1.60E-05 |
| 157769 | 1.29095187 | 5.722616788 | 2.148239785 | Up | 8.14E-48 |
| 11077 | 0.32902617 | 1.458528976 | 2.148239785 | Up | 1.60E-05 |
| 25879 | 7.87240942 | 34.85590749 | 2.146526031 | Up | 9.96E-141 |
| 3710 | 2.94204299 | 13.02553193 | 2.146452058 | Up | 2.47E-179 |
| 8317 | 0.91968627 | 4.071344292 | 2.146291513 | Up | 5.39E-22 |
| 79677 | 4.20289646 | 18.59969294 | 2.145822985 | Up | 1.81E-149 |
| 57089 | 0.34691974 | 1.533588774 | 2.144237854 | Up | 2.04E-21 |
| 54502 | 1.18498383 | 5.23160796 | 2.142387059 | Up | 2.92E-42 |
| 84264 | 1.69888052 | 7.498772269 | 2.142070018 | Up | 7.75E-21 |
| 7940 | 5.74283737 | 25.33542417 | 2.141320371 | Up | 3.19E-30 |
| 51373 | 19.7592873 | 86.9672998 | 2.137942132 | Up | 2.98E-78 |
| 3858 | 8.58069576 | 37.75595027 | 2.137537491 | Up | 3.89E-123 |
| 256714 | 0.30957991 | 1.362008037 | 2.137351469 | Up | 1.14E-08 |
| 655 | 3.21633085 | 14.14003175 | 2.136297637 | Up | 2.25E-87 |
| 65993 | 25.6967638 | 112.8565974 | 2.134832172 | Up | 1.45E-171 |
| 1020 | 5.3579612 | 23.52675496 | 2.134546305 | Up | 7.46E-42 |
| 90417 | 2.99803242 | 13.1643309 | 2.134546305 | Up | 7.46E-42 |
| 9424 | 4.41257561 | 19.35445832 | 2.132973028 | Up | 8.59E-79 |
| 114836 | 0.28577603 | 1.253472054 | 2.132973028 | Up | 1.65E-06 |
| 313 | 0.75540771 | 3.310307476 | 2.131637818 | Up | 2.55E-13 |
| 100134868 | 0.65336786 | 2.861114578 | 2.130609891 | Up | 4.44E-08 |
| 9585 | 1.42342276 | 6.226096326 | 2.128963683 | Up | 1.67E-60 |
| 9631 | 1.64992838 | 7.213491766 | 2.128294374 | Up | 1.08E-48 |
| 65999 | 1.35345973 | 5.906831073 | 2.125732385 | Up | 6.79E-19 |
| 8270 | 4.85990775 | 21.19212505 | 2.124527428 | Up | 3.76E-59 |
| 3993 | 1.40884037 | 6.142483616 | 2.124313946 | Up | 3.41E-34 |
| 221002 | 1.97972557 | 8.629406322 | 2.123960849 | Up | 2.40E-43 |
| 81502 | 33.9053006 | 147.733346 | 2.123412764 | Up | 0 |
| 3604 | 0.24873436 | 1.083262134 | 2.122704693 | Up | 0.00025742 |
| 92745 | 2.47383764 | 10.73715609 | 2.117789202 | Up | 2.05E-33 |
| 6676 | 1.07579323 | 4.668450537 | 2.117542988 | Up | 1.43E-11 |
| 9744 | 1.77435135 | 7.698420729 | 2.117270807 | Up | 2.32E-30 |
| 6911 | 0.73763556 | 3.19897625 | 2.116630178 | Up | 5.09E-10 |
| 2307 | 0.82961778 | 3.594631502 | 2.115325162 | Up | 1.84E-08 |
| 10884 | 6.82813047 | 29.54803652 | 2.113499736 | Up | 7.08E-75 |
| 586 | 1.48343895 | 6.419369995 | 2.113486163 | Up | 2.20E-80 |
| 54069 | 3.13205796 | 13.54762279 | 2.112856907 | Up | 3.27E-33 |
| 284338 | 0.40837955 | 1.762652587 | 2.109765637 | Up | 2.58E-05 |
| 10095 | 35.8751222 | 154.7170691 | 2.108576719 | Up | 0 |
| 1564 | 0.60436527 | 2.60314523 | 2.106763149 | Up | 7.13E-08 |
| 80728 | 0.8048682 | 3.458037763 | 2.103129154 | Up | 7.51E-28 |
| 337876 | 0.22366148 | 0.960152078 | 2.101946133 | Up | 2.65E-06 |
| 3654 | 10.6950898 | 45.90458176 | 2.101689562 | Up | 9.74E-243 |
| 5424 | 1.97125767 | 8.458145222 | 2.101224965 | Up | 1.32E-44 |
| 151534 | 1.24377335 | 5.327465862 | 2.09872583 | Up | 8.93E-21 |
| 5226 | 13.5883181 | 58.17498747 | 2.098032094 | Up | 4.19E-167 |
| 9473 | 2.90013287 | 12.40704955 | 2.096969172 | Up | 3.03E-51 |
| 84433 | 0.21437124 | 0.916935877 | 2.096709484 | Up | 2.78E-07 |
| 90407 | 6.2161593 | 26.5563171 | 2.094959702 | Up | 9.52E-112 |
| 57801 | 5.87464019 | 25.09261304 | 2.094690317 | Up | 7.84E-40 |
| 81030 | 0.47582082 | 2.029954853 | 2.092957349 | Up | 2.94E-08 |
| 11035 | 1.38234669 | 5.881129776 | 2.088973835 | Up | 4.39E-18 |
| 340075 | 2.56478345 | 10.8860295 | 2.085568927 | Up | 5.78E-52 |
| 1295 | 13.046482 | 55.33789244 | 2.084606864 | Up | 9.19E-216 |
| 5479 | 85.624141 | 363.1640821 | 2.084532009 | Up | 0 |
| 27338 | 15.0827836 | 63.89180636 | 2.082728217 | Up | 2.08E-112 |
| 9214 | 0.70864306 | 2.999606105 | 2.081642036 | Up | 5.21E-15 |
| 10051 | 10.9038847 | 46.14357228 | 2.08128748 | Up | 0 |
| 200424 | 0.51916672 | 2.196863048 | 2.081175119 | Up | 9.36E-37 |
| 11200 | 1.49365795 | 6.309384716 | 2.078649513 | Up | 8.55E-20 |
| 221035 | 5.17506251 | 21.86006527 | 2.078649513 | Up | 1.68E-74 |
| 91442 | 1.30399752 | 5.508237008 | 2.078649513 | Up | 1.40E-10 |
| 10984 | 0.44790674 | 1.888977824 | 2.076335489 | Up | 2.17E-164 |
| 79101 | 19.464052 | 82.08529881 | 2.076311787 | Up | 1.79E-152 |
| 117289 | 0.62761008 | 2.646530984 | 2.076162107 | Up | 1.98E-14 |
| 25987 | 7.11857538 | 29.93520958 | 2.072182917 | Up | 5.96E-119 |
| 3486 | 27.934069 | 117.4076874 | 2.071429231 | Up | 0 |
| 494514 | 1.11046334 | 4.663454489 | 2.070237273 | Up | 4.44E-07 |
| 100009676 | 0.6176337 | 2.59378813 | 2.070237273 | Up | 6.33E-12 |
| 8541 | 0.7946003 | 3.336969554 | 2.070237273 | Up | 2.31E-24 |
| 29943 | 0.24362648 | 1.023123366 | 2.070237273 | Up | 4.44E-07 |
| 64127 | 0.48759698 | 2.047691499 | 2.070237273 | Up | 8.28E-15 |
| 64175 | 8.74864482 | 36.72696287 | 2.069708135 | Up | 4.97E-145 |
| 963 | 7.80032211 | 32.73471687 | 2.069215896 | Up | 5.70E-76 |
| 3732 | 17.0892712 | 71.63806413 | 2.067635478 | Up | 3.34E-176 |
| 493869 | 5.310976 | 22.21628265 | 2.064568523 | Up | 1.54E-121 |
| 23428 | 2.57501418 | 10.77069862 | 2.064459552 | Up | 4.76E-67 |
| 11332 | 10.6011823 | 44.33855788 | 2.064336671 | Up | 8.26E-117 |
| 29886 | 3.92343879 | 16.40347644 | 2.063811004 | Up | 6.63E-37 |
| 7779 | 2.28347508 | 9.535406117 | 2.062063341 | Up | 2.67E-29 |
| 90268 | 1.51338602 | 6.314011836 | 2.060776944 | Up | 2.79E-73 |
| 9530 | 0.53691055 | 2.238084534 | 2.059510866 | Up | 1.93E-08 |
| 80149 | 7.70673551 | 32.12334887 | 2.059430518 | Up | 8.62E-127 |
| 10609 | 6.51355175 | 27.08791709 | 2.056133118 | Up | 1.43E-102 |
| 65084 | 0.91246417 | 3.791395946 | 2.054889309 | Up | 4.20E-21 |
| 137075 | 0.80023144 | 3.32327762 | 2.054117608 | Up | 9.26E-11 |
| 85465 | 1.46040022 | 6.061303103 | 2.053264194 | Up | 4.12E-72 |
| 5002 | 3.29226215 | 13.66060389 | 2.052870141 | Up | 9.36E-32 |
| 713 | 42.5674068 | 176.4952414 | 2.051808177 | Up | 2.27E-264 |
| 1802 | 8.13242809 | 33.68555414 | 2.050371967 | Up | 1.28E-121 |
| 4082 | 16.5633548 | 68.57355494 | 2.049657408 | Up | 0 |
| 26235 | 0.64687055 | 2.677199416 | 2.049175657 | Up | 4.18E-12 |
| 84959 | 0.6336006 | 2.618606473 | 2.04715366 | Up | 2.25E-27 |
| 10287 | 4.07193142 | 16.80271381 | 2.044909093 | Up | 1.04E-39 |
| 23753 | 6.16599381 | 25.4357669 | 2.044453247 | Up | 2.94E-33 |
| 147968 | 2.59593134 | 10.6921133 | 2.042222897 | Up | 3.57E-49 |
| 2588 | 3.60270085 | 14.80587046 | 2.039018542 | Up | 3.02E-51 |
| 3689 | 4.72788748 | 19.42367141 | 2.038548314 | Up | 5.22E-83 |
| 63928 | 0.33010392 | 1.355483907 | 2.037815795 | Up | 6.80E-06 |
| 11219 | 0.62233138 | 2.555438186 | 2.037815795 | Up | 6.80E-06 |
| 8797 | 0.68551131 | 2.81486974 | 2.037815795 | Up | 3.08E-08 |
| 284992 | 0.21194732 | 0.870305271 | 2.037815795 | Up | 6.80E-06 |
| 6362 | 34.9940168 | 143.5989569 | 2.03686509 | Up | 6.10E-158 |
| 124491 | 1.33382029 | 5.463147296 | 2.034168018 | Up | 1.36E-09 |
| 151354 | 1.11747709 | 4.566073768 | 2.030708909 | Up | 3.23E-18 |
| 147184 | 1.1346623 | 4.623891867 | 2.026844677 | Up | 5.74E-15 |
| 3663 | 2.5745128 | 10.47038845 | 2.023943621 | Up | 5.13E-44 |
| 55353 | 33.9561297 | 137.9597183 | 2.022503151 | Up | 0 |
| 493860 | 0.33069589 | 1.343391511 | 2.022302796 | Up | 5.22E-09 |
| 10525 | 7.41652131 | 30.10869499 | 2.021365619 | Up | 1.69E-195 |
| 29901 | 3.140053 | 12.74494589 | 2.021064431 | Up | 1.29E-29 |
| 2564 | 5.82300388 | 23.60999868 | 2.019562476 | Up | 9.47E-106 |
| 5698 | 16.9172591 | 68.57598619 | 2.019207621 | Up | 3.65E-102 |
| 1265 | 43.2455747 | 175.162656 | 2.018070817 | Up | 0 |
| 79191 | 1.79351697 | 7.263542151 | 2.017881868 | Up | 4.69E-25 |
| 162417 | 0.67849739 | 2.743855783 | 2.015789489 | Up | 2.16E-09 |
| 192669 | 1.27388923 | 5.144798841 | 2.01387483 | Up | 3.87E-27 |
| 374354 | 0.67382625 | 2.71543777 | 2.010736261 | Up | 3.99E-11 |
| 6610 | 2.41746106 | 9.71840886 | 2.007227475 | Up | 2.77E-24 |
| 91057 | 3.90512941 | 15.67781052 | 2.005281831 | Up | 9.34E-48 |
| 80129 | 0.29573112 | 1.186742468 | 2.004648931 | Up | 3.68E-10 |
| 79850 | 6.27073665 | 25.16039986 | 2.004448013 | Up | 3.50E-74 |
| 147138 | 1.00743325 | 4.041089903 | 2.004060196 | Up | 2.28E-26 |
| 23310 | 1.03114925 | 4.135308677 | 2.003741861 | Up | 9.30E-34 |
| 51513 | 1.29690645 | 5.19988666 | 2.003405764 | Up | 3.12E-13 |
| 51050 | 0.19722876 | 0.790378617 | 2.002673989 | Up | 8.26E-09 |
| 148170 | 2.76706313 | 11.0789861 | 2.001398397 | Up | 5.95E-15 |
| 492303 | 0.32122099 | 1.284748527 | 1.999847945 | Up | 0.00026498 |
| 1474 | 0.8977309 | 3.590545145 | 1.999847945 | Up | 0.00026498 |
| 3014 | 10.2469811 | 40.95092246 | 1.998697012 | Up | 1.08E-91 |
| 3936 | 9.7705534 | 39.03137684 | 1.998122172 | Up | 1.99E-205 |
| 100134934 | 6.20734856 | 24.78487572 | 1.99741096 | Up | 1.22E-35 |
| 8870 | 42.9564235 | 171.3457369 | 1.995964511 | Up | 7.66E-294 |
| 241 | 20.9347144 | 83.49148505 | 1.995731837 | Up | 4.80E-102 |
| 57210 | 0.76344355 | 3.044199458 | 1.995469504 | Up | 1.03E-15 |
| 728 | 4.67080835 | 18.58785804 | 1.992616376 | Up | 1.66E-61 |
| 2207 | 63.9621873 | 254.4299172 | 1.99197714 | Up | 5.29E-208 |
| 54863 | 1.49717422 | 5.947121667 | 1.989949484 | Up | 7.14E-35 |
| 5358 | 18.650677 | 74.06991894 | 1.989659757 | Up | 0 |
| 374659 | 3.81170526 | 15.13560806 | 1.989438162 | Up | 2.57E-23 |
| 1174 | 12.5855366 | 49.95951509 | 1.988992748 | Up | 1.60E-90 |
| 84966 | 0.32157525 | 1.27544738 | 1.987775113 | Up | 0.00010584 |
| 10682 | 13.0958532 | 51.90991077 | 1.986899956 | Up | 6.38E-84 |
| 4796 | 1.83363773 | 7.248447804 | 1.982963452 | Up | 2.18E-46 |
| 125058 | 0.62309521 | 2.460167938 | 1.981232267 | Up | 1.86E-12 |
| 4311 | 0.60184131 | 2.374287278 | 1.980039464 | Up | 4.42E-20 |
| 80115 | 0.80105219 | 3.160181902 | 1.980039464 | Up | 9.92E-11 |
| 90522 | 3.44135328 | 13.57399103 | 1.979797055 | Up | 1.94E-55 |
| 290 | 2.26818475 | 8.946261307 | 1.979746739 | Up | 3.41E-46 |
| 2281 | 2.435795 | 9.60597254 | 1.979538963 | Up | 1.47E-14 |
| 95681 | 1.39548016 | 5.498645966 | 1.978314783 | Up | 4.80E-28 |
| 10970 | 26.8147931 | 105.597637 | 1.977476526 | Up | 0 |
| 84823 | 4.48226288 | 17.64410152 | 1.976886797 | Up | 7.06E-113 |
| 158219 | 0.73985154 | 2.911920282 | 1.976663158 | Up | 4.11E-11 |
| 7371 | 7.83533188 | 30.82825925 | 1.976187138 | Up | 6.37E-54 |
| 9600 | 3.17956099 | 12.50741969 | 1.975884702 | Up | 1.60E-73 |
| 27065 | 1.0500348 | 4.129701588 | 1.975600399 | Up | 3.97E-19 |
| 54733 | 3.22962091 | 12.7001934 | 1.975413727 | Up | 2.26E-56 |
| 7305 | 68.3052671 | 268.2435378 | 1.973474681 | Up | 1.85E-207 |
| 388152 | 2.74615775 | 10.77249135 | 1.971865532 | Up | 1.05E-50 |
| 246269 | 0.34635294 | 1.357560439 | 1.970701599 | Up | 1.71E-05 |
| 6119 | 4.16481807 | 16.3243662 | 1.970701599 | Up | 6.32E-37 |
| 91373 | 2.80844374 | 11.00360706 | 1.970133721 | Up | 7.78E-52 |
| 83855 | 5.98082883 | 23.41207124 | 1.968835241 | Up | 4.39E-93 |
| 140885 | 8.79374535 | 34.40538819 | 1.96808486 | Up | 6.86E-197 |
| 3017 | 2.49774575 | 9.772200791 | 1.968056878 | Up | 2.93E-12 |
| 3875 | 11.4465279 | 44.7662903 | 1.967502718 | Up | 1.92E-90 |
| 64116 | 1.60799446 | 6.288382204 | 1.967426467 | Up | 2.35E-36 |
| 79693 | 5.48760015 | 21.4376666 | 1.965900613 | Up | 7.63E-55 |
| 8928 | 0.39391493 | 1.537299888 | 1.964442609 | Up | 6.94E-06 |
| 118881 | 7.20946968 | 28.1086287 | 1.963048027 | Up | 1.29E-38 |
| 29933 | 0.36516612 | 1.423281826 | 1.96259655 | Up | 2.05E-08 |
| 80774 | 3.1967571 | 12.45312391 | 1.961826661 | Up | 8.87E-56 |
| 145173 | 0.66738504 | 2.595112739 | 1.959205961 | Up | 2.89E-16 |
| 23231 | 1.35636502 | 5.271526459 | 1.958475297 | Up | 6.89E-34 |
| 875 | 2.41429948 | 9.378564965 | 1.957762544 | Up | 2.89E-34 |
| 55920 | 9.06837678 | 35.22359017 | 1.957625724 | Up | 6.19E-195 |
| 53827 | 22.9375474 | 88.97617431 | 1.955707931 | Up | 4.76E-196 |
| 4486 | 0.42562718 | 1.649949943 | 1.954760055 | Up | 4.59E-12 |
| 64218 | 1.81352759 | 7.018015067 | 1.952264351 | Up | 5.42E-32 |
| 4061 | 91.4517551 | 353.7178531 | 1.951516275 | Up | 0 |
| 5654 | 58.6874637 | 226.7781539 | 1.950157403 | Up | 0 |
| 6167 | 496.623018 | 1918.720317 | 1.949921397 | Up | 0 |
| 10900 | 1.14519924 | 4.417458573 | 1.947617986 | Up | 1.36E-13 |
| 353 | 41.4224384 | 159.6853798 | 1.946747844 | Up | 2.91E-173 |
| 1728 | 9.97759811 | 38.42728629 | 1.945366633 | Up | 3.55E-134 |
| 23105 | 0.11609452 | 0.446916795 | 1.944706391 | Up | 0.00016789 |
| 3109 | 9.31709815 | 35.85362915 | 1.944166561 | Up | 1.77E-66 |
| 57168 | 0.58936454 | 2.266064245 | 1.942956609 | Up | 1.73E-11 |
| 8208 | 1.43936319 | 5.532968625 | 1.942623076 | Up | 2.38E-18 |
| 11151 | 7.90668177 | 30.37002769 | 1.941503959 | Up | 4.87E-67 |
| 149428 | 0.84190186 | 3.233425833 | 1.94133954 | Up | 7.18E-12 |
| 6581 | 1.62482269 | 6.233847789 | 1.939840636 | Up | 3.32E-48 |
| 9123 | 13.5747926 | 52.05783519 | 1.939185163 | Up | 2.34E-143 |
| 29841 | 0.99686988 | 3.822376404 | 1.93899274 | Up | 7.22E-20 |
| 54517 | 2.08602548 | 7.996335837 | 1.938582288 | Up | 1.38E-38 |
| 3932 | 1.43882898 | 5.508781593 | 1.936838147 | Up | 5.11E-17 |
| 375791 | 1.05544038 | 4.038392871 | 1.935936181 | Up | 2.71E-05 |
| 51256 | 5.55260588 | 21.24003811 | 1.935549452 | Up | 1.22E-37 |
| 348654 | 0.53973832 | 2.060601847 | 1.932733749 | Up | 6.46E-19 |
| 79017 | 19.9781728 | 76.2665691 | 1.932626162 | Up | 6.55E-119 |
| 152217 | 8.47842618 | 32.33065205 | 1.931034211 | Up | 5.34E-61 |
| 1051 | 31.7172187 | 120.8584923 | 1.929980678 | Up | 1.10E-293 |
| 2124 | 1.91830918 | 7.307074331 | 1.929458617 | Up | 8.21E-21 |
| 59269 | 0.4313601 | 1.643103392 | 1.929458617 | Up | 8.21E-21 |
| 388753 | 11.9408013 | 45.37026289 | 1.925847364 | Up | 6.76E-40 |
| 63922 | 2.4517641 | 9.293818182 | 1.922451249 | Up | 2.50E-39 |
| 51060 | 25.0573032 | 94.85932851 | 1.920558506 | Up | 5.54E-200 |
| 7422 | 26.2051924 | 99.07909854 | 1.918728039 | Up | 0 |
| 4794 | 3.11684099 | 11.77618121 | 1.917715318 | Up | 2.13E-41 |
| 1611 | 16.7076843 | 63.05346825 | 1.916063937 | Up | 3.81E-194 |
| 8444 | 1.5889956 | 5.99609923 | 1.915909127 | Up | 2.73E-19 |
| 6636 | 39.3960092 | 148.5335629 | 1.914667564 | Up | 4.23E-155 |
| 55775 | 1.31317402 | 4.940904425 | 1.911717035 | Up | 9.95E-26 |
| 78999 | 3.30576619 | 12.42672335 | 1.910389344 | Up | 1.60E-42 |
| 11118 | 11.2892251 | 42.42880888 | 1.910097715 | Up | 1.42E-211 |
| 50814 | 8.4810786 | 31.74878968 | 1.904381936 | Up | 1.12E-68 |
| 3704 | 13.537782 | 50.60051547 | 1.902160696 | Up | 2.95E-75 |
| 84953 | 0.2565788 | 0.957793252 | 1.900312271 | Up | 4.26E-05 |
| 138429 | 0.40415311 | 1.508679254 | 1.900312271 | Up | 0.00026498 |
| 114805 | 0.12410369 | 0.463271609 | 1.900312271 | Up | 0.00010596 |
| 159013 | 0.58547667 | 2.185549219 | 1.900312271 | Up | 4.26E-05 |
| 8364 | 8.21078492 | 30.65036641 | 1.900312271 | Up | 5.22E-17 |
| 256281 | 8.13568627 | 30.37002769 | 1.900312271 | Up | 1.52E-35 |
| 728554 | 1.53896726 | 5.744872233 | 1.900312271 | Up | 4.16E-15 |
| 51427 | 0.25537631 | 0.953304411 | 1.900312271 | Up | 3.24E-08 |
| 122509 | 6.01630582 | 22.45850787 | 1.900312271 | Up | 2.55E-22 |
| 302 | 246.50835 | 918.7940825 | 1.89810505 | Up | 0 |
| 51280 | 10.1244096 | 37.72277123 | 1.897597887 | Up | 4.07E-151 |
| 7464 | 1.0799507 | 4.018469893 | 1.895680823 | Up | 7.98E-31 |
| 85019 | 2.24492734 | 8.331456794 | 1.891900032 | Up | 1.18E-33 |
| 5307 | 5.50618331 | 20.43190449 | 1.891699141 | Up | 3.72E-64 |
| 80179 | 2.92884706 | 10.86694809 | 1.891542062 | Up | 5.00E-63 |
| 85301 | 1.06319124 | 3.942718264 | 1.890789497 | Up | 7.03E-30 |
| 90353 | 1.40925848 | 5.226067443 | 1.890789497 | Up | 1.12E-15 |
| 126321 | 10.3954408 | 38.45451842 | 1.887202189 | Up | 6.16E-114 |
| 5996 | 5.17714947 | 19.11818136 | 1.884715416 | Up | 6.46E-36 |
| 1009 | 6.70123632 | 24.73558834 | 1.884089024 | Up | 9.47E-117 |
| 6890 | 15.2924444 | 56.44380756 | 1.883996277 | Up | 6.06E-214 |
| 1956 | 4.08643899 | 15.07221088 | 1.882974955 | Up | 3.07E-109 |
| 161882 | 1.44589796 | 5.329982027 | 1.882164925 | Up | 1.22E-23 |
| 79174 | 7.38875279 | 27.21154481 | 1.880816096 | Up | 2.98E-56 |
| 6510 | 10.5551391 | 38.8685579 | 1.880657997 | Up | 1.38E-143 |
| 3920 | 8.68992064 | 31.98533699 | 1.879995777 | Up | 2.90E-269 |
| 79669 | 0.58285514 | 2.14376666 | 1.878938621 | Up | 1.23E-07 |
| 161497 | 0.22658956 | 0.832628864 | 1.877592195 | Up | 3.01E-07 |
| 79064 | 9.33563488 | 34.30484238 | 1.877592195 | Up | 1.64E-42 |
| 10969 | 33.5170788 | 123.1289426 | 1.877201601 | Up | 2.96E-236 |
| 114299 | 0.25859022 | 0.949732362 | 1.876853299 | Up | 8.06E-13 |
| 83990 | 0.2567475 | 0.942449248 | 1.876064725 | Up | 7.35E-07 |
| 5360 | 66.7340739 | 244.81201 | 1.875178851 | Up | 0 |
| 3106 | 294.722998 | 1080.19929 | 1.873865957 | Up | 0 |
| 199675 | 0.77624897 | 2.841965894 | 1.872297895 | Up | 4.42E-06 |
| 5898 | 13.3339064 | 48.80943892 | 1.872060659 | Up | 5.59E-174 |
| 4646 | 5.29279835 | 19.37062214 | 1.871767696 | Up | 1.47E-213 |
| 8743 | 26.7298227 | 97.75039725 | 1.870652298 | Up | 1.27E-218 |
| 5905 | 12.6422263 | 46.22758534 | 1.870503462 | Up | 6.43E-178 |
| 166815 | 0.4495113 | 1.643040347 | 1.869938622 | Up | 1.09E-05 |
| 9891 | 1.31678605 | 4.808624821 | 1.868603412 | Up | 4.47E-43 |
| 84283 | 1.12276856 | 4.092995645 | 1.866096556 | Up | 5.19E-13 |
| 223082 | 1.8735592 | 6.825356512 | 1.865122843 | Up | 2.18E-31 |
| 118788 | 0.50325925 | 1.833185625 | 1.864979198 | Up | 1.25E-12 |
| 6187 | 861.532903 | 3136.817637 | 1.864323859 | Up | 0 |
| 957 | 0.38173308 | 1.38936197 | 1.863786395 | Up | 6.67E-05 |
| 623 | 2.42366449 | 8.821208973 | 1.863786395 | Up | 7.21E-16 |
| 4174 | 6.04328746 | 21.98665948 | 1.863222953 | Up | 4.79E-72 |
| 64005 | 0.92894204 | 3.378769778 | 1.862837566 | Up | 1.73E-15 |
| 23406 | 17.5035389 | 63.62229581 | 1.86188579 | Up | 2.45E-147 |
| 254359 | 2.78726017 | 10.12795456 | 1.861423258 | Up | 2.44E-18 |
| 5357 | 0.58802702 | 2.136272988 | 1.861141674 | Up | 1.75E-11 |
| 4016 | 19.2196543 | 69.80089718 | 1.860663195 | Up | 4.03E-208 |
| 5836 | 7.56187603 | 27.45536355 | 1.860271911 | Up | 4.25E-99 |
| 3692 | 59.1012262 | 214.0975752 | 1.857008487 | Up | 0 |
| 6662 | 0.83383546 | 3.020019022 | 1.856723003 | Up | 1.93E-16 |
| 5771 | 6.09781534 | 22.08461237 | 1.856677143 | Up | 5.57E-70 |
| 7040 | 20.3412964 | 73.6647935 | 1.856563649 | Up | 1.67E-217 |
| 90850 | 8.01302751 | 29.0001941 | 1.855643222 | Up | 1.68E-117 |
| 142678 | 12.399147 | 44.86974991 | 1.855502267 | Up | 2.30E-183 |
| 140700 | 1.71890153 | 6.21603868 | 1.854508582 | Up | 1.57E-18 |
| 23233 | 0.24087151 | 0.871060162 | 1.854508582 | Up | 0.00041607 |
| 79172 | 0.38450505 | 1.390480037 | 1.854508582 | Up | 0.00041607 |
| 84804 | 0.23090196 | 0.835007413 | 1.854508582 | Up | 0.00041607 |
| 11184 | 0.64640501 | 2.334306554 | 1.852483746 | Up | 1.44E-09 |
| 81622 | 6.64631096 | 23.99166593 | 1.851907639 | Up | 6.36E-71 |
| 4010 | 0.40749176 | 1.470437515 | 1.851402671 | Up | 4.65E-12 |
| 440173 | 1.31786005 | 4.738628638 | 1.846272432 | Up | 2.14E-13 |
| 50509 | 5.09804714 | 18.32236462 | 1.845589085 | Up | 6.52E-142 |
| 203427 | 4.89724337 | 17.59819983 | 1.845386061 | Up | 2.80E-62 |
| 3118 | 2.10469775 | 7.557813569 | 1.844355865 | Up | 1.39E-17 |
| 10863 | 0.31532101 | 1.131802274 | 1.843728743 | Up | 6.88E-06 |
| 254427 | 0.30481346 | 1.094086858 | 1.843728743 | Up | 6.88E-06 |
| 54620 | 2.11922197 | 7.60370767 | 1.843168364 | Up | 1.97E-37 |
| 8532 | 6.23692418 | 22.37534945 | 1.843003588 | Up | 9.66E-71 |
| 6502 | 1.22494649 | 4.389745533 | 1.841418582 | Up | 3.81E-10 |
| 4324 | 3.62612873 | 12.99401861 | 1.841345629 | Up | 5.34E-73 |
| 51547 | 4.78737406 | 17.14763034 | 1.840702782 | Up | 5.35E-38 |
| 113878 | 3.93422048 | 14.09152047 | 1.840677572 | Up | 8.32E-51 |
| 54852 | 0.25672874 | 0.918421582 | 1.838911727 | Up | 1.22E-07 |
| 375035 | 1.0948961 | 3.916882076 | 1.838911727 | Up | 1.70E-05 |
| 7074 | 0.44487443 | 1.589806395 | 1.837381016 | Up | 1.10E-15 |
| 637 | 5.77143624 | 20.60771035 | 1.836181934 | Up | 6.41E-65 |
| 834 | 6.16751066 | 22.00089976 | 1.834802318 | Up | 2.23E-38 |
| 130502 | 1.04899691 | 3.737849562 | 1.833198076 | Up | 4.19E-05 |
| 6303 | 89.5647428 | 319.0407687 | 1.832737959 | Up | 0 |
| 23594 | 2.78252464 | 9.894510249 | 1.830233836 | Up | 2.21E-21 |
| 23246 | 11.7499469 | 41.773186 | 1.829922944 | Up | 3.59E-123 |
| 1043 | 10.5167842 | 37.38219646 | 1.829657718 | Up | 8.66E-25 |
| 56925 | 6.15862703 | 21.87948231 | 1.828897938 | Up | 1.36E-31 |
| 1951 | 0.09793837 | 0.347318156 | 1.82631169 | Up | 1.77E-06 |
| 2187 | 0.25964956 | 0.920793393 | 1.82631169 | Up | 0.00010397 |
| 117247 | 0.27034472 | 0.958721539 | 1.82631169 | Up | 0.00010397 |
| 359821 | 3.51286004 | 12.45763055 | 1.82631169 | Up | 1.11E-11 |
| 219833 | 0.21551487 | 0.764278842 | 1.82631169 | Up | 0.00010397 |
| 8989 | 0.30097337 | 1.067339701 | 1.82631169 | Up | 3.18E-08 |
| 27161 | 2.32142589 | 8.228289868 | 1.825581396 | Up | 7.06E-37 |
| 11116 | 2.03025296 | 7.192138122 | 1.824761238 | Up | 3.69E-18 |
| 441478 | 6.70472428 | 23.74910673 | 1.82462334 | Up | 1.01E-77 |
| 6748 | 93.4288418 | 330.5708383 | 1.823019573 | Up | 3.94E-258 |
| 56937 | 23.9520961 | 84.7394228 | 1.822881384 | Up | 0 |
| 29766 | 2.10290389 | 7.436855151 | 1.822309759 | Up | 8.13E-21 |
| 120224 | 0.31849719 | 1.122879139 | 1.817850111 | Up | 0.00025918 |
| 55974 | 19.3657199 | 68.27489769 | 1.817850111 | Up | 4.62E-113 |
| 629 | 8.56244591 | 30.15962247 | 1.816523495 | Up | 7.57E-98 |
| 2896 | 77.5943284 | 273.2432895 | 1.816162954 | Up | 0 |
| 3619 | 1.0737693 | 3.779771898 | 1.815615107 | Up | 1.25E-20 |
| 9526 | 10.3427932 | 36.39892922 | 1.815270148 | Up | 4.89E-73 |
| 1534 | 6.76030646 | 23.75679528 | 1.81317968 | Up | 2.09E-92 |
| 10016 | 26.9058255 | 94.52972143 | 1.81284943 | Up | 1.74E-131 |
| 81552 | 10.6648235 | 37.45911839 | 1.812456852 | Up | 4.69E-135 |
| 55969 | 36.7108912 | 128.927008 | 1.812274471 | Up | 1.09E-183 |
| 113000 | 6.3244047 | 22.18641633 | 1.810675059 | Up | 1.12E-56 |
| 26517 | 14.5743582 | 51.12598526 | 1.810624384 | Up | 1.77E-122 |
| 2232 | 2.5061851 | 8.780308899 | 1.808778722 | Up | 8.99E-22 |
| 199990 | 14.249169 | 49.89183149 | 1.807925841 | Up | 1.44E-45 |
| 5499 | 37.3104846 | 130.5971809 | 1.807470752 | Up | 5.75E-238 |
| 10465 | 18.7446212 | 65.59925981 | 1.807202867 | Up | 5.78E-65 |
| 64420 | 0.72993073 | 2.55448831 | 1.807202867 | Up | 9.88E-11 |
| 10940 | 0.70792818 | 2.473646437 | 1.804964395 | Up | 1.07E-15 |
| 112597 | 19.6227896 | 68.5365401 | 1.804343213 | Up | 5.56E-68 |
| 100131187 | 10.2125227 | 35.63175908 | 1.802824427 | Up | 1.76E-30 |
| 11322 | 3.80922343 | 13.2867558 | 1.802420071 | Up | 7.72E-48 |
| 57106 | 8.80364285 | 30.69847734 | 1.801994576 | Up | 4.23E-50 |
| 135228 | 1.48829831 | 5.185346248 | 1.800776598 | Up | 1.84E-60 |
| 4157 | 1.38613822 | 4.821572644 | 1.798432657 | Up | 1.67E-19 |
| 441250 | 0.54426891 | 1.893196531 | 1.798432657 | Up | 1.28E-08 |
| 57673 | 0.42217615 | 1.466516652 | 1.79647646 | Up | 3.18E-13 |
| 9028 | 1.25244689 | 4.348037319 | 1.795614893 | Up | 1.39E-09 |
| 54885 | 0.43770277 | 1.519033259 | 1.795130035 | Up | 6.92E-12 |
| 11177 | 16.1502027 | 56.02300852 | 1.794467184 | Up | 0 |
| 9743 | 1.96222131 | 6.805770003 | 1.794270634 | Up | 8.20E-84 |
| 10420 | 0.88868355 | 3.080444941 | 1.793397068 | Up | 7.63E-13 |
| 30851 | 44.2260854 | 153.2179992 | 1.792616331 | Up | 1.72E-253 |
| 57761 | 2.19782977 | 7.609847068 | 1.791787815 | Up | 7.16E-24 |
| 57332 | 1.23238798 | 4.264982192 | 1.791083202 | Up | 3.36E-09 |
| 9047 | 1.26047214 | 4.356728419 | 1.789280959 | Up | 3.66E-10 |
| 57125 | 5.85988064 | 20.25381082 | 1.789250198 | Up | 4.43E-152 |
| 3838 | 10.6897987 | 36.92259124 | 1.788269121 | Up | 1.55E-88 |
| 84106 | 0.36108455 | 1.246813897 | 1.787837542 | Up | 0.00016133 |
| 151507 | 1.1762438 | 4.061533823 | 1.787837542 | Up | 4.00E-11 |
| 202309 | 0.3524485 | 1.216993889 | 1.787837542 | Up | 0.00016133 |
| 6627 | 16.0777755 | 55.47477012 | 1.786763975 | Up | 2.04E-71 |
| 153020 | 0.79169658 | 2.730491692 | 1.786141252 | Up | 8.12E-09 |
| 113179 | 1.28523246 | 4.428642012 | 1.784835054 | Up | 8.82E-10 |
| 112399 | 7.66105476 | 26.37573308 | 1.783596254 | Up | 1.13E-86 |
| 163486 | 0.46506938 | 1.600445077 | 1.782955321 | Up | 1.72E-06 |
| 706 | 41.7569265 | 143.5878076 | 1.78184582 | Up | 1.48E-148 |
| 80381 | 13.0668547 | 44.87727467 | 1.780073156 | Up | 1.18E-181 |
| 4116 | 30.0482792 | 103.1778209 | 1.779778606 | Up | 1.28E-84 |
| 54491 | 0.31037077 | 1.065494017 | 1.779457877 | Up | 2.32E-10 |
| 3676 | 0.43704542 | 1.499507621 | 1.778633715 | Up | 2.80E-12 |
| 144347 | 1.89075167 | 6.48120144 | 1.777301381 | Up | 5.31E-18 |
| 645840 | 1.03310302 | 3.535135498 | 1.774781389 | Up | 4.22E-06 |
| 3957 | 1.77505882 | 6.074005538 | 1.774781389 | Up | 4.06E-05 |
| 9363 | 0.84587641 | 2.894471953 | 1.774781389 | Up | 4.06E-05 |
| 56603 | 1.32815797 | 4.539406965 | 1.773077083 | Up | 8.68E-26 |
| 219855 | 2.1969262 | 7.505993971 | 1.772556724 | Up | 1.19E-38 |
| 6632 | 18.2076659 | 62.18263169 | 1.771965687 | Up | 2.42E-118 |
| 92421 | 1.60861699 | 5.491296063 | 1.771325831 | Up | 1.97E-13 |
| 26872 | 8.80364285 | 30.03232593 | 1.77034369 | Up | 6.61E-48 |
| 10549 | 33.5815689 | 114.5157787 | 1.769804858 | Up | 3.95E-125 |
| 6520 | 29.4757474 | 100.4870507 | 1.769409299 | Up | 2.80E-283 |
| 80380 | 0.7429113 | 2.532091639 | 1.769067738 | Up | 1.24E-08 |
| 134145 | 3.59627302 | 12.24014117 | 1.767045741 | Up | 4.54E-28 |
| 84259 | 9.31728234 | 31.68792641 | 1.765952139 | Up | 6.44E-48 |
| 54951 | 3.26580631 | 11.10626101 | 1.765862072 | Up | 6.48E-20 |
| 80765 | 5.28201394 | 17.94508627 | 1.764428843 | Up | 3.28E-29 |
| 714 | 39.4119694 | 133.8423185 | 1.763828596 | Up | 2.90E-187 |
| 79627 | 3.0971716 | 10.51431303 | 1.763331369 | Up | 5.89E-23 |
| 1942 | 19.6503319 | 66.69770066 | 1.763083346 | Up | 2.70E-123 |
| 84318 | 1.08888273 | 3.695212494 | 1.762808748 | Up | 1.03E-11 |
| 10327 | 27.1196609 | 92.02415165 | 1.762673404 | Up | 5.12E-166 |
| 1175 | 33.4349282 | 113.4269349 | 1.762335345 | Up | 3.69E-119 |
| 51330 | 80.1459699 | 271.6351779 | 1.760968445 | Up | 0 |
| 92960 | 1.17668683 | 3.980704194 | 1.758293267 | Up | 2.65E-06 |
| 1736 | 15.0383866 | 50.87451222 | 1.758293267 | Up | 1.22E-153 |
| 951 | 6.28339406 | 21.22725668 | 1.756301969 | Up | 2.97E-32 |
| 1909 | 1.66638175 | 5.628878972 | 1.756128682 | Up | 8.58E-30 |
| 6509 | 5.43922725 | 18.371313 | 1.755981131 | Up | 2.28E-100 |
| 81567 | 34.1104335 | 115.1513093 | 1.755245821 | Up | 0 |
| 5033 | 9.2808319 | 31.31928494 | 1.75472524 | Up | 4.22E-108 |
| 10105 | 22.2947388 | 75.2072322 | 1.754168105 | Up | 7.82E-193 |
| 79006 | 12.2303504 | 41.24983981 | 1.753922784 | Up | 8.32E-54 |
| 5558 | 1.50592195 | 5.078528732 | 1.753763608 | Up | 5.64E-15 |
| 55033 | 3.23255038 | 10.89915012 | 1.753470883 | Up | 5.48E-30 |
| 344148 | 0.18512597 | 0.623877314 | 1.752755083 | Up | 6.82E-07 |
| 6665 | 11.3175917 | 38.11721696 | 1.751875793 | Up | 4.61E-63 |
| 54584 | 1.1825935 | 3.982692242 | 1.751789747 | Up | 1.90E-08 |
| 6051 | 16.0218737 | 53.94864761 | 1.751543922 | Up | 8.02E-152 |
| 2123 | 2.32529597 | 7.829191026 | 1.751448886 | Up | 4.47E-17 |
| 23446 | 6.2275632 | 20.96736009 | 1.751405566 | Up | 1.62E-111 |
| 79690 | 3.44389365 | 11.59322002 | 1.751168832 | Up | 1.13E-35 |
| 9263 | 6.91067905 | 23.2582058 | 1.750840424 | Up | 3.47E-107 |
| 9722 | 0.53389069 | 1.796306009 | 1.750416841 | Up | 4.58E-13 |
| 26115 | 1.21006819 | 4.070364484 | 1.750069636 | Up | 7.68E-57 |
| 285958 | 13.2278356 | 44.47715138 | 1.749487369 | Up | 7.29E-43 |
| 10388 | 0.28504594 | 0.957653428 | 1.748309178 | Up | 1.77E-07 |
| 91687 | 0.40080014 | 1.346546558 | 1.748309178 | Up | 6.47E-06 |
| 54498 | 3.46884206 | 11.64740622 | 1.747482655 | Up | 6.02E-31 |
| 2729 | 6.47351915 | 21.69381095 | 1.744661401 | Up | 3.19E-94 |
| 112724 | 1.90022792 | 6.342013205 | 1.738768411 | Up | 3.45E-19 |
| 26232 | 4.06784314 | 13.57160612 | 1.738255513 | Up | 1.83E-25 |
| 339761 | 0.70017262 | 2.333662747 | 1.736813539 | Up | 4.90E-14 |
| 148304 | 0.70047164 | 2.334659399 | 1.736813539 | Up | 1.58E-05 |
| 10591 | 30.5746644 | 101.7872245 | 1.73514793 | Up | 4.23E-94 |
| 65109 | 21.9120239 | 72.88806645 | 1.733959884 | Up | 5.92E-199 |
| 23170 | 6.77836361 | 22.54247686 | 1.733637107 | Up | 1.08E-87 |
| 23526 | 2.12474208 | 7.059749401 | 1.732329248 | Up | 6.54E-36 |
| 79783 | 3.30284577 | 10.96947269 | 1.731712667 | Up | 1.13E-21 |
| 5471 | 1.16788917 | 3.873090848 | 1.72958197 | Up | 4.66E-18 |
| 84231 | 8.20002631 | 27.19140303 | 1.729450151 | Up | 1.03E-114 |
| 11270 | 13.0446183 | 43.25000138 | 1.729245446 | Up | 2.71E-71 |
| 5832 | 8.36732897 | 27.74082344 | 1.72917155 | Up | 1.44E-110 |
| 126129 | 1.99112523 | 6.595257718 | 1.727845075 | Up | 1.92E-22 |
| 221336 | 0.89739816 | 2.971715089 | 1.727475675 | Up | 1.37E-10 |
| 3105 | 315.082284 | 1043.306356 | 1.727362311 | Up | 0 |
| 8875 | 0.42870682 | 1.418480335 | 1.726282872 | Up | 0.00015434 |
| 11260 | 5.27147027 | 17.43508184 | 1.72571583 | Up | 6.29E-78 |
| 5716 | 11.669921 | 38.56561343 | 1.724520263 | Up | 4.71E-69 |
| 24139 | 3.40482813 | 11.25096044 | 1.72439428 | Up | 3.05E-30 |
| 9454 | 11.7094744 | 38.67004434 | 1.723540105 | Up | 3.82E-88 |
| 10265 | 0.49240235 | 1.626018941 | 1.723434509 | Up | 3.89E-05 |
| 3101 | 0.71280067 | 2.351994322 | 1.722313969 | Up | 1.25E-09 |
| 55144 | 2.70315431 | 8.916030322 | 1.721757653 | Up | 9.83E-40 |
| 126014 | 1.29095187 | 4.254313796 | 1.720491234 | Up | 8.68E-11 |
| 100129424 | 1.17085009 | 3.856511453 | 1.719740026 | Up | 2.54E-06 |
| 5478 | 291.40913 | 959.6822001 | 1.719510658 | Up | 0 |
| 2004 | 1.30715629 | 4.303479516 | 1.719071957 | Up | 6.05E-12 |
| 10347 | 0.93933884 | 3.089564565 | 1.717685949 | Up | 2.56E-25 |
| 51236 | 6.81976862 | 22.42709737 | 1.717448214 | Up | 2.76E-62 |
| 54503 | 2.02155426 | 6.64796662 | 1.717448214 | Up | 8.55E-20 |
| 2760 | 15.6289886 | 51.34576915 | 1.716020981 | Up | 8.58E-214 |
| 90835 | 1.06755861 | 3.506916265 | 1.7158877 | Up | 1.15E-08 |
| 3902 | 1.05861942 | 3.476087507 | 1.715280378 | Up | 3.01E-09 |
| 79786 | 2.37656562 | 7.79623394 | 1.713899147 | Up | 1.45E-20 |
| 51126 | 16.720758 | 54.85181646 | 1.713899147 | Up | 5.54E-68 |
| 286827 | 1.2291687 | 4.024263731 | 1.71104192 | Up | 4.83E-19 |
| 3614 | 12.7728093 | 41.81781829 | 1.71104192 | Up | 1.35E-123 |
| 191 | 22.4656116 | 73.5028962 | 1.710082757 | Up | 5.71E-196 |
| 58505 | 26.5404166 | 86.79079024 | 1.709350946 | Up | 3.17E-106 |
| 51691 | 8.30624312 | 27.15875162 | 1.709149161 | Up | 2.68E-41 |
| 8906 | 6.92495132 | 22.63179532 | 1.7084752 | Up | 2.90E-74 |
| 136853 | 0.27834137 | 0.909152646 | 1.707667194 | Up | 0.00038273 |
| 79924 | 0.29431027 | 0.961312228 | 1.707667194 | Up | 6.18E-06 |
| 9420 | 0.39132821 | 1.278204088 | 1.707667194 | Up | 9.57E-05 |
| 124599 | 0.40855757 | 1.334480728 | 1.707667194 | Up | 9.57E-05 |
| 63910 | 2.3201772 | 7.564386526 | 1.704988093 | Up | 1.92E-23 |
| 249 | 1.41402991 | 4.604639021 | 1.703275424 | Up | 7.61E-15 |
| 2582 | 4.21399618 | 13.71958087 | 1.702975498 | Up | 1.70E-26 |
| 133 | 18.2185471 | 59.20583452 | 1.70033144 | Up | 1.14E-96 |
| 10116 | 1.58707878 | 5.155427912 | 1.69971844 | Up | 3.42E-16 |
| 5696 | 17.8604782 | 58.01576989 | 1.699674403 | Up | 2.67E-103 |
| 10509 | 18.6553377 | 60.58079986 | 1.699272153 | Up | 7.14E-257 |
| 3613 | 32.5948135 | 105.5419202 | 1.695101812 | Up | 4.98E-176 |
| 1048 | 0.32542745 | 1.052827627 | 1.693861394 | Up | 1.51E-05 |
| 6283 | 6.87168695 | 22.21052669 | 1.692507386 | Up | 6.13E-13 |
| 6850 | 1.89741623 | 6.132790106 | 1.692507386 | Up | 7.44E-36 |
| 55009 | 14.3115357 | 46.25745446 | 1.692507386 | Up | 2.94E-47 |
| 100125556 | 0.49795667 | 1.608613531 | 1.69172565 | Up | 5.93E-05 |
| 54436 | 1.66838862 | 5.389610548 | 1.69172565 | Up | 6.57E-27 |
| 7076 | 391.621384 | 1264.56021 | 1.691104281 | Up | 0 |
| 5982 | 11.8029885 | 38.07906659 | 1.689845916 | Up | 1.63E-72 |
| 84189 | 0.6164519 | 1.987380908 | 1.688808166 | Up | 1.25E-10 |
| 10009 | 2.3540231 | 7.584052989 | 1.687840564 | Up | 5.30E-45 |
| 10234 | 4.58009005 | 14.75115631 | 1.687380179 | Up | 2.38E-38 |
| 8942 | 0.93424149 | 3.007940541 | 1.686908633 | Up | 6.22E-07 |
| 2650 | 0.26147502 | 0.841860828 | 1.686908633 | Up | 6.22E-07 |
| 10636 | 5.0964698 | 16.38921195 | 1.685176306 | Up | 7.95E-45 |
| 10859 | 1.42100396 | 4.56778314 | 1.68458358 | Up | 2.07E-16 |
| 3693 | 20.475293 | 65.79812405 | 1.684162356 | Up | 1.13E-243 |
| 83605 | 13.8971272 | 44.65677311 | 1.684092325 | Up | 1.45E-105 |
| 9242 | 6.86908089 | 22.06475481 | 1.683554737 | Up | 2.68E-51 |
| 56005 | 25.414924 | 81.62631229 | 1.683358362 | Up | 2.47E-95 |
| 5210 | 0.87277964 | 2.798567594 | 1.680999243 | Up | 3.49E-12 |
| 84230 | 1.17056335 | 3.752632897 | 1.680700146 | Up | 3.86E-31 |
| 353514 | 1.31601431 | 4.21843022 | 1.680531064 | Up | 1.01E-07 |
| 84868 | 3.80413468 | 12.18990077 | 1.680046151 | Up | 1.78E-32 |
| 8111 | 7.35507672 | 23.54486582 | 1.678600206 | Up | 9.49E-74 |
| 113230 | 2.10438074 | 6.733308679 | 1.67791985 | Up | 4.99E-11 |
| 219623 | 0.21211178 | 0.678686167 | 1.67791985 | Up | 3.68E-05 |
| 23046 | 0.2999392 | 0.95759966 | 1.674752571 | Up | 8.31E-12 |
| 64064 | 0.81356863 | 2.597436579 | 1.674752571 | Up | 1.51E-06 |
| 1263 | 7.61814898 | 24.29602215 | 1.673207723 | Up | 1.00E-63 |
| 149111 | 0.39512271 | 1.259869614 | 1.672901775 | Up | 0.00014586 |
| 55911 | 1.25533225 | 4.002693175 | 1.672901775 | Up | 8.87E-18 |
| 79778 | 5.69576797 | 18.13381711 | 1.670720359 | Up | 2.05E-62 |
| 91543 | 2.8243248 | 8.986484275 | 1.669850799 | Up | 9.23E-36 |
| 26220 | 0.31661267 | 1.007297767 | 1.669699343 | Up | 2.44E-07 |
| 10288 | 1.92318275 | 6.10724223 | 1.667025209 | Up | 7.32E-21 |
| 26958 | 3.46622438 | 11.00672819 | 1.666948732 | Up | 3.33E-22 |
| 1509 | 131.263925 | 416.5277948 | 1.665942288 | Up | 0 |
| 23308 | 1.08542819 | 3.444062436 | 1.665847018 | Up | 1.47E-13 |
| 221692 | 0.54490178 | 1.728972739 | 1.665847018 | Up | 2.29E-05 |
| 83448 | 0.62668285 | 1.988463986 | 1.665847018 | Up | 7.50E-11 |
| 4602 | 0.2123507 | 0.673788543 | 1.665847018 | Up | 0.00058487 |
| 968 | 31.0137972 | 98.37532676 | 1.66538635 | Up | 9.58E-198 |
| 92714 | 6.89140484 | 21.84817511 | 1.664642765 | Up | 3.21E-39 |
| 121053 | 8.53854662 | 27.0693623 | 1.664598472 | Up | 1.02E-19 |
| 4500 | 12.100401 | 38.32611945 | 1.663273074 | Up | 3.81E-19 |
| 404636 | 4.51004863 | 14.26361337 | 1.661124608 | Up | 4.87E-35 |
| 2730 | 5.259348 | 16.62151418 | 1.660095948 | Up | 2.09E-56 |
| 7984 | 1.8785608 | 5.936764888 | 1.660049173 | Up | 1.43E-36 |
| 6664 | 0.49267604 | 1.554900833 | 1.658111369 | Up | 4.43E-16 |
| 1050 | 2.92620744 | 9.234177596 | 1.657951434 | Up | 3.26E-27 |
| 114822 | 1.05117885 | 3.315769376 | 1.657335518 | Up | 9.86E-15 |
| 9991 | 3.33891806 | 10.52782808 | 1.65675524 | Up | 6.51E-83 |
| 5034 | 114.609035 | 361.160652 | 1.655919946 | Up | 0 |
| 24145 | 3.92205916 | 12.34989276 | 1.654815311 | Up | 3.97E-38 |
| 9986 | 9.55708082 | 30.07685679 | 1.654011881 | Up | 7.97E-49 |
| 9902 | 15.3933386 | 48.43364496 | 1.653703414 | Up | 0 |
| 57224 | 1.01147544 | 3.182162581 | 1.653546255 | Up | 4.88E-27 |
| 84304 | 15.2991653 | 48.11394726 | 1.653002218 | Up | 6.07E-59 |
| 3714 | 4.18434193 | 13.15057492 | 1.652053218 | Up | 1.12E-72 |
| 157378 | 2.53874213 | 7.970810146 | 1.650612504 | Up | 3.76E-38 |
| 284996 | 9.10984512 | 28.59637844 | 1.650334018 | Up | 3.96E-76 |
| 84295 | 1.67292398 | 5.250022426 | 1.649951696 | Up | 3.34E-28 |
| 7873 | 26.7924736 | 83.97460083 | 1.648125248 | Up | 3.67E-84 |
| 440836 | 6.52196871 | 20.43871554 | 1.64792511 | Up | 1.20E-22 |
| 54935 | 30.8989802 | 96.82940215 | 1.647885965 | Up | 5.82E-72 |
| 9692 | 4.27918395 | 13.40470188 | 1.64733153 | Up | 9.62E-39 |
| 6536 | 3.00839599 | 9.416058362 | 1.646128783 | Up | 2.63E-35 |
| 25796 | 18.8836045 | 59.00126849 | 1.643611799 | Up | 4.31E-66 |
| 3099 | 2.31235634 | 7.224067491 | 1.643447633 | Up | 8.82E-56 |
| 51690 | 49.1145122 | 153.2051089 | 1.641243132 | Up | 1.38E-85 |
| 5721 | 89.5965759 | 278.9792942 | 1.638642547 | Up | 2.81E-244 |
| 6423 | 76.3756968 | 237.7469544 | 1.638241318 | Up | 0 |
| 79989 | 0.43571876 | 1.355426619 | 1.637277866 | Up | 0.00022132 |
| 57471 | 0.24827313 | 0.772323883 | 1.637277866 | Up | 0.00022132 |
| 940 | 0.24618625 | 0.765832061 | 1.637277866 | Up | 0.00022132 |
| 55964 | 0.301943 | 0.939279207 | 1.637277866 | Up | 5.50E-06 |
| 11182 | 1.74925247 | 5.441545236 | 1.637277866 | Up | 3.95E-16 |
| 79781 | 1.71492509 | 5.32713911 | 1.635215399 | Up | 2.04E-19 |
| 85014 | 11.6516682 | 36.18917985 | 1.635021892 | Up | 3.23E-34 |
| 6227 | 404.121725 | 1254.558235 | 1.634317623 | Up | 0 |
| 26873 | 3.36867309 | 10.44892501 | 1.63310219 | Up | 1.55E-45 |
| 1803 | 1.24163089 | 3.849985049 | 1.632616488 | Up | 2.66E-17 |
| 57650 | 1.0804344 | 3.349603992 | 1.632379066 | Up | 1.58E-16 |
| 118987 | 0.95669055 | 2.96339285 | 1.631125639 | Up | 1.96E-13 |
| 10652 | 24.3117601 | 75.25969551 | 1.630223108 | Up | 1.79E-218 |
| 91875 | 0.7956832 | 2.462170609 | 1.629664682 | Up | 3.42E-06 |
| 11314 | 2.10864584 | 6.520954595 | 1.628766366 | Up | 2.08E-14 |
| 2178 | 2.44548221 | 7.559819025 | 1.628232726 | Up | 9.40E-22 |
| 23201 | 0.70721076 | 2.186229497 | 1.628232726 | Up | 2.14E-05 |
| 160897 | 0.63724702 | 1.969947742 | 1.628232726 | Up | 1.53E-09 |
| 112950 | 15.1267958 | 46.76208164 | 1.628232726 | Up | 4.20E-74 |
| 57707 | 3.19927086 | 9.884601575 | 1.627439673 | Up | 1.95E-53 |
| 4481 | 2.40890727 | 7.441908407 | 1.627293777 | Up | 8.39E-31 |
| 160418 | 2.83043259 | 8.734018515 | 1.625623024 | Up | 4.71E-67 |
| 54732 | 32.9679267 | 101.718881 | 1.62545243 | Up | 1.01E-148 |
| 2937 | 13.7296129 | 42.35180282 | 1.625132436 | Up | 3.04E-85 |
| 54868 | 1.51304235 | 4.665818531 | 1.624677829 | Up | 1.17E-24 |
| 5074 | 3.56285513 | 10.98364507 | 1.624251185 | Up | 6.88E-24 |
| 2355 | 10.5871962 | 32.63617508 | 1.624151407 | Up | 1.04E-136 |
| 90990 | 5.98754448 | 18.44642568 | 1.623304923 | Up | 7.98E-64 |
| 8061 | 17.7463315 | 54.66604984 | 1.623124307 | Up | 3.50E-96 |
| 55233 | 5.28911433 | 16.28875526 | 1.622778296 | Up | 4.35E-24 |
| 169834 | 0.65660015 | 2.022115337 | 1.622778296 | Up | 2.13E-06 |
| 4854 | 9.90918726 | 30.51527401 | 1.622692907 | Up | 2.07E-256 |
| 27235 | 4.26568102 | 13.1274346 | 1.621737015 | Up | 1.50E-22 |
| 55785 | 1.76588539 | 5.430506243 | 1.620694981 | Up | 6.96E-54 |
| 27230 | 22.1602552 | 68.10852111 | 1.619860813 | Up | 1.31E-224 |
| 4067 | 4.31938635 | 13.27166281 | 1.619450869 | Up | 4.16E-44 |
| 11068 | 2.47324863 | 7.592506922 | 1.618169043 | Up | 6.28E-11 |
| 57489 | 2.38221402 | 7.304686398 | 1.616519305 | Up | 6.75E-19 |
| 441951 | 48.203053 | 147.7919601 | 1.616371359 | Up | 2.91E-106 |
| 2702 | 2.85171553 | 8.741061794 | 1.615978464 | Up | 2.76E-30 |
| 4860 | 20.3587413 | 62.39218488 | 1.615716961 | Up | 4.89E-98 |
| 115825 | 1.23028493 | 3.769742476 | 1.615473495 | Up | 2.21E-16 |
| 8507 | 1.40151463 | 4.293750516 | 1.615251559 | Up | 4.75E-26 |
| 28978 | 8.47266736 | 25.94930176 | 1.614807585 | Up | 9.45E-29 |
| 60681 | 22.5124006 | 68.94407118 | 1.61470659 | Up | 7.10E-206 |
| 1503 | 8.10567655 | 24.81247401 | 1.614061079 | Up | 2.63E-83 |
| 375757 | 8.27998897 | 25.33621882 | 1.613500482 | Up | 3.24E-27 |
| 64747 | 11.8183926 | 36.12360906 | 1.611908211 | Up | 8.35E-82 |
| 29775 | 2.34367859 | 7.159817761 | 1.611148134 | Up | 2.59E-30 |
| 27000 | 20.9379904 | 63.95828978 | 1.61100838 | Up | 1.26E-146 |
| 4488 | 1.9454902 | 5.941961939 | 1.610805654 | Up | 4.90E-15 |
| 29095 | 12.1193671 | 36.99042053 | 1.609837338 | Up | 2.91E-52 |
| 80194 | 23.7187903 | 72.33468634 | 1.608657188 | Up | 5.95E-70 |
| 54874 | 1.68480159 | 5.138103298 | 1.608657188 | Up | 5.62E-29 |
| 54407 | 16.2827022 | 49.64687877 | 1.608362879 | Up | 3.03E-249 |
| 55379 | 18.6142661 | 56.75372077 | 1.608306244 | Up | 1.10E-165 |
| 400506 | 19.585726 | 59.6934575 | 1.607770214 | Up | 1.24E-120 |
| 64167 | 5.76504166 | 17.56230916 | 1.60707961 | Up | 9.16E-104 |
| 203054 | 3.02692336 | 9.217868679 | 1.606581068 | Up | 4.28E-20 |
| 64866 | 3.62753822 | 11.04180797 | 1.605913695 | Up | 2.06E-69 |
| 1520 | 5.7733799 | 17.56853126 | 1.60550552 | Up | 3.50E-75 |
| 3107 | 224.218447 | 681.706552 | 1.604245874 | Up | 0 |
| 94081 | 4.28245552 | 13.01311517 | 1.603456199 | Up | 2.37E-41 |
| 10519 | 62.833812 | 190.8369232 | 1.602727318 | Up | 3.37E-191 |
| 7738 | 0.60447021 | 1.833366393 | 1.60075199 | Up | 3.23E-07 |
| 387628 | 0.25954889 | 0.78721534 | 1.60075199 | Up | 0.00033416 |
| 132014 | 0.69066401 | 2.094793362 | 1.60075199 | Up | 3.23E-07 |
| 114769 | 8.65516809 | 26.25124293 | 1.60075199 | Up | 7.32E-22 |
| 84955 | 1.34353048 | 4.07494629 | 1.60075199 | Up | 5.55E-18 |
| 727936 | 0.80935325 | 2.45477944 | 1.60075199 | Up | 3.22E-05 |
| 4124 | 0.5483021 | 1.663007756 | 1.60075199 | Up | 3.53E-10 |
| 26636 | 1.40937001 | 4.274638484 | 1.60075199 | Up | 0.00033416 |
| 5631 | 9.49113085 | 28.76982586 | 1.599904592 | Up | 1.10E-64 |
| 79081 | 19.1706353 | 58.08958023 | 1.599381258 | Up | 1.66E-79 |
| 3671 | 60.6443626 | 183.5064261 | 1.597385138 | Up | 0 |
| 79728 | 1.28952031 | 3.897661513 | 1.595774313 | Up | 1.31E-17 |
| 10212 | 14.2622118 | 43.0990306 | 1.59545769 | Up | 7.29E-67 |
| 1969 | 2.41472777 | 7.296195197 | 1.595283777 | Up | 8.55E-31 |
| 10134 | 62.0694207 | 187.5353388 | 1.595207894 | Up | 0 |
| 83861 | 1.36392388 | 4.12005155 | 1.594899263 | Up | 1.39E-10 |
| 128439 | 5.31701153 | 16.02598701 | 1.591725714 | Up | 3.51E-19 |
| 4017 | 12.176624 | 36.69273267 | 1.591380156 | Up | 2.11E-142 |
| 64928 | 32.3495882 | 97.43488625 | 1.590691072 | Up | 1.00E-77 |
| 284021 | 1.18664153 | 3.573931435 | 1.590627772 | Up | 1.24E-06 |
| 81553 | 1.62495318 | 4.89181654 | 1.589972151 | Up | 8.08E-09 |
| 4060 | 124.905084 | 375.7386715 | 1.588897403 | Up | 0 |
| 23658 | 7.24475126 | 21.79102687 | 1.588726121 | Up | 2.41E-67 |
| 145864 | 8.4167607 | 25.29712607 | 1.58763649 | Up | 8.53E-50 |
| 147495 | 7.77367681 | 23.35718289 | 1.587197246 | Up | 9.01E-62 |
| 64946 | 1.79933969 | 5.404946612 | 1.586812799 | Up | 5.06E-09 |
| 8566 | 5.72463084 | 17.1925381 | 1.586527975 | Up | 7.08E-129 |
| 6801 | 0.83063977 | 2.493153178 | 1.585676669 | Up | 3.30E-10 |
| 55161 | 3.04637541 | 9.14288562 | 1.585555832 | Up | 2.07E-72 |
| 1075 | 8.93692207 | 26.81472958 | 1.585175754 | Up | 2.69E-166 |
| 6404 | 3.51369181 | 10.52763706 | 1.583122096 | Up | 2.37E-28 |
| 25804 | 30.7780034 | 92.20626366 | 1.582965113 | Up | 2.22E-107 |
| 391356 | 15.8389864 | 47.40272853 | 1.581490087 | Up | 9.46E-29 |
| 200916 | 5.41383894 | 16.1973481 | 1.581033755 | Up | 2.07E-33 |
| 81037 | 18.6253028 | 55.70997534 | 1.580671795 | Up | 6.07E-121 |
| 9235 | 84.9880368 | 254.0687744 | 1.579887394 | Up | 3.84E-294 |
| 23094 | 0.77290548 | 2.309993838 | 1.579525098 | Up | 5.19E-20 |
| 63027 | 2.19989381 | 6.57440391 | 1.57942621 | Up | 5.99E-42 |
| 11044 | 8.53646522 | 25.50421595 | 1.579025042 | Up | 9.40E-100 |
| 378708 | 2.65835903 | 7.938795529 | 1.578384177 | Up | 3.56E-13 |
| 8767 | 4.56227885 | 13.61893079 | 1.577786911 | Up | 9.89E-37 |
| 11135 | 21.2054668 | 63.27978757 | 1.577308513 | Up | 1.21E-137 |
| 127687 | 23.2292979 | 69.29343495 | 1.576773127 | Up | 1.83E-92 |
| 51388 | 9.54825598 | 28.46772925 | 1.576018271 | Up | 1.78E-63 |
| 1192 | 153.95762 | 458.9442841 | 1.575785747 | Up | 0 |
| 121457 | 4.73106027 | 14.09368841 | 1.574813779 | Up | 1.42E-42 |
| 7105 | 3.96364029 | 11.80159607 | 1.574084039 | Up | 7.00E-26 |
| 153562 | 0.68769575 | 2.047589288 | 1.574084039 | Up | 2.97E-06 |
| 201176 | 2.31098069 | 6.869450646 | 1.571689524 | Up | 1.77E-26 |
| 401237 | 1.11752511 | 3.321872663 | 1.571689524 | Up | 1.17E-07 |
| 5880 | 13.2476461 | 39.37345824 | 1.571487395 | Up | 6.80E-59 |
| 55270 | 2.93560668 | 8.723497567 | 1.571247995 | Up | 4.51E-19 |
| 7328 | 5.49695636 | 16.32500315 | 1.570378341 | Up | 1.00E-45 |
| 51678 | 1.39277592 | 4.132656717 | 1.569106363 | Up | 1.92E-10 |
| 388327 | 0.85166389 | 2.52466683 | 1.567736932 | Up | 2.98E-05 |
| 647087 | 11.698185 | 34.67802281 | 1.567736932 | Up | 4.78E-87 |
| 115201 | 4.49931357 | 13.33396466 | 1.567328988 | Up | 1.43E-31 |
| 132299 | 19.8323326 | 58.76838866 | 1.56718597 | Up | 5.92E-46 |
| 6238 | 28.691167 | 84.99763445 | 1.566816039 | Up | 0 |
| 84941 | 1.52040586 | 4.503452265 | 1.566574874 | Up | 4.99E-12 |
| 55752 | 11.7821726 | 34.87605412 | 1.565631227 | Up | 4.93E-192 |
| 4493 | 98.0141176 | 290.1117902 | 1.565547458 | Up | 5.68E-146 |
| 27346 | 4.28834304 | 12.68729194 | 1.56489194 | Up | 9.91E-34 |
| 8771 | 6.97344538 | 20.6249199 | 1.564444986 | Up | 3.34E-31 |
| 55959 | 31.5690735 | 93.32011528 | 1.563676168 | Up | 0 |
| 80339 | 0.33412872 | 0.987431916 | 1.563277284 | Up | 0.00050198 |
| 8780 | 7.30093622 | 21.56098767 | 1.562269886 | Up | 5.68E-87 |
| 57419 | 3.59484785 | 10.60803436 | 1.561154737 | Up | 3.93E-42 |
| 1962 | 1.02739527 | 3.02981176 | 1.56023683 | Up | 7.79E-13 |
| 55191 | 11.249344 | 33.17456852 | 1.56023683 | Up | 1.30E-80 |
| 83442 | 148.079705 | 436.4132122 | 1.559320849 | Up | 0 |
| 8630 | 0.98144787 | 2.892383589 | 1.559275354 | Up | 1.14E-05 |
| 9158 | 27.2551101 | 80.27740836 | 1.558467294 | Up | 2.16E-115 |
| 54512 | 16.2313759 | 47.80479889 | 1.558370153 | Up | 1.59E-43 |
| 11014 | 34.9003873 | 102.7823678 | 1.558277841 | Up | 2.39E-288 |
| 11257 | 12.7784206 | 37.62627972 | 1.558031115 | Up | 9.71E-28 |
| 23271 | 4.50489694 | 13.24696732 | 1.556096109 | Up | 1.25E-96 |
| 55003 | 12.5382081 | 36.85844698 | 1.5556641 | Up | 1.98E-55 |
| 84727 | 1.70888969 | 5.020652876 | 1.554815705 | Up | 1.73E-07 |
| 578 | 13.4978937 | 39.65137404 | 1.554636561 | Up | 2.29E-86 |
| 3730 | 0.50715966 | 1.489160686 | 1.553987522 | Up | 1.12E-10 |
| 3145 | 7.09082478 | 20.81826152 | 1.553824248 | Up | 2.91E-32 |
| 79616 | 0.52481886 | 1.536126019 | 1.54940511 | Up | 2.74E-06 |
| 65244 | 4.52649352 | 13.24888458 | 1.54940511 | Up | 1.87E-50 |
| 920 | 5.990466 | 17.52320257 | 1.548526328 | Up | 2.29E-54 |
| 1404 | 0.51756739 | 1.511360078 | 1.546028803 | Up | 2.63E-08 |
| 5784 | 0.74492564 | 2.174698975 | 1.54564739 | Up | 4.20E-10 |
| 54888 | 12.1907508 | 35.58540417 | 1.545498636 | Up | 1.21E-106 |
| 84856 | 0.54214378 | 1.58108604 | 1.544168461 | Up | 7.19E-05 |
| 57491 | 0.2207847 | 0.643887513 | 1.544168461 | Up | 7.19E-05 |
| 84910 | 2.53248991 | 7.383902357 | 1.543826955 | Up | 8.33E-39 |
| 5820 | 3.98524516 | 11.61761396 | 1.543573413 | Up | 6.13E-23 |
| 2219 | 2.5567488 | 7.442195406 | 1.54141785 | Up | 1.03E-10 |
| 9610 | 3.20092575 | 9.314682114 | 1.541017324 | Up | 1.05E-25 |
| 64333 | 2.55630343 | 7.437557801 | 1.540769885 | Up | 3.34E-20 |
| 26508 | 2.12627367 | 6.183260863 | 1.540040576 | Up | 4.18E-26 |
| 22995 | 1.07262727 | 3.117441335 | 1.539213579 | Up | 7.35E-17 |
| 84154 | 24.6076648 | 71.50557415 | 1.538947955 | Up | 8.01E-67 |
| 3059 | 5.53210659 | 16.06190545 | 1.537742192 | Up | 5.43E-33 |
| 4940 | 3.24052682 | 9.402428608 | 1.536805073 | Up | 2.17E-61 |
| 402665 | 0.70544273 | 2.045568317 | 1.535900845 | Up | 1.60E-07 |
| 222229 | 3.6599484 | 10.61154744 | 1.535739839 | Up | 5.67E-24 |
| 54843 | 1.8704285 | 5.418792112 | 1.534602485 | Up | 1.54E-26 |
| 4818 | 4.63320439 | 13.4128161 | 1.533529941 | Up | 3.96E-12 |
| 22824 | 0.47769167 | 1.381975572 | 1.532580487 | Up | 1.05E-05 |
| 27075 | 2.95656485 | 8.545761734 | 1.53128717 | Up | 4.28E-17 |
| 23400 | 4.0458548 | 11.69200465 | 1.531005861 | Up | 3.31E-46 |
| 78996 | 8.76661704 | 25.32684157 | 1.530575041 | Up | 1.32E-46 |
| 140 | 0.730581 | 2.110344908 | 1.530362662 | Up | 6.53E-06 |
| 54757 | 2.13804465 | 6.173486209 | 1.529793439 | Up | 9.01E-27 |
| 672 | 1.0314828 | 2.978092686 | 1.529668892 | Up | 3.07E-22 |
| 26272 | 4.34154515 | 12.53489041 | 1.529668892 | Up | 3.07E-22 |
| 51192 | 16.3744491 | 47.27280328 | 1.529564048 | Up | 1.70E-31 |
| 55039 | 5.03319969 | 14.51848823 | 1.528343494 | Up | 2.70E-32 |
| 25824 | 103.106019 | 297.2414927 | 1.52750697 | Up | 1.32E-250 |
| 64098 | 3.25427451 | 9.375095504 | 1.526497435 | Up | 2.84E-21 |
| 84261 | 2.06834718 | 5.958609923 | 1.526497435 | Up | 2.36E-11 |
| 5450 | 2.13803259 | 6.154137537 | 1.52527284 | Up | 2.65E-19 |
| 8510 | 1.511663 | 4.349771708 | 1.524803136 | Up | 1.57E-06 |
| 654433 | 2.44799157 | 7.044033288 | 1.524803136 | Up | 1.67E-19 |
| 57085 | 17.2069765 | 49.48416386 | 1.523973286 | Up | 1.26E-61 |
| 7355 | 2.56465162 | 7.37508118 | 1.523896066 | Up | 6.63E-20 |
| 6158 | 98.3961227 | 282.8865557 | 1.523550241 | Up | 0 |
| 435 | 8.30313923 | 23.8478431 | 1.522129993 | Up | 4.22E-48 |
| 5790 | 4.56387781 | 13.09895792 | 1.521119971 | Up | 9.05E-13 |
| 58477 | 11.0104508 | 31.57786719 | 1.52004019 | Up | 1.22E-53 |
| 3119 | 8.80568397 | 25.24880733 | 1.519708269 | Up | 8.41E-31 |
| 9111 | 8.87169359 | 25.42950797 | 1.519222104 | Up | 4.07E-37 |
| 6185 | 50.9771384 | 146.0671032 | 1.518709 | Up | 0 |
| 84918 | 5.24569622 | 15.02888269 | 1.518531588 | Up | 7.04E-53 |
| 55676 | 0.92447962 | 2.647770577 | 1.518064707 | Up | 1.47E-07 |
| 8840 | 1.22820367 | 3.516758031 | 1.51769625 | Up | 8.79E-11 |
| 6553 | 0.31366501 | 0.897684353 | 1.516983632 | Up | 0.00017331 |
| 581 | 14.7249643 | 42.10953343 | 1.515882758 | Up | 1.50E-37 |
| 57698 | 4.08042941 | 11.66430888 | 1.515307939 | Up | 7.44E-45 |
| 6591 | 6.59549528 | 18.84796375 | 1.514855763 | Up | 9.54E-39 |
| 9937 | 0.8740218 | 2.495943189 | 1.513843924 | Up | 5.36E-12 |
| 3561 | 6.29772765 | 17.98340992 | 1.513763329 | Up | 4.12E-26 |
| 65260 | 3.29056753 | 9.393257635 | 1.513289148 | Up | 8.58E-16 |
| 10897 | 30.1680827 | 86.02222294 | 1.511686403 | Up | 8.33E-92 |
| 387521 | 20.250025 | 57.69672288 | 1.510565691 | Up | 8.13E-131 |
| 163786 | 1.28267017 | 3.647208868 | 1.507642585 | Up | 8.01E-15 |
| 6634 | 53.4593889 | 151.5650768 | 1.503422119 | Up | 5.09E-196 |
| 56606 | 1.1288918 | 3.199583217 | 1.502976774 | Up | 5.56E-07 |
| 4439 | 2.30031878 | 6.515980993 | 1.502148591 | Up | 4.81E-19 |
| 6648 | 37.1636923 | 105.2370074 | 1.501676379 | Up | 1.12E-156 |
| 7184 | 104.932794 | 297.0844176 | 1.501407313 | Up | 0 |
| 55152 | 3.17008721 | 8.972839581 | 1.501042088 | Up | 3.01E-20 |
| 8321 | 3.82433363 | 10.82429677 | 1.500993006 | Up | 2.88E-45 |
| 1318 | 2.77365449 | 7.847587763 | 1.500461176 | Up | 1.86E-14 |
| 5777 | 6.47313205 | 18.30591874 | 1.499774342 | Up | 4.09E-42 |
| 4332 | 3.27965277 | 9.269508151 | 1.498949709 | Up | 7.25E-16 |
| 4261 | 1.20829101 | 3.414181024 | 1.498571595 | Up | 2.87E-16 |
| 1445 | 8.57988287 | 24.23395202 | 1.497999838 | Up | 5.98E-63 |
| 3251 | 11.646393 | 32.86401865 | 1.496625697 | Up | 1.53E-44 |
| 2166 | 4.84646132 | 13.67303585 | 1.496329961 | Up | 4.69E-28 |
| 123920 | 15.6572718 | 44.14444307 | 1.495398989 | Up | 5.72E-88 |
| 514 | 306.183495 | 863.0345545 | 1.495021808 | Up | 0 |
| 7979 | 77.4752582 | 218.3714762 | 1.49497686 | Up | 1.21E-101 |
| 29062 | 4.21033654 | 11.86660012 | 1.494899191 | Up | 4.12E-52 |
| 3695 | 1.40928524 | 3.971886696 | 1.494860821 | Up | 1.15E-11 |
| 86 | 8.27739917 | 23.32724057 | 1.494766221 | Up | 1.38E-42 |
| 8711 | 3.41612732 | 9.615568555 | 1.493010421 | Up | 1.08E-26 |
| 80790 | 14.6274299 | 41.16996363 | 1.492915872 | Up | 4.42E-166 |
| 811 | 260.709791 | 733.4576232 | 1.492268851 | Up | 0 |
| 51278 | 9.23758195 | 25.9869921 | 1.492202493 | Up | 1.59E-57 |
| 58191 | 9.71241308 | 27.31734332 | 1.491915498 | Up | 6.52E-60 |
| 222183 | 1.64199079 | 4.617252342 | 1.491588546 | Up | 6.57E-17 |
| 57609 | 1.70275293 | 4.783932761 | 1.490328 | Up | 7.20E-40 |
| 51614 | 76.7303612 | 215.3559562 | 1.488853774 | Up | 5.43E-270 |
| 1947 | 8.18552651 | 22.96083535 | 1.488028008 | Up | 1.59E-71 |
| 6628 | 53.9199135 | 151.2127103 | 1.487689325 | Up | 1.18E-160 |
| 3927 | 20.2861396 | 56.88391944 | 1.487526521 | Up | 1.31E-214 |
| 5373 | 9.61572976 | 26.95258247 | 1.486955256 | Up | 7.90E-59 |
| 51121 | 15.4005104 | 43.11688438 | 1.485274772 | Up | 1.45E-29 |
| 9535 | 14.4061176 | 40.33287824 | 1.485274772 | Up | 9.11E-26 |
| 348793 | 4.27550769 | 11.97015986 | 1.485274772 | Up | 9.37E-19 |
| 254042 | 0.71280067 | 1.995631546 | 1.485274772 | Up | 0.00041756 |
| 27132 | 0.41168533 | 1.152597436 | 1.485274772 | Up | 0.00041756 |
| 144577 | 0.54071023 | 1.513829072 | 1.485274772 | Up | 6.00E-05 |
| 282997 | 0.54264826 | 1.519254983 | 1.485274772 | Up | 0.00041756 |
| 254394 | 0.47507657 | 1.330074205 | 1.485274772 | Up | 0.00025642 |
| 6772 | 18.129663 | 50.74078721 | 1.484793794 | Up | 2.42E-200 |
| 1508 | 114.341872 | 319.9202126 | 1.484358326 | Up | 0 |
| 7037 | 6.14474804 | 17.18952869 | 1.484104228 | Up | 2.29E-83 |
| 1072 | 361.123503 | 1009.415641 | 1.482956125 | Up | 0 |
| 124935 | 4.81472137 | 13.4555111 | 1.482672977 | Up | 2.19E-38 |
| 339230 | 6.70615202 | 18.74066984 | 1.482615426 | Up | 1.38E-37 |
| 5877 | 3.72988894 | 10.41258092 | 1.481123129 | Up | 1.32E-24 |
| 57403 | 3.65640368 | 10.20314975 | 1.480517323 | Up | 1.09E-81 |
| 161291 | 1.60003051 | 4.463204429 | 1.479980473 | Up | 1.35E-19 |
| 3801 | 6.11937805 | 17.06804502 | 1.479840886 | Up | 3.36E-54 |
| 4688 | 5.50474295 | 15.35121798 | 1.479606022 | Up | 3.13E-35 |
| 26011 | 0.38624693 | 1.075997438 | 1.478079271 | Up | 9.01E-15 |
| 3002 | 5.41556408 | 15.08301267 | 1.477741099 | Up | 3.63E-14 |
| 113655 | 3.18675844 | 8.871576553 | 1.477100841 | Up | 3.70E-13 |
| 55088 | 3.59592926 | 10.00948117 | 1.476930648 | Up | 1.86E-58 |
| 285908 | 1.88943634 | 5.256905539 | 1.476257899 | Up | 1.22E-22 |
| 285753 | 1.29184157 | 3.591658685 | 1.475221108 | Up | 6.21E-11 |
| 28982 | 2.02597795 | 5.629652466 | 1.47442739 | Up | 4.95E-19 |
| 1734 | 5.75124361 | 15.97114167 | 1.473521591 | Up | 6.82E-98 |
| 1486 | 2.94869404 | 8.184913561 | 1.472891048 | Up | 1.76E-24 |
| 146223 | 1.05843103 | 2.936355517 | 1.472099383 | Up | 4.44E-23 |
| 4939 | 3.85480282 | 10.69238156 | 1.471854256 | Up | 9.64E-37 |
| 84319 | 23.3395047 | 64.73531111 | 1.47177893 | Up | 2.23E-71 |
| 954 | 1.22732639 | 3.401443101 | 1.470627996 | Up | 7.01E-08 |
| 51629 | 40.9917311 | 113.45186 | 1.46867544 | Up | 4.54E-165 |
| 64761 | 6.57207218 | 18.18837005 | 1.468596031 | Up | 1.84E-63 |
| 55646 | 22.6926422 | 62.78799594 | 1.468264169 | Up | 1.07E-89 |
| 1893 | 29.5572557 | 81.76524113 | 1.467975348 | Up | 2.55E-158 |
| 6996 | 10.620796 | 29.33053553 | 1.465511517 | Up | 1.35E-86 |
| 5476 | 36.249714 | 100.021813 | 1.464273144 | Up | 3.22E-205 |
| 283663 | 1.43022255 | 3.946169203 | 1.464213157 | Up | 2.30E-10 |
| 65065 | 0.38801242 | 1.070226633 | 1.463741611 | Up | 3.67E-10 |
| 5908 | 1.66806667 | 4.600907849 | 1.463741611 | Up | 3.67E-10 |
| 54939 | 27.7791908 | 76.59156821 | 1.463181001 | Up | 8.27E-65 |
| 55299 | 28.5068316 | 78.49484079 | 1.461290132 | Up | 1.45E-90 |
| 100128842 | 0.44414324 | 1.222745499 | 1.461027226 | Up | 3.37E-05 |
| 79713 | 3.85288688 | 10.60411669 | 1.460612718 | Up | 7.81E-13 |
| 22936 | 9.62395439 | 26.47406449 | 1.459877995 | Up | 1.28E-143 |
| 29889 | 24.657127 | 67.8183842 | 1.459671704 | Up | 4.87E-146 |
| 219541 | 6.80947268 | 18.72751509 | 1.459544497 | Up | 7.75E-27 |
| 445328 | 3.01334282 | 8.284462193 | 1.459043231 | Up | 1.08E-15 |
| 57180 | 1.31414087 | 3.612914617 | 1.459043231 | Up | 1.56E-08 |
| 90701 | 20.0006628 | 54.96936126 | 1.458579906 | Up | 9.11E-40 |
| 23590 | 1.73775845 | 4.775114366 | 1.458307725 | Up | 2.49E-08 |
| 10418 | 5.237792 | 14.3799232 | 1.457025295 | Up | 3.26E-70 |
| 23150 | 0.95011702 | 2.604629632 | 1.454901123 | Up | 1.11E-13 |
| 54994 | 7.72733988 | 21.1732671 | 1.454200139 | Up | 2.11E-84 |
| 64854 | 0.5926431 | 1.622958564 | 1.453390711 | Up | 4.47E-13 |
| 90693 | 0.94746367 | 2.593671592 | 1.452853294 | Up | 4.21E-07 |
| 51602 | 41.8931949 | 114.6178755 | 1.452044243 | Up | 1.10E-199 |
| 30817 | 0.83051046 | 2.271360511 | 1.451486459 | Up | 3.93E-15 |
| 7083 | 12.4376535 | 33.9991984 | 1.450786396 | Up | 2.09E-49 |
| 84898 | 4.12492467 | 11.26758468 | 1.449738615 | Up | 2.35E-27 |
| 89857 | 0.50667818 | 1.383950629 | 1.449650862 | Up | 3.46E-09 |
| 1123 | 8.43807461 | 23.03284401 | 1.448706811 | Up | 1.57E-56 |
| 5531 | 35.5011765 | 96.87377127 | 1.448239272 | Up | 1.04E-114 |
| 388698 | 0.22261207 | 0.607267381 | 1.447800067 | Up | 2.80E-06 |
| 5805 | 9.70010732 | 26.4550905 | 1.447472739 | Up | 2.57E-23 |
| 752014 | 4.28887045 | 11.69158291 | 1.446800624 | Up | 8.93E-16 |
| 6895 | 4.67280442 | 12.73205007 | 1.446104175 | Up | 1.62E-22 |
| 5519 | 1.37951048 | 3.758189332 | 1.445881334 | Up | 4.51E-20 |
| 54704 | 2.84954372 | 7.760454987 | 1.445410311 | Up | 2.86E-31 |
| 168374 | 0.60194673 | 1.638460068 | 1.444632788 | Up | 7.26E-06 |
| 84816 | 1.3925727 | 3.788188583 | 1.443755503 | Up | 3.03E-10 |
| 80005 | 0.9510838 | 2.585572708 | 1.442839506 | Up | 1.14E-18 |
| 3751 | 0.33683846 | 0.915713567 | 1.442839506 | Up | 1.17E-05 |
| 54965 | 12.0058949 | 32.62928584 | 1.442424468 | Up | 4.24E-88 |
| 83985 | 10.9809697 | 29.82084093 | 1.441315483 | Up | 3.70E-59 |
| 51025 | 23.690609 | 64.32978076 | 1.441171489 | Up | 2.59E-30 |
| 440278 | 1.04835689 | 2.846147805 | 1.440880653 | Up | 1.89E-05 |
| 56926 | 10.5029465 | 28.48746021 | 1.439532885 | Up | 4.23E-94 |
| 123169 | 6.50854902 | 17.64515575 | 1.438864307 | Up | 1.26E-34 |
| 527 | 85.962867 | 233.0017422 | 1.438555237 | Up | 1.99E-222 |
| 10561 | 11.267407 | 30.53190733 | 1.438162176 | Up | 1.86E-47 |
| 199 | 13.5853394 | 36.81116586 | 1.438092835 | Up | 2.73E-49 |
| 57221 | 0.27367246 | 0.741485925 | 1.437969057 | Up | 3.82E-07 |
| 9662 | 0.84025712 | 2.275341757 | 1.437180484 | Up | 9.37E-13 |
| 347240 | 0.25085142 | 0.678899607 | 1.436365172 | Up | 4.92E-05 |
| 10769 | 7.82428083 | 21.17287101 | 1.436186852 | Up | 8.56E-53 |
| 729440 | 3.33808322 | 9.02613864 | 1.435089015 | Up | 7.38E-16 |
| 6356 | 17.224787 | 46.56956462 | 1.43490125 | Up | 5.67E-39 |
| 9466 | 2.08561239 | 5.637750996 | 1.434648699 | Up | 1.66E-17 |
| 6574 | 8.01479057 | 21.66222681 | 1.434444828 | Up | 3.21E-63 |
| 55505 | 105.401998 | 284.8765364 | 1.434434591 | Up | 2.43E-133 |
| 9551 | 189.655174 | 512.5724431 | 1.434377189 | Up | 1.19E-220 |
| 8985 | 18.9156755 | 51.12202981 | 1.434362819 | Up | 3.83E-133 |
| 567 | 1243.5483 | 3359.645081 | 1.433846287 | Up | 0 |
| 8407 | 240.510029 | 649.4541098 | 1.433130535 | Up | 0 |
| 57407 | 16.2857086 | 43.93619865 | 1.431803564 | Up | 2.58E-53 |
| 55341 | 10.4107622 | 28.08568452 | 1.431759263 | Up | 3.79E-88 |
| 27044 | 21.2399855 | 57.28403626 | 1.431350367 | Up | 1.25E-174 |
| 53340 | 3.00715848 | 8.107335734 | 1.430826988 | Up | 5.25E-08 |
| 6183 | 16.2441781 | 43.78698142 | 1.430579251 | Up | 2.53E-42 |
| 154214 | 1.92629702 | 5.188780515 | 1.42956534 | Up | 1.79E-09 |
| 4150 | 57.3614187 | 154.3527424 | 1.428078504 | Up | 0 |
| 84284 | 5.19529412 | 13.97964024 | 1.428049903 | Up | 2.15E-12 |
| 9332 | 4.62057297 | 12.43200561 | 1.427915393 | Up | 1.81E-46 |
| 26031 | 1.58371135 | 4.260045035 | 1.427559274 | Up | 3.34E-26 |
| 51478 | 1.7481817 | 4.7024559 | 1.427559274 | Up | 1.34E-07 |
| 5052 | 221.839199 | 596.5716946 | 1.427181215 | Up | 0 |
| 8833 | 17.0451903 | 45.8221395 | 1.426680118 | Up | 3.61E-98 |
| 116039 | 3.84453618 | 10.33458406 | 1.426598865 | Up | 3.42E-18 |
| 10954 | 6.62123434 | 17.79774279 | 1.426522185 | Up | 3.04E-27 |
| 2140 | 0.95479937 | 2.566230213 | 1.426381083 | Up | 6.56E-06 |
| 29926 | 14.6576679 | 39.39244978 | 1.426263557 | Up | 4.54E-63 |
| 3720 | 2.87660679 | 7.72441288 | 1.425057249 | Up | 8.90E-40 |
| 6274 | 6.03231372 | 16.1973481 | 1.424974262 | Up | 8.68E-12 |
| 2923 | 75.2383152 | 202.0054337 | 1.424854652 | Up | 0 |
| 650 | 2.00835227 | 5.391403011 | 1.424648413 | Up | 3.83E-16 |
| 339229 | 8.06220265 | 21.63129714 | 1.423874228 | Up | 4.85E-13 |
| 148709 | 0.53840647 | 1.444571761 | 1.423874228 | Up | 0.0003399 |
| 64386 | 0.70382507 | 1.88839823 | 1.423874228 | Up | 3.45E-07 |
| 400966 | 0.18458514 | 0.495251264 | 1.423874228 | Up | 0.0003399 |
| 114884 | 0.79635573 | 2.136662667 | 1.423874228 | Up | 1.16E-08 |
| 64834 | 39.8149405 | 106.8009227 | 1.423542306 | Up | 6.11E-137 |
| 10808 | 11.6291282 | 31.18663111 | 1.423184768 | Up | 2.28E-99 |
| 64785 | 2.80921062 | 7.529632963 | 1.422414748 | Up | 5.47E-17 |
| 92675 | 11.5411286 | 30.92544151 | 1.422009879 | Up | 8.72E-38 |
| 79572 | 3.53849062 | 9.479029384 | 1.421605244 | Up | 5.75E-61 |
| 196743 | 1.5999452 | 4.282910643 | 1.420569083 | Up | 2.97E-08 |
| 2687 | 8.06987095 | 21.60078862 | 1.420466477 | Up | 1.13E-47 |
| 55135 | 2.06999724 | 5.535332264 | 1.419041074 | Up | 8.90E-11 |
| 57622 | 0.54861333 | 1.466139268 | 1.418160576 | Up | 2.75E-05 |
| 115353 | 11.9386687 | 31.89384471 | 1.417636055 | Up | 3.74E-48 |
| 353500 | 1.11059493 | 2.966826736 | 1.417587965 | Up | 8.76E-16 |
| 5318 | 0.51024444 | 1.362854137 | 1.417370708 | Up | 1.43E-06 |
| 92799 | 14.1734392 | 37.83149642 | 1.416397968 | Up | 1.46E-77 |
| 2331 | 19.7741315 | 52.72545999 | 1.414885444 | Up | 6.61E-135 |
| 120425 | 3.16680198 | 8.434427735 | 1.413263527 | Up | 2.83E-17 |
| 92255 | 0.82035465 | 2.184165105 | 1.412762272 | Up | 1.95E-07 |
| 3309 | 42.3901059 | 112.7605043 | 1.41146236 | Up | 0 |
| 271 | 5.30289159 | 14.0995363 | 1.410796556 | Up | 4.00E-48 |
| 684 | 46.246048 | 122.8773261 | 1.409816739 | Up | 7.53E-99 |
| 3978 | 1.64666501 | 4.373756826 | 1.409325919 | Up | 6.32E-13 |
| 26090 | 18.3139843 | 48.6414767 | 1.409241336 | Up | 3.41E-82 |
| 91300 | 27.9770465 | 74.29828333 | 1.409085209 | Up | 9.49E-115 |
| 51367 | 11.9554777 | 31.73420208 | 1.408366786 | Up | 6.19E-23 |
| 23322 | 0.40428386 | 1.072923657 | 1.408106912 | Up | 4.99E-07 |
| 2683 | 14.0618139 | 37.30327573 | 1.407519615 | Up | 3.43E-133 |
| 55714 | 0.77307835 | 2.049766161 | 1.406772798 | Up | 2.99E-20 |
| 92667 | 5.06730474 | 13.43306161 | 1.406497659 | Up | 1.17E-25 |
| 6747 | 33.2649731 | 88.13651639 | 1.405736007 | Up | 9.27E-228 |
| 51477 | 17.1183755 | 45.33038653 | 1.404932664 | Up | 4.98E-71 |
| 51450 | 16.2887751 | 43.09806221 | 1.403744887 | Up | 6.20E-49 |
| 203547 | 5.83103794 | 15.42341338 | 1.40329747 | Up | 3.38E-62 |
| 22853 | 0.41825734 | 1.105941744 | 1.402812612 | Up | 1.28E-06 |
| 27085 | 0.45957718 | 1.215198231 | 1.402812612 | Up | 0.00018784 |
| 7737 | 10.7956473 | 28.53610015 | 1.402338431 | Up | 1.40E-32 |
| 56950 | 6.07207066 | 16.04835172 | 1.402164647 | Up | 2.91E-24 |
| 27301 | 4.89869547 | 12.94067341 | 1.401443181 | Up | 5.07E-23 |
| 90861 | 11.3877518 | 30.0814176 | 1.401389602 | Up | 3.76E-94 |
| 123355 | 4.41683602 | 11.66209063 | 1.400741262 | Up | 1.03E-15 |
| 4176 | 21.1146307 | 55.74680393 | 1.400646077 | Up | 2.08E-136 |
| 1794 | 1.1917212 | 3.145129036 | 1.40007245 | Up | 1.32E-17 |
| 4678 | 20.6630634 | 54.51125115 | 1.399499878 | Up | 3.39E-151 |
| 3669 | 10.2938558 | 27.14241805 | 1.398765779 | Up | 2.89E-23 |
| 2633 | 12.1635178 | 32.07074924 | 1.398697524 | Up | 4.19E-83 |
| 400759 | 1.07596758 | 2.835192212 | 1.397811931 | Up | 0.0003048 |
| 8372 | 0.68369928 | 1.8015588 | 1.397811931 | Up | 0.0003048 |
| 55331 | 4.30837163 | 11.34774319 | 1.397190802 | Up | 4.36E-43 |
| 124044 | 3.03376476 | 7.990318895 | 1.397143862 | Up | 5.27E-17 |
| 80336 | 2.67801265 | 7.052579469 | 1.39698824 | Up | 4.60E-14 |
| 51319 | 10.9861749 | 28.92241458 | 1.396498838 | Up | 8.17E-41 |
| 83982 | 66.2283189 | 174.338266 | 1.396369122 | Up | 2.64E-69 |
| 7319 | 19.6365462 | 51.66469534 | 1.39563756 | Up | 8.16E-115 |
| 23536 | 3.89510524 | 10.23693783 | 1.394050012 | Up | 8.59E-25 |
| 200558 | 0.54801712 | 1.439577196 | 1.393352283 | Up | 5.32E-06 |
| 112479 | 1.56249819 | 4.103729377 | 1.393081074 | Up | 1.84E-13 |
| 79070 | 2.59477037 | 6.814878543 | 1.393081074 | Up | 1.84E-13 |
| 22861 | 2.61594274 | 6.869372377 | 1.392847332 | Up | 1.36E-33 |
| 729082 | 5.94124284 | 15.59411881 | 1.392165368 | Up | 4.46E-26 |
| 8821 | 0.60618065 | 1.591056508 | 1.392165368 | Up | 7.21E-07 |
| 65980 | 14.9297775 | 39.18494937 | 1.39210697 | Up | 1.14E-94 |
| 83440 | 5.94503828 | 15.59257699 | 1.391101377 | Up | 2.16E-37 |
| 89846 | 0.94998804 | 2.490817214 | 1.390637898 | Up | 1.36E-08 |
| 3292 | 3.02151263 | 7.901945126 | 1.386936853 | Up | 4.78E-16 |
| 56998 | 5.26768709 | 13.77450605 | 1.386759032 | Up | 1.33E-35 |
| 54991 | 5.23406128 | 13.68383377 | 1.386469762 | Up | 2.53E-25 |
| 54432 | 12.0790588 | 31.5551995 | 1.385369698 | Up | 2.02E-48 |
| 51181 | 29.9597383 | 78.25136205 | 1.385090842 | Up | 6.38E-55 |
| 155400 | 3.14621205 | 8.213298724 | 1.384345863 | Up | 5.95E-14 |
| 10068 | 4.95775399 | 12.94148742 | 1.384244852 | Up | 1.00E-24 |
| 837 | 27.7853752 | 72.50673875 | 1.383789364 | Up | 3.85E-81 |
| 7462 | 3.67164153 | 9.580905129 | 1.383736746 | Up | 7.93E-19 |
| 54814 | 3.15394733 | 8.228787355 | 1.383521273 | Up | 3.88E-17 |
| 410 | 10.0004775 | 26.08214647 | 1.382993722 | Up | 1.48E-45 |
| 9989 | 13.5488101 | 35.3229611 | 1.382440138 | Up | 3.58E-116 |
| 83451 | 7.58138677 | 19.75720483 | 1.381845183 | Up | 3.90E-28 |
| 9678 | 15.972612 | 41.60387169 | 1.381117541 | Up | 3.22E-146 |
| 79053 | 5.55635166 | 14.46888755 | 1.380744189 | Up | 3.03E-22 |
| 84250 | 1.16754459 | 3.039392228 | 1.380305213 | Up | 5.33E-11 |
| 79670 | 2.22676053 | 5.795420903 | 1.379967028 | Up | 2.23E-28 |
| 2023 | 309.005218 | 803.9006667 | 1.379386047 | Up | 0 |
| 283951 | 2.04189773 | 5.308374588 | 1.378359568 | Up | 4.77E-06 |
| 653553 | 5.73982276 | 14.92196636 | 1.378359568 | Up | 4.77E-06 |
| 84293 | 26.2278142 | 68.17619488 | 1.378170499 | Up | 1.19E-98 |
| 2617 | 24.604162 | 63.952716 | 1.378103249 | Up | 1.29E-144 |
| 163590 | 0.58819893 | 1.527451294 | 1.376750315 | Up | 3.57E-05 |
| 8818 | 17.3785837 | 45.07179271 | 1.374914325 | Up | 3.14E-58 |
| 126119 | 13.3048622 | 34.50098334 | 1.37468391 | Up | 3.54E-23 |
| 5687 | 94.1374025 | 244.0103443 | 1.374102358 | Up | 1.27E-202 |
| 9034 | 1.80688463 | 4.679337985 | 1.372800043 | Up | 4.99E-08 |
| 84300 | 56.0301176 | 144.9118475 | 1.370901123 | Up | 6.41E-61 |
| 63940 | 7.93899935 | 20.52479893 | 1.370339005 | Up | 8.59E-26 |
| 51123 | 17.1497991 | 44.29351731 | 1.368903889 | Up | 1.07E-103 |
| 85027 | 12.5359597 | 32.36599204 | 1.368406274 | Up | 3.45E-53 |
| 51122 | 7.51408328 | 19.39928173 | 1.368334227 | Up | 6.54E-24 |
| 134637 | 1.17409936 | 3.030688834 | 1.368091233 | Up | 7.11E-17 |
| 25946 | 19.7855558 | 51.05802991 | 1.367690277 | Up | 2.11E-100 |
| 54663 | 21.8412802 | 56.34685079 | 1.367277563 | Up | 1.31E-63 |
| 6222 | 1549.9473 | 3996.605991 | 1.366556183 | Up | 0 |
| 10746 | 3.493356 | 8.998526723 | 1.365075145 | Up | 2.23E-37 |
| 388962 | 34.6809327 | 89.33392982 | 1.36506553 | Up | 2.04E-41 |
| 84791 | 2.57934571 | 6.6436943 | 1.364980538 | Up | 1.98E-05 |
| 91179 | 7.1022593 | 18.28434962 | 1.364259371 | Up | 3.97E-53 |
| 2185 | 3.21355294 | 8.270438081 | 1.363794511 | Up | 6.12E-33 |
| 283234 | 2.09756697 | 5.398017536 | 1.363712792 | Up | 6.47E-23 |
| 3965 | 12.6628913 | 32.55997526 | 1.362492754 | Up | 1.16E-47 |
| 53335 | 1.28726096 | 3.309750747 | 1.362418024 | Up | 2.29E-17 |
| 143187 | 1.9387798 | 4.979826904 | 1.360946637 | Up | 4.23E-19 |
| 9653 | 1.3986108 | 3.592086152 | 1.360827401 | Up | 4.95E-21 |
| 83874 | 9.76282353 | 25.07409724 | 1.360827401 | Up | 1.79E-40 |
| 10082 | 2.70761509 | 6.953948843 | 1.36080979 | Up | 4.11E-41 |
| 5871 | 3.83244511 | 9.841738922 | 1.360648118 | Up | 6.87E-25 |
| 79961 | 1.68291571 | 4.319028423 | 1.35974389 | Up | 1.58E-08 |
| 83596 | 5.22087382 | 13.39883054 | 1.35974389 | Up | 1.78E-21 |
| 79763 | 31.4412983 | 80.67525304 | 1.359465404 | Up | 6.10E-77 |
| 6478 | 9.62867786 | 24.70404695 | 1.359337783 | Up | 2.61E-53 |
| 283991 | 15.6303481 | 40.09073007 | 1.358918786 | Up | 1.79E-52 |
| 221955 | 4.26892799 | 10.9494964 | 1.358918786 | Up | 4.63E-27 |
| 1476 | 87.0048589 | 223.0950849 | 1.358490851 | Up | 1.68E-118 |
| 23184 | 7.19729248 | 18.44963077 | 1.358065752 | Up | 5.09E-64 |
| 54440 | 1.71239417 | 4.387910355 | 1.357519225 | Up | 6.14E-11 |
| 8879 | 5.57972366 | 14.29450376 | 1.357194958 | Up | 2.94E-67 |
| 100132911 | 2.55954175 | 6.55173631 | 1.355991755 | Up | 5.20E-07 |
| 26007 | 3.07548508 | 7.871429954 | 1.355811767 | Up | 3.42E-28 |
| 5901 | 109.080603 | 279.142885 | 1.355609208 | Up | 4.50E-238 |
| 54962 | 1.64747647 | 4.213841342 | 1.354878135 | Up | 1.10E-05 |
| 91419 | 1.81790507 | 4.649755963 | 1.354878135 | Up | 1.10E-05 |
| 9524 | 63.9997993 | 163.5751104 | 1.353813959 | Up | 3.35E-152 |
| 64960 | 77.6979116 | 198.4972421 | 1.353171236 | Up | 3.97E-153 |
| 1992 | 39.8371561 | 101.7725015 | 1.35316124 | Up | 1.02E-105 |
| 55272 | 29.9617688 | 76.53246977 | 1.352949164 | Up | 5.87E-72 |
| 79751 | 3.31808381 | 8.469201621 | 1.351875647 | Up | 4.40E-20 |
| 115701 | 1.11269441 | 2.835645325 | 1.349619673 | Up | 5.96E-18 |
| 286257 | 17.4095671 | 44.34323255 | 1.348833616 | Up | 4.67E-30 |
| 4327 | 9.02476127 | 22.98191647 | 1.348538435 | Up | 1.33E-60 |
| 3683 | 1.04956444 | 2.672609079 | 1.348458083 | Up | 1.56E-12 |
| 55824 | 1.43947803 | 3.663739727 | 1.347771248 | Up | 1.14E-32 |
| 55640 | 0.70535999 | 1.795272573 | 1.347771248 | Up | 1.33E-06 |
| 64926 | 1.21070881 | 3.079465422 | 1.346828002 | Up | 1.76E-09 |
| 2050 | 13.0859083 | 33.26952424 | 1.346187175 | Up | 2.72E-115 |
| 55840 | 1.37828097 | 3.501485545 | 1.345097114 | Up | 0.00039328 |
| 790955 | 20.6239647 | 52.38829776 | 1.344922886 | Up | 5.56E-28 |
| 956 | 1.39818454 | 3.549150389 | 1.343918923 | Up | 2.80E-09 |
| 27351 | 11.5319258 | 29.25306291 | 1.342954232 | Up | 1.09E-18 |
| 126133 | 6.06206884 | 15.37574349 | 1.342774032 | Up | 4.62E-39 |
| 51692 | 8.27434145 | 20.98343691 | 1.3425346 | Up | 2.44E-37 |
| 10237 | 15.1463704 | 38.41065897 | 1.3425346 | Up | 2.44E-37 |
| 4007 | 2.35485191 | 5.970228559 | 1.34214983 | Up | 7.84E-11 |
| 5883 | 4.01186716 | 10.16723264 | 1.341581312 | Up | 1.72E-18 |
| 83744 | 0.57208035 | 1.44911747 | 1.340884863 | Up | 0.00013363 |
| 26292 | 9.22589158 | 23.3548563 | 1.339962325 | Up | 8.53E-40 |
| 8986 | 7.61855021 | 19.28501599 | 1.339891955 | Up | 6.85E-49 |
| 171586 | 1.96770087 | 4.979271981 | 1.339423906 | Up | 1.58E-09 |
| 79905 | 0.78189465 | 1.978274551 | 1.339196513 | Up | 2.01E-08 |
| 339559 | 1.62789091 | 4.118732796 | 1.339196513 | Up | 2.01E-08 |
| 23160 | 9.16885569 | 23.19260384 | 1.338851205 | Up | 6.03E-65 |
| 4938 | 6.48115284 | 16.39214483 | 1.338682275 | Up | 6.84E-23 |
| 9841 | 1.39543663 | 3.529152405 | 1.338605123 | Up | 5.30E-17 |
| 138428 | 7.57068033 | 19.13870702 | 1.33799851 | Up | 1.01E-13 |
| 4125 | 16.6670536 | 42.11863145 | 1.337459476 | Up | 3.25E-107 |
| 80183 | 1.61036264 | 4.068682613 | 1.337176133 | Up | 3.59E-14 |
| 55300 | 1.84357678 | 4.6554444 | 1.336411386 | Up | 1.28E-14 |
| 92106 | 1.07562861 | 2.714016246 | 1.335249328 | Up | 8.87E-10 |
| 4645 | 0.30354998 | 0.76563095 | 1.334715096 | Up | 4.15E-07 |
| 7163 | 6.00081163 | 15.13419433 | 1.334582326 | Up | 1.74E-51 |
| 79139 | 13.6389208 | 34.38029329 | 1.333852357 | Up | 7.88E-86 |
| 284207 | 18.1770288 | 45.801285 | 1.333271679 | Up | 6.28E-49 |
| 79918 | 2.88491763 | 7.269226238 | 1.333271679 | Up | 2.18E-18 |
| 10200 | 7.0937864 | 17.87445771 | 1.333271679 | Up | 7.51E-17 |
| 9415 | 10.7146136 | 26.98872197 | 1.332776842 | Up | 1.95E-67 |
| 54896 | 3.67134118 | 9.244340135 | 1.332263154 | Up | 9.63E-18 |
| 3988 | 15.1678342 | 38.18599492 | 1.332028512 | Up | 4.35E-80 |
| 28998 | 16.9574088 | 42.68220108 | 1.331718843 | Up | 2.61E-38 |
| 6891 | 5.17108173 | 13.00151634 | 1.330141879 | Up | 1.20E-58 |
| 7690 | 7.73872439 | 19.4509786 | 1.329675055 | Up | 1.02E-37 |
| 8530 | 7.40627992 | 18.61473249 | 1.329623902 | Up | 6.49E-15 |
| 8832 | 0.62099917 | 1.560295001 | 1.32915557 | Up | 2.53E-05 |
| 92689 | 12.9186701 | 32.44827911 | 1.328684409 | Up | 1.44E-54 |
| 51728 | 7.94762518 | 19.96180714 | 1.328646593 | Up | 8.06E-14 |
| 4192 | 46.3065092 | 116.2509161 | 1.327955178 | Up | 9.78E-90 |
| 8882 | 11.2892942 | 28.32713944 | 1.327229623 | Up | 2.03E-40 |
| 10981 | 11.1667873 | 27.99192577 | 1.325796558 | Up | 1.60E-27 |
| 10036 | 3.85489891 | 9.658813472 | 1.325152946 | Up | 1.95E-26 |
| 55223 | 1.93222549 | 4.840223163 | 1.3248101 | Up | 7.42E-16 |
| 79930 | 2.55193323 | 6.392590523 | 1.3248101 | Up | 5.67E-13 |
| 84787 | 3.21340229 | 8.04956992 | 1.3248101 | Up | 7.42E-16 |
| 9394 | 4.74307216 | 11.8775088 | 1.324338554 | Up | 1.16E-37 |
| 3122 | 187.994737 | 470.7184627 | 1.324172163 | Up | 0 |
| 8884 | 5.4386222 | 13.60422735 | 1.322741905 | Up | 2.05E-34 |
| 10592 | 2.90560224 | 7.267113768 | 1.322545272 | Up | 2.67E-35 |
| 10626 | 6.54220232 | 16.35647406 | 1.322013501 | Up | 5.90E-38 |
| 83732 | 15.6079052 | 39.01880344 | 1.321892617 | Up | 1.66E-75 |
| 440689 | 1.20821683 | 3.020223748 | 1.32177604 | Up | 1.40E-05 |
| 5724 | 4.99543807 | 12.48583118 | 1.321608761 | Up | 8.87E-19 |
| 103910 | 254.310578 | 635.4015412 | 1.321077117 | Up | 0 |
| 10130 | 18.4856212 | 46.18122767 | 1.320903002 | Up | 1.08E-83 |
| 53838 | 13.3391195 | 33.32323114 | 1.320864858 | Up | 3.78E-55 |
| 26580 | 20.9244422 | 52.25194558 | 1.320295593 | Up | 7.38E-83 |
| 5971 | 7.54830839 | 18.84925284 | 1.320282066 | Up | 2.89E-34 |
| 84166 | 2.50725107 | 6.260980202 | 1.320282066 | Up | 2.89E-34 |
| 56241 | 0.90788431 | 2.267018089 | 1.320215526 | Up | 5.91E-07 |
| 170394 | 4.42541465 | 11.0504113 | 1.320215526 | Up | 1.14E-23 |
| 6929 | 6.16738098 | 15.39750155 | 1.319966399 | Up | 7.16E-54 |
| 79675 | 3.19794219 | 7.982747624 | 1.31974154 | Up | 6.46E-20 |
| 147040 | 5.41988501 | 13.52783426 | 1.319596742 | Up | 3.50E-33 |
| 3117 | 5.26761944 | 13.14065011 | 1.318813626 | Up | 4.81E-17 |
| 94031 | 23.1506292 | 57.74734473 | 1.318703206 | Up | 4.38E-113 |
| 55741 | 7.5938167 | 18.93240993 | 1.317960984 | Up | 2.41E-32 |
| 29107 | 11.8866965 | 29.62505118 | 1.317469823 | Up | 7.93E-27 |
| 6773 | 12.5846656 | 31.32951466 | 1.315855536 | Up | 9.23E-107 |
| 8631 | 1.04070708 | 2.589931295 | 1.315349771 | Up | 0.00019147 |
| 84321 | 2.56354228 | 6.379699471 | 1.315349771 | Up | 2.24E-10 |
| 136647 | 8.40344304 | 20.91303172 | 1.315349771 | Up | 1.20E-16 |
| 147015 | 1.70760474 | 4.249590548 | 1.315349771 | Up | 1.16E-07 |
| 63943 | 2.44070588 | 6.074005538 | 1.315349771 | Up | 1.16E-07 |
| 25816 | 5.80413249 | 14.43220069 | 1.314138948 | Up | 1.43E-23 |
| 317648 | 2.63449312 | 6.543661389 | 1.312572686 | Up | 1.05E-20 |
| 57415 | 5.39691939 | 13.39924038 | 1.311943169 | Up | 2.83E-09 |
| 90378 | 9.01877462 | 22.38825136 | 1.311738517 | Up | 1.28E-23 |
| 5062 | 10.2796695 | 25.51497879 | 1.311550556 | Up | 8.81E-120 |
| 10870 | 6.77813389 | 16.82032303 | 1.311245372 | Up | 6.47E-08 |
| 79710 | 4.27284018 | 10.60155201 | 1.311008224 | Up | 4.28E-32 |
| 55266 | 3.62176021 | 8.981912869 | 1.310331696 | Up | 3.16E-33 |
| 57128 | 8.9064355 | 22.08282175 | 1.31000447 | Up | 2.48E-26 |
| 11018 | 7.19441675 | 17.8325999 | 1.309567418 | Up | 7.28E-20 |
| 84619 | 9.19084038 | 22.77523959 | 1.309197544 | Up | 6.22E-36 |
| 51316 | 1.58168649 | 3.919260556 | 1.309117817 | Up | 1.25E-05 |
| 280636 | 20.4611881 | 50.69494576 | 1.308952 | Up | 4.58E-51 |
| 55159 | 2.64968393 | 6.564636033 | 1.308894749 | Up | 3.91E-26 |
| 79576 | 17.7032533 | 43.82539615 | 1.307752623 | Up | 1.44E-53 |
| 84894 | 0.61267378 | 1.516429759 | 1.307487653 | Up | 0.00010529 |
| 5290 | 1.93729796 | 4.795008955 | 1.307487653 | Up | 5.85E-15 |
| 4199 | 6.79152941 | 16.80028691 | 1.306677469 | Up | 4.83E-44 |
| 27158 | 2.64293008 | 6.53242105 | 1.305479527 | Up | 1.53E-25 |
| 27102 | 14.2800161 | 35.29265328 | 1.305370286 | Up | 1.41E-119 |
| 7260 | 7.31076553 | 18.05250948 | 1.304105014 | Up | 6.69E-25 |
| 7130 | 6.14721706 | 15.16153736 | 1.302410715 | Up | 9.78E-18 |
| 64782 | 4.33624768 | 10.68993786 | 1.301734399 | Up | 1.58E-26 |
| 2495 | 1767.57151 | 4355.373331 | 1.301027803 | Up | 0 |
| 91614 | 1.95844003 | 4.825087649 | 1.300850201 | Up | 1.14E-08 |
| 6248 | 0.92678368 | 2.280209199 | 1.298861648 | Up | 0.00016953 |
| 1201 | 10.7375749 | 26.40430217 | 1.298104824 | Up | 3.17E-39 |
| 6637 | 63.5342298 | 156.2246813 | 1.298016422 | Up | 7.17E-69 |
| 26043 | 5.46321993 | 13.43161326 | 1.297809189 | Up | 6.65E-49 |
| 10797 | 6.26052284 | 15.38854491 | 1.297501769 | Up | 9.25E-28 |
| 79077 | 22.4544941 | 55.18667889 | 1.297315848 | Up | 1.41E-47 |
| 6373 | 0.99996575 | 2.455797387 | 1.296240948 | Up | 0.00049945 |
| 381 | 79.4601997 | 195.0389263 | 1.295457765 | Up | 9.83E-143 |
| 5337 | 3.05928578 | 7.509126503 | 1.295450213 | Up | 8.77E-33 |
| 285 | 1.95664104 | 4.800182019 | 1.294710005 | Up | 2.33E-20 |
| 4326 | 2.27042408 | 5.569520028 | 1.294591211 | Up | 1.78E-11 |
| 150372 | 0.71078373 | 1.742862403 | 1.29397612 | Up | 1.01E-08 |
| 23542 | 1.18442819 | 2.904252425 | 1.29397612 | Up | 1.01E-08 |
| 8974 | 11.0880765 | 27.18773074 | 1.293946623 | Up | 2.27E-53 |
| 9051 | 1.68415285 | 4.125739611 | 1.292629694 | Up | 4.15E-07 |
| 8717 | 12.753594 | 31.23071673 | 1.292061816 | Up | 7.79E-36 |
| 126917 | 4.69900728 | 11.50666939 | 1.292042397 | Up | 3.27E-51 |
| 9536 | 5.11646295 | 12.52891176 | 1.292042397 | Up | 3.92E-18 |
| 3163 | 19.5995678 | 47.97446018 | 1.291444729 | Up | 4.65E-67 |
| 80270 | 14.2453479 | 34.84806428 | 1.290587663 | Up | 1.59E-58 |
| 83443 | 60.4049868 | 147.7540994 | 1.290458595 | Up | 8.15E-82 |
| 10606 | 15.6485869 | 38.26678062 | 1.290060152 | Up | 1.24E-95 |
| 341676 | 7.19295314 | 17.58330675 | 1.289550301 | Up | 2.51E-39 |
| 10952 | 75.879027 | 185.4625178 | 1.289354562 | Up | 2.75E-78 |
| 80736 | 0.62292228 | 1.522538418 | 1.289354562 | Up | 0.00027319 |
| 9976 | 14.179567 | 34.65580785 | 1.289283666 | Up | 1.28E-53 |
| 79585 | 4.88974656 | 11.94750321 | 1.288877559 | Up | 1.54E-32 |
| 5281 | 3.79138778 | 9.263796619 | 1.288877559 | Up | 3.17E-09 |
| 6635 | 13.6818367 | 33.4050673 | 1.287805047 | Up | 1.27E-39 |
| 2530 | 1.8284192 | 4.459764641 | 1.286370704 | Up | 7.20E-14 |
| 4644 | 1.27167526 | 3.101112302 | 1.286055464 | Up | 1.94E-29 |
| 65010 | 3.6892105 | 8.994243279 | 1.285689797 | Up | 1.28E-19 |
| 3001 | 4.27123529 | 10.41258092 | 1.285602427 | Up | 2.54E-08 |
| 63933 | 10.80345 | 26.33222254 | 1.285337202 | Up | 2.39E-25 |
| 140823 | 110.122955 | 268.310301 | 1.284787213 | Up | 4.61E-92 |
| 51253 | 42.3428732 | 103.1147427 | 1.284059543 | Up | 2.83E-113 |
| 5083 | 0.58003214 | 1.412103322 | 1.283640911 | Up | 0.00014985 |
| 26035 | 1.14606217 | 2.789489753 | 1.283315943 | Up | 7.92E-12 |
| 10329 | 3.98317817 | 9.694966799 | 1.283315943 | Up | 7.92E-12 |
| 63951 | 0.94998804 | 2.31139394 | 1.282781908 | Up | 2.83E-05 |
| 54923 | 6.44883756 | 15.68400429 | 1.282182907 | Up | 2.60E-15 |
| 84720 | 2.53492541 | 6.165108153 | 1.282182907 | Up | 6.44E-20 |
| 7029 | 9.57977059 | 23.29001498 | 1.281648556 | Up | 2.24E-23 |
| 10892 | 3.64965366 | 8.870053026 | 1.281183168 | Up | 3.62E-34 |
| 5436 | 24.787049 | 60.23085731 | 1.280916274 | Up | 2.67E-38 |
| 284252 | 3.32351439 | 8.072709196 | 1.280343321 | Up | 8.51E-22 |
| 475 | 63.8875977 | 155.16662 | 1.280210435 | Up | 7.25E-57 |
| 9518 | 1.95256471 | 4.737724319 | 1.278823895 | Up | 1.57E-05 |
| 10762 | 4.03746866 | 9.796558041 | 1.278823895 | Up | 7.66E-39 |
| 79639 | 2.44070588 | 5.922155399 | 1.278823895 | Up | 2.26E-08 |
| 79072 | 1.46320419 | 3.550334627 | 1.278823895 | Up | 1.15E-07 |
| 389792 | 9.5971077 | 23.26501014 | 1.277490227 | Up | 2.15E-47 |
| 6650 | 5.07074139 | 12.29141087 | 1.277381921 | Up | 5.90E-44 |
| 10272 | 24.5411956 | 59.44833138 | 1.276430781 | Up | 1.28E-109 |
| 4094 | 6.08739091 | 14.74295484 | 1.276129786 | Up | 5.07E-75 |
| 6617 | 3.41110702 | 8.260281627 | 1.275950953 | Up | 1.70E-17 |
| 83933 | 4.41926627 | 10.70139141 | 1.275919626 | Up | 1.37E-22 |
| 5208 | 0.94964267 | 2.298040268 | 1.274947417 | Up | 2.52E-13 |
| 5351 | 29.7694619 | 72.01457431 | 1.274455765 | Up | 1.29E-157 |
| 154467 | 16.628293 | 40.20599795 | 1.273770667 | Up | 1.26E-19 |
| 1841 | 8.82640836 | 21.31958639 | 1.272281049 | Up | 8.55E-18 |
| 57175 | 17.2561739 | 41.67213014 | 1.271970224 | Up | 5.46E-59 |
| 5919 | 211.615796 | 510.7089521 | 1.271054024 | Up | 1.06E-272 |
| 374 | 10.9141743 | 26.31741979 | 1.269815065 | Up | 4.62E-25 |
| 6503 | 2.48374811 | 5.984449515 | 1.268699677 | Up | 1.85E-16 |
| 55257 | 10.706842 | 25.78292681 | 1.267883032 | Up | 7.24E-32 |
| 5788 | 1.6265267 | 3.91106698 | 1.265767742 | Up | 1.65E-16 |
| 113452 | 36.4950664 | 87.71524613 | 1.265126179 | Up | 7.31E-64 |
| 3215 | 2.01582737 | 4.843193872 | 1.264586657 | Up | 9.01E-08 |
| 3579 | 0.57368113 | 1.376689608 | 1.262882351 | Up | 0.00038809 |
| 1984 | 219.472314 | 526.4856419 | 1.262355231 | Up | 0 |
| 3790 | 5.06158404 | 12.1375836 | 1.261820374 | Up | 9.24E-22 |
| 91010 | 1.78647807 | 4.278722271 | 1.260061832 | Up | 1.14E-35 |
| 6941 | 3.04756016 | 7.297625528 | 1.259772423 | Up | 9.60E-19 |
| 389119 | 3.45453756 | 8.269992155 | 1.259393365 | Up | 1.43E-07 |
| 10400 | 10.4601681 | 25.01911805 | 1.258124902 | Up | 1.73E-19 |
| 6633 | 119.901436 | 286.7603168 | 1.257996455 | Up | 2.94E-177 |
| 64858 | 0.68454747 | 1.637036825 | 1.257864276 | Up | 1.22E-05 |
| 64376 | 1.50740822 | 3.604838027 | 1.257864276 | Up | 1.22E-05 |
| 1609 | 2.72921037 | 6.524708278 | 1.257429795 | Up | 4.75E-23 |
| 2737 | 3.88040136 | 9.275210599 | 1.257174156 | Up | 1.55E-55 |
| 7884 | 20.3293114 | 48.5920443 | 1.257158783 | Up | 6.13E-61 |
| 6091 | 1.27883631 | 3.056408537 | 1.257005785 | Up | 7.44E-18 |
| 3956 | 1437.89944 | 3436.189025 | 1.256846615 | Up | 0 |
| 5691 | 119.329153 | 285.0754748 | 1.256397377 | Up | 3.22E-153 |
| 8482 | 1.36534895 | 3.261066084 | 1.256073961 | Up | 2.76E-09 |
| 55324 | 11.3266973 | 27.03546477 | 1.255125907 | Up | 9.98E-51 |
| 283897 | 0.81231505 | 1.937317637 | 1.253949226 | Up | 6.41E-05 |
| 170384 | 1.36822052 | 3.263115384 | 1.253949226 | Up | 3.76E-06 |
| 10960 | 30.8827274 | 73.64441449 | 1.25377594 | Up | 1.59E-96 |
| 8099 | 62.4142816 | 148.8037215 | 1.25346252 | Up | 3.04E-170 |
| 25800 | 8.52224374 | 20.31603841 | 1.253313887 | Up | 1.96E-53 |
| 6132 | 1175.35906 | 2801.912346 | 1.253310273 | Up | 0 |
| 7923 | 10.5821505 | 25.20497343 | 1.252075596 | Up | 3.79E-19 |
| 26229 | 13.5406706 | 32.24867295 | 1.251940601 | Up | 2.36E-34 |
| 5730 | 22.699767 | 54.03770444 | 1.251288899 | Up | 1.75E-32 |
| 790 | 2.63711609 | 6.275674827 | 1.250809519 | Up | 5.63E-33 |
| 29068 | 3.21107001 | 7.639980413 | 1.25051482 | Up | 1.01E-51 |
| 6745 | 9.00818044 | 21.4272283 | 1.250137612 | Up | 1.90E-149 |
| 923 | 0.73636868 | 1.75126437 | 1.249896709 | Up | 1.95E-05 |
| 56905 | 3.93423925 | 9.35180071 | 1.249159491 | Up | 1.16E-30 |
| 6809 | 4.51460048 | 10.73122502 | 1.24914455 | Up | 9.42E-24 |
| 4726 | 106.277914 | 252.5878041 | 1.24894316 | Up | 3.69E-96 |
| 284184 | 19.665116 | 46.72645689 | 1.248600953 | Up | 6.06E-20 |
| 154807 | 4.49805482 | 10.68516157 | 1.248235575 | Up | 4.30E-10 |
| 55210 | 6.31162241 | 14.99076664 | 1.247991361 | Up | 4.89E-29 |
| 92170 | 7.11343276 | 16.88979979 | 1.247534387 | Up | 1.09E-20 |
| 64121 | 9.57139561 | 22.71371819 | 1.246762685 | Up | 2.67E-24 |
| 55759 | 4.41702494 | 10.48105471 | 1.246637021 | Up | 2.60E-18 |
| 823 | 39.505832 | 93.68552605 | 1.245760533 | Up | 1.43E-197 |
| 28985 | 3.89475404 | 9.235765624 | 1.245699339 | Up | 1.16E-64 |
| 6515 | 15.0769538 | 35.749773 | 1.245589112 | Up | 1.16E-99 |
| 84520 | 4.18406723 | 9.916743735 | 1.244960443 | Up | 1.21E-09 |
| 388796 | 10.0097752 | 23.72242298 | 1.244841795 | Up | 3.09E-21 |
| 55726 | 4.72394687 | 11.19282472 | 1.24450953 | Up | 7.59E-25 |
| 1820 | 1.86223253 | 4.409598326 | 1.243614017 | Up | 3.80E-10 |
| 10280 | 39.8771523 | 94.42417793 | 1.243593931 | Up | 3.56E-110 |
| 29925 | 2.56853822 | 6.081403839 | 1.243456852 | Up | 1.95E-08 |
| 79759 | 2.82656986 | 6.691818672 | 1.243345999 | Up | 8.56E-14 |
| 57510 | 3.85925524 | 9.13309354 | 1.242781145 | Up | 1.05E-35 |
| 55624 | 19.6387428 | 46.46459023 | 1.242429109 | Up | 3.01E-96 |
| 5209 | 9.27543383 | 21.94496657 | 1.242403406 | Up | 6.47E-71 |
| 282974 | 2.21149377 | 5.228403068 | 1.241349189 | Up | 3.41E-09 |
| 162966 | 1.37990439 | 3.262363398 | 1.241349189 | Up | 3.18E-07 |
| 10256 | 3.88049137 | 9.168491009 | 1.240444962 | Up | 1.01E-17 |
| 10228 | 4.73609962 | 11.18634934 | 1.239967959 | Up | 8.55E-37 |
| 79929 | 1.24204303 | 2.931886677 | 1.239114185 | Up | 9.52E-06 |
| 3665 | 7.42564832 | 17.52014469 | 1.238425794 | Up | 1.73E-26 |
| 9060 | 3.94764248 | 9.314104915 | 1.238425794 | Up | 1.73E-26 |
| 284361 | 34.0978851 | 80.44921789 | 1.238396135 | Up | 1.56E-114 |
| 90427 | 2.43289479 | 5.738360683 | 1.237964719 | Up | 3.31E-20 |
| 929 | 38.3218179 | 90.36123307 | 1.237537958 | Up | 1.11E-101 |
| 10849 | 7.46324184 | 17.58984683 | 1.236868578 | Up | 1.56E-41 |
| 118672 | 0.96309709 | 2.268389619 | 1.235915303 | Up | 0.00054884 |
| 100037417 | 3.00945753 | 7.088197288 | 1.235915303 | Up | 1.69E-09 |
| 90637 | 21.781944 | 51.26460674 | 1.234830407 | Up | 2.09E-33 |
| 84930 | 2.09184303 | 4.920293089 | 1.233969659 | Up | 5.94E-14 |
| 134957 | 0.91056958 | 2.141433944 | 1.233736005 | Up | 5.25E-11 |
| 91768 | 1.20157828 | 2.825231747 | 1.233438573 | Up | 2.95E-11 |
| 949 | 7.34882121 | 17.27835273 | 1.233380924 | Up | 3.37E-34 |
| 7535 | 0.8681638 | 2.040505771 | 1.232887611 | Up | 9.03E-05 |
| 64979 | 34.3447864 | 80.72294004 | 1.232887611 | Up | 1.11E-35 |
| 10094 | 109.467315 | 257.2519992 | 1.232682113 | Up | 2.76E-157 |
| 23443 | 1.05396699 | 2.475955724 | 1.232155835 | Up | 4.97E-05 |
| 9533 | 15.3095426 | 35.9417418 | 1.23122915 | Up | 8.24E-34 |
| 10165 | 3.98832139 | 9.362188987 | 1.231064217 | Up | 4.23E-22 |
| 83787 | 14.5866759 | 34.23992471 | 1.231028384 | Up | 2.94E-63 |
| 23765 | 1.51033426 | 3.54509787 | 1.230957583 | Up | 8.36E-10 |
| 3939 | 288.109412 | 675.9972033 | 1.230400488 | Up | 0 |
| 5315 | 340.946714 | 799.697118 | 1.22990741 | Up | 0 |
| 124944 | 41.4892296 | 97.29439971 | 1.229619897 | Up | 3.24E-60 |
| 51026 | 6.75520552 | 15.83845209 | 1.229359778 | Up | 4.52E-37 |
| 9731 | 3.78150334 | 8.861358438 | 1.228567986 | Up | 2.21E-24 |
| 10189 | 21.2553162 | 49.7902043 | 1.228038211 | Up | 7.51E-39 |
| 4175 | 5.47613455 | 12.82487973 | 1.227715496 | Up | 1.84E-34 |
| 54187 | 16.5786769 | 38.82657293 | 1.227715496 | Up | 1.84E-34 |
| 83858 | 7.44751355 | 17.43561639 | 1.227206619 | Up | 1.45E-30 |
| 10678 | 4.71627124 | 11.0299875 | 1.225712558 | Up | 2.94E-22 |
| 9521 | 9.70888528 | 22.68523615 | 1.224376111 | Up | 2.42E-18 |
| 2194 | 5.15266543 | 12.0388542 | 1.224307265 | Up | 1.00E-70 |
| 10534 | 35.3491837 | 82.58418538 | 1.224188638 | Up | 2.54E-38 |
| 9403 | 53.0588235 | 123.949326 | 1.224085835 | Up | 4.97E-156 |
| 3959 | 93.6608727 | 218.6641994 | 1.223198649 | Up | 0 |
| 151194 | 34.7519945 | 81.10977967 | 1.222780095 | Up | 9.48E-265 |
| 27314 | 1.26758258 | 2.957380973 | 1.222240366 | Up | 0.00014429 |
| 10745 | 1.54086487 | 3.594972451 | 1.222240366 | Up | 2.08E-09 |
| 962 | 3.64965366 | 8.514961034 | 1.222240366 | Up | 1.22E-07 |
| 79873 | 1.84858197 | 4.31290334 | 1.222240366 | Up | 7.38E-06 |
| 57520 | 0.24808792 | 0.578810808 | 1.222240366 | Up | 0.00048121 |
| 55329 | 2.62529708 | 6.125047601 | 1.222240366 | Up | 6.52E-10 |
| 5271 | 2.16567649 | 5.052712571 | 1.222240366 | Up | 3.62E-13 |
| 10263 | 30.6629174 | 71.51281992 | 1.221706924 | Up | 2.78E-68 |
| 6146 | 227.227962 | 529.8630327 | 1.2214791 | Up | 0 |
| 7019 | 4.4779893 | 10.42871612 | 1.219638571 | Up | 3.23E-15 |
| 2519 | 12.1848981 | 28.37627015 | 1.219590788 | Up | 8.59E-42 |
| 25896 | 2.45903585 | 5.726618739 | 1.219590788 | Up | 5.74E-15 |
| 54480 | 6.61019386 | 15.38544073 | 1.218801282 | Up | 7.84E-43 |
| 994 | 14.2642452 | 33.19045595 | 1.218365045 | Up | 1.04E-83 |
| 55798 | 5.29751744 | 12.32616706 | 1.218335912 | Up | 9.60E-20 |
| 29105 | 16.576382 | 38.56123113 | 1.218021957 | Up | 3.47E-35 |
| 25873 | 707.577452 | 1645.614372 | 1.217666319 | Up | 0 |
| 284086 | 1.61233475 | 3.748966329 | 1.217341567 | Up | 1.04E-08 |
| 1536 | 4.99219878 | 11.59659603 | 1.215954103 | Up | 2.72E-35 |
| 7993 | 2.33385079 | 5.419751988 | 1.215514503 | Up | 1.11E-06 |
| 493856 | 7.216 | 16.75633267 | 1.215435188 | Up | 2.49E-22 |
| 23178 | 0.70688877 | 1.641188251 | 1.215185609 | Up | 2.00E-06 |
| 80176 | 16.1470519 | 37.48529132 | 1.215053826 | Up | 6.73E-80 |
| 5277 | 1.64654555 | 3.821316105 | 1.214627182 | Up | 8.96E-11 |
| 26578 | 13.2054643 | 30.64296472 | 1.214420862 | Up | 1.27E-28 |
| 1022 | 7.98015898 | 18.49828977 | 1.212902502 | Up | 2.12E-20 |
| 257000 | 1.31193727 | 3.041116834 | 1.212902502 | Up | 5.15E-09 |
| 25893 | 1.40875499 | 3.265543706 | 1.212902502 | Up | 7.87E-13 |
| 2286 | 90.1924409 | 208.8792261 | 1.211590587 | Up | 2.61E-103 |
| 23589 | 14.8273085 | 34.32730378 | 1.211099809 | Up | 1.26E-70 |
| 11274 | 2.95523307 | 6.841091249 | 1.210954559 | Up | 7.91E-11 |
| 3955 | 3.35571356 | 7.767767823 | 1.210880504 | Up | 6.97E-14 |
| 91978 | 3.50550216 | 8.11321386 | 1.210652392 | Up | 1.69E-07 |
| 904 | 0.76034451 | 1.759758614 | 1.210652392 | Up | 0.00023067 |
| 10139 | 11.7422789 | 27.15875162 | 1.209704731 | Up | 6.05E-49 |
| 55321 | 1.04439508 | 2.415476621 | 1.20964033 | Up | 0.00042148 |
| 10447 | 15.1780797 | 35.09637818 | 1.209332883 | Up | 4.98E-61 |
| 10923 | 24.9429423 | 57.67447517 | 1.209301311 | Up | 5.20E-136 |
| 9688 | 10.7048504 | 24.74890853 | 1.209100272 | Up | 9.97E-47 |
| 50486 | 45.0934985 | 104.2231231 | 1.208684043 | Up | 2.23E-68 |
| 399664 | 2.82265494 | 6.52156384 | 1.208165181 | Up | 6.15E-14 |
| 84525 | 11.4843017 | 26.53247607 | 1.208096184 | Up | 3.62E-31 |
| 9046 | 3.618312 | 8.359478808 | 1.208096184 | Up | 2.20E-11 |
| 79888 | 11.2027534 | 25.87840373 | 1.207895269 | Up | 1.81E-69 |
| 126282 | 8.21380672 | 18.96356187 | 1.207107063 | Up | 3.42E-29 |
| 4293 | 0.73918521 | 1.705060126 | 1.205814818 | Up | 8.31E-08 |
| 415116 | 21.118572 | 48.70443128 | 1.205540753 | Up | 2.60E-78 |
| 6775 | 0.96178486 | 2.217529005 | 1.205166853 | Up | 1.86E-05 |
| 1650 | 42.8351918 | 98.73830263 | 1.204813295 | Up | 2.87E-140 |
| 5688 | 137.235137 | 316.2186542 | 1.204272565 | Up | 8.26E-207 |
| 148867 | 4.06962597 | 9.370562232 | 1.203239404 | Up | 1.73E-52 |
| 23533 | 0.78276536 | 1.801909883 | 1.202875041 | Up | 8.61E-07 |
| 1138 | 1.3832007 | 3.182098453 | 1.201967971 | Up | 1.55E-06 |
| 85441 | 2.01464385 | 4.627466726 | 1.199697798 | Up | 4.31E-32 |
| 29927 | 31.4687271 | 72.24658732 | 1.199010618 | Up | 1.02E-173 |
| 23643 | 19.8565902 | 45.58592631 | 1.198970587 | Up | 2.73E-19 |
| 7128 | 5.63917604 | 12.94289447 | 1.198604002 | Up | 2.53E-39 |
| 30850 | 4.04550627 | 9.283789953 | 1.198393624 | Up | 3.60E-23 |
| 10457 | 53.9884847 | 123.8780912 | 1.198197429 | Up | 9.82E-226 |
| 1672 | 4.06607835 | 9.328407637 | 1.19799282 | Up | 0.00036883 |
| 1032 | 4.6336775 | 10.62842427 | 1.197698182 | Up | 2.68E-11 |
| 54587 | 48.1397827 | 110.4063409 | 1.197521497 | Up | 1.01E-165 |
| 10539 | 26.369041 | 60.46662747 | 1.197294009 | Up | 6.00E-51 |
| 3784 | 0.84214128 | 1.931107121 | 1.197294009 | Up | 1.63E-05 |
| 84269 | 17.2916577 | 39.63897819 | 1.196843589 | Up | 4.26E-19 |
| 11073 | 3.11183152 | 7.130650721 | 1.196269787 | Up | 1.33E-26 |
| 93109 | 2.19819651 | 5.03533764 | 1.195768155 | Up | 3.10E-09 |
| 90956 | 6.83893222 | 15.66291783 | 1.195510001 | Up | 7.33E-26 |
| 7903 | 0.65507789 | 1.499517113 | 1.19475963 | Up | 1.15E-07 |
| 3433 | 2.12296019 | 4.85920443 | 1.194642808 | Up | 1.17E-12 |
| 23513 | 7.21453575 | 16.51086519 | 1.194437255 | Up | 6.91E-58 |
| 606495 | 0.68530434 | 1.56792897 | 1.194043475 | Up | 5.36E-05 |
| 93323 | 3.32579703 | 7.609193751 | 1.194043475 | Up | 9.89E-09 |
| 5261 | 11.7302366 | 26.83648074 | 1.193963376 | Up | 2.72E-29 |
| 56954 | 25.6245313 | 58.60865768 | 1.193588179 | Up | 3.11E-50 |
| 84938 | 1.72888961 | 3.952977594 | 1.193094021 | Up | 4.40E-06 |
| 54805 | 0.94377973 | 2.156983471 | 1.192493023 | Up | 3.70E-07 |
| 1434 | 17.2172232 | 39.34031556 | 1.192156048 | Up | 1.02E-92 |
| 8538 | 2.87304371 | 6.558688492 | 1.190827414 | Up | 4.87E-09 |
| 151987 | 3.95320898 | 9.023761989 | 1.190704793 | Up | 4.23E-10 |
| 8772 | 7.75546478 | 17.70082564 | 1.190531507 | Up | 6.02E-23 |
| 4597 | 8.78926062 | 20.05944559 | 1.19046802 | Up | 4.91E-25 |
| 55110 | 4.3609045 | 9.948274289 | 1.189818889 | Up | 2.88E-18 |
| 11173 | 1.2803703 | 2.920833264 | 1.189818889 | Up | 5.79E-12 |
| 23528 | 4.61384219 | 10.5160857 | 1.18855724 | Up | 1.22E-25 |
| 4234 | 8.78430028 | 20.02051711 | 1.18847996 | Up | 1.43E-18 |
| 126433 | 4.64175589 | 10.5785006 | 1.188392596 | Up | 1.60E-17 |
| 57502 | 0.46669385 | 1.063217841 | 1.187888862 | Up | 2.59E-05 |
| 132949 | 1.79848701 | 4.093685282 | 1.186616457 | Up | 5.80E-11 |
| 4758 | 12.9731001 | 29.5282896 | 1.186574517 | Up | 3.56E-41 |
| 161753 | 2.09495626 | 4.765523205 | 1.18571449 | Up | 4.69E-05 |
| 8551 | 1.33366213 | 3.033761571 | 1.18571449 | Up | 4.69E-05 |
| 9540 | 7.69368779 | 17.49313595 | 1.185041748 | Up | 1.59E-20 |
| 345757 | 8.40424291 | 19.10658053 | 1.184879839 | Up | 7.83E-17 |
| 908 | 37.1456379 | 84.43414905 | 1.184633809 | Up | 2.98E-146 |
| 25963 | 18.4931968 | 42.03122954 | 1.184467024 | Up | 5.41E-83 |
| 10135 | 20.237453 | 45.98264834 | 1.184061829 | Up | 5.52E-137 |
| 11119 | 6.22732501 | 14.14609645 | 1.183719521 | Up | 8.33E-40 |
| 51661 | 4.89823262 | 11.12660697 | 1.18368052 | Up | 2.01E-22 |
| 51527 | 4.2697593 | 9.697251221 | 1.183421117 | Up | 1.75E-14 |
| 80256 | 8.15073246 | 18.51087289 | 1.183371311 | Up | 1.00E-37 |
| 84734 | 2.45091804 | 5.565720555 | 1.183246235 | Up | 8.51E-05 |
| 3703 | 24.6595757 | 55.95972851 | 1.182240989 | Up | 3.00E-102 |
| 84515 | 2.74414499 | 6.225034865 | 1.181725207 | Up | 2.17E-16 |
| 3954 | 4.66156056 | 10.56890308 | 1.180940737 | Up | 1.11E-38 |
| 64840 | 5.39648306 | 12.21958479 | 1.179103863 | Up | 3.38E-16 |
| 5437 | 30.8224587 | 69.78077982 | 1.178847771 | Up | 2.67E-38 |
| 27071 | 2.6858568 | 6.080209833 | 1.178738728 | Up | 5.46E-13 |
| 1777 | 15.6360759 | 35.39136693 | 1.178518989 | Up | 4.42E-47 |
| 9219 | 23.8136323 | 53.88000206 | 1.177962215 | Up | 8.97E-107 |
| 55829 | 21.7018802 | 49.08097354 | 1.177343828 | Up | 3.95E-42 |
| 192111 | 1.7642635 | 3.989529267 | 1.177152477 | Up | 0.00028181 |
| 9650 | 3.71508835 | 8.400929723 | 1.177152477 | Up | 2.98E-16 |
| 160428 | 1.54402092 | 3.491122726 | 1.176998778 | Up | 2.10E-18 |
| 80267 | 1.81360051 | 4.09940422 | 1.176557551 | Up | 5.90E-19 |
| 10650 | 1.72753105 | 3.903202627 | 1.175946714 | Up | 1.10E-05 |
| 6653 | 3.65346234 | 8.246947101 | 1.174595818 | Up | 6.51E-59 |
| 6218 | 1435.15519 | 3239.386129 | 1.174513696 | Up | 0 |
| 9448 | 14.0310321 | 31.66072333 | 1.174073084 | Up | 2.59E-151 |
| 5550 | 7.89524233 | 17.80628089 | 1.173330766 | Up | 7.29E-34 |
| 397 | 40.849709 | 92.12907742 | 1.173330766 | Up | 1.06E-72 |
| 1038 | 2.16450591 | 4.881648792 | 1.173330766 | Up | 1.99E-05 |
| 6351 | 9.01634077 | 20.32560774 | 1.172684542 | Up | 3.97E-10 |
| 112770 | 14.9799286 | 33.75384276 | 1.172021011 | Up | 1.46E-35 |
| 4815 | 5.14800487 | 11.59894843 | 1.171908691 | Up | 2.56E-09 |
| 2512 | 1639.07831 | 3692.939578 | 1.171884873 | Up | 0 |
| 6447 | 3.641439 | 8.202837061 | 1.171614293 | Up | 6.03E-08 |
| 1786 | 6.36509332 | 14.33730813 | 1.171520609 | Up | 1.16E-50 |
| 140701 | 2.0229274 | 4.555504153 | 1.171166182 | Up | 1.45E-06 |
| 51067 | 5.16828788 | 11.6364567 | 1.170893486 | Up | 1.60E-17 |
| 5150 | 4.23522807 | 9.532410605 | 1.170401435 | Up | 1.59E-20 |
| 7023 | 4.34511828 | 9.779745242 | 1.170401435 | Up | 8.12E-15 |
| 23642 | 33.6022763 | 75.62625857 | 1.170328278 | Up | 4.33E-55 |
| 56063 | 4.04227813 | 9.094167256 | 1.169772946 | Up | 1.08E-07 |
| 2177 | 0.84045829 | 1.890831855 | 1.169772946 | Up | 1.08E-07 |
| 8894 | 66.3510414 | 149.2068212 | 1.169122478 | Up | 7.21E-245 |
| 51109 | 19.9287866 | 44.80614291 | 1.168842665 | Up | 3.77E-74 |
| 79666 | 4.78290032 | 10.7531455 | 1.168801107 | Up | 3.94E-21 |
| 163859 | 1.81180861 | 4.072568442 | 1.168508382 | Up | 7.47E-12 |
| 79228 | 15.2355049 | 34.23685938 | 1.168113057 | Up | 2.65E-32 |
| 55195 | 0.83947846 | 1.886036751 | 1.167792582 | Up | 0.00024609 |
| 10213 | 23.4990878 | 52.78541022 | 1.167534474 | Up | 1.21E-58 |
| 84524 | 3.12215218 | 7.011094462 | 1.167098812 | Up | 1.45E-08 |
| 11079 | 23.8625754 | 53.5407976 | 1.165888872 | Up | 1.59E-105 |
| 84769 | 7.99430027 | 17.92681458 | 1.165075491 | Up | 6.87E-17 |
| 1871 | 1.35654326 | 3.041246933 | 1.164727908 | Up | 4.20E-11 |
| 80772 | 8.51186438 | 19.07786144 | 1.16435239 | Up | 6.86E-28 |
| 374403 | 1.83231879 | 4.103961513 | 1.163346677 | Up | 8.44E-06 |
| 65992 | 30.8922518 | 69.16810134 | 1.162861819 | Up | 6.49E-56 |
| 9026 | 3.89377983 | 8.717888565 | 1.162807453 | Up | 4.72E-26 |
| 83640 | 10.0865472 | 22.57876404 | 1.16253412 | Up | 8.43E-18 |
| 79717 | 23.9185898 | 53.50346315 | 1.161499943 | Up | 2.09E-51 |
| 5027 | 1.44403893 | 3.229659304 | 1.161272349 | Up | 8.30E-08 |
| 1603 | 143.31881 | 320.3101113 | 1.160241374 | Up | 1.62E-140 |
| 2359 | 3.22712323 | 7.210619208 | 1.159874485 | Up | 7.00E-13 |
| 54435 | 1.07094636 | 2.391528135 | 1.15904654 | Up | 0.00021479 |
| 55236 | 2.07159029 | 4.624045165 | 1.15841679 | Up | 3.59E-20 |
| 340390 | 0.50466625 | 1.126237858 | 1.158110029 | Up | 2.00E-06 |
| 22856 | 5.76943044 | 12.87394514 | 1.157953419 | Up | 5.95E-38 |
| 6202 | 1464.56201 | 3267.039574 | 1.157514653 | Up | 0 |
| 92002 | 10.733042 | 23.9376181 | 1.157220574 | Up | 8.88E-21 |
| 222487 | 1.58078702 | 3.524198116 | 1.156652025 | Up | 2.66E-07 |
| 90874 | 0.75335174 | 1.678721216 | 1.155967147 | Up | 9.75E-07 |
| 79943 | 1.1672035 | 2.59869921 | 1.154733547 | Up | 4.83E-09 |
| 375346 | 1.9302921 | 4.295684525 | 1.154068864 | Up | 1.33E-05 |
| 3113 | 103.686755 | 230.6337176 | 1.153371829 | Up | 5.32E-166 |
| 150864 | 0.81187722 | 1.80578543 | 1.153293013 | Up | 6.38E-08 |
| 9791 | 23.1126269 | 51.40588073 | 1.153252167 | Up | 4.72E-81 |
| 285590 | 7.09201637 | 15.7681965 | 1.152749888 | Up | 3.12E-77 |
| 2005 | 0.87697754 | 1.949779849 | 1.152699433 | Up | 4.97E-05 |
| 246777 | 1.89272131 | 4.208077963 | 1.152699433 | Up | 4.97E-05 |
| 65990 | 8.10155053 | 18.0096388 | 1.152499295 | Up | 4.44E-11 |
| 10126 | 2.88879782 | 6.421208275 | 1.152375559 | Up | 2.33E-07 |
| 10478 | 5.69462088 | 12.65798061 | 1.152375559 | Up | 2.71E-19 |
| 6018 | 4.19879514 | 9.329672506 | 1.151851038 | Up | 1.33E-37 |
| 3015 | 94.2674322 | 209.4281191 | 1.151623824 | Up | 1.37E-123 |
| 51150 | 31.5990359 | 70.16073001 | 1.150783216 | Up | 7.85E-94 |
| 51497 | 22.0085273 | 48.82886862 | 1.14967174 | Up | 2.54E-69 |
| 5538 | 46.7555031 | 103.7141297 | 1.149404372 | Up | 9.82E-161 |
| 4502 | 410.879405 | 911.275204 | 1.149171792 | Up | 9.88E-235 |
| 5778 | 2.0345449 | 4.51222477 | 1.149132818 | Up | 1.40E-10 |
| 23452 | 23.1990051 | 51.44199763 | 1.148883733 | Up | 3.23E-114 |
| 83786 | 4.93476764 | 10.94001104 | 1.148560135 | Up | 7.85E-27 |
| 550 | 57.0543548 | 126.451474 | 1.14817494 | Up | 1.12E-125 |
| 10471 | 46.4145011 | 102.828619 | 1.147594332 | Up | 4.36E-39 |
| 55892 | 2.86683615 | 6.351242305 | 1.147579361 | Up | 8.97E-14 |
| 5625 | 2.93193796 | 6.494323424 | 1.14732466 | Up | 2.50E-10 |
| 6352 | 49.3781213 | 109.3024463 | 1.146381839 | Up | 1.01E-83 |
| 132660 | 1.33868295 | 2.962639332 | 1.146068686 | Up | 1.15E-12 |
| 1522 | 81.1245696 | 179.4242596 | 1.145164143 | Up | 2.01E-165 |
| 55105 | 3.97868493 | 8.798987474 | 1.145045861 | Up | 3.90E-14 |
| 5080 | 0.51738466 | 1.144124315 | 1.14493464 | Up | 2.75E-06 |
| 79768 | 4.81727643 | 10.64059364 | 1.143289025 | Up | 2.20E-19 |
| 51069 | 20.406275 | 45.05910795 | 1.142805899 | Up | 6.24E-37 |
| 9014 | 1.3940819 | 3.077989746 | 1.142673109 | Up | 1.02E-05 |
| 219293 | 2.10486374 | 4.646401748 | 1.142387059 | Up | 1.79E-12 |
| 79001 | 63.5463944 | 140.2255387 | 1.14186695 | Up | 1.33E-86 |
| 1173 | 142.322954 | 314.0082492 | 1.141634096 | Up | 0 |
| 6897 | 24.3077178 | 53.63027205 | 1.141633123 | Up | 2.19E-94 |
| 6921 | 47.9347183 | 105.7155162 | 1.141044283 | Up | 1.07E-43 |
| 84975 | 9.8072 | 21.61793789 | 1.140315709 | Up | 1.00E-24 |
| 7518 | 2.16439956 | 4.770386425 | 1.140139286 | Up | 2.41E-06 |
| 712 | 67.5039674 | 148.7633487 | 1.139974928 | Up | 1.65E-100 |
| 84661 | 41.8825129 | 92.29451415 | 1.139896893 | Up | 2.07E-46 |
| 92140 | 24.7063366 | 54.42359671 | 1.139351196 | Up | 2.00E-253 |
| 9277 | 14.3219905 | 31.54324636 | 1.139099131 | Up | 1.02E-40 |
| 84191 | 9.94162982 | 21.89311886 | 1.138923203 | Up | 4.47E-16 |
| 55364 | 5.1541889 | 11.34633286 | 1.138408776 | Up | 1.90E-27 |
| 4628 | 7.91379421 | 17.42081231 | 1.138370441 | Up | 7.69E-82 |
| 11335 | 38.5660538 | 84.85887801 | 1.13773407 | Up | 2.00E-96 |
| 84933 | 7.16648943 | 15.75651825 | 1.136610295 | Up | 8.28E-14 |
| 5610 | 5.17129765 | 11.36854825 | 1.136449784 | Up | 9.87E-22 |
| 23299 | 9.10547631 | 20.01695341 | 1.13641602 | Up | 4.91E-79 |
| 54982 | 9.62335462 | 21.150555 | 1.136083723 | Up | 3.17E-30 |
| 55365 | 45.1460048 | 99.2204604 | 1.136039331 | Up | 1.04E-63 |
| 595135 | 17.070089 | 37.51591656 | 1.136032224 | Up | 2.95E-46 |
| 746 | 93.3050385 | 205.0087708 | 1.13565874 | Up | 2.05E-52 |
| 115939 | 39.3107711 | 86.32756043 | 1.134896554 | Up | 3.55E-64 |
| 151579 | 2.67044248 | 5.863883061 | 1.134777525 | Up | 2.62E-10 |
| 26155 | 19.6930097 | 43.23824228 | 1.134624256 | Up | 1.99E-74 |
| 5480 | 26.0341961 | 57.1428288 | 1.1341646 | Up | 3.98E-44 |
| 9184 | 19.2430762 | 42.21917308 | 1.133558876 | Up | 1.07E-71 |
| 25764 | 50.9339193 | 111.7142416 | 1.133114476 | Up | 8.49E-91 |
| 57819 | 34.6312527 | 75.94987554 | 1.132973028 | Up | 9.85E-41 |
| 122416 | 8.79014369 | 19.27544193 | 1.132805283 | Up | 1.93E-20 |
| 10973 | 2.32497167 | 5.09794037 | 1.13270136 | Up | 7.13E-25 |
| 23144 | 5.38638539 | 11.8106693 | 1.13270136 | Up | 7.13E-25 |
| 57486 | 1.3302144 | 2.913495878 | 1.131092478 | Up | 1.84E-06 |
| 528 | 7.079074 | 15.50021469 | 1.130655636 | Up | 1.54E-54 |
| 8420 | 41.1722909 | 90.12038887 | 1.130179813 | Up | 3.06E-52 |
| 7372 | 5.37726623 | 11.76410102 | 1.129446271 | Up | 2.62E-20 |
| 7295 | 270.284154 | 591.282917 | 1.129371665 | Up | 1.71E-180 |
| 93349 | 5.53275456 | 12.1024788 | 1.129232736 | Up | 6.48E-21 |
| 114803 | 2.89604035 | 6.334420607 | 1.129130962 | Up | 2.42E-11 |
| 51491 | 10.1809705 | 22.26004264 | 1.128581259 | Up | 1.24E-08 |
| 8938 | 0.70798278 | 1.547038333 | 1.127722768 | Up | 1.40E-05 |
| 79168 | 1.62962143 | 3.560943705 | 1.127722768 | Up | 7.26E-10 |
| 5902 | 76.6006154 | 167.3787046 | 1.1276881 | Up | 1.11E-89 |
| 5551 | 1.5410929 | 3.365392176 | 1.126820801 | Up | 1.61E-06 |
| 55171 | 4.05562101 | 8.85511454 | 1.126588127 | Up | 9.55E-18 |
| 219285 | 1.19349252 | 2.605699838 | 1.126481384 | Up | 1.27E-12 |
| 113 | 2.25222517 | 4.916203896 | 1.126193679 | Up | 1.44E-19 |
| 6156 | 1256.29173 | 2741.890294 | 1.125999329 | Up | 0 |
| 197259 | 4.98659781 | 10.87794767 | 1.125278636 | Up | 8.38E-18 |
| 391 | 43.7587663 | 95.43418557 | 1.124934085 | Up | 2.80E-76 |
| 22883 | 26.0392104 | 56.77182988 | 1.124489544 | Up | 6.05E-176 |
| 6631 | 66.1626414 | 144.1404961 | 1.123386979 | Up | 1.78E-62 |
| 1018 | 3.1340529 | 6.824551445 | 1.122704693 | Up | 8.02E-08 |
| 51411 | 3.28518349 | 7.153645599 | 1.122704693 | Up | 6.67E-11 |
| 126070 | 1.5691503 | 3.416900508 | 1.122704693 | Up | 5.56E-10 |
| 79837 | 7.35397273 | 16.01363051 | 1.122704693 | Up | 3.80E-32 |
| 28959 | 51.1574686 | 111.3468503 | 1.122043967 | Up | 1.53E-95 |
| 3265 | 25.5700532 | 55.64064716 | 1.121684038 | Up | 3.22E-42 |
| 7407 | 9.22600471 | 20.0730882 | 1.121484655 | Up | 1.61E-52 |
| 222484 | 2.30861106 | 5.022054537 | 1.121252559 | Up | 7.60E-16 |
| 7443 | 5.27410314 | 11.47034751 | 1.120911411 | Up | 4.22E-13 |
| 220002 | 6.71745539 | 14.60486966 | 1.120462742 | Up | 2.70E-29 |
| 25884 | 22.2950773 | 48.4327736 | 1.119258421 | Up | 1.72E-49 |
| 55183 | 3.25155711 | 7.060134792 | 1.118564962 | Up | 1.81E-36 |
| 51504 | 158.356022 | 343.6151704 | 1.117621998 | Up | 1.20E-118 |
| 5361 | 4.54932817 | 9.868571025 | 1.117187688 | Up | 9.58E-54 |
| 100188893 | 69.6887263 | 151.1531214 | 1.117013581 | Up | 2.58E-77 |
| 2312 | 0.20832259 | 0.451726465 | 1.116630178 | Up | 9.41E-05 |
| 55332 | 4.71745805 | 10.22449027 | 1.115947327 | Up | 7.24E-23 |
| 78992 | 19.2140676 | 41.62955391 | 1.115445132 | Up | 2.56E-38 |
| 83879 | 1.55649286 | 3.372045779 | 1.115325162 | Up | 5.23E-07 |
| 27315 | 7.0471246 | 15.26594379 | 1.115210156 | Up | 9.58E-21 |
| 26585 | 47.9119893 | 103.6195371 | 1.112837424 | Up | 1.45E-250 |
| 51154 | 16.9399806 | 36.62040118 | 1.11221537 | Up | 3.75E-50 |
| 23362 | 0.72174891 | 1.559169728 | 1.111209054 | Up | 3.63E-12 |
| 57231 | 7.15494601 | 15.43463051 | 1.109158161 | Up | 4.87E-33 |
| 64395 | 0.96546251 | 2.081628496 | 1.108420467 | Up | 0.00035573 |
| 79794 | 1.99830046 | 4.307619062 | 1.108117157 | Up | 2.01E-08 |
| 84287 | 7.32460562 | 15.78869941 | 1.108069346 | Up | 1.43E-19 |
| 79441 | 1.57599866 | 3.397104526 | 1.108039306 | Up | 1.37E-12 |
| 113402 | 15.7109403 | 33.85972407 | 1.107800686 | Up | 3.82E-15 |
| 5163 | 3.31116742 | 7.135894618 | 1.107754351 | Up | 1.54E-20 |
| 7922 | 24.8566102 | 53.5225329 | 1.106516829 | Up | 1.78E-77 |
| 8703 | 11.1002652 | 23.89711363 | 1.106242226 | Up | 1.45E-28 |
| 7301 | 2.59089366 | 5.574119035 | 1.105294009 | Up | 2.75E-14 |
| 10765 | 5.13913317 | 11.05368139 | 1.104929991 | Up | 2.43E-42 |
| 6206 | 948.25548 | 2039.5617 | 1.104911443 | Up | 0 |
| 388730 | 2.69723653 | 5.800401685 | 1.10467077 | Up | 7.05E-06 |
| 3426 | 5.9634091 | 12.8169668 | 1.103845666 | Up | 1.46E-17 |
| 253714 | 1.67175504 | 3.5925392 | 1.103640431 | Up | 1.69E-19 |
| 813 | 52.2897973 | 112.3673212 | 1.103621147 | Up | 2.18E-221 |
| 80761 | 2.08726619 | 4.484722547 | 1.103404137 | Up | 1.45E-05 |
| 84326 | 57.2752314 | 123.0419979 | 1.103167548 | Up | 4.63E-61 |
| 537 | 33.5256941 | 72.01993798 | 1.103129154 | Up | 1.84E-88 |
| 51608 | 17.8049962 | 38.22434519 | 1.102209663 | Up | 1.73E-47 |
| 400745 | 0.61113136 | 1.311757064 | 1.101946133 | Up | 0.00030909 |
| 57446 | 8.01052187 | 17.18918976 | 1.101533403 | Up | 6.83E-31 |
| 4542 | 2.38839593 | 5.124644505 | 1.101409914 | Up | 8.07E-13 |
| 9616 | 50.5053635 | 108.3520538 | 1.101217989 | Up | 7.82E-101 |
| 2677 | 4.52210859 | 9.697457563 | 1.100610922 | Up | 1.14E-19 |
| 80019 | 9.28001209 | 19.89558654 | 1.100249842 | Up | 2.29E-19 |
| 1012 | 5.43002835 | 11.63999179 | 1.100058406 | Up | 3.76E-27 |
| 4694 | 150.580504 | 322.7471584 | 1.099869396 | Up | 2.73E-91 |
| 9716 | 2.85966169 | 6.126615034 | 1.09924572 | Up | 2.00E-19 |
| 79033 | 35.533765 | 76.1080212 | 1.098857951 | Up | 2.22E-77 |
| 9831 | 3.14649577 | 6.737552361 | 1.098478577 | Up | 3.49E-17 |
| 26064 | 8.00489334 | 17.1396896 | 1.098386897 | Up | 7.04E-54 |
| 55192 | 8.02262235 | 17.17603983 | 1.098251649 | Up | 2.73E-11 |
| 8635 | 20.4316371 | 43.67452942 | 1.095987354 | Up | 9.92E-33 |
| 84365 | 17.0413822 | 36.42305221 | 1.095809466 | Up | 1.17E-37 |
| 55093 | 7.4162695 | 15.84288091 | 1.095069126 | Up | 1.01E-13 |
| 56243 | 4.86504386 | 10.38706647 | 1.094263547 | Up | 2.27E-47 |
| 4221 | 5.16237854 | 11.01849961 | 1.093819947 | Up | 2.11E-21 |
| 3055 | 6.13743386 | 13.09680159 | 1.093507052 | Up | 4.12E-17 |
| 84946 | 16.231905 | 34.63589486 | 1.093435617 | Up | 2.78E-37 |
| 57449 | 4.45787613 | 9.507139102 | 1.092654739 | Up | 9.08E-30 |
| 6688 | 10.3175294 | 22.00101438 | 1.092472491 | Up | 3.65E-19 |
| 7388 | 163.777861 | 349.1975807 | 1.092303214 | Up | 2.04E-105 |
| 741 | 5.30326216 | 11.3043992 | 1.091932344 | Up | 8.14E-10 |
| 10207 | 3.65531869 | 7.791069192 | 1.091824135 | Up | 3.90E-39 |
| 29911 | 7.39131774 | 15.7505247 | 1.091496392 | Up | 6.87E-25 |
| 6892 | 45.2310551 | 96.36359776 | 1.091174607 | Up | 4.20E-196 |
| 5916 | 12.3347679 | 26.27618493 | 1.091025255 | Up | 1.51E-45 |
| 84501 | 1.10749663 | 2.359200671 | 1.090995833 | Up | 9.61E-06 |
| 5796 | 4.63159134 | 9.864461309 | 1.090732303 | Up | 6.01E-36 |
| 5496 | 33.9727192 | 72.30573836 | 1.089733449 | Up | 2.33E-93 |
| 55809 | 1.28797144 | 2.740514372 | 1.089346096 | Up | 2.77E-13 |
| 55240 | 2.93522799 | 6.244145749 | 1.089031646 | Up | 1.12E-16 |
| 3310 | 3.26899973 | 6.953499552 | 1.08888998 | Up | 6.02E-12 |
| 6646 | 1.97961173 | 4.21045123 | 1.088757361 | Up | 1.44E-09 |
| 83595 | 5.65855194 | 12.03469008 | 1.088694178 | Up | 1.96E-23 |
| 26973 | 3.48713507 | 7.413806877 | 1.088172287 | Up | 1.04E-15 |
| 55715 | 5.9357967 | 12.61626168 | 1.087770903 | Up | 3.84E-21 |
| 3608 | 43.5087619 | 92.46656073 | 1.087625765 | Up | 2.55E-85 |
| 126432 | 2.6129054 | 5.552916623 | 1.08759084 | Up | 5.70E-11 |
| 55284 | 2.86189231 | 6.081524732 | 1.087463687 | Up | 2.10E-15 |
| 3336 | 51.0292121 | 108.4133425 | 1.087147051 | Up | 1.36E-59 |
| 23507 | 1.34313999 | 2.853036482 | 1.086888513 | Up | 4.54E-14 |
| 4747 | 1.74190328 | 3.69825149 | 1.086178817 | Up | 5.95E-09 |
| 51466 | 23.8293456 | 50.57054773 | 1.085558072 | Up | 7.78E-54 |
| 10300 | 3.45351581 | 7.32737176 | 1.085229987 | Up | 1.99E-12 |
| 11047 | 70.54248 | 149.666955 | 1.085191521 | Up | 3.68E-119 |
| 84132 | 5.7215219 | 12.13139922 | 1.084275106 | Up | 2.15E-36 |
| 197335 | 2.04383239 | 4.327770053 | 1.082346955 | Up | 5.72E-15 |
| 6234 | 628.500932 | 1330.684229 | 1.082181471 | Up | 6.35E-284 |
| 10813 | 8.88385549 | 18.80793355 | 1.082083516 | Up | 9.64E-28 |
| 286075 | 3.68988606 | 7.811713159 | 1.082062708 | Up | 1.64E-11 |
| 5989 | 1.92757759 | 4.080798967 | 1.082062708 | Up | 1.64E-11 |
| 25925 | 1.93253236 | 4.090881219 | 1.081919078 | Up | 6.58E-13 |
| 9907 | 7.4589323 | 15.78821093 | 1.081806662 | Up | 4.43E-27 |
| 8614 | 3.11357876 | 6.588982986 | 1.08148203 | Up | 3.03E-21 |
| 79913 | 3.65465923 | 7.732779942 | 1.081250266 | Up | 3.07E-12 |
| 7329 | 57.387964 | 121.3650416 | 1.080532826 | Up | 3.11E-101 |
| 25966 | 2.79333347 | 5.90270461 | 1.079388333 | Up | 1.92E-22 |
| 57129 | 20.0551561 | 42.37390982 | 1.079203054 | Up | 2.06E-29 |
| 10695 | 21.9926268 | 46.4583993 | 1.078919516 | Up | 3.81E-47 |
| 5134 | 11.4845525 | 24.2485458 | 1.078203583 | Up | 3.61E-29 |
| 51296 | 10.556399 | 22.28666304 | 1.078062837 | Up | 8.69E-28 |
| 56342 | 5.02826531 | 10.61306383 | 1.077708522 | Up | 2.54E-11 |
| 10428 | 28.944978 | 61.09148338 | 1.077658213 | Up | 6.35E-45 |
| 55695 | 14.6701849 | 30.95482562 | 1.077277277 | Up | 1.15E-30 |
| 201254 | 29.5291977 | 62.30431982 | 1.077190034 | Up | 8.00E-27 |
| 3134 | 20.6917276 | 43.62170178 | 1.075991948 | Up | 1.38E-39 |
| 545 | 1.0601733 | 2.234963133 | 1.075950924 | Up | 9.57E-12 |
| 51534 | 6.23568753 | 13.14411803 | 1.075796798 | Up | 3.38E-25 |
| 10468 | 10.3984426 | 21.91440769 | 1.075512215 | Up | 3.71E-18 |
| 5699 | 25.36938 | 53.45621217 | 1.07526935 | Up | 1.55E-30 |
| 79871 | 3.21244579 | 6.768846788 | 1.075237954 | Up | 3.36E-13 |
| 389289 | 2.16094118 | 4.551902964 | 1.074810003 | Up | 0.0001527 |
| 94097 | 2.13178562 | 4.489482354 | 1.074486746 | Up | 8.36E-12 |
| 387338 | 4.66573171 | 9.825758282 | 1.074465396 | Up | 1.13E-25 |
| 3281 | 38.9893407 | 82.10025465 | 1.074306937 | Up | 4.55E-87 |
| 5862 | 26.8052437 | 56.44380756 | 1.074300061 | Up | 2.31E-37 |
| 10410 | 1097.2946 | 2309.613327 | 1.073700432 | Up | 0 |
| 8202 | 5.05062747 | 10.63046677 | 1.073670405 | Up | 4.54E-48 |
| 4837 | 58.5521938 | 123.2353367 | 1.073620864 | Up | 1.79E-108 |
| 57026 | 7.79078187 | 16.39527081 | 1.073439705 | Up | 1.02E-19 |
| 10248 | 17.4306205 | 36.67865147 | 1.073316666 | Up | 1.93E-20 |
| 84759 | 15.6032765 | 32.82376502 | 1.072891726 | Up | 5.71E-18 |
| 10629 | 4.72879194 | 9.947502866 | 1.072862742 | Up | 1.37E-12 |
| 7481 | 2.55343179 | 5.371097787 | 1.072779467 | Up | 3.70E-07 |
| 2643 | 1.68220959 | 3.538497585 | 1.072779467 | Up | 3.70E-07 |
| 154761 | 2.31804278 | 4.874587745 | 1.072373017 | Up | 2.90E-08 |
| 64223 | 18.0493176 | 37.95512729 | 1.072350493 | Up | 7.40E-36 |
| 51645 | 13.7377678 | 28.87779795 | 1.071813128 | Up | 3.82E-29 |
| 51010 | 6.40185149 | 13.44247127 | 1.070237273 | Up | 1.60E-10 |
| 9052 | 2.19158438 | 4.601842147 | 1.070237273 | Up | 1.08E-08 |
| 440603 | 0.87949828 | 1.846751734 | 1.070237273 | Up | 1.78E-06 |
| 97 | 4.55408678 | 9.562574316 | 1.070237273 | Up | 5.56E-05 |
| 6731 | 26.1953985 | 54.98573434 | 1.069743959 | Up | 5.18E-118 |
| 84626 | 3.36196759 | 7.054853906 | 1.069310389 | Up | 4.05E-17 |
| 5330 | 2.08741149 | 4.379206045 | 1.068954307 | Up | 9.11E-13 |
| 1810 | 6.01350975 | 12.60999903 | 1.068289001 | Up | 9.85E-24 |
| 338707 | 1.65116969 | 3.461813756 | 1.068039717 | Up | 5.14E-08 |
| 4582 | 18.2391365 | 38.2373593 | 1.067945461 | Up | 1.13E-26 |
| 84246 | 20.9691774 | 43.95972879 | 1.06791222 | Up | 2.59E-26 |
| 116988 | 18.4623692 | 38.6992333 | 1.067717281 | Up | 8.07E-70 |
| 8480 | 10.3796468 | 21.75526166 | 1.067607017 | Up | 1.97E-23 |
| 116225 | 9.97660798 | 20.90876651 | 1.067486662 | Up | 3.10E-17 |
| 27242 | 9.54252867 | 19.99708372 | 1.0673461 | Up | 2.81E-41 |
| 23649 | 3.46842338 | 7.268208484 | 1.06731979 | Up | 2.27E-11 |
| 286053 | 13.9192731 | 29.16725431 | 1.067265706 | Up | 6.60E-21 |
| 6309 | 4.98503484 | 10.43631668 | 1.065937135 | Up | 1.65E-41 |
| 414918 | 3.10106703 | 6.490882832 | 1.065650003 | Up | 1.05E-07 |
| 90379 | 7.71340885 | 16.13996157 | 1.065196656 | Up | 1.92E-21 |
| 57418 | 12.286932 | 25.70584935 | 1.064971957 | Up | 2.65E-23 |
| 51351 | 2.17087144 | 4.541277095 | 1.064823777 | Up | 2.07E-17 |
| 114049 | 28.1296314 | 58.84378048 | 1.06479929 | Up | 5.76E-41 |
| 10588 | 9.52040579 | 19.90847865 | 1.064288007 | Up | 4.01E-11 |
| 4507 | 4.55505849 | 9.523882692 | 1.064080272 | Up | 4.43E-27 |
| 10042 | 10.7873457 | 22.55242527 | 1.063942663 | Up | 1.27E-53 |
| 3428 | 40.5653531 | 84.80502538 | 1.063901714 | Up | 4.29E-127 |
| 85025 | 10.8376298 | 22.65213992 | 1.063598076 | Up | 2.83E-12 |
| 4893 | 5.7951035 | 12.10716369 | 1.062954597 | Up | 6.56E-31 |
| 3304 | 40.8179003 | 85.26746821 | 1.06279345 | Up | 9.91E-118 |
| 376267 | 3.47787992 | 7.265162625 | 1.062786968 | Up | 6.19E-15 |
| 664 | 19.6529329 | 41.0529918 | 1.062742736 | Up | 9.35E-36 |
| 25807 | 5.66221295 | 11.82648719 | 1.0625837 | Up | 1.07E-12 |
| 58516 | 7.07969459 | 14.78378976 | 1.062257114 | Up | 1.55E-26 |
| 57620 | 3.61641557 | 7.55171832 | 1.062244482 | Up | 1.38E-17 |
| 58986 | 8.90830943 | 18.60081127 | 1.062141969 | Up | 1.92E-38 |
| 1512 | 43.2325438 | 90.26340351 | 1.062023452 | Up | 4.61E-74 |
| 79074 | 6.69199178 | 13.96952796 | 1.061775694 | Up | 3.41E-08 |
| 55686 | 2.26067249 | 4.718555189 | 1.061593178 | Up | 1.01E-09 |
| 221504 | 3.16904637 | 6.61396693 | 1.061467063 | Up | 3.06E-11 |
| 7150 | 29.3747747 | 61.29001891 | 1.061074365 | Up | 6.58E-125 |
| 114882 | 3.67808073 | 7.670308885 | 1.06033153 | Up | 1.28E-31 |
| 343099 | 1.00647665 | 2.097723562 | 1.059510866 | Up | 1.02E-06 |
| 80271 | 7.96023425 | 16.58015101 | 1.058574355 | Up | 7.49E-32 |
| 57804 | 39.1363732 | 81.46313309 | 1.057637236 | Up | 7.92E-42 |
| 93663 | 3.10267498 | 6.457645213 | 1.057495604 | Up | 1.02E-13 |
| 4084 | 6.52952179 | 13.58332925 | 1.056787885 | Up | 2.32E-42 |
| 392617 | 0.73051592 | 1.519312983 | 1.056431473 | Up | 0.00020734 |
| 3071 | 2.02629371 | 4.212641909 | 1.05588198 | Up | 2.52E-10 |
| 23583 | 6.56658703 | 13.64910289 | 1.055590497 | Up | 4.73E-13 |
| 54542 | 1.89825885 | 3.944831459 | 1.055286931 | Up | 5.86E-10 |
| 3417 | 10.429328 | 21.65673765 | 1.054169729 | Up | 1.32E-28 |
| 728215 | 0.80977282 | 1.681450989 | 1.054117608 | Up | 7.53E-05 |
| 51124 | 19.4597266 | 40.40587614 | 1.054073677 | Up | 1.25E-33 |
| 7171 | 185.64498 | 385.108026 | 1.052716886 | Up | 0 |
| 26262 | 6.91093227 | 14.33540411 | 1.052630329 | Up | 2.06E-21 |
| 55238 | 3.80232579 | 7.885474192 | 1.052315365 | Up | 3.14E-13 |
| 6176 | 3111.44237 | 6451.875117 | 1.05213499 | Up | 0 |
| 7286 | 2.7364777 | 5.669852807 | 1.050991184 | Up | 6.30E-11 |
| 79023 | 6.48154259 | 13.42834087 | 1.050871948 | Up | 3.88E-10 |
| 598 | 21.0898353 | 43.66448061 | 1.049912353 | Up | 6.34E-61 |
| 28232 | 7.43397138 | 15.38843236 | 1.049641231 | Up | 2.42E-43 |
| 11313 | 16.543898 | 34.24482432 | 1.049586764 | Up | 3.65E-31 |
| 1386 | 1.70190386 | 3.521829833 | 1.049175657 | Up | 2.39E-05 |
| 23086 | 0.88169212 | 1.824527039 | 1.049175657 | Up | 2.07E-11 |
| 9238 | 13.1387532 | 27.18299942 | 1.048876281 | Up | 4.98E-34 |
| 3123 | 62.1468663 | 128.5707953 | 1.048809421 | Up | 6.48E-81 |
| 29960 | 12.6349596 | 26.13226118 | 1.04841091 | Up | 8.40E-24 |
| 56943 | 46.3948591 | 95.94580276 | 1.048254744 | Up | 1.46E-30 |
| 91351 | 1.60738226 | 3.323885767 | 1.048157739 | Up | 1.82E-13 |
| 81572 | 11.1493862 | 23.03963645 | 1.04715366 | Up | 2.43E-18 |
| 4012 | 0.57659629 | 1.190342025 | 1.045742711 | Up | 0.000374 |
| 51241 | 12.2719604 | 25.33200908 | 1.045595767 | Up | 1.63E-24 |
| 3303 | 73.5045355 | 151.7176056 | 1.045483331 | Up | 3.00E-195 |
| 57414 | 25.8262552 | 53.28824028 | 1.044978723 | Up | 2.41E-54 |
| 1647 | 39.0801143 | 80.63410451 | 1.044955476 | Up | 3.62E-59 |
| 6210 | 1464.53141 | 3020.894909 | 1.044536865 | Up | 0 |
| 282991 | 7.94917647 | 16.3854102 | 1.043534479 | Up | 4.69E-19 |
| 203068 | 303.099694 | 624.3774359 | 1.042626006 | Up | 0 |
| 6184 | 62.0216623 | 127.6864419 | 1.041761245 | Up | 7.69E-158 |
| 83690 | 0.63346565 | 1.304056532 | 1.041668121 | Up | 0.00032392 |
| 56951 | 13.9762526 | 28.77160518 | 1.041668121 | Up | 4.92E-36 |
| 8607 | 10.7407634 | 22.10979265 | 1.041588958 | Up | 4.13E-22 |
| 643837 | 3.69506736 | 7.605300518 | 1.041404591 | Up | 1.25E-07 |
| 2524 | 2.09180859 | 4.304135579 | 1.040972678 | Up | 1.74E-08 |
| 10452 | 22.6679543 | 46.63703697 | 1.040821933 | Up | 1.25E-45 |
| 78991 | 2.69424844 | 5.543036022 | 1.040793495 | Up | 6.52E-09 |
| 56052 | 2.82059194 | 5.802127365 | 1.040583999 | Up | 1.62E-13 |
| 118980 | 1.49376535 | 3.072564862 | 1.04048993 | Up | 1.56E-05 |
| 8625 | 10.9169821 | 22.45439043 | 1.040423469 | Up | 1.64E-18 |
| 6526 | 0.72866962 | 1.498562527 | 1.040242533 | Up | 1.30E-10 |
| 81873 | 18.7446212 | 38.54178733 | 1.039946807 | Up | 2.75E-22 |
| 57001 | 2.61987292 | 5.385992271 | 1.039715328 | Up | 2.92E-07 |
| 80307 | 0.81913339 | 1.683992415 | 1.039715328 | Up | 2.92E-07 |
| 4831 | 55.3939281 | 113.854352 | 1.039389685 | Up | 1.66E-50 |
| 84962 | 1.55024041 | 3.186269446 | 1.039376309 | Up | 1.52E-08 |
| 23556 | 1.50597811 | 3.094939437 | 1.039210377 | Up | 2.13E-09 |
| 57038 | 16.0385435 | 32.96085146 | 1.039210377 | Up | 3.66E-33 |
| 80781 | 27.1429577 | 55.77627274 | 1.039073594 | Up | 4.73E-193 |
| 8508 | 10.8370193 | 22.2496678 | 1.037815795 | Up | 1.87E-25 |
| 26519 | 26.0728225 | 53.53055326 | 1.037815795 | Up | 2.00E-20 |
| 29071 | 7.49103843 | 15.37203363 | 1.037070409 | Up | 2.78E-16 |
| 11282 | 12.7569355 | 26.17138706 | 1.036708585 | Up | 3.81E-41 |
| 3437 | 17.5200611 | 35.9420493 | 1.036664862 | Up | 3.99E-49 |
| 9180 | 3.78974846 | 7.773793299 | 1.036516655 | Up | 1.91E-18 |
| 57560 | 1.83792204 | 3.770072403 | 1.036516655 | Up | 6.07E-10 |
| 29109 | 7.17392741 | 14.71378197 | 1.036333066 | Up | 3.98E-31 |
| 29982 | 8.77418317 | 17.99135817 | 1.035967371 | Up | 2.42E-19 |
| 10956 | 63.0640227 | 129.2681016 | 1.035477213 | Up | 6.64E-183 |
| 84628 | 1.03597739 | 2.122266574 | 1.034613363 | Up | 8.81E-05 |
| 85352 | 1.25130982 | 2.562597797 | 1.034168018 | Up | 1.73E-10 |
| 7090 | 5.5086425 | 11.2798077 | 1.033973729 | Up | 8.62E-33 |
| 125488 | 3.54808475 | 7.262372722 | 1.033400505 | Up | 2.57E-21 |
| 127544 | 11.8819151 | 24.305356 | 1.032506884 | Up | 2.45E-34 |
| 8519 | 231.039874 | 472.6033765 | 1.032488076 | Up | 4.27E-176 |
| 64768 | 1.57944339 | 3.229532597 | 1.031909154 | Up | 7.60E-09 |
| 10330 | 25.4527872 | 52.02845438 | 1.031477201 | Up | 4.27E-34 |
| 56648 | 2.45437066 | 5.015415084 | 1.031015963 | Up | 6.48E-16 |
| 26986 | 253.642252 | 518.0404639 | 1.030269702 | Up | 0 |
| 2629 | 8.65767037 | 17.67242974 | 1.029449628 | Up | 7.70E-25 |
| 23762 | 0.77382749 | 1.578681624 | 1.028636368 | Up | 6.63E-05 |
| 57154 | 6.07177172 | 12.37458196 | 1.027190334 | Up | 5.11E-38 |
| 114990 | 6.37401736 | 12.98789495 | 1.026892768 | Up | 2.74E-20 |
| 81614 | 9.48171499 | 19.31391955 | 1.026421041 | Up | 4.81E-27 |
| 81555 | 9.55618795 | 19.46358632 | 1.026270429 | Up | 5.45E-36 |
| 85013 | 3.96826083 | 8.079970415 | 1.025843154 | Up | 1.17E-08 |
| 2585 | 1.33904063 | 2.726486276 | 1.025843154 | Up | 6.71E-06 |
| 81788 | 4.82256499 | 9.815564374 | 1.025270542 | Up | 1.14E-18 |
| 8932 | 30.104558 | 61.26659456 | 1.025118727 | Up | 2.39E-82 |
| 9644 | 6.73597498 | 13.70013092 | 1.024230996 | Up | 4.35E-80 |
| 219743 | 3.32788514 | 6.767355986 | 1.023986642 | Up | 1.05E-14 |
| 140809 | 8.67806536 | 17.64658451 | 1.023943621 | Up | 1.04E-24 |
| 5987 | 11.4694269 | 23.32221729 | 1.02391165 | Up | 6.16E-37 |
| 7534 | 194.797943 | 396.0346703 | 1.023648294 | Up | 0 |
| 126792 | 5.06324086 | 10.28578015 | 1.022518204 | Up | 3.31E-16 |
| 54602 | 1.14263683 | 2.320876471 | 1.022302796 | Up | 6.03E-07 |
| 23464 | 5.46022207 | 11.09057625 | 1.022302796 | Up | 9.42E-10 |
| 6175 | 987.589927 | 2005.402126 | 1.02190753 | Up | 0 |
| 10320 | 0.71943237 | 1.460588274 | 1.021618568 | Up | 5.06E-06 |
| 865 | 6.98012279 | 14.16815591 | 1.021327672 | Up | 3.61E-24 |
| 54739 | 22.4566493 | 45.57515413 | 1.021104855 | Up | 2.71E-85 |
| 102 | 2.62529708 | 5.326935338 | 1.020825079 | Up | 3.58E-12 |
| 10961 | 31.4108235 | 63.7275418 | 1.020655244 | Up | 3.48E-49 |
| 9538 | 52.8513755 | 107.2205701 | 1.020568787 | Up | 3.39E-124 |
| 6199 | 21.9746265 | 44.55643384 | 1.019795123 | Up | 1.43E-41 |
| 51633 | 1.51563543 | 3.071995524 | 1.019253344 | Up | 1.41E-06 |
| 6873 | 2.17815983 | 4.413938686 | 1.018956768 | Up | 8.96E-13 |
| 79415 | 15.056362 | 30.4953824 | 1.018217588 | Up | 1.75E-34 |
| 11194 | 4.21950776 | 8.544853402 | 1.017981036 | Up | 7.82E-22 |
| 5998 | 16.4166843 | 33.22908366 | 1.017283736 | Up | 1.89E-78 |
| 8694 | 9.48614432 | 19.20074301 | 1.017268418 | Up | 1.62E-37 |
| 4725 | 280.007714 | 566.2732364 | 1.016031775 | Up | 8.46E-147 |
| 55103 | 0.88402861 | 1.787513892 | 1.015789489 | Up | 1.07E-06 |
| 23607 | 3.29217787 | 6.656813612 | 1.015789489 | Up | 9.76E-20 |
| 23186 | 3.41053157 | 6.893924749 | 1.015328932 | Up | 9.39E-21 |
| 11108 | 4.96185538 | 10.02711399 | 1.014954837 | Up | 1.00E-22 |
| 347734 | 13.2085259 | 26.68989492 | 1.014824153 | Up | 5.40E-29 |
| 5725 | 31.4933631 | 63.57989826 | 1.013522882 | Up | 6.01E-106 |
| 3987 | 5.35648058 | 10.80704839 | 1.012615241 | Up | 8.27E-10 |
| 84632 | 4.6162244 | 9.312660402 | 1.012480012 | Up | 3.75E-20 |
| 85460 | 1.88049773 | 3.793626296 | 1.012463006 | Up | 9.33E-15 |
| 6307 | 4.52768627 | 9.132305661 | 1.012205152 | Up | 2.54E-11 |
| 206358 | 0.93349707 | 1.881226728 | 1.010956335 | Up | 6.99E-07 |
| 1952 | 2.00073044 | 4.031664397 | 1.010848748 | Up | 7.67E-23 |
| 8624 | 23.2646007 | 46.84738314 | 1.009832034 | Up | 5.67E-28 |
| 55100 | 13.5446524 | 27.27291459 | 1.009745513 | Up | 1.78E-30 |
| 10043 | 14.320963 | 28.8113011 | 1.008506306 | Up | 2.99E-36 |
| 149473 | 4.81124773 | 9.67849753 | 1.008372017 | Up | 1.57E-08 |
| 79760 | 5.83279803 | 11.72758811 | 1.007646316 | Up | 3.87E-11 |
| 440295 | 1.85703456 | 3.732722196 | 1.007227475 | Up | 1.26E-09 |
| 114991 | 1.44840748 | 2.911363567 | 1.007227475 | Up | 9.40E-15 |
| 3073 | 23.1391336 | 46.50838873 | 1.00715611 | Up | 8.06E-58 |
| 92610 | 1.16912433 | 2.348167452 | 1.006106935 | Up | 5.79E-05 |
| 221 | 2.7082859 | 5.437883767 | 1.005665272 | Up | 3.36E-09 |
| 10907 | 20.4970022 | 41.14733672 | 1.005386129 | Up | 7.10E-30 |
| 79007 | 3.41171475 | 6.847164533 | 1.00500965 | Up | 1.03E-08 |
| 54677 | 3.29348264 | 6.608225302 | 1.004648931 | Up | 2.10E-12 |
| 55062 | 13.2741925 | 26.63217813 | 1.004546325 | Up | 5.93E-27 |
| 8795 | 8.67179508 | 17.39693494 | 1.004430578 | Up | 2.58E-37 |
| 10867 | 7.27630138 | 14.59450508 | 1.004148082 | Up | 9.86E-33 |
| 6881 | 75.0276832 | 150.4631608 | 1.003915389 | Up | 3.61E-58 |
| 1662 | 9.54559994 | 19.14139758 | 1.003788391 | Up | 8.85E-32 |
| 54680 | 6.98092494 | 13.99388333 | 1.003306265 | Up | 6.37E-21 |
| 1973 | 224.904958 | 450.8071572 | 1.003194952 | Up | 0 |
| 7374 | 9.23305791 | 18.5045285 | 1.002997935 | Up | 1.92E-20 |
| 1798 | 12.141999 | 24.33005019 | 1.002733336 | Up | 2.61E-27 |
| 5552 | 55.8676409 | 111.8895757 | 1.001990827 | Up | 1.45E-70 |
| 116150 | 7.55517028 | 15.11741258 | 1.00067506 | Up | 2.60E-37 |
| 10124 | 4.47218237 | 8.946716161 | 1.000379227 | Up | 1.80E-15 |
| 9607 | 30.4496875 | 0 | -14.8941398 | Down | 1.04E-112 |
| 4618 | 27.764444 | 0 | -14.76095089 | Down | 3.09E-146 |
| 246705 | 21.1667392 | 0 | -14.36951141 | Down | 1.02E-50 |
| 128826 | 19.269573 | 0 | -14.23403698 | Down | 6.03E-96 |
| 201140 | 18.9315604 | 0 | -14.20850571 | Down | 2.46E-76 |
| 131377 | 18.629679 | 0 | -14.18531519 | Down | 3.04E-183 |
| 57467 | 16.2574057 | 0 | -13.98880944 | Down | 1.83E-123 |
| 57408 | 14.0058674 | 0 | -13.77374372 | Down | 3.58E-73 |
| 27231 | 13.3870333 | 0 | -13.70854866 | Down | 8.71E-62 |
| 492307 | 12.6569412 | 0 | -13.62764117 | Down | 1.39E-65 |
| 146713 | 10.6410768 | 0 | -13.37735653 | Down | 3.45E-116 |
| 786 | 10.5660938 | 0 | -13.3671545 | Down | 7.92E-55 |
| 284485 | 10.4708964 | 0 | -13.35409734 | Down | 4.91E-20 |
| 22953 | 10.438239 | 0 | -13.34959072 | Down | 2.14E-81 |
| 419 | 9.588931 | 0 | -13.22715427 | Down | 8.71E-62 |
| 779 | 9.00307072 | 0 | -13.13620144 | Down | 1.26E-225 |
| 859 | 7.52994693 | 0 | -12.87842398 | Down | 4.49E-44 |
| 118461 | 7.0478499 | 0 | -12.78296748 | Down | 1.30E-151 |
| 5126 | 5.97623093 | 0 | -12.54502018 | Down | 1.48E-115 |
| 92235 | 5.65343474 | 0 | -12.46491193 | Down | 4.24E-93 |
| 253017 | 5.45549084 | 0 | -12.41349329 | Down | 9.80E-63 |
| 8788 | 4.79532547 | 0 | -12.22741302 | Down | 1.79E-30 |
| 6779 | 4.68615529 | 0 | -12.19418905 | Down | 3.96E-12 |
| 255275 | 4.46118415 | 0 | -12.12321099 | Down | 5.05E-45 |
| 10777 | 4.12080919 | 0 | -12.00871195 | Down | 7.92E-55 |
| 6750 | 4.07814147 | 0 | -11.99369611 | Down | 3.52E-11 |
| 4626 | 3.9710988 | 0 | -11.95532254 | Down | 7.63E-98 |
| 146862 | 3.86455842 | 0 | -11.91608786 | Down | 1.28E-89 |
| 245806 | 3.84863568 | 0 | -11.91013139 | Down | 3.23E-35 |
| 83869 | 3.79138778 | 0 | -11.88851031 | Down | 1.20E-08 |
| 144453 | 3.56016894 | 0 | -11.79772999 | Down | 1.15E-51 |
| 3596 | 3.53350243 | 0 | -11.78688319 | Down | 4.36E-19 |
| 5506 | 3.34517603 | 0 | -11.70786642 | Down | 6.12E-59 |
| 136371 | 3.27110116 | 0 | -11.67556066 | Down | 2.32E-26 |
| 6456 | 3.18025946 | 0 | -11.63492876 | Down | 7.52E-36 |
| 51209 | 2.97817305 | 0 | -11.54021187 | Down | 2.15E-13 |
| 56704 | 2.87548976 | 0 | -11.48959198 | Down | 1.15E-51 |
| 885 | 2.73217739 | 0 | -11.41583544 | Down | 6.49E-10 |
| 5913 | 2.62845249 | 0 | -11.35999794 | Down | 1.87E-18 |
| 118421 | 2.60641549 | 0 | -11.34785137 | Down | 6.49E-10 |
| 4624 | 2.52580376 | 0 | -11.30252684 | Down | 1.80E-61 |
| 89792 | 2.49363685 | 0 | -11.28403567 | Down | 2.87E-34 |
| 59285 | 2.4908393 | 0 | -11.28241623 | Down | 2.11E-19 |
| 58157 | 2.41468556 | 0 | -11.23761962 | Down | 4.36E-19 |
| 398 | 2.40443212 | 0 | -11.23148048 | Down | 6.49E-10 |
| 377047 | 2.39426539 | 0 | -11.22536736 | Down | 6.49E-10 |
| 441549 | 2.29022627 | 0 | -11.16127443 | Down | 4.46E-13 |
| 4888 | 2.27949182 | 0 | -11.15449652 | Down | 9.04E-19 |
| 340359 | 2.21446815 | 0 | -11.11274453 | Down | 3.45E-17 |
| 143379 | 2.12149888 | 0 | -11.0508682 | Down | 2.78E-09 |
| 266743 | 2.1051273 | 0 | -11.03969176 | Down | 6.83E-29 |
| 254956 | 2.10489245 | 0 | -11.0395308 | Down | 9.44E-07 |
| 142685 | 2.07811765 | 0 | -11.02106162 | Down | 1.63E-23 |
| 285525 | 2.00049319 | 0 | -10.96614 | Down | 2.48E-08 |
| 653316 | 1.98822305 | 0 | -10.9572639 | Down | 2.72E-15 |
| 5207 | 1.93791765 | 0 | -10.92029155 | Down | 2.42E-14 |
| 84651 | 1.84942376 | 0 | -10.85286012 | Down | 7.46E-05 |
| 8528 | 1.82803015 | 0 | -10.83607415 | Down | 2.15E-13 |
| 3158 | 1.79502281 | 0 | -10.80978646 | Down | 1.32E-15 |
| 54810 | 1.77065733 | 0 | -10.79006932 | Down | 5.52E-21 |
| 730112 | 1.7570886 | 0 | -10.77897122 | Down | 4.56E-07 |
| 338872 | 1.75367257 | 0 | -10.77616369 | Down | 1.04E-13 |
| 4654 | 1.68660074 | 0 | -10.71990278 | Down | 4.46E-13 |
| 57348 | 1.65520517 | 0 | -10.69279434 | Down | 2.42E-14 |
| 169693 | 1.57084852 | 0 | -10.61732835 | Down | 1.20E-08 |
| 3745 | 1.55955647 | 0 | -10.60692008 | Down | 1.83E-24 |
| 145814 | 1.49140621 | 0 | -10.54245754 | Down | 9.44E-07 |
| 23237 | 1.37999816 | 0 | -10.43045063 | Down | 3.45E-17 |
| 10590 | 1.3730002 | 0 | -10.42311612 | Down | 5.77E-09 |
| 1144 | 1.34582289 | 0 | -10.39427285 | Down | 3.13E-10 |
| 389257 | 1.34060387 | 0 | -10.38866728 | Down | 2.78E-09 |
| 29119 | 1.32254999 | 0 | -10.36910653 | Down | 3.88E-18 |
| 137735 | 1.30454577 | 0 | -10.34933185 | Down | 2.72E-15 |
| 50632 | 1.27125271 | 0 | -10.31203513 | Down | 8.40E-06 |
| 8048 | 133.432535 | 0.108707034 | -10.26144947 | Down | 0 |
| 401 | 1.19521324 | 0 | -10.22305232 | Down | 5.77E-09 |
| 22866 | 1.19022536 | 0 | -10.21701905 | Down | 1.48E-16 |
| 10814 | 1.1733567 | 0 | -10.19642594 | Down | 3.37E-23 |
| 343263 | 1.13852169 | 0 | -10.15294607 | Down | 4.56E-07 |
| 57628 | 1.12424042 | 0 | -10.13473488 | Down | 6.99E-23 |
| 53828 | 1.11430411 | 0 | -10.12192731 | Down | 0.00032026 |
| 139411 | 1.09234389 | 0 | -10.0932114 | Down | 3.79E-24 |
| 4604 | 281.772549 | 0.259256334 | -10.08593612 | Down | 0 |
| 257019 | 1.05292942 | 0 | -10.04019302 | Down | 1.70E-11 |
| 345557 | 1.04957607 | 0 | -10.03559101 | Down | 1.23E-33 |
| 203430 | 0.97333286 | 0 | -9.92678945 | Down | 3.52E-11 |
| 57582 | 0.96665675 | 0 | -9.916859877 | Down | 2.11E-19 |
| 33 | 0.94099504 | 0 | -9.878043306 | Down | 3.13E-10 |
| 1180 | 0.90905049 | 0 | -9.828216621 | Down | 3.96E-12 |
| 129446 | 20.6691106 | 0.023135396 | -9.803158775 | Down | 0 |
| 405754 | 0.88085626 | 0 | -9.782762803 | Down | 3.96E-12 |
| 90586 | 0.86655067 | 0 | -9.759140293 | Down | 5.13E-08 |
| 26254 | 0.86540264 | 0 | -9.757227711 | Down | 8.40E-06 |
| 946 | 0.86529842 | 0 | -9.757053965 | Down | 5.13E-08 |
| 55118 | 0.86510309 | 0 | -9.756728249 | Down | 7.30E-11 |
| 279 | 0.86142561 | 0 | -9.750582397 | Down | 1.96E-06 |
| 123103 | 0.86016066 | 0 | -9.748462345 | Down | 1.74E-05 |
| 129807 | 0.84820361 | 0 | -9.728266809 | Down | 1.20E-08 |
| 29895 | 91.934497 | 0.111279491 | -9.690274778 | Down | 5.31E-242 |
| 92737 | 0.81157946 | 0 | -9.664588546 | Down | 1.70E-11 |
| 112885 | 0.80913259 | 0 | -9.660232328 | Down | 9.24E-13 |
| 7761 | 0.79039351 | 0 | -9.626427289 | Down | 2.78E-09 |
| 90485 | 0.79032381 | 0 | -9.626300061 | Down | 4.05E-06 |
| 442721 | 53.6069417 | 0.067834403 | -9.626186968 | Down | 0 |
| 9362 | 0.77714018 | 0 | -9.602031047 | Down | 4.56E-07 |
| 203190 | 0.76524275 | 0 | -9.579773662 | Down | 7.30E-11 |
| 10747 | 0.73500799 | 0 | -9.521616124 | Down | 5.13E-08 |
| 100130613 | 0.7001862 | 0 | -9.451594823 | Down | 0.00032026 |
| 554 | 0.67719007 | 0 | -9.403417011 | Down | 1.74E-05 |
| 6869 | 0.67019802 | 0 | -9.388443604 | Down | 1.04E-13 |
| 4632 | 364.825208 | 0.554281874 | -9.362369871 | Down | 0 |
| 116135 | 0.64662107 | 0 | -9.336776715 | Down | 3.60E-05 |
| 157753 | 0.63619741 | 0 | -9.313330694 | Down | 4.05E-06 |
| 257240 | 0.63252096 | 0 | -9.304969479 | Down | 5.13E-08 |
| 7134 | 2065.27966 | 3.355997304 | -9.265379881 | Down | 0 |
| 84457 | 0.61119968 | 0 | -9.255499988 | Down | 1.34E-09 |
| 130752 | 0.60336763 | 0 | -9.2368935 | Down | 1.96E-06 |
| 1749 | 0.60332056 | 0 | -9.236780925 | Down | 0.00032026 |
| 4633 | 2331.96051 | 3.868606663 | -9.235513591 | Down | 0 |
| 58 | 2839.96087 | 4.738701371 | -9.227163589 | Down | 0 |
| 124857 | 0.58254461 | 0 | -9.186224725 | Down | 1.34E-09 |
| 1310 | 0.58185309 | 0 | -9.184511135 | Down | 2.66E-21 |
| 192666 | 0.58130581 | 0 | -9.183153526 | Down | 3.60E-05 |
| 135138 | 0.57853769 | 0 | -9.176267141 | Down | 0.0001546 |
| 219736 | 0.57768187 | 0 | -9.174131396 | Down | 1.20E-08 |
| 55363 | 0.5711341 | 0 | -9.157685715 | Down | 8.40E-06 |
| 57795 | 0.5707328 | 0 | -9.156671661 | Down | 5.77E-09 |
| 2103 | 0.57047043 | 0 | -9.156008302 | Down | 1.06E-07 |
| 339398 | 0.5694356 | 0 | -9.153388883 | Down | 9.44E-07 |
| 5081 | 0.56783111 | 0 | -9.149318084 | Down | 1.17E-14 |
| 8557 | 968.001205 | 1.742646079 | -9.117585437 | Down | 0 |
| 8929 | 0.54130507 | 0 | -9.080298082 | Down | 2.20E-07 |
| 121952 | 0.54050234 | 0 | -9.078157055 | Down | 8.40E-06 |
| 388531 | 0.53975527 | 0 | -9.076161626 | Down | 4.56E-07 |
| 4620 | 19.1470689 | 0.03592905 | -9.057757048 | Down | 0 |
| 1160 | 70.5872356 | 0.133169427 | -9.050000604 | Down | 0 |
| 22844 | 0.52428507 | 0 | -9.034207657 | Down | 3.52E-11 |
| 10321 | 0.52367636 | 0 | -9.032531663 | Down | 3.60E-05 |
| 114786 | 0.51948511 | 0 | -9.020938587 | Down | 5.77E-09 |
| 4892 | 75.4225974 | 0.145564403 | -9.017195432 | Down | 0 |
| 253559 | 0.51583817 | 0 | -9.010774718 | Down | 1.06E-07 |
| 386617 | 0.51244462 | 0 | -9.001252279 | Down | 4.05E-06 |
| 1128 | 0.49799737 | 0 | -8.959994326 | Down | 1.96E-06 |
| 11189 | 0.49746871 | 0 | -8.958461989 | Down | 2.20E-07 |
| 6860 | 0.49576838 | 0 | -8.953522456 | Down | 5.77E-09 |
| 644139 | 0.49574638 | 0 | -8.953458417 | Down | 1.06E-07 |
| 1158 | 2216.96981 | 4.494061901 | -8.94635341 | Down | 0 |
| 100144604 | 0.4875871 | 0 | -8.929516147 | Down | 0.00032026 |
| 1501 | 0.47413271 | 0 | -8.889147116 | Down | 3.52E-11 |
| 4625 | 683.248695 | 1.450508785 | -8.879707956 | Down | 0 |
| 1496 | 0.46755844 | 0 | -8.86900289 | Down | 5.13E-08 |
| 133121 | 0.45650814 | 0 | -8.834496773 | Down | 5.13E-08 |
| 199974 | 0.45051309 | 0 | -8.815425228 | Down | 0.00032026 |
| 29106 | 0.42472707 | 0 | -8.730392237 | Down | 1.96E-06 |
| 361 | 0.42030991 | 0 | -8.715309668 | Down | 1.34E-09 |
| 4842 | 0.41648868 | 0 | -8.702133483 | Down | 9.24E-13 |
| 147658 | 0.41224975 | 0 | -8.687374794 | Down | 0.00032026 |
| 88 | 98.4216803 | 0.241457817 | -8.671061165 | Down | 0 |
| 57818 | 0.40559604 | 0 | -8.663899771 | Down | 8.40E-06 |
| 3359 | 0.40207253 | 0 | -8.651311949 | Down | 0.0001546 |
| 10345 | 136.291198 | 0.342044558 | -8.638292403 | Down | 0 |
| 1339 | 705.214812 | 1.782104314 | -8.628337181 | Down | 0 |
| 3739 | 0.38910783 | 0 | -8.6040262 | Down | 1.96E-06 |
| 9829 | 0.38032565 | 0 | -8.571091419 | Down | 1.34E-09 |
| 170302 | 0.38019341 | 0 | -8.570589699 | Down | 3.60E-05 |
| 2038 | 0.36898861 | 0 | -8.527432457 | Down | 0.0001546 |
| 6532 | 0.36198507 | 0 | -8.499786385 | Down | 2.20E-07 |
| 123624 | 0.35775592 | 0 | -8.48283181 | Down | 8.40E-06 |
| 56100 | 0.35663282 | 0 | -8.478295678 | Down | 2.20E-07 |
| 286753 | 0.34750874 | 0 | -8.440905432 | Down | 8.40E-06 |
| 200150 | 0.34343388 | 0 | -8.423888559 | Down | 0.0001546 |
| 51725 | 4.34054929 | 0.012738215 | -8.412570684 | Down | 4.20E-99 |
| 5502 | 54.1692978 | 0.158970701 | -8.412570684 | Down | 0 |
| 167465 | 0.33835056 | 0 | -8.402374978 | Down | 0.0001546 |
| 338 | 0.33185718 | 0 | -8.374418695 | Down | 1.02E-19 |
| 6329 | 3.08207523 | 0.009338638 | -8.366474269 | Down | 5.92E-96 |
| 58529 | 278.503389 | 0.851385591 | -8.353666493 | Down | 0 |
| 4634 | 693.675409 | 2.196841615 | -8.302686072 | Down | 0 |
| 79981 | 0.30952149 | 0 | -8.273895748 | Down | 0.0001546 |
| 2891 | 0.29861956 | 0 | -8.222164851 | Down | 1.06E-07 |
| 4703 | 42.0219696 | 0.141626477 | -8.212908972 | Down | 0 |
| 270 | 18.3496042 | 0.062270881 | -8.202975537 | Down | 4.32E-170 |
| 761 | 404.847378 | 1.375246537 | -8.201544055 | Down | 0 |
| 6588 | 622.466929 | 2.19662666 | -8.146563691 | Down | 0 |
| 6833 | 0.28241193 | 0 | -8.141657224 | Down | 1.96E-06 |
| 26050 | 0.28107094 | 0 | -8.134790505 | Down | 8.40E-06 |
| 844 | 119.429878 | 0.447166052 | -8.061137423 | Down | 0 |
| 3798 | 0.26054238 | 0 | -8.02537424 | Down | 7.46E-05 |
| 57159 | 21.4704326 | 0.082968772 | -8.015567107 | Down | 5.19E-149 |
| 84700 | 4.32231486 | 0.01701998 | -7.988430977 | Down | 3.51E-146 |
| 84631 | 0.24822616 | 0 | -7.955511356 | Down | 3.60E-05 |
| 56112 | 0.23744544 | 0 | -7.891452219 | Down | 3.60E-05 |
| 1582 | 0.23727369 | 0 | -7.890408301 | Down | 0.0001546 |
| 131096 | 0.23139222 | 0 | -7.854196564 | Down | 1.74E-05 |
| 4547 | 0.22915185 | 0 | -7.840160111 | Down | 0.0001546 |
| 23676 | 113.010743 | 0.493598644 | -7.838905774 | Down | 0 |
| 4151 | 2276.59032 | 9.967598831 | -7.835412974 | Down | 0 |
| 255798 | 8.84587325 | 0.04026965 | -7.779167828 | Down | 1.50E-63 |
| 150572 | 29.3648479 | 0.134170394 | -7.773879997 | Down | 0 |
| 282973 | 0.21671753 | 0 | -7.759672016 | Down | 1.96E-06 |
| 221458 | 0.21629619 | 0 | -7.756864449 | Down | 0.00032026 |
| 148738 | 13.9632618 | 0.065813153 | -7.729044349 | Down | 1.15E-121 |
| 650655 | 0.20514051 | 0 | -7.680468629 | Down | 0.00032026 |
| 6271 | 125.568976 | 0.613535913 | -7.677116557 | Down | 2.11E-291 |
| 56109 | 0.20352466 | 0 | -7.669059797 | Down | 0.0001546 |
| 401145 | 0.20142511 | 0 | -7.654099767 | Down | 8.40E-06 |
| 57144 | 0.19619658 | 0 | -7.616156077 | Down | 0.0001546 |
| 7273 | 25.9500783 | 0.133548543 | -7.602230843 | Down | 0 |
| 157310 | 47.17606 | 0.244590827 | -7.591540825 | Down | 5.70E-165 |
| 7135 | 264.999552 | 1.376365898 | -7.588982057 | Down | 0 |
| 56163 | 0.18351891 | 0 | -7.519784895 | Down | 0.0001546 |
| 487 | 97.8932893 | 0.535941665 | -7.512990176 | Down | 0 |
| 80309 | 0.18076688 | 0 | -7.497986527 | Down | 8.40E-06 |
| 10324 | 201.152202 | 1.12408544 | -7.483392021 | Down | 0 |
| 11155 | 136.849061 | 0.825459416 | -7.373172536 | Down | 0 |
| 2890 | 0.16356563 | 0 | -7.353725815 | Down | 0.0001546 |
| 2819 | 8.14700155 | 0.050687112 | -7.328506419 | Down | 1.54E-91 |
| 5837 | 207.805587 | 1.310211372 | -7.309291055 | Down | 0 |
| 51778 | 24.3655951 | 0.168852664 | -7.172936711 | Down | 2.02E-243 |
| 492 | 0.14256633 | 0 | -7.155489497 | Down | 0.0001546 |
| 26576 | 15.957305 | 0.112250616 | -7.151349848 | Down | 1.89E-120 |
| 7432 | 31.8524444 | 0.230075967 | -7.113149988 | Down | 1.64E-194 |
| 3746 | 0.13801076 | 0 | -7.108636921 | Down | 0.0001546 |
| 6445 | 11.8923578 | 0.088082256 | -7.076967652 | Down | 1.66E-76 |
| 6345 | 23.3343993 | 0.17296646 | -7.075822201 | Down | 0 |
| 51059 | 0.13462095 | 0 | -7.07275914 | Down | 0.0001546 |
| 4653 | 121.641439 | 0.926694243 | -7.036325668 | Down | 0 |
| 1917 | 164.596013 | 1.265555142 | -7.023015214 | Down | 0 |
| 2027 | 360.871906 | 2.871493097 | -6.973541931 | Down | 0 |
| 54715 | 3.76200948 | 0.030529033 | -6.945177779 | Down | 1.26E-69 |
| 2849 | 0.12130086 | 0 | -6.922445913 | Down | 8.40E-06 |
| 4621 | 33.7618135 | 0.278614846 | -6.920976456 | Down | 0 |
| 776 | 0.12065281 | 0 | -6.914717753 | Down | 0.0001546 |
| 9499 | 72.7375491 | 0.609807689 | -6.898202163 | Down | 0 |
| 4656 | 9.1806368 | 0.083587232 | -6.779167828 | Down | 8.11E-62 |
| 2066 | 0.10480931 | 0 | -6.711623074 | Down | 8.40E-06 |
| 29765 | 51.2215915 | 0.496212253 | -6.68965091 | Down | 1.24E-256 |
| 5121 | 538.149308 | 5.243745788 | -6.681264944 | Down | 0 |
| 4852 | 52.3046371 | 0.529132969 | -6.627164735 | Down | 1.09E-109 |
| 4608 | 7.95729581 | 0.080717682 | -6.623249685 | Down | 2.88E-55 |
| 84665 | 4.81695949 | 0.050651888 | -6.571362943 | Down | 2.50E-105 |
| 2662 | 5.05300963 | 0.05451613 | -6.534315956 | Down | 7.68E-52 |
| 5224 | 208.554513 | 2.359685605 | -6.465686069 | Down | 0 |
| 2558 | 5.41272189 | 0.061979648 | -6.448415883 | Down | 9.95E-49 |
| 53353 | 0.08504305 | 0 | -6.41012136 | Down | 1.96E-06 |
| 347730 | 5.50813913 | 0.065902411 | -6.385089947 | Down | 1.49E-46 |
| 27063 | 21.5237123 | 0.258468321 | -6.379795648 | Down | 2.49E-159 |
| 286223 | 2.45766215 | 0.029786705 | -6.366474269 | Down | 6.25E-46 |
| 78986 | 21.3609868 | 0.265208247 | -6.331708851 | Down | 5.22E-132 |
| 252995 | 38.389906 | 0.493784417 | -6.280701909 | Down | 0 |
| 3754 | 17.2651418 | 0.222899286 | -6.275326381 | Down | 1.58E-147 |
| 51207 | 12.7451429 | 0.164625785 | -6.274613411 | Down | 8.33E-85 |
| 8736 | 44.3609878 | 0.573431 | -6.27352778 | Down | 0 |
| 140730 | 2.18979219 | 0.028383203 | -6.26961273 | Down | 7.98E-43 |
| 91977 | 13.1903875 | 0.171232732 | -6.267384629 | Down | 3.52E-188 |
| 116729 | 20.2343376 | 0.27367234 | -6.208212185 | Down | 7.04E-61 |
| 60495 | 9.26467684 | 0.127148829 | -6.18715057 | Down | 1.56E-79 |
| 477 | 57.6427168 | 0.801701922 | -6.16792859 | Down | 0 |
| 827 | 2.93322501 | 0.040856539 | -6.165776919 | Down | 1.01E-39 |
| 7504 | 2.05816063 | 0.028667873 | -6.165776919 | Down | 1.01E-39 |
| 7136 | 98.9228993 | 1.380829405 | -6.162697526 | Down | 4.41E-270 |
| 23281 | 3.57927988 | 0.052520584 | -6.090642589 | Down | 2.74E-92 |
| 5563 | 16.8448375 | 0.249403136 | -6.077683097 | Down | 0 |
| 2104 | 1.78568792 | 0.026451848 | -6.076967652 | Down | 3.04E-37 |
| 2273 | 1183.57333 | 17.89391936 | -6.047535861 | Down | 0 |
| 84676 | 10.6704783 | 0.165279062 | -6.012577065 | Down | 6.20E-70 |
| 284612 | 11.0364599 | 0.173588938 | -5.990458659 | Down | 3.52E-153 |
| 339855 | 4.01193169 | 0.063892064 | -5.972516488 | Down | 1.48E-84 |
| 653 | 2.05059766 | 0.033573499 | -5.932577743 | Down | 2.80E-17 |
| 202333 | 18.9743489 | 0.310955915 | -5.931196512 | Down | 0 |
| 92973 | 1.65288604 | 0.027793352 | -5.894103595 | Down | 1.32E-32 |
| 8854 | 20.893539 | 0.366271691 | -5.833998869 | Down | 4.71E-256 |
| 6900 | 0.54202399 | 0.009544071 | -5.827608183 | Down | 4.81E-16 |
| 8789 | 8.92429881 | 0.167687269 | -5.733893748 | Down | 5.94E-43 |
| 4884 | 13.9627949 | 0.268118692 | -5.702572137 | Down | 1.99E-272 |
| 11075 | 10.7004958 | 0.205898493 | -5.699600571 | Down | 2.72E-28 |
| 56203 | 24.6559525 | 0.480111684 | -5.682422128 | Down | 0 |
| 219537 | 19.8097253 | 0.388830616 | -5.670923246 | Down | 6.14E-147 |
| 364 | 8.7678861 | 0.1740957 | -5.65427658 | Down | 1.71E-40 |
| 1271 | 7.05146037 | 0.14305803 | -5.623249685 | Down | 2.30E-52 |
| 9172 | 13.5600183 | 0.276366646 | -5.616631806 | Down | 1.77E-242 |
| 5166 | 54.0596563 | 1.103170735 | -5.614824344 | Down | 0 |
| 29114 | 4.77322735 | 0.097902037 | -5.607482369 | Down | 1.90E-26 |
| 53405 | 3.56793952 | 0.07318079 | -5.607482369 | Down | 5.37E-77 |
| 266722 | 0.91119686 | 0.018689248 | -5.607482369 | Down | 1.90E-26 |
| 246329 | 127.112794 | 2.617404699 | -5.601828429 | Down | 0 |
| 7125 | 56.9999799 | 1.17734028 | -5.597358151 | Down | 5.92E-139 |
| 22865 | 9.82294513 | 0.207166461 | -5.567293279 | Down | 5.02E-148 |
| 84675 | 2.41074281 | 0.051131579 | -5.559119347 | Down | 1.58E-25 |
| 1996 | 1.6356563 | 0.034692083 | -5.559119347 | Down | 2.84E-13 |
| 80144 | 1.3049597 | 0.027996185 | -5.542631224 | Down | 2.57E-73 |
| 460 | 3.62001332 | 0.079724437 | -5.504829192 | Down | 1.49E-94 |
| 64344 | 26.5189221 | 0.592184697 | -5.484831118 | Down | 0 |
| 1608 | 11.6978885 | 0.263814098 | -5.470582673 | Down | 1.82E-239 |
| 10991 | 1.27358481 | 0.029713847 | -5.421615823 | Down | 4.78E-12 |
| 4489 | 23.4702222 | 0.552182322 | -5.409542991 | Down | 9.50E-34 |
| 4606 | 4.2546157 | 0.101289698 | -5.392469478 | Down | 9.91E-55 |
| 23109 | 1.60627652 | 0.038935933 | -5.366474269 | Down | 3.69E-22 |
| 10930 | 42.0883513 | 1.026592485 | -5.357485486 | Down | 3.38E-240 |
| 9671 | 23.8099152 | 0.581846485 | -5.354780113 | Down | 0 |
| 113622 | 19.0667109 | 0.467231195 | -5.350775583 | Down | 2.17E-125 |
| 2208 | 1.83884656 | 0.045159892 | -5.347615242 | Down | 1.96E-11 |
| 590 | 40.7588905 | 1.012747961 | -5.330767697 | Down | 0 |
| 414332 | 2.90416168 | 0.07227374 | -5.328506419 | Down | 1.51E-21 |
| 171024 | 314.116267 | 7.886730894 | -5.315727432 | Down | 0 |
| 161247 | 22.8934805 | 0.58136045 | -5.299360073 | Down | 8.33E-81 |
| 845 | 27.5094672 | 0.701885084 | -5.292549541 | Down | 5.69E-258 |
| 200539 | 33.152764 | 0.846879161 | -5.290829216 | Down | 5.18E-297 |
| 221662 | 11.5472551 | 0.29605226 | -5.285554274 | Down | 1.35E-99 |
| 85366 | 11.1575126 | 0.287063634 | -5.280501046 | Down | 5.47E-109 |
| 2173 | 5.5508346 | 0.145922055 | -5.249434848 | Down | 2.50E-20 |
| 253868 | 19.1253146 | 0.507537793 | -5.235824417 | Down | 2.38E-171 |
| 635 | 2.15563143 | 0.058310453 | -5.208212185 | Down | 1.02E-19 |
| 4619 | 0.89474989 | 0.024203243 | -5.208212185 | Down | 1.02E-19 |
| 5212 | 3.92087013 | 0.106834835 | -5.197719812 | Down | 5.36E-38 |
| 2170 | 170.230892 | 4.651016091 | -5.193803126 | Down | 0 |
| 23336 | 342.221879 | 9.376136914 | -5.189794552 | Down | 0 |
| 6854 | 0.68369928 | 0.018766238 | -5.18715057 | Down | 3.25E-10 |
| 8839 | 85.0561662 | 2.336155976 | -5.186207324 | Down | 0 |
| 4629 | 3649.35525 | 100.5859517 | -5.181140867 | Down | 0 |
| 126393 | 984.349916 | 28.26476153 | -5.122094825 | Down | 0 |
| 443 | 3.58016095 | 0.102804043 | -5.122055542 | Down | 1.67E-18 |
| 7352 | 7.28484727 | 0.21057221 | -5.112511769 | Down | 2.26E-61 |
| 6332 | 2.63205859 | 0.076362563 | -5.107182265 | Down | 1.42E-52 |
| 8988 | 62.2364006 | 1.815037041 | -5.099687729 | Down | 1.62E-162 |
| 685 | 3.77820533 | 0.110186042 | -5.099687729 | Down | 3.36E-18 |
| 6450 | 44.6846104 | 1.306876497 | -5.09558333 | Down | 2.50E-187 |
| 1804 | 10.8908674 | 0.319614411 | -5.090642589 | Down | 8.86E-170 |
| 5239 | 154.684305 | 4.541856747 | -5.08990081 | Down | 0 |
| 9413 | 20.4684868 | 0.601634886 | -5.088372415 | Down | 1.78E-169 |
| 286 | 5.52808221 | 0.163368322 | -5.080578906 | Down | 4.28E-160 |
| 285313 | 0.62505882 | 0.01851831 | -5.076967652 | Down | 6.75E-18 |
| 7140 | 46.7446912 | 1.39353743 | -5.067978869 | Down | 1.47E-191 |
| 149134 | 2.15599309 | 0.064904779 | -5.053884039 | Down | 2.65E-09 |
| 388228 | 2.40941478 | 0.073004874 | -5.044546174 | Down | 5.90E-42 |
| 6913 | 5.49734956 | 0.166839637 | -5.042202234 | Down | 3.15E-66 |
| 53632 | 3.09149001 | 0.095112744 | -5.022519868 | Down | 3.14E-25 |
| 126 | 6.36273631 | 0.197930934 | -5.006578324 | Down | 7.66E-33 |
| 728464 | 2.12813592 | 0.066201695 | -5.006578324 | Down | 5.34E-09 |
| 28999 | 1.85590309 | 0.057733122 | -5.006578324 | Down | 5.47E-17 |
| 339896 | 2.54287497 | 0.079768062 | -4.994505492 | Down | 1.54E-32 |
| 26287 | 25.1909106 | 0.803727818 | -4.970052448 | Down | 8.62E-124 |
| 6123 | 48.7821921 | 1.573123736 | -4.954650532 | Down | 1.44E-250 |
| 11170 | 21.1941204 | 0.694372458 | -4.931810556 | Down | 4.80E-246 |
| 145781 | 17.7948883 | 0.595900715 | -4.900247089 | Down | 5.11E-276 |
| 2045 | 17.3509061 | 0.581437014 | -4.89924428 | Down | 0 |
| 2277 | 9.21247961 | 0.310749691 | -4.889764659 | Down | 5.82E-66 |
| 347 | 233.754826 | 7.933833782 | -4.880834168 | Down | 0 |
| 27129 | 276.869894 | 9.501660115 | -4.864884779 | Down | 0 |
| 84709 | 3.14976831 | 0.108869405 | -4.854575231 | Down | 3.55E-15 |
| 572558 | 47.7457332 | 1.650295844 | -4.854575231 | Down | 4.47E-85 |
| 196410 | 2.802352 | 0.096861218 | -4.854575231 | Down | 3.55E-15 |
| 23671 | 1.16571027 | 0.040291911 | -4.854575231 | Down | 4.31E-08 |
| 7148 | 12.1274724 | 0.422056613 | -4.844698551 | Down | 0 |
| 127294 | 16.603533 | 0.578077768 | -4.844082857 | Down | 0 |
| 79442 | 10.6461812 | 0.371149575 | -4.842191506 | Down | 8.93E-181 |
| 8842 | 1.96311415 | 0.06848773 | -4.841154715 | Down | 6.37E-29 |
| 2318 | 161.4274 | 5.647269758 | -4.837188121 | Down | 0 |
| 59353 | 20.960801 | 0.740159282 | -4.823714267 | Down | 3.65E-144 |
| 3131 | 29.0426127 | 1.028426014 | -4.819661334 | Down | 0 |
| 6097 | 4.02485614 | 0.142638095 | -4.818505976 | Down | 5.20E-42 |
| 203859 | 3.69348676 | 0.131310133 | -4.813933246 | Down | 2.26E-82 |
| 5346 | 2.76585894 | 0.098331287 | -4.813933246 | Down | 2.54E-28 |
| 10351 | 7.16850679 | 0.254853379 | -4.813933246 | Down | 2.66E-136 |
| 478 | 2.30942601 | 0.082104271 | -4.813933246 | Down | 2.54E-28 |
| 2167 | 55.3615005 | 2.00050779 | -4.790444893 | Down | 1.37E-153 |
| 91807 | 9.06216577 | 0.328078319 | -4.787743722 | Down | 5.75E-239 |
| 729 | 3.28538612 | 0.119455995 | -4.781511769 | Down | 8.25E-41 |
| 57644 | 5.65060862 | 0.208615868 | -4.759485462 | Down | 1.41E-117 |
| 5507 | 65.1918142 | 2.409757156 | -4.757731153 | Down | 0 |
| 6262 | 8.52852583 | 0.316226869 | -4.753264528 | Down | 0 |
| 730 | 123.714344 | 4.632716088 | -4.739010702 | Down | 0 |
| 154796 | 4.44211985 | 0.167920671 | -4.725396572 | Down | 7.49E-102 |
| 341640 | 0.35760373 | 0.013529526 | -4.724178593 | Down | 4.14E-20 |
| 2875 | 4.05835294 | 0.154587628 | -4.714397573 | Down | 3.20E-26 |
| 92293 | 0.78891503 | 0.030050739 | -4.714397573 | Down | 1.13E-13 |
| 79698 | 1.55443819 | 0.059210452 | -4.714397573 | Down | 1.13E-13 |
| 3270 | 26.4710114 | 1.008937161 | -4.713505092 | Down | 2.04E-206 |
| 440503 | 15.6141184 | 0.597198414 | -4.708496971 | Down | 3.23E-125 |
| 4129 | 79.9686231 | 3.06157967 | -4.707085916 | Down | 0 |
| 165904 | 20.5456661 | 0.790910658 | -4.699175561 | Down | 0 |
| 1674 | 8175.79144 | 315.2214333 | -4.696920856 | Down | 0 |
| 9717 | 0.58068839 | 0.022579946 | -4.684650229 | Down | 2.25E-13 |
| 340533 | 0.31998329 | 0.012442483 | -4.684650229 | Down | 2.25E-13 |
| 3679 | 76.5148299 | 3.016422886 | -4.664828788 | Down | 0 |
| 93986 | 3.4754388 | 0.137598831 | -4.658655021 | Down | 5.25E-43 |
| 7138 | 1806.19688 | 71.5666225 | -4.657524452 | Down | 0 |
| 55607 | 3.40908006 | 0.135381818 | -4.65427658 | Down | 5.20E-108 |
| 84502 | 0.8335199 | 0.033100848 | -4.65427658 | Down | 4.48E-13 |
| 1288 | 59.6555451 | 2.37229001 | -4.652303953 | Down | 0 |
| 4137 | 8.52653348 | 0.342247707 | -4.63884654 | Down | 2.05E-188 |
| 1114 | 1.35676702 | 0.05505141 | -4.623249685 | Down | 8.91E-13 |
| 115265 | 11.7214147 | 0.478046732 | -4.615851232 | Down | 5.79E-99 |
| 2903 | 2.54809561 | 0.104253467 | -4.611252152 | Down | 1.95E-121 |
| 282996 | 1.4685403 | 0.060462934 | -4.602188069 | Down | 1.98E-35 |
| 729359 | 36.4265652 | 1.502965009 | -4.599107638 | Down | 0 |
| 643008 | 2.27042408 | 0.094170628 | -4.591540825 | Down | 1.77E-12 |
| 2925 | 1.95183641 | 0.081560686 | -4.580814418 | Down | 5.12E-18 |
| 5350 | 185.094932 | 7.739572984 | -4.579867613 | Down | 0 |
| 2888 | 7.98367471 | 0.335889707 | -4.570993447 | Down | 2.97E-62 |
| 26353 | 476.808108 | 20.09700933 | -4.568356037 | Down | 0 |
| 6663 | 10.2608572 | 0.432947984 | -4.566813739 | Down | 4.27E-95 |
| 90523 | 10.4567102 | 0.441745857 | -4.565068614 | Down | 5.90E-62 |
| 9365 | 0.68661616 | 0.0291261 | -4.559119347 | Down | 3.52E-12 |
| 255167 | 1.13378881 | 0.048095062 | -4.559119347 | Down | 3.52E-12 |
| 152573 | 8.67806536 | 0.369802468 | -4.552546693 | Down | 6.98E-56 |
| 10891 | 4.30194693 | 0.184585164 | -4.542631224 | Down | 7.66E-88 |
| 7111 | 36.2661042 | 1.559541962 | -4.539427499 | Down | 4.00E-308 |
| 140458 | 29.9184377 | 1.29401825 | -4.531104971 | Down | 2.98E-279 |
| 158696 | 1.15488696 | 0.050129344 | -4.525952483 | Down | 6.99E-12 |
| 23242 | 7.89851033 | 0.343164155 | -4.524609817 | Down | 2.16E-134 |
| 43 | 11.3046695 | 0.492986584 | -4.51922662 | Down | 2.38E-107 |
| 84417 | 74.7068235 | 3.262253614 | -4.517299171 | Down | 2.23E-186 |
| 139728 | 53.0429342 | 2.338654539 | -4.503409915 | Down | 0 |
| 1837 | 19.9986695 | 0.88288213 | -4.501539368 | Down | 0 |
| 284427 | 2.1149637 | 0.09398848 | -4.492005151 | Down | 1.39E-11 |
| 283174 | 23.5661975 | 1.057742389 | -4.47765878 | Down | 0 |
| 79785 | 8.54031829 | 0.385651145 | -4.468921538 | Down | 7.28E-32 |
| 26038 | 2.32380705 | 0.105788195 | -4.457239733 | Down | 7.37E-72 |
| 119 | 1.18337255 | 0.054313015 | -4.445462565 | Down | 3.10E-16 |
| 2134 | 2.37382057 | 0.108950772 | -4.445462565 | Down | 2.84E-31 |
| 92949 | 10.2611434 | 0.475539387 | -4.431482853 | Down | 3.49E-252 |
| 1264 | 2153.58284 | 100.048752 | -4.427963745 | Down | 0 |
| 1297 | 0.82604535 | 0.03854472 | -4.421615823 | Down | 5.44E-11 |
| 6517 | 64.3868908 | 3.032180599 | -4.408339395 | Down | 0 |
| 10218 | 6.87498207 | 0.325683943 | -4.399811453 | Down | 2.28E-49 |
| 90865 | 22.5682214 | 1.075126548 | -4.391714338 | Down | 6.04E-187 |
| 816 | 8.32799076 | 0.397340092 | -4.389522194 | Down | 3.38E-120 |
| 6376 | 46.126582 | 2.204680287 | -4.386957031 | Down | 0 |
| 80000 | 0.99403294 | 0.048190457 | -4.366474269 | Down | 5.60E-20 |
| 5179 | 9.01634077 | 0.437109844 | -4.366474269 | Down | 1.05E-38 |
| 1410 | 1399.29094 | 67.93041215 | -4.364494545 | Down | 0 |
| 348093 | 116.683857 | 5.665822618 | -4.364175732 | Down | 0 |
| 25802 | 614.484467 | 30.00408685 | -4.356145614 | Down | 0 |
| 660 | 1.14901214 | 0.056436753 | -4.347615242 | Down | 2.12E-10 |
| 170961 | 0.7371829 | 0.036208677 | -4.347615242 | Down | 2.12E-10 |
| 81493 | 7.22219754 | 0.354737226 | -4.347615242 | Down | 2.02E-79 |
| 100192420 | 0.62377015 | 0.030638111 | -4.347615242 | Down | 1.07E-05 |
| 1285 | 2.36352941 | 0.117038644 | -4.335885949 | Down | 1.73E-60 |
| 64091 | 32.0201609 | 1.596089046 | -4.326367508 | Down | 1.68E-163 |
| 125 | 43.2738427 | 2.174140685 | -4.314978034 | Down | 0 |
| 3757 | 13.3878699 | 0.673330868 | -4.31346702 | Down | 9.69E-162 |
| 114905 | 7.6944104 | 0.387273322 | -4.312386754 | Down | 2.91E-108 |
| 4969 | 58.9270913 | 2.970658072 | -4.310076593 | Down | 0 |
| 5409 | 2.49766272 | 0.125994929 | -4.309141094 | Down | 4.19E-10 |
| 9892 | 0.61146758 | 0.030845563 | -4.309141094 | Down | 4.19E-10 |
| 164045 | 0.5860466 | 0.029563199 | -4.309141094 | Down | 4.19E-10 |
| 7123 | 170.780659 | 8.640486751 | -4.304888205 | Down | 0 |
| 171019 | 2.25605826 | 0.114581358 | -4.299360073 | Down | 1.21E-36 |
| 2254 | 0.94826542 | 0.048270243 | -4.296084941 | Down | 1.83E-14 |
| 117245 | 4.59131823 | 0.234140914 | -4.293459472 | Down | 3.01E-45 |
| 5354 | 17.2647827 | 0.883491715 | -4.288471757 | Down | 4.42E-167 |
| 167838 | 11.20994 | 0.576005651 | -4.282551786 | Down | 6.44E-162 |
| 57863 | 16.3681736 | 0.847535656 | -4.271475474 | Down | 3.74E-186 |
| 2823 | 23.3939867 | 1.211771678 | -4.270947941 | Down | 5.91E-259 |
| 2735 | 0.39051294 | 0.020246685 | -4.26961273 | Down | 2.12E-05 |
| 55698 | 1.1491934 | 0.059581526 | -4.26961273 | Down | 3.61E-14 |
| 1908 | 2.11883435 | 0.109853906 | -4.26961273 | Down | 1.66E-18 |
| 4684 | 21.7577106 | 1.136203517 | -4.259233584 | Down | 0 |
| 1756 | 8.71257304 | 0.455453373 | -4.257723575 | Down | 0 |
| 654790 | 22.3228605 | 1.177265118 | -4.245010757 | Down | 4.10E-98 |
| 1113 | 2.02417466 | 0.106926259 | -4.242645682 | Down | 7.09E-14 |
| 55885 | 11.8400758 | 0.625816508 | -4.241794786 | Down | 1.62E-135 |
| 79041 | 14.4189394 | 0.762521311 | -4.241043578 | Down | 1.13E-72 |
| 10628 | 895.097821 | 47.33864514 | -4.240955035 | Down | 0 |
| 26577 | 29.5235962 | 1.564612945 | -4.237990751 | Down | 1.30E-172 |
| 6258 | 5.21531941 | 0.276990481 | -4.234847312 | Down | 1.40E-30 |
| 5332 | 20.0520305 | 1.068198549 | -4.2304966 | Down | 0 |
| 84688 | 4.67280442 | 0.249189971 | -4.228970745 | Down | 6.41E-18 |
| 65055 | 16.216473 | 0.870192332 | -4.219981962 | Down | 1.61E-190 |
| 79933 | 14.3563928 | 0.77067496 | -4.219426973 | Down | 4.79E-215 |
| 4118 | 28.2530196 | 1.518501384 | -4.217684938 | Down | 8.85E-92 |
| 5596 | 4.28772655 | 0.230853251 | -4.215164946 | Down | 6.41E-63 |
| 126306 | 12.249998 | 0.660816559 | -4.212387861 | Down | 2.55E-42 |
| 10316 | 1.64602278 | 0.089050784 | -4.208212185 | Down | 1.26E-17 |
| 221476 | 54.7431383 | 2.962117769 | -4.207977238 | Down | 0 |
| 55638 | 2.89447517 | 0.157571484 | -4.199223402 | Down | 1.06E-29 |
| 154091 | 8.3138315 | 0.455631778 | -4.189573232 | Down | 1.18E-141 |
| 128344 | 2.82980392 | 0.15534541 | -4.18715057 | Down | 2.29E-21 |
| 29958 | 0.86133247 | 0.047283858 | -4.18715057 | Down | 3.19E-09 |
| 80725 | 0.37666496 | 0.020677466 | -4.18715057 | Down | 3.19E-09 |
| 158931 | 3.41571371 | 0.190308267 | -4.165776919 | Down | 4.83E-17 |
| 10486 | 34.5746509 | 1.92634467 | -4.165776919 | Down | 0 |
| 128209 | 1.24844291 | 0.069905435 | -4.158581418 | Down | 5.36E-13 |
| 9068 | 13.942171 | 0.781090391 | -4.157821905 | Down | 1.50E-149 |
| 401265 | 2.47512991 | 0.139607998 | -4.14805075 | Down | 5.42E-44 |
| 374462 | 0.34137555 | 0.019308097 | -4.144081848 | Down | 6.27E-09 |
| 253152 | 1.78612988 | 0.101022961 | -4.144081848 | Down | 6.27E-09 |
| 5348 | 162.487652 | 9.194289918 | -4.143448114 | Down | 7.65E-267 |
| 84913 | 5.70247488 | 0.323283816 | -4.140714997 | Down | 2.40E-101 |
| 55859 | 5.8894063 | 0.338227687 | -4.122055542 | Down | 1.85E-16 |
| 256691 | 15.3053441 | 0.886422035 | -4.109897927 | Down | 6.11E-166 |
| 285359 | 2.54509452 | 0.148448201 | -4.099687729 | Down | 1.23E-08 |
| 122060 | 1.57915932 | 0.092107919 | -4.099687729 | Down | 2.05E-12 |
| 7049 | 29.0450086 | 1.704053923 | -4.091247366 | Down | 0 |
| 145270 | 11.897271 | 0.698926665 | -4.089345784 | Down | 5.05E-130 |
| 64753 | 9.40678966 | 0.559601278 | -4.071231282 | Down | 3.52E-117 |
| 7704 | 9.15416869 | 0.545297255 | -4.06931408 | Down | 1.76E-66 |
| 23066 | 9.57573109 | 0.571422124 | -4.066753839 | Down | 4.07E-131 |
| 762 | 8.45998573 | 0.505289889 | -4.065472013 | Down | 3.18E-30 |
| 2899 | 1.58424818 | 0.094875453 | -4.061619688 | Down | 1.64E-44 |
| 291 | 472.856394 | 28.36368197 | -4.059285374 | Down | 0 |
| 89876 | 2.16706933 | 0.131537228 | -4.042202234 | Down | 1.21E-29 |
| 143662 | 2.83297504 | 0.171956512 | -4.042202234 | Down | 1.21E-29 |
| 25854 | 9.70505534 | 0.593024209 | -4.032573533 | Down | 2.07E-78 |
| 140578 | 1.87200703 | 0.114558847 | -4.030425066 | Down | 2.69E-15 |
| 196374 | 2.67956011 | 0.163977652 | -4.030425066 | Down | 2.69E-15 |
| 32 | 19.2955362 | 1.182096439 | -4.028847488 | Down | 0 |
| 55022 | 15.9692393 | 0.981483531 | -4.024187724 | Down | 2.58E-133 |
| 58473 | 18.3770796 | 1.133133806 | -4.01951738 | Down | 2.52E-129 |
| 146395 | 0.47604178 | 0.029617256 | -4.006578324 | Down | 4.69E-08 |
| 132228 | 0.81697268 | 0.050828498 | -4.006578324 | Down | 0.00016133 |
| 5026 | 2.13686972 | 0.13294677 | -4.006578324 | Down | 5.24E-15 |
| 221481 | 2.00434358 | 0.124701568 | -4.006578324 | Down | 4.69E-08 |
| 114815 | 1.27808141 | 0.080184892 | -3.994505492 | Down | 1.73E-28 |
| 286133 | 24.5133227 | 1.546536544 | -3.98645322 | Down | 1.12E-259 |
| 8654 | 34.6315526 | 2.190083553 | -3.98302925 | Down | 0 |
| 57452 | 4.75547235 | 0.300879531 | -3.982330778 | Down | 1.27E-41 |
| 89944 | 1.46101861 | 0.092438892 | -3.982330778 | Down | 1.02E-14 |
| 57191 | 2.61729922 | 0.166537852 | -3.974156846 | Down | 2.98E-11 |
| 54511 | 1.34186407 | 0.08538235 | -3.974156846 | Down | 2.98E-11 |
| 72 | 6592.65649 | 420.5712625 | -3.970437732 | Down | 0 |
| 2353 | 302.846771 | 19.33765028 | -3.969103619 | Down | 0 |
| 5137 | 2.11636561 | 0.13527852 | -3.967584193 | Down | 6.99E-18 |
| 1393 | 1.23230417 | 0.079312368 | -3.957668724 | Down | 9.15E-08 |
| 56997 | 111.784997 | 7.20405308 | -3.955773953 | Down | 0 |
| 126326 | 10.4776178 | 0.675514981 | -3.955179172 | Down | 4.51E-132 |
| 3779 | 184.624839 | 11.91578146 | -3.953651191 | Down | 0 |
| 111 | 11.4450175 | 0.739884069 | -3.951276622 | Down | 2.25E-128 |
| 10580 | 103.17991 | 6.672139231 | -3.950868892 | Down | 0 |
| 1580 | 5.17547271 | 0.337757491 | -3.93763097 | Down | 1.28E-33 |
| 5588 | 6.49257712 | 0.424807749 | -3.933909256 | Down | 2.12E-62 |
| 140597 | 78.741029 | 5.163732228 | -3.930629471 | Down | 1.93E-250 |
| 8470 | 8.05155938 | 0.528507418 | -3.929272598 | Down | 3.13E-148 |
| 358 | 640.985729 | 42.08134556 | -3.929039497 | Down | 0 |
| 81578 | 5.31539302 | 0.349331735 | -3.927506753 | Down | 3.05E-65 |
| 347273 | 12.6451809 | 0.833006474 | -3.924116164 | Down | 8.37E-97 |
| 57571 | 5.03248539 | 0.332822221 | -3.918447414 | Down | 2.08E-58 |
| 100128569 | 1.63199438 | 0.108788159 | -3.907042651 | Down | 0.00031556 |
| 339240 | 0.34679234 | 0.023117052 | -3.907042651 | Down | 0.00031556 |
| 547 | 0.49611444 | 0.033070811 | -3.907042651 | Down | 7.47E-14 |
| 89874 | 0.54808834 | 0.036535372 | -3.907042651 | Down | 0.00031556 |
| 51086 | 1.6724323 | 0.111483736 | -3.907042651 | Down | 5.11E-17 |
| 1381 | 2.83273636 | 0.188829188 | -3.907042651 | Down | 1.78E-07 |
| 7957 | 15.9360837 | 1.072811368 | -3.892828791 | Down | 5.63E-159 |
| 6297 | 5.50152596 | 0.370969394 | -3.890459766 | Down | 1.36E-78 |
| 23026 | 0.94304842 | 0.063620657 | -3.889764659 | Down | 6.89E-20 |
| 222256 | 5.62338635 | 0.381260655 | -3.882589607 | Down | 1.39E-105 |
| 8643 | 2.04739125 | 0.140788208 | -3.862188415 | Down | 1.82E-22 |
| 5375 | 1.18270885 | 0.081758908 | -3.854575231 | Down | 2.80E-13 |
| 79940 | 0.71898053 | 0.049702057 | -3.854575231 | Down | 3.46E-07 |
| 50859 | 0.70574628 | 0.048787193 | -3.854575231 | Down | 3.46E-07 |
| 57453 | 0.30566312 | 0.021130038 | -3.854575231 | Down | 3.46E-07 |
| 171425 | 7.09426369 | 0.490415922 | -3.854575231 | Down | 2.50E-25 |
| 126823 | 4.58415778 | 0.320857226 | -3.836653323 | Down | 4.98E-19 |
| 85445 | 0.64202703 | 0.044937156 | -3.836653323 | Down | 4.22E-10 |
| 441376 | 3.50235822 | 0.245139237 | -3.836653323 | Down | 4.98E-19 |
| 117283 | 3.62559853 | 0.25828514 | -3.811182635 | Down | 3.48E-30 |
| 64093 | 3.89555019 | 0.278122903 | -3.808032645 | Down | 1.37E-41 |
| 9087 | 1.21815674 | 0.08744819 | -3.800127447 | Down | 6.70E-07 |
| 57094 | 1.06652694 | 0.076563095 | -3.800127447 | Down | 6.70E-07 |
| 57158 | 40.6916933 | 2.924662228 | -3.798392395 | Down | 0 |
| 4660 | 94.3557901 | 6.782989433 | -3.798117901 | Down | 0 |
| 5649 | 0.870882 | 0.06300291 | -3.788986889 | Down | 1.29E-29 |
| 389125 | 95.0910043 | 6.897599509 | -3.785142597 | Down | 2.88E-189 |
| 57447 | 136.293065 | 9.96114009 | -3.774257473 | Down | 0 |
| 1589 | 7.05657254 | 0.517181172 | -3.770225961 | Down | 5.11E-43 |
| 1152 | 845.429135 | 61.98796427 | -3.7696238 | Down | 0 |
| 22874 | 4.1484413 | 0.305383168 | -3.76387692 | Down | 8.27E-87 |
| 287 | 4.99005513 | 0.368457543 | -3.759485462 | Down | 1.25E-198 |
| 11248 | 5.67123298 | 0.420255518 | -3.754323757 | Down | 8.46E-89 |
| 9200 | 30.8346867 | 2.286465742 | -3.753362979 | Down | 3.21E-113 |
| 3899 | 2.89873192 | 0.214982622 | -3.753129967 | Down | 6.41E-67 |
| 51286 | 4.17559402 | 0.309973551 | -3.751764425 | Down | 1.80E-20 |
| 10875 | 115.433722 | 8.573057488 | -3.751111119 | Down | 0 |
| 6442 | 57.3225002 | 4.275556971 | -3.74491726 | Down | 2.04E-228 |
| 2018 | 0.67422814 | 0.050337062 | -3.743543918 | Down | 1.30E-06 |
| 9628 | 0.34040528 | 0.025414249 | -3.743543918 | Down | 1.30E-06 |
| 3856 | 138.24506 | 10.38939032 | -3.73404503 | Down | 0 |
| 10290 | 29.1289653 | 2.190063969 | -3.733409541 | Down | 0 |
| 116496 | 65.4726577 | 4.946201449 | -3.726499639 | Down | 0 |
| 81616 | 1.06555891 | 0.080628392 | -3.724178593 | Down | 3.04E-09 |
| 26548 | 16.3794092 | 1.239392223 | -3.724178593 | Down | 2.94E-57 |
| 6525 | 542.568769 | 41.26783362 | -3.716716397 | Down | 0 |
| 3357 | 5.56126804 | 0.424337154 | -3.712130966 | Down | 1.28E-35 |
| 5648 | 3.014297 | 0.230042453 | -3.711849539 | Down | 3.58E-38 |
| 4638 | 364.707989 | 27.86787227 | -3.710067043 | Down | 0 |
| 53342 | 12.2127099 | 0.939985811 | -3.699600571 | Down | 8.27E-64 |
| 57188 | 2.29995352 | 0.178866421 | -3.684650229 | Down | 1.35E-47 |
| 5732 | 7.18619456 | 0.558867338 | -3.684650229 | Down | 3.69E-50 |
| 255426 | 1.69175281 | 0.131566907 | -3.684650229 | Down | 1.44E-11 |
| 153579 | 9.37498915 | 0.729088976 | -3.684650229 | Down | 4.21E-91 |
| 79974 | 8.80856258 | 0.685038219 | -3.684650229 | Down | 1.93E-129 |
| 1768 | 0.14649958 | 0.011393211 | -3.684650229 | Down | 2.50E-06 |
| 340543 | 1.6048477 | 0.124808333 | -3.684650229 | Down | 2.50E-06 |
| 8912 | 27.9075767 | 2.180874392 | -3.677678269 | Down | 0 |
| 94274 | 185.684008 | 14.51670404 | -3.677063735 | Down | 0 |
| 30812 | 2.10118512 | 0.167393853 | -3.649884811 | Down | 8.97E-19 |
| 158471 | 26.2824363 | 2.096581649 | -3.647988096 | Down | 0 |
| 25924 | 1.49179312 | 0.119758581 | -3.63884654 | Down | 4.52E-21 |
| 26166 | 1.08268406 | 0.08710333 | -3.635740629 | Down | 1.33E-13 |
| 22973 | 1.16419931 | 0.094475783 | -3.623249685 | Down | 4.80E-06 |
| 491 | 0.40088363 | 0.032532054 | -3.623249685 | Down | 5.28E-11 |
| 91851 | 36.0605365 | 2.933300235 | -3.61982421 | Down | 0 |
| 64072 | 4.26678706 | 0.347679554 | -3.61731998 | Down | 5.86E-129 |
| 1073 | 100.646433 | 8.232536431 | -3.61181524 | Down | 0 |
| 8110 | 1.96030274 | 0.160475708 | -3.610649648 | Down | 2.55E-13 |
| 130733 | 4.8934054 | 0.402943856 | -3.602188069 | Down | 8.36E-23 |
| 56099 | 1.12022274 | 0.092243915 | -3.602188069 | Down | 1.26E-15 |
| 26084 | 14.2648314 | 1.180828849 | -3.594590921 | Down | 3.67E-199 |
| 55026 | 3.01511279 | 0.250116328 | -3.591540825 | Down | 1.12E-29 |
| 124602 | 0.96475884 | 0.080030817 | -3.591540825 | Down | 1.01E-10 |
| 56776 | 0.54668167 | 0.045349551 | -3.591540825 | Down | 1.01E-10 |
| 23127 | 1.68805285 | 0.140656246 | -3.585114556 | Down | 8.13E-25 |
| 23632 | 3.50316382 | 0.293396472 | -3.577735025 | Down | 1.19E-17 |
| 6512 | 2.59661201 | 0.217820146 | -3.57542116 | Down | 6.00E-20 |
| 9378 | 2.45117444 | 0.205876953 | -3.573618917 | Down | 6.58E-64 |
| 50937 | 4.82378356 | 0.40760072 | -3.564936674 | Down | 1.31E-104 |
| 23500 | 28.174739 | 2.380736523 | -3.564922374 | Down | 0 |
| 80274 | 12.8130683 | 1.085323874 | -3.561418463 | Down | 1.25E-131 |
| 6493 | 3.29089191 | 0.279197765 | -3.559119347 | Down | 5.56E-38 |
| 84152 | 2.33332013 | 0.197957812 | -3.559119347 | Down | 9.29E-13 |
| 100129534 | 0.76537057 | 0.064933689 | -3.559119347 | Down | 9.22E-06 |
| 3242 | 1.20748907 | 0.10244282 | -3.559119347 | Down | 9.22E-06 |
| 5334 | 2.86116051 | 0.242739549 | -3.559119347 | Down | 1.13E-51 |
| 65266 | 3.53550811 | 0.299950891 | -3.559119347 | Down | 2.89E-40 |
| 2562 | 0.5914826 | 0.050181113 | -3.559119347 | Down | 1.93E-10 |
| 441455 | 1.04326469 | 0.088510099 | -3.559119347 | Down | 9.22E-06 |
| 1950 | 1.40667781 | 0.119341902 | -3.559119347 | Down | 1.14E-19 |
| 10391 | 5.17846407 | 0.441344635 | -3.552546693 | Down | 7.82E-47 |
| 4306 | 3.31360444 | 0.285640744 | -3.536127705 | Down | 7.71E-53 |
| 3778 | 17.790251 | 1.543569191 | -3.52674481 | Down | 0 |
| 54112 | 0.96923847 | 0.084142068 | -3.525952483 | Down | 3.67E-10 |
| 5997 | 341.001479 | 29.66500919 | -3.522943861 | Down | 0 |
| 7169 | 6357.38588 | 554.2129544 | -3.519921322 | Down | 0 |
| 9659 | 38.514298 | 3.364198691 | -3.517059309 | Down | 0 |
| 128434 | 5.09797618 | 0.446025904 | -3.514725228 | Down | 1.01E-27 |
| 10149 | 0.52417844 | 0.04586078 | -3.514725228 | Down | 7.84E-08 |
| 768096 | 1.78903566 | 0.156524123 | -3.514725228 | Down | 1.65E-14 |
| 93649 | 11.7322449 | 1.027774174 | -3.512883881 | Down | 4.10E-215 |
| 338328 | 6.54280454 | 0.575432104 | -3.507191555 | Down | 1.34E-40 |
| 93058 | 16.4799294 | 1.454235909 | -3.502376835 | Down | 7.60E-73 |
| 9472 | 4.12807557 | 0.364896453 | -3.499910349 | Down | 1.38E-113 |
| 81617 | 30.4676582 | 2.696620522 | -3.498054196 | Down | 1.07E-293 |
| 51301 | 0.47748307 | 0.042438467 | -3.492005151 | Down | 1.76E-05 |
| 79804 | 6.42358102 | 0.570924802 | -3.492005151 | Down | 3.14E-14 |
| 1036 | 6.09722808 | 0.54191871 | -3.492005151 | Down | 3.63E-27 |
| 339965 | 0.45233159 | 0.040203015 | -3.492005151 | Down | 1.76E-05 |
| 23017 | 5.39726582 | 0.48044441 | -3.489787326 | Down | 9.77E-68 |
| 7145 | 194.922049 | 17.42759627 | -3.483451792 | Down | 0 |
| 10349 | 1.80113377 | 0.161180371 | -3.482157365 | Down | 3.47E-31 |
| 395 | 4.13556104 | 0.370279353 | -3.481397052 | Down | 1.27E-56 |
| 342527 | 14.9369064 | 1.339736889 | -3.478859775 | Down | 2.77E-90 |
| 7173 | 2.5827247 | 0.231758558 | -3.478199352 | Down | 1.38E-22 |
| 2354 | 80.4812423 | 7.257921871 | -3.471024142 | Down | 0 |
| 6547 | 0.60785993 | 0.055344014 | -3.457239733 | Down | 1.33E-09 |
| 948 | 14.7547305 | 1.356917675 | -3.442772475 | Down | 4.73E-181 |
| 8639 | 114.058885 | 10.55745161 | -3.433445299 | Down | 0 |
| 4128 | 44.8519405 | 4.151738624 | -3.43338288 | Down | 0 |
| 6546 | 6.85155697 | 0.638207091 | -3.424335325 | Down | 7.50E-108 |
| 27190 | 2.2475565 | 0.209749832 | -3.421615823 | Down | 3.36E-05 |
| 79608 | 3.2268241 | 0.301138508 | -3.421615823 | Down | 1.39E-49 |
| 92749 | 0.62707819 | 0.058521129 | -3.421615823 | Down | 3.36E-05 |
| 9211 | 0.66020785 | 0.061612905 | -3.421615823 | Down | 3.36E-05 |
| 148823 | 0.59734293 | 0.055746131 | -3.421615823 | Down | 3.36E-05 |
| 374977 | 7.6588627 | 0.714751849 | -3.421615823 | Down | 1.36E-87 |
| 286097 | 2.74250372 | 0.255940038 | -3.421615823 | Down | 1.58E-29 |
| 10398 | 5703.98928 | 534.9627927 | -3.41446081 | Down | 0 |
| 85480 | 9.44717499 | 0.887188333 | -3.412570684 | Down | 4.80E-65 |
| 388135 | 7.49645378 | 0.7050185 | -3.410475266 | Down | 3.05E-27 |
| 100126784 | 2.53524495 | 0.238586142 | -3.409542991 | Down | 3.13E-25 |
| 91624 | 403.257329 | 38.08933777 | -3.404241645 | Down | 0 |
| 9118 | 1.42708353 | 0.13543772 | -3.397368277 | Down | 4.03E-13 |
| 51083 | 15.7610621 | 1.498983372 | -3.394308477 | Down | 1.02E-32 |
| 3851 | 1.48564706 | 0.141475284 | -3.392469478 | Down | 4.34E-11 |
| 120376 | 9.16904501 | 0.876306315 | -3.387264319 | Down | 1.89E-34 |
| 254778 | 0.87958445 | 0.084190663 | -3.385089947 | Down | 4.76E-09 |
| 56899 | 2.06228906 | 0.197394899 | -3.385089947 | Down | 1.11E-24 |
| 23205 | 1.07632542 | 0.103022002 | -3.385089947 | Down | 4.76E-09 |
| 64409 | 9.32424745 | 0.897083895 | -3.377672476 | Down | 2.67E-78 |
| 441027 | 3.59900698 | 0.347453707 | -3.372706223 | Down | 1.23E-18 |
| 2676 | 12.0935649 | 1.170134676 | -3.369493092 | Down | 1.42E-60 |
| 57569 | 2.67535122 | 0.259094759 | -3.368176564 | Down | 2.23E-43 |
| 164832 | 2.73965376 | 0.267200359 | -3.357999724 | Down | 1.51E-97 |
| 116362 | 29.6168836 | 2.897690715 | -3.353444321 | Down | 2.70E-50 |
| 8987 | 40.3089591 | 3.951941638 | -3.350466982 | Down | 9.45E-250 |
| 155 | 0.56103939 | 0.05511385 | -3.347615242 | Down | 6.38E-05 |
| 137902 | 0.61741176 | 0.060651605 | -3.347615242 | Down | 8.98E-09 |
| 163933 | 1.72485375 | 0.169441456 | -3.347615242 | Down | 1.43E-12 |
| 401431 | 0.48384388 | 0.047530529 | -3.347615242 | Down | 6.38E-05 |
| 9745 | 0.30069892 | 0.029539237 | -3.347615242 | Down | 6.38E-05 |
| 51299 | 14.777192 | 1.453507162 | -3.345762069 | Down | 7.75E-78 |
| 2934 | 882.161254 | 86.82293713 | -3.344894263 | Down | 0 |
| 130497 | 59.5872439 | 5.888001144 | -3.339153663 | Down | 6.41E-284 |
| 23114 | 20.4156674 | 2.017418659 | -3.339094316 | Down | 0 |
| 9760 | 5.52068646 | 0.546969271 | -3.335315977 | Down | 1.16E-58 |
| 1675 | 387.884813 | 38.48791775 | -3.333150763 | Down | 0 |
| 651 | 1.2803703 | 0.127115568 | -3.332348485 | Down | 7.84E-20 |
| 89 | 1.28485182 | 0.127560494 | -3.332348485 | Down | 1.54E-10 |
| 51617 | 2.50864844 | 0.249723568 | -3.328506419 | Down | 4.52E-16 |
| 5260 | 7.14985259 | 0.713090797 | -3.325755808 | Down | 4.65E-27 |
| 5101 | 5.0429233 | 0.503402965 | -3.324474665 | Down | 6.33E-80 |
| 79443 | 22.4803217 | 2.245346668 | -3.32365257 | Down | 0 |
| 65975 | 3.20258119 | 0.323109271 | -3.309141094 | Down | 4.87E-23 |
| 196951 | 1.44924562 | 0.146214777 | -3.309141094 | Down | 1.69E-08 |
| 445577 | 2.50415577 | 0.252644944 | -3.309141094 | Down | 1.69E-08 |
| 23551 | 49.3528468 | 5.003083969 | -3.302243734 | Down | 0 |
| 27443 | 0.63745902 | 0.06466294 | -3.30132159 | Down | 2.90E-10 |
| 23171 | 23.1274654 | 2.34642217 | -3.301072656 | Down | 1.03E-225 |
| 1795 | 2.71575444 | 0.276027794 | -3.298467593 | Down | 3.28E-60 |
| 166929 | 8.96649817 | 0.912856324 | -3.296084941 | Down | 9.64E-140 |
| 375719 | 5.62436877 | 0.57301939 | -3.295035327 | Down | 7.71E-46 |
| 1903 | 67.3323787 | 6.862499837 | -3.294494318 | Down | 0 |
| 401093 | 11.2666038 | 1.151210436 | -3.290829216 | Down | 1.52E-186 |
| 306 | 118.553743 | 12.11797891 | -3.290320211 | Down | 0 |
| 28513 | 7.60182177 | 0.77808297 | -3.288349293 | Down | 2.15E-61 |
| 2766 | 25.3640088 | 2.597990487 | -3.287314732 | Down | 1.86E-96 |
| 143098 | 11.3268256 | 1.162064832 | -3.284981131 | Down | 5.27E-147 |
| 10472 | 6.08229253 | 0.624903642 | -3.282909553 | Down | 1.41E-64 |
| 3708 | 9.56277379 | 0.99076901 | -3.270808499 | Down | 5.48E-233 |
| 165186 | 1.18870907 | 0.123260541 | -3.26961273 | Down | 9.47E-12 |
| 728819 | 1.20157828 | 0.124594985 | -3.26961273 | Down | 0.00012064 |
| 4588 | 0.17566495 | 0.018215186 | -3.26961273 | Down | 0.00012064 |
| 116173 | 2.34307765 | 0.242960222 | -3.26961273 | Down | 3.18E-08 |
| 10406 | 4.96765579 | 0.51511001 | -3.26961273 | Down | 3.18E-08 |
| 1776 | 0.9609341 | 0.099641923 | -3.26961273 | Down | 0.00012064 |
| 66000 | 3.70935775 | 0.384633596 | -3.26961273 | Down | 4.23E-36 |
| 285489 | 3.01331108 | 0.312458586 | -3.26961273 | Down | 1.75E-20 |
| 116442 | 0.40363095 | 0.041853613 | -3.26961273 | Down | 0.00012064 |
| 441432 | 15.8576413 | 1.659690318 | -3.256192214 | Down | 5.96E-22 |
| 65268 | 16.8913704 | 1.770473958 | -3.254078851 | Down | 1.63E-283 |
| 25890 | 24.8468551 | 2.60721901 | -3.25247958 | Down | 6.58E-273 |
| 23048 | 65.6781581 | 6.892456791 | -3.252323444 | Down | 0 |
| 79825 | 6.1866312 | 0.65054429 | -3.249434848 | Down | 3.27E-42 |
| 91461 | 35.9728207 | 3.791696134 | -3.245992034 | Down | 1.08E-220 |
| 130827 | 3.85288688 | 0.406287996 | -3.245365184 | Down | 2.75E-35 |
| 7079 | 5.793474 | 0.612076135 | -3.242645682 | Down | 1.11E-21 |
| 10235 | 17.9196415 | 1.893196531 | -3.242645682 | Down | 1.77E-102 |
| 222389 | 3.16720362 | 0.335187597 | -3.240168952 | Down | 6.11E-20 |
| 114800 | 0.86279535 | 0.091498954 | -3.237191252 | Down | 1.02E-09 |
| 2247 | 4.45048702 | 0.473438873 | -3.232713147 | Down | 7.12E-75 |
| 49860 | 4.65431001 | 0.4953188 | -3.232138025 | Down | 3.79E-23 |
| 147948 | 1.19006991 | 0.126927412 | -3.228970745 | Down | 5.96E-08 |
| 9201 | 1.94313903 | 0.207504088 | -3.227177464 | Down | 1.14E-39 |
| 9464 | 38.3256788 | 4.093797651 | -3.226799646 | Down | 1.52E-221 |
| 4135 | 8.47960648 | 0.90920665 | -3.221317173 | Down | 1.64E-90 |
| 815 | 7.47095939 | 0.802764754 | -3.218244339 | Down | 3.05E-90 |
| 5593 | 1.22035294 | 0.131408774 | -3.215164946 | Down | 3.31E-11 |
| 100101467 | 0.87983852 | 0.094741854 | -3.215164946 | Down | 3.59E-06 |
| 81796 | 1.42267122 | 0.15393467 | -3.208212185 | Down | 1.94E-14 |
| 1958 | 208.95431 | 22.63806656 | -3.206364862 | Down | 0 |
| 79658 | 42.2459353 | 4.57935496 | -3.205596237 | Down | 0 |
| 55679 | 253.257901 | 27.55486493 | -3.20022832 | Down | 0 |
| 3248 | 8.45876407 | 0.920968349 | -3.199223402 | Down | 5.46E-58 |
| 7044 | 0.65762457 | 0.072202146 | -3.18715057 | Down | 0.00022721 |
| 3026 | 0.43994168 | 0.048302231 | -3.18715057 | Down | 0.00022721 |
| 148753 | 0.91253883 | 0.100189782 | -3.18715057 | Down | 1.11E-07 |
| 26112 | 113.05229 | 12.41606075 | -3.186710791 | Down | 0 |
| 9104 | 6.49981271 | 0.717466426 | -3.179414921 | Down | 2.52E-36 |
| 157869 | 7.30411455 | 0.806453844 | -3.17904552 | Down | 6.31E-68 |
| 92591 | 2.77503352 | 0.306681907 | -3.177690241 | Down | 1.21E-15 |
| 374864 | 1.95547464 | 0.217252061 | -3.170077056 | Down | 4.05E-17 |
| 50651 | 2.73245207 | 0.303573788 | -3.170077056 | Down | 4.05E-17 |
| 367 | 1.21299801 | 0.135165631 | -3.165776919 | Down | 6.72E-14 |
| 22849 | 1.23038324 | 0.137289166 | -3.163818066 | Down | 1.36E-18 |
| 5569 | 13.1601431 | 1.468440899 | -3.163818066 | Down | 4.88E-70 |
| 153 | 3.1928731 | 0.356545398 | -3.162697526 | Down | 2.87E-23 |
| 26033 | 0.4472205 | 0.050083417 | -3.158581418 | Down | 1.15E-10 |
| 1524 | 1.25647664 | 0.140710553 | -3.158581418 | Down | 1.15E-10 |
| 23043 | 5.47498336 | 0.615142139 | -3.153862869 | Down | 4.67E-77 |
| 2674 | 6.49425349 | 0.731853821 | -3.149536278 | Down | 1.08E-148 |
| 7068 | 7.97810997 | 0.899255795 | -3.149243553 | Down | 4.64E-144 |
| 57575 | 0.48031782 | 0.054333259 | -3.144081848 | Down | 2.08E-07 |
| 22854 | 1.53827837 | 0.174009111 | -3.144081848 | Down | 1.25E-13 |
| 9737 | 9.56331524 | 1.086606016 | -3.137681894 | Down | 2.27E-136 |
| 100129583 | 4.15601053 | 0.472991995 | -3.135311638 | Down | 1.39E-16 |
| 783 | 13.8969467 | 1.582395355 | -3.134585937 | Down | 5.16E-148 |
| 317649 | 15.0415138 | 1.715013328 | -3.132658073 | Down | 1.97E-221 |
| 9369 | 44.5809064 | 5.089085227 | -3.130947691 | Down | 0 |
| 274 | 82.1254228 | 9.399529363 | -3.127168465 | Down | 0 |
| 10100 | 21.218763 | 2.432633534 | -3.124749651 | Down | 1.68E-161 |
| 2946 | 66.6806796 | 7.649052336 | -3.123915884 | Down | 6.32E-233 |
| 57538 | 7.09828378 | 0.814690253 | -3.123146701 | Down | 1.36E-183 |
| 57595 | 17.956296 | 2.063053416 | -3.121636701 | Down | 1.63E-159 |
| 2042 | 2.94447699 | 0.338780822 | -3.119587286 | Down | 9.21E-42 |
| 5138 | 10.9970473 | 1.268059611 | -3.116421744 | Down | 8.52E-113 |
| 29951 | 4.84077096 | 0.561887654 | -3.10688323 | Down | 2.63E-38 |
| 1843 | 274.172627 | 31.85237428 | -3.105611724 | Down | 0 |
| 316 | 5.33561164 | 0.620575469 | -3.103975087 | Down | 4.02E-63 |
| 5139 | 2.12111782 | 0.247437665 | -3.099687729 | Down | 6.23E-22 |
| 23416 | 0.32432946 | 0.037834449 | -3.099687729 | Down | 0.0004259 |
| 8785 | 0.55465664 | 0.064703122 | -3.099687729 | Down | 0.0004259 |
| 2843 | 0.81890001 | 0.095528265 | -3.099687729 | Down | 0.0004259 |
| 9542 | 0.82105218 | 0.095779325 | -3.099687729 | Down | 3.86E-07 |
| 401491 | 1.41362151 | 0.164905128 | -3.099687729 | Down | 0.0004259 |
| 4081 | 0.49727076 | 0.058008807 | -3.099687729 | Down | 0.0004259 |
| 1356 | 1.20621758 | 0.140710553 | -3.099687729 | Down | 1.42E-14 |
| 57692 | 1.72221804 | 0.200904263 | -3.099687729 | Down | 4.77E-16 |
| 2620 | 1.39968796 | 0.163279719 | -3.099687729 | Down | 1.23E-08 |
| 339327 | 1.63245122 | 0.190432571 | -3.099687729 | Down | 5.41E-19 |
| 1760 | 147.873268 | 17.35430154 | -3.090996061 | Down | 0 |
| 1346 | 226.927993 | 26.68736291 | -3.088005923 | Down | 0 |
| 4857 | 13.1167695 | 1.54408104 | -3.086592071 | Down | 5.41E-121 |
| 119385 | 4.10935963 | 0.485150363 | -3.082409737 | Down | 1.17E-31 |
| 9734 | 2.64925052 | 0.312958637 | -3.081540382 | Down | 3.39E-30 |
| 1465 | 1630.59077 | 192.7814982 | -3.08035625 | Down | 0 |
| 119587 | 42.0084544 | 4.971420215 | -3.078949748 | Down | 0 |
| 2878 | 228.231641 | 27.06916267 | -3.075776645 | Down | 0 |
| 3316 | 62.7792505 | 7.453476474 | -3.07430241 | Down | 9.00E-124 |
| 9132 | 1.83967553 | 0.218508122 | -3.07369252 | Down | 2.39E-11 |
| 56892 | 18.0662834 | 2.14727528 | -3.072720681 | Down | 3.14E-78 |
| 10800 | 8.09914554 | 0.962854246 | -3.072380383 | Down | 6.25E-30 |
| 96626 | 3.10560207 | 0.369990185 | -3.06931408 | Down | 7.32E-10 |
| 10826 | 16.7270782 | 1.993314503 | -3.068944205 | Down | 7.08E-119 |
| 9510 | 28.3795687 | 3.385040282 | -3.067607758 | Down | 4.00E-306 |
| 7871 | 70.7694267 | 8.443142539 | -3.067274256 | Down | 0 |
| 64168 | 5.94242304 | 0.709165854 | -3.066856336 | Down | 3.90E-72 |
| 2290 | 8.24835845 | 0.986879043 | -3.063161853 | Down | 8.64E-51 |
| 51232 | 22.197136 | 2.662816181 | -3.059348799 | Down | 1.04E-286 |
| 260293 | 1.02722963 | 0.123696337 | -3.053884039 | Down | 7.16E-07 |
| 29091 | 8.33253749 | 1.004582845 | -3.052159358 | Down | 4.14E-39 |
| 1286 | 1.11024857 | 0.133920633 | -3.05143256 | Down | 6.15E-28 |
| 51776 | 166.8066 | 20.13224611 | -3.050596325 | Down | 0 |
| 377007 | 1.86638178 | 0.225405884 | -3.049647046 | Down | 1.00E-16 |
| 8404 | 457.105401 | 55.2116192 | -3.049483048 | Down | 0 |
| 6769 | 3.24219317 | 0.393590639 | -3.042202234 | Down | 1.53E-23 |
| 317719 | 0.89415606 | 0.108842309 | -3.038287184 | Down | 2.33E-05 |
| 25823 | 1.62713725 | 0.198065398 | -3.038287184 | Down | 2.33E-05 |
| 142689 | 3.36082232 | 0.409100467 | -3.038287184 | Down | 1.35E-09 |
| 256309 | 1.23972362 | 0.15090697 | -3.038287184 | Down | 1.35E-09 |
| 5959 | 17.4210863 | 2.127720503 | -3.03345403 | Down | 8.87E-55 |
| 6358 | 7.04897006 | 0.864285373 | -3.027832826 | Down | 1.15E-10 |
| 51807 | 15.0167514 | 1.842067091 | -3.027175229 | Down | 1.08E-70 |
| 388115 | 24.85149 | 3.051255645 | -3.025857387 | Down | 9.51E-303 |
| 79686 | 6.28030022 | 0.77458094 | -3.01934562 | Down | 6.56E-42 |
| 646962 | 11.7564949 | 1.457761329 | -3.011631552 | Down | 6.98E-27 |
| 54932 | 9.56966805 | 1.189079876 | -3.00862325 | Down | 4.48E-64 |
| 7832 | 313.476305 | 38.95787838 | -3.00836938 | Down | 0 |
| 2549 | 10.5269135 | 1.308649063 | -3.007932332 | Down | 5.62E-190 |
| 216 | 31.8595959 | 3.964331859 | -3.006578324 | Down | 1.14E-152 |
| 9312 | 0.65412553 | 0.081393709 | -3.006578324 | Down | 1.32E-06 |
| 140699 | 1.18165103 | 0.148448201 | -2.992772525 | Down | 1.50E-10 |
| 6943 | 13.3660601 | 1.681333204 | -2.990896702 | Down | 3.79E-98 |
| 57687 | 1.53467645 | 0.193542396 | -2.987212999 | Down | 1.85E-14 |
| 158326 | 0.391943 | 0.049429043 | -2.987212999 | Down | 7.72E-08 |
| 4359 | 14.7416138 | 1.864144922 | -2.983308545 | Down | 2.38E-66 |
| 79667 | 1.9459682 | 0.246243468 | -2.982330778 | Down | 9.07E-12 |
| 11126 | 7.0137026 | 0.8883087 | -2.981043232 | Down | 4.28E-26 |
| 4881 | 12.6345167 | 1.602318307 | -2.979137796 | Down | 3.77E-120 |
| 7168 | 3272.14026 | 415.9267013 | -2.975833379 | Down | 0 |
| 158062 | 2.4200802 | 0.307977746 | -2.974156846 | Down | 4.30E-05 |
| 2330 | 0.65283318 | 0.083079103 | -2.974156846 | Down | 4.30E-05 |
| 26261 | 0.76231453 | 0.097011623 | -2.974156846 | Down | 4.30E-05 |
| 25822 | 69.8342169 | 8.889424414 | -2.973772179 | Down | 0 |
| 163175 | 28.5312433 | 3.634308023 | -2.972790011 | Down | 7.99E-186 |
| 80332 | 43.2127705 | 5.509583881 | -2.971442462 | Down | 0 |
| 79085 | 113.113315 | 14.47228371 | -2.966404262 | Down | 0 |
| 728264 | 222.863626 | 28.52840528 | -2.965690161 | Down | 0 |
| 7691 | 1.49453979 | 0.192380248 | -2.957668724 | Down | 1.66E-11 |
| 1805 | 208.671037 | 26.96387661 | -2.95213054 | Down | 0 |
| 4916 | 0.70610075 | 0.091521932 | -2.947684635 | Down | 1.42E-07 |
| 8309 | 14.8557349 | 1.926417701 | -2.947027521 | Down | 1.14E-77 |
| 387496 | 35.3309181 | 4.582075467 | -2.946858113 | Down | 1.81E-122 |
| 64067 | 1.04684501 | 0.136031342 | -2.944036858 | Down | 3.81E-15 |
| 11240 | 10.2752431 | 1.336476121 | -2.94266656 | Down | 7.06E-101 |
| 122622 | 32.1548183 | 4.183421033 | -2.942279831 | Down | 1.46E-130 |
| 5169 | 1.06110941 | 0.138176429 | -2.940989983 | Down | 8.39E-09 |
| 112476 | 24.8273128 | 3.236945686 | -2.939223056 | Down | 1.71E-142 |
| 253738 | 1.66556769 | 0.217276969 | -2.938406822 | Down | 1.46E-17 |
| 26960 | 2.37376287 | 0.310679305 | -2.933677777 | Down | 3.52E-58 |
| 56920 | 7.69393582 | 1.008026451 | -2.932188299 | Down | 2.09E-81 |
| 5630 | 6.05050056 | 0.795287141 | -2.927506753 | Down | 5.43E-26 |
| 8436 | 30.5038982 | 4.027965175 | -2.920870504 | Down | 2.83E-217 |
| 10439 | 10.8974036 | 1.445587957 | -2.914256141 | Down | 2.71E-70 |
| 11030 | 280.490811 | 37.22976381 | -2.913425138 | Down | 0 |
| 23089 | 2.90361746 | 0.385534582 | -2.912919217 | Down | 1.01E-43 |
| 4222 | 8.77557171 | 1.165453094 | -2.912602176 | Down | 4.09E-46 |
| 9314 | 17.5484506 | 2.335996675 | -2.909233532 | Down | 3.21E-114 |
| 9848 | 1.06004482 | 0.141324414 | -2.907042651 | Down | 7.82E-16 |
| 440078 | 0.66979249 | 0.089296253 | -2.907042651 | Down | 4.48E-06 |
| 57101 | 0.88251512 | 0.117656282 | -2.907042651 | Down | 1.53E-08 |
| 162466 | 1.50957603 | 0.201255591 | -2.907042651 | Down | 1.53E-08 |
| 6657 | 1.52958706 | 0.203923447 | -2.907042651 | Down | 9.18E-10 |
| 4131 | 149.131021 | 19.94549477 | -2.90244557 | Down | 0 |
| 10500 | 10.5006822 | 1.405296911 | -2.901536174 | Down | 9.23E-91 |
| 3033 | 37.7849865 | 5.070142698 | -2.897714848 | Down | 6.00E-159 |
| 23263 | 7.00698396 | 0.943796643 | -2.892245649 | Down | 5.14E-84 |
| 22885 | 23.5478853 | 3.177818814 | -2.88948873 | Down | 3.70E-223 |
| 83699 | 9.06097612 | 1.223691172 | -2.888426972 | Down | 4.71E-93 |
| 10253 | 11.9930041 | 1.623573044 | -2.88494888 | Down | 1.58E-56 |
| 219621 | 3.43651388 | 0.466483625 | -2.881047442 | Down | 1.01E-10 |
| 2295 | 74.0313971 | 10.06501878 | -2.87878739 | Down | 0 |
| 6641 | 9.15436448 | 1.245574115 | -2.877648866 | Down | 4.02E-100 |
| 6261 | 12.3870066 | 1.690665956 | -2.873164044 | Down | 0 |
| 158376 | 2.07128468 | 0.28287736 | -2.872277232 | Down | 2.80E-08 |
| 22871 | 1.30223917 | 0.177848068 | -2.872277232 | Down | 2.58E-15 |
| 84911 | 1.1383598 | 0.155466903 | -2.872277232 | Down | 2.80E-08 |
| 10382 | 4.88354618 | 0.669282639 | -2.867241643 | Down | 1.22E-25 |
| 192668 | 23.0372474 | 3.163218772 | -2.864503106 | Down | 2.03E-136 |
| 199964 | 4.34298804 | 0.597987783 | -2.860500065 | Down | 1.11E-11 |
| 63876 | 4.25062343 | 0.586231097 | -2.85813305 | Down | 1.15E-35 |
| 10129 | 7.79058195 | 1.074583792 | -2.857953118 | Down | 8.64E-181 |
| 1215 | 16.8358896 | 2.324236813 | -2.856710975 | Down | 6.30E-30 |
| 2840 | 0.81168972 | 0.112221811 | -2.854575231 | Down | 8.18E-06 |
| 2532 | 61.0532915 | 8.459721779 | -2.851386953 | Down | 1.13E-171 |
| 7025 | 13.6563487 | 1.892308263 | -2.85135277 | Down | 3.64E-95 |
| 8975 | 8.15394566 | 1.130403754 | -2.850660181 | Down | 4.50E-140 |
| 57562 | 9.92500589 | 1.376418123 | -2.850149165 | Down | 3.12E-151 |
| 140612 | 2.69451063 | 0.374516613 | -2.846921658 | Down | 4.00E-25 |
| 220382 | 1.97249954 | 0.274162528 | -2.846921658 | Down | 3.04E-09 |
| 10974 | 378.193188 | 52.93061969 | -2.836948927 | Down | 0 |
| 387923 | 5.97723889 | 0.836725253 | -2.836653323 | Down | 2.02E-11 |
| 84660 | 0.48859924 | 0.068396684 | -2.836653323 | Down | 0.00014533 |
| 1136 | 1.56309383 | 0.218810073 | -2.836653323 | Down | 2.02E-11 |
| 1363 | 37.6680934 | 5.283484224 | -2.83378147 | Down | 4.18E-196 |
| 56475 | 6.31829232 | 0.891900201 | -2.82458049 | Down | 2.92E-21 |
| 22997 | 2.89585462 | 0.408867249 | -2.824284765 | Down | 1.58E-46 |
| 7026 | 58.8062949 | 8.304787525 | -2.823955433 | Down | 0 |
| 23492 | 43.1910073 | 6.107732979 | -2.822022066 | Down | 0 |
| 6840 | 79.8346451 | 11.32369744 | -2.817669849 | Down | 0 |
| 4825 | 26.1741649 | 3.722777588 | -2.813692174 | Down | 2.37E-63 |
| 116535 | 120.386775 | 17.1444992 | -2.811859249 | Down | 0 |
| 2487 | 20.6098722 | 2.937012997 | -2.810914002 | Down | 3.89E-90 |
| 8991 | 116.672899 | 16.62959405 | -2.810644628 | Down | 0 |
| 8516 | 2.34714923 | 0.335271695 | -2.807506977 | Down | 1.19E-17 |
| 84812 | 16.1870649 | 2.313157549 | -2.806905979 | Down | 6.24E-107 |
| 7122 | 39.9288513 | 5.732769272 | -2.800127447 | Down | 1.31E-195 |
| 4635 | 2.23641772 | 0.321092804 | -2.800127447 | Down | 1.49E-05 |
| 27147 | 11.6860603 | 1.684299629 | -2.794567921 | Down | 1.77E-86 |
| 163255 | 3.05262738 | 0.440061412 | -2.79427472 | Down | 1.07E-21 |
| 283120 | 616.52992 | 88.96260104 | -2.792900035 | Down | 0 |
| 200958 | 1.01793909 | 0.14702015 | -2.791565433 | Down | 3.09E-15 |
| 389813 | 2.23835463 | 0.323283816 | -2.791565433 | Down | 3.09E-15 |
| 4886 | 1.64188116 | 0.237764624 | -2.787743722 | Down | 6.61E-11 |
| 166614 | 16.6157307 | 2.422872015 | -2.777759634 | Down | 1.49E-126 |
| 389333 | 2.76277996 | 0.403563579 | -2.775252778 | Down | 3.07E-20 |
| 120114 | 1.23015574 | 0.179857149 | -2.773917567 | Down | 2.17E-50 |
| 64321 | 4.45350929 | 0.651340168 | -2.773459496 | Down | 2.46E-23 |
| 283417 | 3.56149342 | 0.521014032 | -2.773088194 | Down | 1.18E-31 |
| 126669 | 3.65421499 | 0.53722978 | -2.765950331 | Down | 6.10E-49 |
| 6710 | 5.56434378 | 0.818397408 | -2.765338075 | Down | 1.05E-117 |
| 100240735 | 4.9246101 | 0.725655529 | -2.762652741 | Down | 1.19E-10 |
| 9148 | 4.12781412 | 0.608245339 | -2.762652741 | Down | 1.64E-38 |
| 3048 | 5.09073474 | 0.750134475 | -2.762652741 | Down | 1.67E-07 |
| 259236 | 0.79739343 | 0.117498226 | -2.762652741 | Down | 0.00026507 |
| 10659 | 15.8927228 | 2.343084288 | -2.761885554 | Down | 2.00E-305 |
| 5740 | 23.3764127 | 3.447320651 | -2.761506153 | Down | 2.12E-272 |
| 2949 | 45.9251188 | 6.786771494 | -2.75848611 | Down | 1.93E-150 |
| 5577 | 22.7416987 | 3.3646772 | -2.756800015 | Down | 1.56E-173 |
| 151126 | 1.94899946 | 0.288358145 | -2.756800015 | Down | 9.99E-15 |
| 51560 | 5.84364689 | 0.865218055 | -2.755733327 | Down | 9.19E-69 |
| 6335 | 1.03949785 | 0.153983451 | -2.755039557 | Down | 7.02E-22 |
| 51676 | 57.0815415 | 8.47968388 | -2.750941908 | Down | 0 |
| 1183 | 1.30017567 | 0.193280082 | -2.74994169 | Down | 9.89E-20 |
| 165140 | 2.21504788 | 0.330745622 | -2.743543918 | Down | 1.96E-09 |
| 5443 | 1.50777197 | 0.225136885 | -2.743543918 | Down | 2.70E-05 |
| 2669 | 51.9615487 | 7.777386024 | -2.740087177 | Down | 2.94E-235 |
| 285382 | 8.1927643 | 1.235181604 | -2.729627138 | Down | 1.86E-100 |
| 127495 | 14.9247762 | 2.251571018 | -2.728705409 | Down | 4.27E-44 |
| 158427 | 4.14501089 | 0.625507345 | -2.728277162 | Down | 3.93E-39 |
| 84803 | 4.19177269 | 0.632808785 | -2.727718951 | Down | 2.87E-23 |
| 1607 | 3.24843201 | 0.49071858 | -2.726775705 | Down | 5.58E-46 |
| 6425 | 1.53467645 | 0.232250876 | -2.724178593 | Down | 3.01E-07 |
| 28965 | 4.56836844 | 0.693438264 | -2.719839658 | Down | 5.37E-28 |
| 7102 | 36.6295084 | 5.569520028 | -2.717381431 | Down | 3.90E-136 |
| 388 | 594.233 | 90.74826018 | -2.711086837 | Down | 0 |
| 6865 | 16.1312879 | 2.473849938 | -2.705031729 | Down | 1.57E-80 |
| 255027 | 2.67219769 | 0.410370231 | -2.703028759 | Down | 5.75E-14 |
| 2901 | 7.39406589 | 1.136293553 | -2.702032307 | Down | 2.17E-54 |
| 90139 | 18.4703795 | 2.841035017 | -2.700724992 | Down | 1.54E-115 |
| 200197 | 0.20146841 | 0.031336228 | -2.684650229 | Down | 0.00048076 |
| 10861 | 0.38411109 | 0.059744317 | -2.684650229 | Down | 0.00048076 |
| 3592 | 1.30442736 | 0.202889538 | -2.684650229 | Down | 4.88E-05 |
| 400566 | 3.95122706 | 0.614570543 | -2.684650229 | Down | 1.28E-15 |
| 3698 | 0.44084245 | 0.068568266 | -2.684650229 | Down | 0.00048076 |
| 644150 | 0.57835918 | 0.089957503 | -2.684650229 | Down | 4.88E-05 |
| 220108 | 4.45452024 | 0.692852343 | -2.684650229 | Down | 2.29E-20 |
| 56981 | 0.84094308 | 0.130799581 | -2.684650229 | Down | 4.88E-05 |
| 146556 | 3.25427451 | 0.506167128 | -2.684650229 | Down | 1.03E-13 |
| 60677 | 3.17004623 | 0.493066332 | -2.684650229 | Down | 3.29E-23 |
| 89801 | 3.50545449 | 0.545235451 | -2.684650229 | Down | 2.91E-22 |
| 64388 | 3.69694298 | 0.57501941 | -2.684650229 | Down | 1.17E-32 |
| 2788 | 8.38784273 | 1.304638024 | -2.684650229 | Down | 3.85E-73 |
| 8613 | 38.3162667 | 5.996559201 | -2.67575017 | Down | 1.09E-253 |
| 285671 | 1.69032705 | 0.265369174 | -2.671229714 | Down | 3.21E-18 |
| 23216 | 38.0351915 | 5.971490676 | -2.671171845 | Down | 0 |
| 658 | 4.08774338 | 0.642358859 | -2.669853227 | Down | 5.44E-47 |
| 23034 | 16.6952361 | 2.6246852 | -2.669220189 | Down | 1.13E-218 |
| 51308 | 23.9256619 | 3.785040135 | -2.660178331 | Down | 2.08E-101 |
| 343450 | 1.48036722 | 0.234366773 | -2.659115137 | Down | 6.43E-19 |
| 6495 | 7.17950848 | 1.139299885 | -2.655737537 | Down | 6.72E-40 |
| 84163 | 2.06748614 | 0.328417083 | -2.65427658 | Down | 4.53E-16 |
| 2257 | 2.87141868 | 0.4565429 | -2.65294137 | Down | 1.85E-22 |
| 84867 | 1.02143736 | 0.162748474 | -2.649884811 | Down | 1.04E-07 |
| 23460 | 3.45090741 | 0.550514097 | -2.648124353 | Down | 9.15E-38 |
| 482 | 7.52428896 | 1.20313435 | -2.644757502 | Down | 3.79E-51 |
| 286411 | 1.97942838 | 0.316675162 | -2.644008245 | Down | 9.67E-07 |
| 345462 | 2.10033852 | 0.336018747 | -2.644008245 | Down | 2.93E-12 |
| 3815 | 11.8451568 | 1.901060466 | -2.639420975 | Down | 2.38E-122 |
| 56978 | 6.42435251 | 1.031854492 | -2.638311515 | Down | 1.79E-53 |
| 114044 | 0.97881377 | 0.157493661 | -2.635740629 | Down | 9.13E-06 |
| 23769 | 0.6975593 | 0.112239092 | -2.635740629 | Down | 9.13E-06 |
| 9099 | 10.0635802 | 1.620602603 | -2.634541381 | Down | 1.71E-75 |
| 22998 | 25.7428158 | 4.149831521 | -2.633045195 | Down | 0 |
| 7225 | 6.06260334 | 0.979848248 | -2.629307195 | Down | 1.71E-56 |
| 220965 | 1.65145027 | 0.267139798 | -2.628066701 | Down | 5.78E-13 |
| 58494 | 39.1204931 | 6.328733472 | -2.627935838 | Down | 2.19E-131 |
| 85458 | 20.4033903 | 3.301291559 | -2.627706432 | Down | 4.85E-237 |
| 84940 | 64.1078796 | 10.38283069 | -2.626301869 | Down | 0 |
| 577 | 0.31867297 | 0.051721175 | -2.623249685 | Down | 8.77E-05 |
| 10389 | 0.42790842 | 0.069450278 | -2.623249685 | Down | 8.77E-05 |
| 2258 | 8.05843527 | 1.307898015 | -2.623249685 | Down | 8.16E-44 |
| 151393 | 0.90496702 | 0.146877716 | -2.623249685 | Down | 8.77E-05 |
| 56911 | 8.76941341 | 1.424879495 | -2.621640432 | Down | 7.86E-36 |
| 280 | 14.1939536 | 2.308066354 | -2.620519892 | Down | 4.35E-62 |
| 9452 | 16.3708482 | 2.665666186 | -2.618561039 | Down | 3.30E-60 |
| 79750 | 10.8134781 | 1.762710192 | -2.616963422 | Down | 1.59E-36 |
| 10752 | 1.69499669 | 0.276343826 | -2.616746165 | Down | 6.85E-27 |
| 9353 | 2.5245281 | 0.412296133 | -2.614260901 | Down | 6.03E-26 |
| 1780 | 5.41203485 | 0.885667803 | -2.611333552 | Down | 1.07E-33 |
| 339453 | 3.98553942 | 0.652534167 | -2.610649648 | Down | 4.26E-10 |
| 115560 | 1.4534272 | 0.237962999 | -2.610649648 | Down | 4.26E-10 |
| 1511 | 54.4351372 | 8.913800335 | -2.610425644 | Down | 1.79E-99 |
| 26278 | 3.32107048 | 0.545297255 | -2.606533539 | Down | 1.73E-102 |
| 89927 | 37.9655631 | 6.242879234 | -2.604407942 | Down | 9.16E-166 |
| 63976 | 0.6091966 | 0.10032769 | -2.602188069 | Down | 9.23E-12 |
| 25850 | 3.68929321 | 0.608961995 | -2.598920355 | Down | 8.27E-24 |
| 4254 | 12.217004 | 2.025413698 | -2.59260202 | Down | 1.01E-129 |
| 57731 | 0.90806509 | 0.151028571 | -2.58797421 | Down | 9.96E-17 |
| 9486 | 6.38138376 | 1.061602389 | -2.587625775 | Down | 3.09E-36 |
| 8999 | 0.92762353 | 0.154587628 | -2.585114556 | Down | 7.56E-10 |
| 5662 | 13.9849004 | 2.334927302 | -2.582420447 | Down | 5.08E-114 |
| 79625 | 1.82266576 | 0.304652315 | -2.580814418 | Down | 1.63E-11 |
| 197257 | 6.53688813 | 1.093144683 | -2.580119646 | Down | 1.51E-27 |
| 51161 | 9.67824916 | 1.61973481 | -2.578988453 | Down | 5.80E-51 |
| 322 | 32.2816148 | 5.40729032 | -2.577735025 | Down | 4.70E-165 |
| 100125288 | 1.53838431 | 0.257685083 | -2.577735025 | Down | 3.29E-07 |
| 9414 | 18.902082 | 3.166980945 | -2.577365056 | Down | 2.58E-168 |
| 70 | 53.0835355 | 8.901304622 | -2.576175754 | Down | 0 |
| 9331 | 2.25514328 | 0.378521326 | -2.574772611 | Down | 2.27E-22 |
| 911 | 3.01321714 | 0.506167128 | -2.573618917 | Down | 1.76E-16 |
| 2781 | 14.1938307 | 2.385675492 | -2.572794284 | Down | 9.00E-89 |
| 25959 | 133.890151 | 22.53811233 | -2.570611248 | Down | 0 |
| 9023 | 16.3233276 | 2.750493074 | -2.569173012 | Down | 1.26E-44 |
| 493 | 84.8106246 | 14.29611004 | -2.568622365 | Down | 0 |
| 104 | 24.7300781 | 4.169774116 | -2.568225658 | Down | 0 |
| 23743 | 21.6712149 | 3.657203535 | -2.566967209 | Down | 4.42E-84 |
| 5152 | 11.5258188 | 1.946040609 | -2.566255528 | Down | 5.87E-47 |
| 1375 | 6.31105128 | 1.068594223 | -2.562166223 | Down | 6.82E-37 |
| 7053 | 0.64185915 | 0.108910073 | -2.559119347 | Down | 0.00015674 |
| 60482 | 0.66793273 | 0.113334214 | -2.559119347 | Down | 6.31E-08 |
| 23245 | 1.62988116 | 0.276556745 | -2.559119347 | Down | 3.10E-16 |
| 3818 | 1.14703401 | 0.194627681 | -2.559119347 | Down | 3.07E-06 |
| 1268 | 0.19155596 | 0.03250304 | -2.559119347 | Down | 0.00015674 |
| 132724 | 0.64962455 | 0.1102277 | -2.559119347 | Down | 0.00015674 |
| 27022 | 1.2463179 | 0.211474081 | -2.559119347 | Down | 3.07E-06 |
| 79570 | 1.37846526 | 0.233896723 | -2.559119347 | Down | 6.31E-08 |
| 5549 | 79.1360818 | 13.47784147 | -2.553746175 | Down | 0 |
| 64094 | 43.9144855 | 7.494090307 | -2.550871632 | Down | 1.66E-246 |
| 147923 | 2.1541918 | 0.367750083 | -2.550349138 | Down | 1.24E-13 |
| 6542 | 1.97192258 | 0.337221722 | -2.54783354 | Down | 7.56E-30 |
| 2294 | 33.3505978 | 5.718384605 | -2.54403306 | Down | 6.59E-163 |
| 7275 | 3.90878248 | 0.670470209 | -2.543474166 | Down | 5.23E-49 |
| 6707 | 34.4570242 | 5.913839063 | -2.542631224 | Down | 8.41E-66 |
| 6700 | 33.1099599 | 5.706155867 | -2.536674201 | Down | 7.39E-44 |
| 10840 | 8.64751857 | 1.492747601 | -2.534315956 | Down | 1.74E-52 |
| 51559 | 17.9070808 | 3.091731125 | -2.534043405 | Down | 3.28E-244 |
| 127833 | 0.55391907 | 0.095729008 | -2.532647136 | Down | 2.36E-09 |
| 5164 | 65.9267699 | 11.39472829 | -2.532497873 | Down | 7.20E-285 |
| 79695 | 13.2697184 | 2.295266831 | -2.531403969 | Down | 9.95E-70 |
| 389524 | 3.63825167 | 0.630273378 | -2.529195711 | Down | 4.45E-26 |
| 1299 | 4.05442007 | 0.703949133 | -2.525952483 | Down | 1.90E-20 |
| 30818 | 6.19138568 | 1.075561722 | -2.525172015 | Down | 1.98E-35 |
| 64112 | 21.3271445 | 3.709239625 | -2.523495437 | Down | 4.22E-98 |
| 3800 | 1.03620579 | 0.180652738 | -2.520019527 | Down | 8.49E-15 |
| 2316 | 971.069395 | 169.7152805 | -2.516457933 | Down | 0 |
| 10252 | 31.5239018 | 5.514268206 | -2.515204767 | Down | 1.39E-147 |
| 9227 | 0.51130991 | 0.089469803 | -2.514725228 | Down | 5.43E-06 |
| 127733 | 2.52282923 | 0.441448586 | -2.514725228 | Down | 1.68E-15 |
| 10371 | 8.43270567 | 1.481202976 | -2.509226247 | Down | 2.30E-90 |
| 340562 | 1.88842937 | 0.332518551 | -2.505680088 | Down | 4.14E-09 |
| 196500 | 5.14685259 | 0.906902209 | -2.504671563 | Down | 2.63E-22 |
| 3776 | 0.80339054 | 0.141845 | -2.501786172 | Down | 1.03E-06 |
| 255743 | 8.62127174 | 1.526451653 | -2.497718803 | Down | 9.60E-75 |
| 9910 | 19.4722983 | 3.460191679 | -2.49249931 | Down | 8.46E-107 |
| 2822 | 0.94018593 | 0.16712655 | -2.492005151 | Down | 1.96E-07 |
| 80017 | 32.9297399 | 5.863491514 | -2.489559214 | Down | 3.24E-192 |
| 83394 | 12.2434626 | 2.181239857 | -2.488791305 | Down | 9.77E-163 |
| 10810 | 1.77799766 | 0.316773468 | -2.488730019 | Down | 2.27E-17 |
| 5308 | 12.724579 | 2.267908723 | -2.48818344 | Down | 7.07E-56 |
| 117854 | 1.55792784 | 0.277953906 | -2.486710852 | Down | 3.06E-11 |
| 83857 | 5.32135308 | 0.95024687 | -2.485418858 | Down | 5.92E-88 |
| 825 | 20.7079524 | 3.699806517 | -2.484663176 | Down | 3.00E-149 |
| 2852 | 5.62569972 | 1.005860224 | -2.48360271 | Down | 2.44E-32 |
| 399959 | 62.4567126 | 11.16985953 | -2.483245592 | Down | 0 |
| 10370 | 49.4771191 | 8.879572637 | -2.478199352 | Down | 2.35E-176 |
| 51085 | 3.7169017 | 0.667065892 | -2.478199352 | Down | 6.34E-24 |
| 51716 | 0.90452886 | 0.162334224 | -2.478199352 | Down | 5.12E-05 |
| 6387 | 71.9725714 | 12.92312415 | -2.477492322 | Down | 0 |
| 9759 | 5.28167013 | 0.950078406 | -2.474875715 | Down | 2.21E-88 |
| 340526 | 4.56644909 | 0.821874209 | -2.474083243 | Down | 1.19E-41 |
| 4248 | 0.95209604 | 0.171973417 | -2.468921538 | Down | 2.74E-10 |
| 284723 | 6.43646737 | 1.163360018 | -2.467971459 | Down | 1.10E-37 |
| 55084 | 11.0536718 | 1.999977433 | -2.466470059 | Down | 4.13E-127 |
| 2651 | 1.72186785 | 0.312020832 | -2.464260161 | Down | 3.50E-16 |
| 1583 | 1.40304051 | 0.254599035 | -2.462257808 | Down | 1.81E-06 |
| 775 | 6.31465314 | 1.146756672 | -2.461144188 | Down | 4.36E-159 |
| 144571 | 5.85680131 | 1.066492056 | -2.457239733 | Down | 7.63E-25 |
| 115557 | 71.1859797 | 12.96676417 | -2.456774623 | Down | 0 |
| 6355 | 13.4336452 | 2.449039033 | -2.455563155 | Down | 4.47E-32 |
| 23090 | 4.39306795 | 0.801798988 | -2.453916315 | Down | 5.39E-40 |
| 66002 | 2.08273569 | 0.380285564 | -2.453324683 | Down | 6.60E-08 |
| 54413 | 1.84478514 | 0.336838305 | -2.453324683 | Down | 1.56E-14 |
| 10396 | 0.86906605 | 0.158682293 | -2.453324683 | Down | 1.56E-14 |
| 59277 | 8.92292363 | 1.633176593 | -2.449835697 | Down | 5.53E-60 |
| 90187 | 1.14286515 | 0.209503196 | -2.447611032 | Down | 2.46E-09 |
| 782 | 19.721592 | 3.617715259 | -2.446625248 | Down | 8.10E-133 |
| 55544 | 82.6678783 | 15.1722426 | -2.445892515 | Down | 0 |
| 59 | 3360.59069 | 619.0504687 | -2.440585902 | Down | 0 |
| 201229 | 12.287578 | 2.263910234 | -2.440311917 | Down | 2.16E-34 |
| 79864 | 2.57569511 | 0.475737981 | -2.436722716 | Down | 5.36E-15 |
| 8605 | 12.8750564 | 2.386047382 | -2.431884159 | Down | 7.02E-61 |
| 93035 | 0.11143981 | 0.020799905 | -2.421615823 | Down | 0.00049214 |
| 4004 | 2.75872564 | 0.514907848 | -2.421615823 | Down | 1.15E-07 |
| 163688 | 2.48282524 | 0.463411867 | -2.421615823 | Down | 3.16E-06 |
| 11251 | 1.0735751 | 0.200379564 | -2.421615823 | Down | 6.01E-07 |
| 9220 | 1.49550193 | 0.279130939 | -2.421615823 | Down | 6.01E-07 |
| 146 | 0.87887384 | 0.164039159 | -2.421615823 | Down | 1.68E-05 |
| 2047 | 2.68185671 | 0.500560492 | -2.421615823 | Down | 3.47E-23 |
| 27151 | 0.52172738 | 0.097378846 | -2.421615823 | Down | 6.01E-07 |
| 161176 | 0.47696237 | 0.089023593 | -2.421615823 | Down | 0.00049214 |
| 55753 | 0.41532884 | 0.077519879 | -2.421615823 | Down | 0.00049214 |
| 440556 | 1.05316327 | 0.196569759 | -2.421615823 | Down | 2.22E-08 |
| 201501 | 3.82000963 | 0.716868277 | -2.413796319 | Down | 3.79E-27 |
| 3569 | 18.6996891 | 3.514906758 | -2.411455957 | Down | 7.06E-41 |
| 375775 | 3.38022021 | 0.636974364 | -2.407810024 | Down | 2.09E-30 |
| 5727 | 0.9118921 | 0.172012578 | -2.406349067 | Down | 1.61E-14 |
| 349565 | 6.62383733 | 1.251968935 | -2.403468477 | Down | 2.07E-23 |
| 54507 | 30.5643073 | 5.777606791 | -2.403303945 | Down | 3.19E-228 |
| 120892 | 1.7950725 | 0.339786086 | -2.401343428 | Down | 7.22E-31 |
| 50652 | 2.78116311 | 0.526901685 | -2.400082662 | Down | 6.62E-20 |
| 2898 | 1.07843919 | 0.204432549 | -2.39924801 | Down | 2.82E-10 |
| 80760 | 11.791402 | 2.237740923 | -2.397620342 | Down | 7.25E-133 |
| 55160 | 17.2924066 | 3.2924516 | -2.392904527 | Down | 3.56E-138 |
| 27295 | 642.970096 | 122.4969046 | -2.392006346 | Down | 0 |
| 54996 | 9.48353106 | 1.807142143 | -2.391714338 | Down | 7.78E-28 |
| 217 | 98.7346886 | 18.82941717 | -2.390568694 | Down | 0 |
| 154661 | 0.83817412 | 0.159998195 | -2.389194346 | Down | 2.00E-07 |
| 2905 | 0.71435294 | 0.136750594 | -2.385089947 | Down | 1.05E-06 |
| 284406 | 1.16437345 | 0.222899286 | -2.385089947 | Down | 1.05E-06 |
| 9912 | 2.86278284 | 0.548030575 | -2.385089947 | Down | 6.16E-23 |
| 1612 | 8.14171474 | 1.558906103 | -2.384798641 | Down | 2.67E-86 |
| 9252 | 5.68825668 | 1.091815045 | -2.381258086 | Down | 3.72E-40 |
| 79746 | 4.81648367 | 0.925422851 | -2.379795648 | Down | 1.88E-15 |
| 30819 | 3.9518884 | 0.760130473 | -2.378223228 | Down | 1.97E-19 |
| 253190 | 5.51379262 | 1.060961666 | -2.377672476 | Down | 9.51E-15 |
| 79633 | 1.88105551 | 0.361951913 | -2.377672476 | Down | 1.53E-54 |
| 100188953 | 4.18001054 | 0.805627262 | -2.375322171 | Down | 4.82E-14 |
| 6330 | 5.69942418 | 1.098815072 | -2.374867564 | Down | 4.07E-47 |
| 3960 | 1.77089528 | 0.341929944 | -2.372706223 | Down | 2.93E-05 |
| 56121 | 1.7061959 | 0.329437588 | -2.372706223 | Down | 2.51E-09 |
| 137872 | 18.670434 | 3.615555803 | -2.368466117 | Down | 4.12E-63 |
| 643763 | 0.58798824 | 0.113887604 | -2.368176564 | Down | 1.29E-08 |
| 83604 | 31.094617 | 6.041043495 | -2.363795169 | Down | 1.03E-219 |
| 126668 | 1.48237415 | 0.288209041 | -2.362722134 | Down | 6.69E-08 |
| 1901 | 16.9521028 | 3.297886285 | -2.361850679 | Down | 3.97E-91 |
| 3632 | 50.1632348 | 9.774641995 | -2.359514617 | Down | 5.49E-256 |
| 12 | 33.4023648 | 6.509500274 | -2.359331545 | Down | 1.94E-93 |
| 933 | 1.59295994 | 0.310634073 | -2.358421997 | Down | 1.65E-10 |
| 222698 | 5.24178444 | 1.022834368 | -2.357485486 | Down | 2.19E-16 |
| 23371 | 55.1497977 | 10.76169998 | -2.357449602 | Down | 0 |
| 51285 | 58.0770604 | 11.33751538 | -2.356863924 | Down | 1.13E-259 |
| 85002 | 2.75505438 | 0.538139949 | -2.356027482 | Down | 4.21E-13 |
| 51177 | 266.661097 | 52.12737879 | -2.354894144 | Down | 0 |
| 6236 | 67.8899869 | 13.28380073 | -2.353530821 | Down | 3.80E-174 |
| 11341 | 16.1054118 | 3.151479592 | -2.353444321 | Down | 1.17E-26 |
| 4303 | 10.6535599 | 2.085024484 | -2.35319936 | Down | 1.23E-62 |
| 23111 | 11.9498674 | 2.340909375 | -2.351853618 | Down | 9.09E-105 |
| 399948 | 1.43491803 | 0.281214293 | -2.351226496 | Down | 5.58E-15 |
| 64881 | 1.24257833 | 0.244130011 | -2.347615242 | Down | 1.09E-11 |
| 404201 | 1.89158595 | 0.371640874 | -2.347615242 | Down | 1.82E-06 |
| 56963 | 30.7456084 | 6.051341338 | -2.345053486 | Down | 4.00E-171 |
| 493829 | 2.66728416 | 0.525887925 | -2.342544252 | Down | 5.55E-11 |
| 3067 | 8.89447597 | 1.757068934 | -2.339738816 | Down | 2.81E-38 |
| 108 | 5.45871897 | 1.0789169 | -2.338978674 | Down | 7.40E-63 |
| 5140 | 5.08026034 | 1.005682914 | -2.336726926 | Down | 1.63E-54 |
| 10335 | 51.5157892 | 10.21795324 | -2.333908437 | Down | 0 |
| 8905 | 43.6868179 | 8.683992149 | -2.330767697 | Down | 2.48E-171 |
| 116 | 5.73454837 | 1.143521595 | -2.326196258 | Down | 1.06E-32 |
| 3489 | 203.503328 | 40.5939749 | -2.325714867 | Down | 0 |
| 22915 | 2.71920865 | 0.542736659 | -2.324862591 | Down | 1.48E-24 |
| 84033 | 9.18509839 | 1.835092809 | -2.323442147 | Down | 0 |
| 140686 | 2.18687247 | 0.437328399 | -2.32208015 | Down | 5.09E-05 |
| 80243 | 0.6391872 | 0.127823967 | -2.32208015 | Down | 6.01E-07 |
| 1013 | 3.0596327 | 0.611862048 | -2.32208015 | Down | 2.20E-16 |
| 154043 | 5.75849318 | 1.151577258 | -2.32208015 | Down | 3.65E-33 |
| 56853 | 1.07887147 | 0.215751553 | -2.32208015 | Down | 7.46E-09 |
| 7434 | 6.93101062 | 1.38605603 | -2.32208015 | Down | 5.10E-48 |
| 55713 | 0.94343075 | 0.188666263 | -2.32208015 | Down | 6.01E-07 |
| 7743 | 6.91957327 | 1.385364844 | -2.320417101 | Down | 6.72E-40 |
| 3897 | 10.0386534 | 2.015806056 | -2.316137011 | Down | 6.33E-87 |
| 27145 | 21.0211207 | 4.226309349 | -2.314369308 | Down | 1.54E-165 |
| 4023 | 4.25218255 | 0.855904703 | -2.312681452 | Down | 1.63E-28 |
| 7538 | 608.663093 | 122.6357382 | -2.311264422 | Down | 0 |
| 59352 | 0.83568414 | 0.168624792 | -2.309141094 | Down | 3.14E-06 |
| 678655 | 20.6159116 | 4.162389582 | -2.30827435 | Down | 1.10E-83 |
| 5915 | 7.45419914 | 1.50605057 | -2.307283148 | Down | 6.73E-40 |
| 9145 | 8.92249036 | 1.806599214 | -2.304169952 | Down | 3.56E-68 |
| 408 | 25.9396981 | 5.258258877 | -2.302504612 | Down | 1.45E-97 |
| 1191 | 279.31827 | 56.62236357 | -2.302466067 | Down | 0 |
| 3083 | 0.88143255 | 0.178822538 | -2.30132159 | Down | 0.00027434 |
| 9514 | 1.00299248 | 0.203484273 | -2.30132159 | Down | 0.00027434 |
| 81575 | 12.2339287 | 2.492688783 | -2.295113102 | Down | 1.72E-98 |
| 57088 | 6.14622525 | 1.253394323 | -2.293860276 | Down | 6.99E-37 |
| 387590 | 1.74775023 | 0.356794731 | -2.292332806 | Down | 1.66E-05 |
| 55070 | 3.50011599 | 0.715503853 | -2.29037129 | Down | 3.24E-15 |
| 5325 | 11.7382252 | 2.408289915 | -2.285133315 | Down | 2.07E-68 |
| 5264 | 44.0380191 | 9.052570352 | -2.282350188 | Down | 6.09E-127 |
| 644538 | 27.7154651 | 5.705523793 | -2.280259974 | Down | 4.06E-72 |
| 89853 | 3.59874939 | 0.741285856 | -2.279393751 | Down | 2.23E-30 |
| 91522 | 2.19289017 | 0.452129697 | -2.27802497 | Down | 1.23E-12 |
| 83439 | 29.7021795 | 6.124603918 | -2.277880346 | Down | 2.38E-137 |
| 1287 | 49.7910068 | 10.27182664 | -2.277192427 | Down | 0 |
| 10365 | 68.2509579 | 14.14443982 | -2.270614254 | Down | 4.71E-187 |
| 534 | 2.64754536 | 0.549062647 | -2.26961273 | Down | 2.20E-08 |
| 254268 | 0.69185364 | 0.143480446 | -2.26961273 | Down | 8.80E-05 |
| 26206 | 1.39954862 | 0.290246158 | -2.26961273 | Down | 5.41E-06 |
| 139221 | 0.71056183 | 0.147360256 | -2.26961273 | Down | 5.41E-06 |
| 9851 | 7.86860654 | 1.633563923 | -2.26808526 | Down | 2.13E-62 |
| 221264 | 2.49394923 | 0.518489249 | -2.266046115 | Down | 1.36E-27 |
| 10979 | 82.5489651 | 17.16291282 | -2.265955706 | Down | 0 |
| 5241 | 0.61705658 | 0.128589823 | -2.2626263 | Down | 9.41E-15 |
| 23302 | 0.89896468 | 0.187823569 | -2.258886323 | Down | 4.79E-10 |
| 57619 | 5.16099521 | 1.078402 | -2.258754215 | Down | 8.10E-95 |
| 57134 | 12.9560995 | 2.709678197 | -2.257438016 | Down | 1.38E-69 |
| 6252 | 2.52552945 | 0.528653247 | -2.256192214 | Down | 3.18E-15 |
| 9256 | 4.07165079 | 0.85282449 | -2.255293059 | Down | 6.60E-53 |
| 9639 | 5.78330737 | 1.21241105 | -2.254015875 | Down | 1.57E-54 |
| 9086 | 37.9837048 | 7.970493598 | -2.252639651 | Down | 6.97E-88 |
| 5087 | 25.5939949 | 5.376899111 | -2.250959047 | Down | 3.13E-280 |
| 800 | 1544.43115 | 325.18109 | -2.247760288 | Down | 0 |
| 11185 | 43.8277908 | 9.228500793 | -2.247677759 | Down | 4.20E-184 |
| 11149 | 11.0946819 | 2.343540011 | -2.243106976 | Down | 7.38E-101 |
| 8490 | 75.4223347 | 15.93332132 | -2.24294478 | Down | 0 |
| 85457 | 9.98735949 | 2.112211677 | -2.241348878 | Down | 3.73E-72 |
| 55273 | 4.12525877 | 0.873492141 | -2.23961799 | Down | 1.23E-16 |
| 57165 | 4.14365047 | 0.877386444 | -2.23961799 | Down | 1.23E-16 |
| 10763 | 22.2628216 | 4.721919007 | -2.237191252 | Down | 1.04E-201 |
| 8671 | 0.44676468 | 0.094758277 | -2.237191252 | Down | 5.84E-07 |
| 92340 | 0.8131836 | 0.172475311 | -2.237191252 | Down | 0.00047434 |
| 10858 | 0.82727826 | 0.175464772 | -2.237191252 | Down | 0.00047434 |
| 23409 | 1.46734154 | 0.311221462 | -2.237191252 | Down | 0.00047434 |
| 100131827 | 0.56005767 | 0.118787592 | -2.237191252 | Down | 0.00047434 |
| 2239 | 24.0153893 | 5.102557156 | -2.234666851 | Down | 9.26E-146 |
| 56952 | 8.19144487 | 1.740836478 | -2.234337255 | Down | 3.84E-27 |
| 3043 | 877.094561 | 186.4118121 | -2.23423911 | Down | 0 |
| 59271 | 21.6165984 | 4.600281483 | -2.232345472 | Down | 3.72E-69 |
| 340156 | 0.7728902 | 0.164504317 | -2.232138025 | Down | 1.25E-08 |
| 84460 | 4.97549974 | 1.059627232 | -2.231284611 | Down | 7.64E-51 |
| 388121 | 12.7288541 | 2.711513544 | -2.230932262 | Down | 4.64E-48 |
| 7745 | 0.50980802 | 0.108747581 | -2.228970745 | Down | 9.27E-06 |
| 284348 | 3.68496818 | 0.786876367 | -2.227443276 | Down | 2.09E-16 |
| 124976 | 5.94434507 | 1.273739492 | -2.222447622 | Down | 3.40E-43 |
| 8997 | 2.58029095 | 0.553102114 | -2.221915988 | Down | 1.91E-46 |
| 50506 | 1.82149118 | 0.390512805 | -2.221678253 | Down | 3.75E-20 |
| 152503 | 23.3285751 | 5.003883948 | -2.220977955 | Down | 8.89E-199 |
| 2596 | 4.19467323 | 0.900708046 | -2.219426973 | Down | 1.05E-15 |
| 5532 | 34.9713082 | 7.522585455 | -2.216871268 | Down | 9.47E-178 |
| 212 | 1.0023037 | 0.215858045 | -2.215164946 | Down | 0.00015135 |
| 57689 | 0.50090461 | 0.107875777 | -2.215164946 | Down | 0.00015135 |
| 2785 | 2.25129412 | 0.484843014 | -2.215164946 | Down | 0.00015135 |
| 84529 | 12.7612707 | 2.752612914 | -2.212898339 | Down | 1.40E-54 |
| 8218 | 4.16584923 | 0.899199659 | -2.211897232 | Down | 2.50E-38 |
| 79689 | 10.0828442 | 2.176495922 | -2.211823439 | Down | 2.30E-73 |
| 4256 | 1135.50056 | 245.3493916 | -2.210418823 | Down | 0 |
| 94030 | 2.1658479 | 0.468337255 | -2.20931222 | Down | 1.03E-11 |
| 11037 | 19.5390852 | 4.226303922 | -2.208894501 | Down | 2.03E-182 |
| 23345 | 4.27882264 | 0.927355828 | -2.206018964 | Down | 2.66E-190 |
| 3572 | 1.04040003 | 0.225799462 | -2.204024388 | Down | 9.95E-07 |
| 114780 | 0.44056294 | 0.095615986 | -2.204024388 | Down | 9.95E-07 |
| 3752 | 1.24708923 | 0.270657506 | -2.204024388 | Down | 9.95E-07 |
| 23530 | 13.3899692 | 2.913652934 | -2.200251695 | Down | 5.70E-101 |
| 340419 | 1.4886135 | 0.324152773 | -2.199223402 | Down | 7.05E-09 |
| 7869 | 32.004444 | 6.974934589 | -2.198020655 | Down | 7.65E-150 |
| 53344 | 1.88784661 | 0.411439368 | -2.197989802 | Down | 2.85E-22 |
| 5906 | 82.4051967 | 17.97344766 | -2.196868148 | Down | 7.83E-250 |
| 11076 | 6.43289335 | 1.404021207 | -2.195903044 | Down | 5.77E-63 |
| 3653 | 5.19179055 | 1.134318509 | -2.194406386 | Down | 1.39E-38 |
| 9920 | 1.29066358 | 0.282133352 | -2.193663877 | Down | 2.99E-15 |
| 4208 | 22.5828608 | 4.944242389 | -2.191406881 | Down | 2.29E-231 |
| 9751 | 3.12785696 | 0.68597099 | -2.188955067 | Down | 3.01E-26 |
| 4058 | 0.84921266 | 0.186474107 | -2.18715057 | Down | 1.58E-05 |
| 79836 | 1.01859916 | 0.223668788 | -2.18715057 | Down | 1.58E-05 |
| 3293 | 3.51255026 | 0.771302291 | -2.18715057 | Down | 1.08E-07 |
| 3781 | 1.58379006 | 0.347776063 | -2.18715057 | Down | 1.08E-07 |
| 648791 | 2.29448848 | 0.503834561 | -2.18715057 | Down | 1.08E-07 |
| 5834 | 148.151024 | 32.55349373 | -2.18618431 | Down | 0 |
| 1730 | 4.85888066 | 1.067796278 | -2.185987575 | Down | 9.29E-40 |
| 22899 | 6.20695239 | 1.365010608 | -2.184972916 | Down | 2.56E-42 |
| 2009 | 23.7418045 | 5.224719808 | -2.184004014 | Down | 4.53E-170 |
| 3707 | 37.935905 | 8.362846962 | -2.181497887 | Down | 0 |
| 11034 | 1276.65972 | 281.4497961 | -2.181424531 | Down | 0 |
| 253639 | 1.89988605 | 0.419082204 | -2.180607724 | Down | 3.59E-08 |
| 8515 | 0.8308786 | 0.183277537 | -2.180607724 | Down | 3.59E-08 |
| 6907 | 16.9217718 | 3.737522483 | -2.178726473 | Down | 5.78E-153 |
| 29970 | 28.1851695 | 6.229749269 | -2.177690241 | Down | 1.45E-94 |
| 8434 | 4.40887428 | 0.974925396 | -2.177046611 | Down | 4.33E-32 |
| 253827 | 80.9740069 | 17.92054943 | -2.175844 | Down | 0 |
| 3690 | 3.83012284 | 0.848921085 | -2.17368831 | Down | 6.27E-31 |
| 9379 | 1.44371451 | 0.320265747 | -2.172444071 | Down | 1.94E-16 |
| 283078 | 1.80286271 | 0.400593935 | -2.170077056 | Down | 9.83E-12 |
| 6711 | 41.6608268 | 9.263437856 | -2.16907186 | Down | 0 |
| 6327 | 2.72930621 | 0.607153743 | -2.168400479 | Down | 1.56E-22 |
| 116987 | 5.42211203 | 1.208623698 | -2.165489787 | Down | 8.56E-40 |
| 10267 | 105.68337 | 23.57435813 | -2.164457984 | Down | 9.62E-151 |
| 3213 | 11.8298005 | 2.639120151 | -2.16429681 | Down | 3.70E-68 |
| 54206 | 38.1024775 | 8.505161207 | -2.163474322 | Down | 1.90E-186 |
| 54768 | 0.35354057 | 0.078998861 | -2.161972007 | Down | 4.41E-10 |
| 284358 | 6.08369036 | 1.359404421 | -2.161972007 | Down | 8.56E-19 |
| 641371 | 3.45931613 | 0.772986357 | -2.161972007 | Down | 4.41E-10 |
| 2628 | 5.79880005 | 1.296539465 | -2.161088273 | Down | 7.04E-25 |
| 6867 | 80.8454887 | 18.0865543 | -2.160249687 | Down | 0 |
| 1896 | 1.84901961 | 0.414136741 | -2.158581418 | Down | 1.10E-16 |
| 54988 | 0.80717846 | 0.180788921 | -2.158581418 | Down | 0.00025865 |
| 116969 | 1.30084258 | 0.291358027 | -2.158581418 | Down | 0.00025865 |
| 441094 | 4.71388402 | 1.056661318 | -2.157403226 | Down | 2.76E-26 |
| 283807 | 9.4782124 | 2.124872446 | -2.157238751 | Down | 3.02E-23 |
| 57458 | 4.4359819 | 0.994549773 | -2.157138001 | Down | 6.61E-42 |
| 79656 | 6.11646775 | 1.372139805 | -2.156271257 | Down | 4.22E-14 |
| 2861 | 3.51171205 | 0.788511875 | -2.154970164 | Down | 1.25E-17 |
| 2327 | 4.07019858 | 0.914442061 | -2.154135513 | Down | 3.18E-34 |
| 283383 | 2.77072703 | 0.62274351 | -2.153554601 | Down | 1.18E-24 |
| 84866 | 9.72808038 | 2.190388711 | -2.150968233 | Down | 1.37E-39 |
| 83850 | 0.50932653 | 0.114732733 | -2.150313802 | Down | 8.30E-05 |
| 23635 | 28.185893 | 6.360868222 | -2.147677671 | Down | 4.15E-86 |
| 10085 | 1.6282315 | 0.367626428 | -2.146993443 | Down | 6.69E-09 |
| 4504 | 4.42849727 | 1.001897821 | -2.144081848 | Down | 2.68E-05 |
| 148741 | 4.78416092 | 1.083676278 | -2.142332066 | Down | 2.63E-26 |
| 3082 | 2.86793818 | 0.649626261 | -2.142332066 | Down | 7.08E-14 |
| 200132 | 1.27697559 | 0.289877419 | -2.139216093 | Down | 8.73E-06 |
| 100133991 | 4.59062075 | 1.042085144 | -2.139216093 | Down | 2.48E-10 |
| 338811 | 0.71265 | 0.16177376 | -2.139216093 | Down | 8.73E-06 |
| 3977 | 3.12928093 | 0.710548513 | -2.138826122 | Down | 8.45E-51 |
| 257194 | 18.6266889 | 4.230053601 | -2.138623391 | Down | 5.07E-163 |
| 84101 | 0.92335817 | 0.210173202 | -2.135311638 | Down | 2.86E-06 |
| 10602 | 51.0285938 | 11.62620502 | -2.133925631 | Down | 0 |
| 1577 | 2.05894345 | 0.469694629 | -2.132109206 | Down | 9.38E-07 |
| 153201 | 7.19365944 | 1.64104711 | -2.132109206 | Down | 3.62E-39 |
| 785 | 0.43584034 | 0.099425686 | -2.132109206 | Down | 9.38E-07 |
| 56242 | 3.01813351 | 0.688509 | -2.132109206 | Down | 3.12E-12 |
| 2205 | 9.5868413 | 2.189770237 | -2.130276048 | Down | 1.36E-18 |
| 118429 | 34.8061659 | 7.950601278 | -2.130207024 | Down | 0 |
| 10350 | 5.15643179 | 1.179905218 | -2.127702105 | Down | 4.56E-51 |
| 5365 | 11.6229193 | 2.663151871 | -2.125765861 | Down | 4.02E-114 |
| 9586 | 6.92963658 | 1.588980352 | -2.124678407 | Down | 2.41E-91 |
| 51062 | 3.49713082 | 0.80296022 | -2.122771342 | Down | 1.74E-16 |
| 55553 | 0.56545648 | 0.129896309 | -2.122055542 | Down | 3.74E-09 |
| 23467 | 4.63457051 | 1.065615007 | -2.120749344 | Down | 1.83E-42 |
| 147081 | 4.50652886 | 1.036603952 | -2.120151831 | Down | 2.27E-18 |
| 118491 | 1.53969456 | 0.354303321 | -2.119587286 | Down | 4.16E-10 |
| 84182 | 6.83004705 | 1.573507472 | -2.117911487 | Down | 7.39E-30 |
| 7466 | 31.6359481 | 7.310839312 | -2.113455884 | Down | 6.53E-175 |
| 4902 | 8.27717647 | 1.91463218 | -2.112071453 | Down | 2.55E-15 |
| 657 | 12.6571119 | 2.942936311 | -2.104620024 | Down | 2.85E-70 |
| 9590 | 15.6075499 | 3.63057257 | -2.103975087 | Down | 1.58E-198 |
| 10742 | 24.0342646 | 5.599218043 | -2.101795392 | Down | 1.05E-81 |
| 26149 | 1.24187966 | 0.289741382 | -2.099687729 | Down | 6.26E-09 |
| 140883 | 0.32724548 | 0.076349232 | -2.099687729 | Down | 0.00043926 |
| 130940 | 1.71496992 | 0.400117474 | -2.099687729 | Down | 5.19E-07 |
| 2897 | 0.53879337 | 0.125705202 | -2.099687729 | Down | 0.00043926 |
| 7098 | 1.0273277 | 0.239684533 | -2.099687729 | Down | 4.81E-06 |
| 4093 | 1.32771081 | 0.309766538 | -2.099687729 | Down | 4.81E-06 |
| 7093 | 0.32383718 | 0.075554045 | -2.099687729 | Down | 0.0001405 |
| 58480 | 7.15609091 | 1.66957857 | -2.099687729 | Down | 6.71E-48 |
| 147947 | 6.50483621 | 1.517635154 | -2.099687729 | Down | 9.51E-36 |
| 2593 | 58.3185678 | 13.64067784 | -2.096039952 | Down | 1.25E-93 |
| 2995 | 134.108926 | 31.37385493 | -2.095770553 | Down | 2.07E-215 |
| 285343 | 7.01736067 | 1.642157849 | -2.095335704 | Down | 3.33E-40 |
| 3164 | 85.298557 | 19.98279575 | -2.093762895 | Down | 0 |
| 92999 | 28.8793618 | 6.768177599 | -2.093199529 | Down | 6.23E-182 |
| 23022 | 146.524704 | 34.40507458 | -2.090450648 | Down | 0 |
| 51196 | 4.31025421 | 1.012460941 | -2.089906708 | Down | 7.64E-53 |
| 64122 | 15.8058273 | 3.71306968 | -2.08977226 | Down | 2.62E-35 |
| 6920 | 78.5950383 | 18.47478103 | -2.088880974 | Down | 2.02E-186 |
| 7414 | 124.912889 | 29.36249122 | -2.088876065 | Down | 0 |
| 339456 | 25.1917534 | 5.93451898 | -2.085748538 | Down | 1.99E-37 |
| 744 | 3.36116882 | 0.793792398 | -2.082129357 | Down | 6.12E-30 |
| 5213 | 54.7174544 | 12.92892482 | -2.081398809 | Down | 2.09E-236 |
| 222166 | 23.6161593 | 5.591344623 | -2.078507184 | Down | 7.76E-204 |
| 2675 | 3.04904999 | 0.722314172 | -2.077661422 | Down | 1.52E-16 |
| 9853 | 14.9968058 | 3.557092861 | -2.075884704 | Down | 5.41E-126 |
| 84281 | 4.25838151 | 1.011581591 | -2.07369252 | Down | 6.56E-27 |
| 6649 | 332.025391 | 78.94220932 | -2.072424774 | Down | 0 |
| 4211 | 43.8268217 | 10.42567439 | -2.071673352 | Down | 4.62E-206 |
| 155038 | 4.73409043 | 1.127611477 | -2.069817199 | Down | 4.93E-29 |
| 6855 | 4.26947975 | 1.018575528 | -2.067507323 | Down | 8.54E-17 |
| 80852 | 0.98027183 | 0.234024732 | -2.066520865 | Down | 2.64E-06 |
| 2145 | 18.6598952 | 4.455490933 | -2.06628457 | Down | 1.93E-128 |
| 91608 | 4.01153287 | 0.958753145 | -2.06492231 | Down | 2.44E-20 |
| 6558 | 5.77230499 | 1.381260735 | -2.063161853 | Down | 1.59E-59 |
| 4026 | 129.435754 | 31.04696259 | -2.059712147 | Down | 0 |
| 4223 | 3.45878185 | 0.830020158 | -2.059045744 | Down | 1.72E-13 |
| 140465 | 173.012063 | 41.51851887 | -2.059045744 | Down | 2.54E-220 |
| 162494 | 0.58648157 | 0.140740742 | -2.059045744 | Down | 2.47E-05 |
| 818 | 80.810637 | 19.47128016 | -2.053197466 | Down | 0 |
| 57669 | 2.1734971 | 0.523813322 | -2.052893517 | Down | 2.99E-22 |
| 79960 | 13.8855061 | 3.34696545 | -2.052654195 | Down | 7.39E-118 |
| 63920 | 2.47635566 | 0.597230327 | -2.051859203 | Down | 1.35E-11 |
| 56181 | 56.7107238 | 13.69034058 | -2.050463232 | Down | 3.09E-174 |
| 5567 | 16.1120119 | 3.892316423 | -2.04943575 | Down | 6.68E-107 |
| 25999 | 54.0817862 | 13.08024125 | -2.047753652 | Down | 4.04E-263 |
| 79844 | 4.0459898 | 0.978926449 | -2.047220309 | Down | 7.91E-17 |
| 91694 | 12.2689055 | 2.969212528 | -2.046854282 | Down | 3.01E-65 |
| 8497 | 2.80209484 | 0.679036496 | -2.044944764 | Down | 4.93E-22 |
| 4133 | 1.49672509 | 0.362703983 | -2.044944764 | Down | 4.93E-22 |
| 596 | 2.40611794 | 0.583823083 | -2.0431042 | Down | 3.67E-24 |
| 60468 | 2.24627587 | 0.545829888 | -2.041011821 | Down | 1.80E-31 |
| 83872 | 4.57585447 | 1.112611601 | -2.040091117 | Down | 1.58E-121 |
| 9077 | 5.50692682 | 1.340675655 | -2.038287184 | Down | 1.80E-14 |
| 163115 | 1.61574419 | 0.394365732 | -2.0345927 | Down | 9.60E-09 |
| 5745 | 6.31280318 | 1.541335992 | -2.034099387 | Down | 7.06E-21 |
| 2624 | 4.8097709 | 1.174599056 | -2.033799793 | Down | 1.33E-25 |
| 6876 | 4069.41071 | 995.9496556 | -2.03067517 | Down | 0 |
| 9002 | 5.23353118 | 1.281079161 | -2.030425066 | Down | 4.55E-22 |
| 152006 | 10.8773011 | 2.66257777 | -2.030425066 | Down | 1.94E-83 |
| 79738 | 2.15801179 | 0.528911451 | -2.02860463 | Down | 1.40E-12 |
| 200845 | 9.24618748 | 2.266568351 | -2.028348947 | Down | 7.44E-26 |
| 2110 | 14.4363387 | 3.538858422 | -2.028348947 | Down | 4.13E-50 |
| 53826 | 75.8644245 | 18.6245328 | -2.026219278 | Down | 1.11E-182 |
| 93664 | 0.52325429 | 0.128505054 | -2.025687147 | Down | 1.34E-05 |
| 57338 | 0.74271731 | 0.182402569 | -2.025687147 | Down | 1.34E-05 |
| 115123 | 3.51319986 | 0.863727994 | -2.024136696 | Down | 2.54E-22 |
| 4212 | 36.2611639 | 8.921860925 | -2.023008669 | Down | 7.84E-193 |
| 84263 | 27.5913816 | 6.789262265 | -2.022890979 | Down | 1.23E-127 |
| 183 | 8.25155054 | 2.030936836 | -2.022519868 | Down | 5.20E-32 |
| 8165 | 30.7843871 | 7.586383933 | -2.020714554 | Down | 4.63E-175 |
| 55222 | 10.387298 | 2.561930138 | -2.01951738 | Down | 2.79E-48 |
| 5737 | 1.25113702 | 0.308677137 | -2.019069268 | Down | 3.68E-11 |
| 2159 | 13.0679461 | 3.226815442 | -2.017849436 | Down | 3.86E-30 |
| 51754 | 10.4094726 | 2.570903798 | -2.017549442 | Down | 1.57E-56 |
| 23363 | 11.1135703 | 2.745783325 | -2.017032682 | Down | 1.12E-93 |
| 283987 | 7.57188467 | 1.875635462 | -2.013272977 | Down | 1.78E-37 |
| 285704 | 34.9567941 | 8.661229391 | -2.012929156 | Down | 1.40E-227 |
| 8291 | 15.1454112 | 3.757331428 | -2.011100466 | Down | 2.57E-151 |
| 2157 | 2.56740107 | 0.637316796 | -2.010226101 | Down | 2.02E-34 |
| 64061 | 71.1360816 | 17.69102679 | -2.007563724 | Down | 3.62E-283 |
| 286367 | 2.05954672 | 0.51254427 | -2.006578324 | Down | 2.04E-11 |
| 7010 | 3.20025314 | 0.796423504 | -2.006578324 | Down | 4.42E-23 |
| 3768 | 2.01603239 | 0.50171518 | -2.006578324 | Down | 1.97E-16 |
| 170689 | 0.82126801 | 0.204382941 | -2.006578324 | Down | 0.00012726 |
| 122773 | 1.78656321 | 0.444608869 | -2.006578324 | Down | 4.77E-08 |
| 54753 | 7.92819897 | 1.977109185 | -2.003600624 | Down | 1.55E-41 |
| 9649 | 2.30511111 | 0.575189918 | -2.002725999 | Down | 3.82E-22 |
| 23179 | 7.85150212 | 1.967250767 | -1.996787821 | Down | 7.30E-58 |
| 9630 | 4.54814269 | 1.141620318 | -1.9941946 | Down | 2.06E-17 |
| 55068 | 3.73511718 | 0.93754439 | -1.9941946 | Down | 2.06E-17 |
| 26040 | 6.5407663 | 1.64198796 | -1.99401612 | Down | 4.46E-92 |
| 1047 | 1.43916888 | 0.361599196 | -1.992772525 | Down | 4.33E-07 |
| 50 | 77.5327043 | 19.49177406 | -1.991939582 | Down | 2.00E-297 |
| 11156 | 156.263812 | 39.2895133 | -1.991767512 | Down | 2.58E-291 |
| 441108 | 2.4298583 | 0.611899817 | -1.989504811 | Down | 2.62E-08 |
| 58476 | 52.4219118 | 13.20256684 | -1.989351523 | Down | 5.20E-302 |
| 196513 | 13.5522177 | 3.414450532 | -1.988803622 | Down | 3.79E-41 |
| 388403 | 4.09023493 | 1.030909197 | -1.988266446 | Down | 1.92E-31 |
| 375190 | 12.7867069 | 3.225135684 | -1.987212999 | Down | 9.37E-26 |
| 10000 | 3.70051282 | 0.93446239 | -1.985516709 | Down | 4.70E-20 |
| 6853 | 2.87106088 | 0.726610008 | -1.982330778 | Down | 2.47E-14 |
| 28971 | 100.417613 | 25.45297559 | -1.980106113 | Down | 1.03E-71 |
| 9311 | 3.02984178 | 0.767977712 | -1.980106113 | Down | 2.97E-10 |
| 84622 | 1.64949079 | 0.418467472 | -1.978833334 | Down | 1.16E-12 |
| 8850 | 5.71521842 | 1.450508785 | -1.978249601 | Down | 8.66E-40 |
| 473 | 33.4657295 | 8.51279956 | -1.974978895 | Down | 0 |
| 2992 | 95.06448 | 24.18833895 | -1.97459469 | Down | 8.67E-238 |
| 653140 | 1.45286664 | 0.369781622 | -1.974156846 | Down | 6.85E-05 |
| 5602 | 8.04763511 | 2.05318497 | -1.970701288 | Down | 1.59E-31 |
| 3400 | 27.0560161 | 6.903221311 | -1.970607779 | Down | 7.51E-89 |
| 21 | 4.26893205 | 1.08952417 | -1.970176996 | Down | 1.50E-40 |
| 256949 | 8.60333498 | 2.196841615 | -1.969465151 | Down | 1.28E-34 |
| 5468 | 3.41876884 | 0.873593293 | -1.968443195 | Down | 1.64E-10 |
| 2272 | 5.2307238 | 1.337395715 | -1.967584193 | Down | 2.64E-09 |
| 9796 | 4.35333575 | 1.113962058 | -1.966421198 | Down | 1.52E-21 |
| 4091 | 2.64842553 | 0.678479342 | -1.964758148 | Down | 1.90E-12 |
| 10156 | 39.1348859 | 10.03738625 | -1.9630716 | Down | 4.12E-301 |
| 166336 | 7.94072703 | 2.040970975 | -1.960015437 | Down | 7.60E-92 |
| 10771 | 29.0024374 | 7.4659364 | -1.957779026 | Down | 1.76E-175 |
| 3741 | 0.79056721 | 0.203526887 | -1.957668724 | Down | 0.0002114 |
| 7220 | 4.16623934 | 1.075045228 | -1.954348365 | Down | 1.08E-24 |
| 63934 | 1.10566917 | 0.285405079 | -1.953836862 | Down | 2.16E-06 |
| 90362 | 2.84302004 | 0.73422045 | -1.953139065 | Down | 1.77E-18 |
| 7170 | 179.627148 | 46.40759361 | -1.95257261 | Down | 0 |
| 26974 | 1.89004774 | 0.488453163 | -1.95213054 | Down | 2.36E-08 |
| 8863 | 6.63550927 | 1.715566291 | -1.951522321 | Down | 1.48E-57 |
| 54776 | 102.035529 | 26.39824295 | -1.950557777 | Down | 0 |
| 84302 | 4.44911824 | 1.151415123 | -1.950111372 | Down | 3.65E-14 |
| 687 | 20.1105167 | 5.206290461 | -1.949622439 | Down | 1.75E-143 |
| 8405 | 75.2464094 | 19.48399446 | -1.949333263 | Down | 0 |
| 7802 | 9.87707326 | 2.55892419 | -1.948546203 | Down | 4.03E-37 |
| 5521 | 2.37173612 | 0.614829747 | -1.947684635 | Down | 4.33E-09 |
| 84553 | 3.21693038 | 0.836420435 | -1.943384498 | Down | 4.57E-44 |
| 10965 | 16.0006434 | 4.166210929 | -1.941322133 | Down | 8.60E-40 |
| 2719 | 4.36346205 | 1.136409871 | -1.940989983 | Down | 3.77E-15 |
| 404093 | 14.6731721 | 3.822219627 | -1.940698205 | Down | 4.85E-45 |
| 1809 | 182.249683 | 47.51435936 | -1.939480823 | Down | 0 |
| 57484 | 7.79182654 | 2.032921828 | -1.938406822 | Down | 1.53E-50 |
| 83478 | 4.08676334 | 1.067267123 | -1.937037391 | Down | 3.69E-27 |
| 284615 | 1.08115432 | 0.282511885 | -1.936188996 | Down | 1.17E-06 |
| 26153 | 2.02278421 | 0.52856523 | -1.936188996 | Down | 5.67E-20 |
| 151556 | 20.0246968 | 5.237641066 | -1.934791296 | Down | 4.18E-202 |
| 147949 | 2.47274604 | 0.647268544 | -1.933677777 | Down | 4.37E-10 |
| 84253 | 3.05218894 | 0.799895804 | -1.931960283 | Down | 7.34E-17 |
| 9020 | 14.9840873 | 3.932398481 | -1.929949786 | Down | 3.80E-91 |
| 146894 | 3.12069913 | 0.820751929 | -1.926851132 | Down | 5.18E-13 |
| 54532 | 7.98197793 | 2.099883643 | -1.926436901 | Down | 6.78E-72 |
| 7728 | 2.41426759 | 0.635483803 | -1.925658329 | Down | 9.67E-14 |
| 394263 | 0.98269121 | 0.259175291 | -1.922809966 | Down | 3.54E-06 |
| 5500 | 115.227164 | 30.45042969 | -1.919946369 | Down | 0 |
| 54510 | 5.27648708 | 1.394573571 | -1.919753702 | Down | 4.59E-43 |
| 26507 | 1.66454585 | 0.440337371 | -1.918447414 | Down | 9.97E-15 |
| 4846 | 6.53706238 | 1.731028555 | -1.917012939 | Down | 2.96E-39 |
| 10656 | 12.0987138 | 3.204690038 | -1.916596957 | Down | 5.03E-33 |
| 4659 | 183.312573 | 48.55809353 | -1.916522052 | Down | 0 |
| 57030 | 2.12649064 | 0.563504379 | -1.915975775 | Down | 7.11E-10 |
| 7008 | 7.03352822 | 1.870637819 | -1.910718309 | Down | 3.49E-42 |
| 8425 | 88.2298095 | 23.47891254 | -1.909900581 | Down | 0 |
| 3485 | 133.249902 | 35.50041585 | -1.908226643 | Down | 6.52E-252 |
| 10638 | 12.6690473 | 3.378056556 | -1.907042651 | Down | 2.52E-20 |
| 92241 | 6.66092308 | 1.779541422 | -1.904216607 | Down | 8.18E-28 |
| 6451 | 162.618849 | 43.49200467 | -1.902672372 | Down | 0 |
| 3488 | 105.993687 | 28.35379665 | -1.902364509 | Down | 0 |
| 84814 | 14.6885059 | 3.931432701 | -1.901560586 | Down | 5.04E-42 |
| 91653 | 24.3614381 | 6.522460152 | -1.901111173 | Down | 3.81E-138 |
| 7404 | 1.12567361 | 0.301744525 | -1.899389078 | Down | 3.95E-11 |
| 11223 | 1.54605806 | 0.414607887 | -1.898775035 | Down | 2.13E-10 |
| 6910 | 10.3497157 | 2.781405568 | -1.895705107 | Down | 2.23E-53 |
| 2690 | 3.17067847 | 0.852589768 | -1.894867937 | Down | 1.19E-19 |
| 1910 | 3.51878823 | 0.94791726 | -1.892245649 | Down | 2.34E-21 |
| 6640 | 44.391642 | 11.9611186 | -1.891935758 | Down | 9.53E-137 |
| 200035 | 3.22737968 | 0.870105476 | -1.891101107 | Down | 5.78E-06 |
| 55861 | 5.65050879 | 1.523384026 | -1.891101107 | Down | 1.17E-10 |
| 90634 | 5.22905415 | 1.411354093 | -1.889470021 | Down | 2.44E-22 |
| 6324 | 32.9835632 | 8.905314892 | -1.889008728 | Down | 3.29E-66 |
| 55137 | 0.65444286 | 0.176795751 | -1.888183623 | Down | 3.25E-05 |
| 80108 | 2.0134555 | 0.54552399 | -1.883959037 | Down | 1.02E-07 |
| 7094 | 107.949001 | 29.2623895 | -1.8832304 | Down | 0 |
| 57326 | 131.448682 | 35.68074775 | -1.881281926 | Down | 0 |
| 7772 | 0.86535906 | 0.234933333 | -1.881047442 | Down | 5.63E-07 |
| 9863 | 2.76991737 | 0.752187895 | -1.880677947 | Down | 2.91E-26 |
| 8292 | 4.71007951 | 1.279146029 | -1.880570439 | Down | 1.10E-20 |
| 55790 | 9.33874946 | 2.536273214 | -1.880519208 | Down | 2.54E-53 |
| 134466 | 3.87285561 | 1.05416625 | -1.877295307 | Down | 1.24E-13 |
| 100113407 | 1.05544038 | 0.287647237 | -1.875472572 | Down | 6.65E-13 |
| 8994 | 5.65510465 | 1.542665951 | -1.874128028 | Down | 2.31E-47 |
| 10873 | 14.633674 | 4.005531995 | -1.869226257 | Down | 5.35E-45 |
| 4781 | 32.7573302 | 8.969189695 | -1.868768219 | Down | 0 |
| 115677 | 8.12150201 | 2.223857569 | -1.868682177 | Down | 2.64E-18 |
| 56971 | 5.80210509 | 1.588752338 | -1.868682177 | Down | 2.64E-18 |
| 147906 | 27.9449628 | 7.664306211 | -1.866361145 | Down | 8.14E-103 |
| 123722 | 1.59776655 | 0.438556357 | -1.865222475 | Down | 1.69E-06 |
| 29122 | 2.95711359 | 0.811671119 | -1.865222475 | Down | 1.69E-06 |
| 646405 | 0.41011398 | 0.112568442 | -1.865222475 | Down | 9.91E-05 |
| 2289 | 19.6631516 | 5.399481629 | -1.864601761 | Down | 1.16E-186 |
| 8786 | 13.4772985 | 3.711892273 | -1.860304591 | Down | 1.98E-42 |
| 79901 | 44.5096917 | 12.26232562 | -1.859886887 | Down | 8.66E-248 |
| 26267 | 4.79712072 | 1.322340878 | -1.859074617 | Down | 1.45E-29 |
| 10869 | 10.7544123 | 2.964545307 | -1.859045935 | Down | 7.61E-62 |
| 11343 | 48.3951668 | 13.34859474 | -1.858175102 | Down | 1.21E-284 |
| 23268 | 8.21297529 | 2.266363316 | -1.857525803 | Down | 2.05E-68 |
| 9254 | 1.03805387 | 0.286531149 | -1.857117425 | Down | 1.65E-08 |
| 4952 | 10.5015704 | 2.899117893 | -1.856919171 | Down | 2.30E-70 |
| 729234 | 8.98363104 | 2.480931839 | -1.856416578 | Down | 3.09E-11 |
| 388536 | 2.52275994 | 0.696688836 | -1.856416578 | Down | 3.09E-11 |
| 84085 | 10.2967106 | 2.844735065 | -1.855817327 | Down | 3.15E-59 |
| 767 | 0.9257111 | 0.255972139 | -1.854575231 | Down | 0.00057242 |
| 51276 | 0.92652455 | 0.25619707 | -1.854575231 | Down | 0.00057242 |
| 284276 | 0.94057533 | 0.260082307 | -1.854575231 | Down | 0.00057242 |
| 5729 | 0.71678106 | 0.198200045 | -1.854575231 | Down | 0.00057242 |
| 162963 | 0.93184705 | 0.257668816 | -1.854575231 | Down | 0.00057242 |
| 399668 | 0.61035308 | 0.16877121 | -1.854575231 | Down | 0.00057242 |
| 360200 | 0.66313518 | 0.183366205 | -1.854575231 | Down | 0.00057242 |
| 1154 | 32.7982638 | 9.098951392 | -1.849847249 | Down | 1.70E-95 |
| 54682 | 7.54844344 | 2.094302301 | -1.849709476 | Down | 1.57E-23 |
| 23522 | 17.0455473 | 4.731330629 | -1.849077038 | Down | 3.02E-185 |
| 3306 | 37.4576516 | 10.39753927 | -1.849018317 | Down | 2.16E-132 |
| 376940 | 6.00372612 | 1.667601139 | -1.848083903 | Down | 5.49E-89 |
| 10003 | 1.14856747 | 0.319284396 | -1.846921658 | Down | 5.06E-06 |
| 6604 | 47.3362852 | 13.16330364 | -1.846424879 | Down | 1.34E-103 |
| 57232 | 1.76941476 | 0.492486935 | -1.845114901 | Down | 4.94E-07 |
| 23194 | 13.0798723 | 3.642805601 | -1.844226542 | Down | 1.10E-76 |
| 51309 | 14.8296054 | 4.134766076 | -1.842602589 | Down | 2.08E-41 |
| 57658 | 81.2318216 | 22.66830835 | -1.841368265 | Down | 0 |
| 64798 | 6.14490147 | 1.717620722 | -1.838978376 | Down | 7.00E-22 |
| 3908 | 7.90534864 | 2.211008206 | -1.83812471 | Down | 1.65E-97 |
| 36 | 6.66520994 | 1.86483523 | -1.837602152 | Down | 3.14E-51 |
| 83888 | 2.00263047 | 0.560677434 | -1.836653323 | Down | 0.00030326 |
| 57690 | 9.53601348 | 2.678007968 | -1.832226022 | Down | 1.15E-117 |
| 2260 | 53.6566502 | 15.06921557 | -1.83215267 | Down | 0 |
| 817 | 17.2836903 | 4.855065414 | -1.831848647 | Down | 6.46E-128 |
| 64397 | 34.8101593 | 9.783446013 | -1.831093797 | Down | 0 |
| 54826 | 2.99294224 | 0.842043033 | -1.829598565 | Down | 8.72E-15 |
| 25789 | 5.11357279 | 1.440653568 | -1.827608183 | Down | 8.11E-12 |
| 9310 | 1.29762921 | 0.365582778 | -1.827608183 | Down | 1.47E-06 |
| 11145 | 34.1734068 | 9.630697589 | -1.827161874 | Down | 1.96E-60 |
| 58499 | 3.02241481 | 0.852337848 | -1.826204373 | Down | 8.96E-33 |
| 83878 | 2.78706331 | 0.786344285 | -1.825512765 | Down | 7.88E-10 |
| 6419 | 11.9992017 | 3.386244118 | -1.825180538 | Down | 5.19E-34 |
| 51149 | 4.9423786 | 1.395858059 | -1.824053286 | Down | 7.83E-09 |
| 80318 | 12.8084907 | 3.621041763 | -1.822623763 | Down | 2.47E-31 |
| 128853 | 1.86361924 | 0.52702868 | -1.822153753 | Down | 0.00016115 |
| 140735 | 27.2486691 | 7.710235676 | -1.821338901 | Down | 6.07E-53 |
| 2296 | 24.5698197 | 6.952790334 | -1.821223282 | Down | 2.59E-106 |
| 64922 | 1.21492915 | 0.344193647 | -1.819579809 | Down | 7.96E-07 |
| 10579 | 16.7840391 | 4.759944915 | -1.818073163 | Down | 7.57E-202 |
| 10912 | 15.9438021 | 4.525505537 | -1.816844842 | Down | 1.63E-22 |
| 9423 | 17.2366142 | 4.896746151 | -1.815581099 | Down | 8.22E-128 |
| 5136 | 6.47080267 | 1.841827694 | -1.812806581 | Down | 8.27E-21 |
| 7052 | 125.290814 | 35.70976171 | -1.810890227 | Down | 0 |
| 4744 | 1.02960097 | 0.294140704 | -1.807506977 | Down | 4.40E-06 |
| 22927 | 27.9809496 | 7.999248364 | -1.806508572 | Down | 1.56E-93 |
| 23024 | 24.4011931 | 6.989266646 | -1.8037387 | Down | 2.40E-125 |
| 7771 | 1.35921506 | 0.390297545 | -1.800127447 | Down | 1.26E-07 |
| 286204 | 0.89995891 | 0.258422501 | -1.800127447 | Down | 1.26E-07 |
| 10140 | 30.9075924 | 8.896572971 | -1.796639666 | Down | 3.76E-70 |
| 8324 | 10.7084307 | 3.085109019 | -1.795353694 | Down | 1.19E-51 |
| 219348 | 43.1711186 | 12.4548702 | -1.793356489 | Down | 7.88E-37 |
| 10144 | 3.22649818 | 0.933186238 | -1.789732275 | Down | 1.89E-24 |
| 23321 | 3.44976096 | 0.998025645 | -1.789347608 | Down | 6.33E-30 |
| 894 | 19.8819654 | 5.756380037 | -1.788226633 | Down | 1.21E-157 |
| 10715 | 1.07702095 | 0.311931811 | -1.787743722 | Down | 0.00049186 |
| 3305 | 2.27812043 | 0.660790512 | -1.785579138 | Down | 2.01E-08 |
| 9532 | 34.6699294 | 10.06210869 | -1.784752221 | Down | 4.87E-87 |
| 57513 | 14.9237616 | 4.334914139 | -1.783535894 | Down | 8.56E-91 |
| 160518 | 3.04393007 | 0.884808814 | -1.782497553 | Down | 5.69E-36 |
| 347746 | 8.64909018 | 2.514780256 | -1.782116039 | Down | 2.90E-20 |
| 23189 | 11.1894399 | 3.253683241 | -1.781992106 | Down | 5.39E-72 |
| 2206 | 9.75757752 | 2.839540472 | -1.780865545 | Down | 3.38E-45 |
| 91754 | 13.2190956 | 3.853519212 | -1.778374987 | Down | 3.18E-89 |
| 125875 | 2.56599879 | 0.748337438 | -1.777759634 | Down | 0.00026044 |
| 2775 | 2.41437722 | 0.704119141 | -1.777759634 | Down | 1.53E-19 |
| 92285 | 1.2626892 | 0.368245536 | -1.777759634 | Down | 7.06E-06 |
| 11142 | 162.527507 | 47.40521838 | -1.777566126 | Down | 2.90E-279 |
| 6196 | 10.1370903 | 2.957122861 | -1.777377513 | Down | 4.09E-72 |
| 54861 | 9.52507116 | 2.781347119 | -1.775946063 | Down | 5.84E-61 |
| 64506 | 3.21339166 | 0.938571207 | -1.775558725 | Down | 8.96E-14 |
| 9592 | 110.129994 | 32.16907332 | -1.775461155 | Down | 8.37E-270 |
| 4784 | 35.1573702 | 10.27439387 | -1.774773875 | Down | 7.10E-235 |
| 9465 | 3.73910212 | 1.093711608 | -1.773459496 | Down | 4.90E-14 |
| 51076 | 22.0377186 | 6.44697079 | -1.773281529 | Down | 1.38E-37 |
| 3339 | 19.7279004 | 5.779827778 | -1.771139011 | Down | 0 |
| 6285 | 34.4064265 | 10.08231404 | -1.770851264 | Down | 4.17E-48 |
| 115330 | 8.62373484 | 2.530547555 | -1.768863233 | Down | 1.32E-19 |
| 55244 | 2.9535593 | 0.866921193 | -1.76848182 | Down | 4.76E-13 |
| 57677 | 2.24138517 | 0.658510432 | -1.767112389 | Down | 1.36E-16 |
| 439921 | 257.326818 | 75.65338846 | -1.766125214 | Down | 0 |
| 8406 | 15.0975845 | 4.447274033 | -1.763324543 | Down | 1.02E-34 |
| 5463 | 7.77087427 | 2.29012156 | -1.762652741 | Down | 5.26E-49 |
| 8287 | 1.92144814 | 0.566261871 | -1.762652741 | Down | 1.97E-24 |
| 388125 | 1.88318423 | 0.554985278 | -1.762652741 | Down | 7.37E-05 |
| 25849 | 5.09937518 | 1.504337946 | -1.761191784 | Down | 5.51E-32 |
| 84775 | 1.81258304 | 0.535941665 | -1.757899211 | Down | 8.32E-11 |
| 23266 | 7.47353894 | 2.209890553 | -1.757816642 | Down | 7.44E-52 |
| 7100 | 1.86663137 | 0.552343261 | -1.756800015 | Down | 5.10E-09 |
| 124961 | 2.61123768 | 0.773386891 | -1.755471566 | Down | 6.44E-17 |
| 8801 | 35.1328819 | 10.41258092 | -1.754494218 | Down | 1.48E-98 |
| 3680 | 2.10928242 | 0.625490727 | -1.753691874 | Down | 2.47E-11 |
| 84251 | 10.0498431 | 2.981361048 | -1.753129967 | Down | 5.67E-57 |
| 23498 | 8.02923804 | 2.38418909 | -1.751764425 | Down | 1.23E-13 |
| 283349 | 2.57031704 | 0.763225827 | -1.751764425 | Down | 2.10E-05 |
| 9666 | 6.4134051 | 1.907375859 | -1.749501374 | Down | 3.23E-43 |
| 284 | 7.16570542 | 2.133882997 | -1.747627662 | Down | 5.88E-38 |
| 54858 | 16.2621849 | 4.846171555 | -1.746603718 | Down | 8.33E-136 |
| 5999 | 8.14690791 | 2.429602215 | -1.745532471 | Down | 1.05E-34 |
| 79812 | 18.3055863 | 5.461806272 | -1.744833931 | Down | 1.21E-90 |
| 57633 | 1.02148297 | 0.305050762 | -1.743543918 | Down | 6.05E-06 |
| 29799 | 5.46936892 | 1.633346027 | -1.743543918 | Down | 6.43E-29 |
| 144363 | 23.39637 | 6.999823122 | -1.74089434 | Down | 1.70E-32 |
| 401884 | 2.51750476 | 0.755171832 | -1.737117649 | Down | 4.39E-09 |
| 25840 | 49.4419334 | 14.83562415 | -1.736669538 | Down | 3.90E-195 |
| 23548 | 3.65972898 | 1.09874855 | -1.735875553 | Down | 6.05E-25 |
| 11154 | 5.04029049 | 1.513388585 | -1.735724414 | Down | 6.32E-12 |
| 8322 | 8.31486311 | 2.497721903 | -1.735079656 | Down | 1.43E-72 |
| 56062 | 0.41352352 | 0.124488585 | -1.731955944 | Down | 0.00041829 |
| 7732 | 1.51418401 | 0.455835312 | -1.731955944 | Down | 5.09E-07 |
| 6543 | 0.94762436 | 0.28527619 | -1.731955944 | Down | 5.09E-07 |
| 11163 | 3.01830073 | 0.908639931 | -1.731955944 | Down | 2.36E-18 |
| 23504 | 0.38297694 | 0.115292734 | -1.731955944 | Down | 0.00041829 |
| 92162 | 16.8894613 | 5.087153379 | -1.731192815 | Down | 1.29E-18 |
| 7067 | 20.6055169 | 6.210863508 | -1.730164889 | Down | 1.51E-59 |
| 55410 | 6.80062724 | 2.050434555 | -1.729738119 | Down | 3.06E-13 |
| 8742 | 51.95569 | 15.67146018 | -1.729142149 | Down | 2.05E-84 |
| 63891 | 12.2063975 | 3.682945778 | -1.728705409 | Down | 2.31E-61 |
| 23294 | 16.1099654 | 4.866090407 | -1.72711837 | Down | 5.80E-119 |
| 124751 | 2.82346424 | 0.853577153 | -1.725872892 | Down | 8.04E-08 |
| 51409 | 4.31756331 | 1.305792011 | -1.725292214 | Down | 1.14E-30 |
| 1801 | 9.32249572 | 2.822544715 | -1.723719794 | Down | 4.50E-25 |
| 644873 | 7.16755889 | 2.17207919 | -1.722405151 | Down | 7.43E-26 |
| 80820 | 7.79859426 | 2.36376917 | -1.722124935 | Down | 1.82E-43 |
| 4239 | 393.356168 | 119.443158 | -1.719511992 | Down | 0 |
| 56122 | 3.61897769 | 1.099604451 | -1.718597561 | Down | 4.81E-13 |
| 6038 | 38.2881477 | 11.63930039 | -1.717893521 | Down | 9.43E-80 |
| 57060 | 34.4985653 | 10.48870214 | -1.717700195 | Down | 1.08E-103 |
| 4130 | 20.1508478 | 6.128981938 | -1.717121181 | Down | 8.60E-237 |
| 84171 | 3.01133851 | 0.916831025 | -1.715677125 | Down | 4.27E-14 |
| 51306 | 10.6030521 | 3.228387531 | -1.715593936 | Down | 1.29E-68 |
| 57198 | 14.175013 | 4.318278822 | -1.714821757 | Down | 6.24E-96 |
| 151230 | 1.73090313 | 0.52745774 | -1.714397573 | Down | 9.62E-06 |
| 65018 | 58.2245987 | 17.75619062 | -1.713306688 | Down | 4.42E-177 |
| 384 | 8.32305359 | 2.540038679 | -1.71226246 | Down | 2.59E-20 |
| 3418 | 165.81572 | 50.69741884 | -1.709596586 | Down | 0 |
| 4286 | 18.7433105 | 5.733800067 | -1.70881228 | Down | 1.35E-102 |
| 2275 | 122.470725 | 37.47062571 | -1.708604961 | Down | 5.45E-237 |
| 157695 | 2.52745776 | 0.773348185 | -1.708496971 | Down | 8.06E-07 |
| 387890 | 3.0287717 | 0.926739561 | -1.708496971 | Down | 8.06E-07 |
| 7003 | 9.03508894 | 2.766825129 | -1.707307338 | Down | 8.25E-98 |
| 8153 | 4.98487136 | 1.527278849 | -1.706592782 | Down | 5.20E-25 |
| 169270 | 1.82012988 | 0.557752161 | -1.7063453 | Down | 2.35E-07 |
| 57381 | 17.3424645 | 5.318341857 | -1.705260509 | Down | 6.33E-39 |
| 25945 | 3.59745255 | 1.104364643 | -1.703759052 | Down | 3.72E-08 |
| 4826 | 4.55898364 | 1.399540446 | -1.703759052 | Down | 3.72E-08 |
| 414235 | 2.34004935 | 0.719374642 | -1.701723743 | Down | 5.92E-09 |
| 3547 | 1.46324003 | 0.449827168 | -1.701723743 | Down | 5.92E-09 |
| 57596 | 2.50110181 | 0.769197208 | -1.701138352 | Down | 3.21E-09 |
| 339318 | 4.65810082 | 1.432567894 | -1.701138352 | Down | 4.78E-17 |
| 4824 | 4.40517647 | 1.35554026 | -1.700331852 | Down | 7.83E-18 |
| 1850 | 6.67006231 | 2.05318497 | -1.699836633 | Down | 3.68E-35 |
| 947 | 53.0130695 | 16.33563923 | -1.698325166 | Down | 2.27E-168 |
| 1464 | 35.4806209 | 10.93760611 | -1.697734243 | Down | 0 |
| 400916 | 448.918102 | 138.6155751 | -1.695362903 | Down | 0 |
| 92249 | 28.0638891 | 8.687974657 | -1.691623153 | Down | 7.15E-92 |
| 22979 | 1.1624279 | 0.361606122 | -1.684650229 | Down | 3.85E-11 |
| 1735 | 1.22615862 | 0.381431366 | -1.684650229 | Down | 0.00035288 |
| 23175 | 22.1796185 | 6.899598523 | -1.684650229 | Down | 4.26E-133 |
| 54997 | 5.97841205 | 1.859754394 | -1.684650229 | Down | 3.16E-08 |
| 10170 | 1.71803878 | 0.534444621 | -1.684650229 | Down | 6.85E-07 |
| 6272 | 22.1019863 | 6.875448845 | -1.684650229 | Down | 3.44E-173 |
| 7041 | 270.975239 | 84.41586831 | -1.682574906 | Down | 0 |
| 50808 | 42.9534673 | 13.43546115 | -1.676728754 | Down | 2.16E-126 |
| 203260 | 30.713545 | 9.607250589 | -1.676679518 | Down | 1.05E-32 |
| 51201 | 3.42708939 | 1.072150566 | -1.676476298 | Down | 9.96E-17 |
| 79762 | 9.17153543 | 2.869415806 | -1.676406235 | Down | 1.15E-31 |
| 4013 | 13.4294043 | 4.205080757 | -1.6751899 | Down | 7.14E-54 |
| 23731 | 29.6705843 | 9.296714741 | -1.674240446 | Down | 1.51E-261 |
| 23078 | 4.45053855 | 1.394645964 | -1.674080988 | Down | 1.13E-36 |
| 150 | 2.46024161 | 0.771601013 | -1.672873061 | Down | 5.31E-12 |
| 27143 | 5.23077085 | 1.643132161 | -1.670575044 | Down | 7.90E-28 |
| 5010 | 2.77320784 | 0.871487751 | -1.670003453 | Down | 6.87E-10 |
| 26468 | 4.48624986 | 1.410037 | -1.669776953 | Down | 4.17E-18 |
| 8916 | 4.59614741 | 1.444655179 | -1.669699888 | Down | 2.89E-26 |
| 57545 | 6.99047975 | 2.197769943 | -1.669351093 | Down | 9.58E-42 |
| 5255 | 2.37058659 | 0.74532514 | -1.669302265 | Down | 1.39E-17 |
| 84976 | 4.10026238 | 1.290870247 | -1.667372238 | Down | 7.06E-23 |
| 65988 | 8.00460942 | 2.520801961 | -1.666948228 | Down | 2.35E-22 |
| 4499 | 13.6679529 | 4.307022109 | -1.666034551 | Down | 4.96E-08 |
| 84056 | 4.70860693 | 1.484146149 | -1.665667126 | Down | 2.96E-40 |
| 27086 | 64.3072259 | 20.27792398 | -1.665070896 | Down | 0 |
| 93 | 0.85155376 | 0.269172495 | -1.661566616 | Down | 4.51E-12 |
| 388335 | 2.98062918 | 0.942924534 | -1.660402683 | Down | 1.52E-11 |
| 4867 | 2.84009412 | 0.901161549 | -1.656081077 | Down | 5.83E-10 |
| 6843 | 13.5895517 | 4.320325163 | -1.653286058 | Down | 1.11E-47 |
| 3039 | 326.376614 | 103.8908031 | -1.651469743 | Down | 1.19E-203 |
| 98 | 18.3432429 | 5.845360485 | -1.649884811 | Down | 9.17E-26 |
| 168667 | 0.94237967 | 0.300303982 | -1.649884811 | Down | 8.38E-05 |
| 56271 | 48.5386775 | 15.48014577 | -1.648715743 | Down | 3.39E-68 |
| 100132707 | 1.73764541 | 0.554768656 | -1.647175524 | Down | 0.00015719 |
| 80863 | 10.0923703 | 3.229218134 | -1.644008245 | Down | 4.09E-22 |
| 10231 | 17.5601359 | 5.623672397 | -1.642719549 | Down | 6.21E-64 |
| 81847 | 19.762653 | 6.329215854 | -1.642677959 | Down | 6.36E-47 |
| 53346 | 2.74259613 | 0.878629732 | -1.642214963 | Down | 4.89E-07 |
| 79018 | 7.36364194 | 2.359561698 | -1.641900582 | Down | 5.84E-35 |
| 80022 | 6.74458169 | 2.162689625 | -1.640902334 | Down | 2.52E-69 |
| 64129 | 80.65359 | 25.89401555 | -1.63912004 | Down | 2.80E-193 |
| 79777 | 11.9772707 | 3.845672145 | -1.638991512 | Down | 1.54E-27 |
| 26018 | 15.679562 | 5.035735796 | -1.638610763 | Down | 3.87E-81 |
| 4675 | 1.69217412 | 0.544550366 | -1.635740629 | Down | 3.12E-06 |
| 582 | 17.3587058 | 5.601783149 | -1.631701349 | Down | 1.40E-63 |
| 130026 | 8.81047206 | 2.84405662 | -1.631269133 | Down | 3.56E-76 |
| 22875 | 3.32102886 | 1.074631749 | -1.627787892 | Down | 5.21E-18 |
| 9846 | 3.83222678 | 1.240289252 | -1.627506322 | Down | 4.04E-26 |
| 79661 | 8.09952767 | 2.622427788 | -1.626934731 | Down | 9.51E-18 |
| 100130015 | 2.77119973 | 0.897246292 | -1.626934731 | Down | 2.01E-05 |
| 90627 | 8.31776087 | 2.694090557 | -1.626396864 | Down | 1.35E-53 |
| 57149 | 27.835705 | 9.016160088 | -1.626351589 | Down | 1.45E-49 |
| 84795 | 5.33549658 | 1.730385299 | -1.624529237 | Down | 3.77E-13 |
| 6623 | 30.7304373 | 9.971369866 | -1.623804674 | Down | 2.40E-28 |
| 118490 | 3.70689131 | 1.203269772 | -1.623249685 | Down | 5.68E-11 |
| 26152 | 22.9641642 | 7.455238577 | -1.62305785 | Down | 9.59E-79 |
| 51015 | 18.0360616 | 5.861102251 | -1.621640432 | Down | 2.18E-38 |
| 84446 | 5.0953003 | 1.656009299 | -1.621456403 | Down | 4.40E-18 |
| 3290 | 4.91245961 | 1.596841032 | -1.621224849 | Down | 8.78E-09 |
| 9672 | 28.886214 | 9.393892571 | -1.62058613 | Down | 2.08E-156 |
| 196883 | 9.39508655 | 3.055453575 | -1.620519892 | Down | 4.32E-34 |
| 116159 | 5.96106146 | 1.94115669 | -1.61865267 | Down | 7.73E-21 |
| 23552 | 4.16106812 | 1.35605705 | -1.617536033 | Down | 7.77E-12 |
| 219654 | 35.8425755 | 11.68220176 | -1.617362099 | Down | 7.02E-186 |
| 80110 | 1.72076291 | 0.561277089 | -1.616263254 | Down | 6.46E-10 |
| 55527 | 22.0857215 | 7.205375976 | -1.615968345 | Down | 3.28E-90 |
| 57556 | 3.22201639 | 1.051883175 | -1.614989351 | Down | 1.05E-24 |
| 83875 | 2.60431456 | 0.851905899 | -1.612137729 | Down | 2.18E-09 |
| 158135 | 2.32962825 | 0.762052355 | -1.612137729 | Down | 2.18E-09 |
| 57653 | 1.66944438 | 0.54645703 | -1.611188067 | Down | 1.95E-12 |
| 9867 | 24.4301174 | 8.008855114 | -1.608992874 | Down | 7.27E-125 |
| 488 | 84.5845206 | 27.72972747 | -1.608960229 | Down | 0 |
| 2192 | 274.670928 | 90.07748151 | -1.608465822 | Down | 0 |
| 6443 | 29.0397798 | 9.538985093 | -1.606122832 | Down | 5.07E-130 |
| 55063 | 4.07724252 | 1.341115472 | -1.604160311 | Down | 2.20E-11 |
| 5523 | 10.1465026 | 3.340117534 | -1.60301176 | Down | 3.16E-72 |
| 57631 | 2.6959269 | 0.887976444 | -1.602188069 | Down | 2.79E-15 |
| 100128573 | 7.61612237 | 2.508575901 | -1.602188069 | Down | 1.68E-05 |
| 202052 | 4.12692399 | 1.35931404 | -1.602188069 | Down | 1.16E-16 |
| 10090 | 8.35987622 | 2.75550422 | -1.601165244 | Down | 1.73E-39 |
| 22835 | 2.25279641 | 0.742949279 | -1.600381312 | Down | 3.01E-12 |
| 8536 | 24.3840178 | 8.060446998 | -1.597004107 | Down | 8.74E-39 |
| 6347 | 262.328506 | 86.74362929 | -1.596544875 | Down | 3.73E-202 |
| 5627 | 21.4733017 | 7.10755384 | -1.595119008 | Down | 2.42E-80 |
| 747 | 0.84185683 | 0.278779114 | -1.59445242 | Down | 2.19E-06 |
| 404217 | 24.5500614 | 8.137147561 | -1.593131573 | Down | 2.23E-33 |
| 23148 | 4.47941315 | 1.485324043 | -1.592532027 | Down | 1.95E-23 |
| 4162 | 162.534018 | 53.98791957 | -1.590033171 | Down | 0 |
| 1740 | 0.84482471 | 0.280931252 | -1.588434914 | Down | 2.10E-08 |
| 169841 | 0.92247151 | 0.307458098 | -1.585114556 | Down | 0.00010932 |
| 5364 | 19.4989362 | 6.501097035 | -1.584640323 | Down | 5.25E-146 |
| 10411 | 3.12812361 | 1.043435405 | -1.58395623 | Down | 2.34E-21 |
| 1153 | 152.040343 | 50.75135979 | -1.582935806 | Down | 0 |
| 2039 | 25.2643425 | 8.437457386 | -1.582222416 | Down | 1.48E-72 |
| 26273 | 12.3251593 | 4.11660176 | -1.582080496 | Down | 1.03E-31 |
| 144983 | 4.42526293 | 1.479334964 | -1.580814418 | Down | 3.89E-12 |
| 114757 | 16.973602 | 5.678619221 | -1.57968067 | Down | 4.03E-35 |
| 57670 | 0.33041484 | 0.110542246 | -1.57968067 | Down | 1.40E-05 |
| 8483 | 26.7975084 | 8.98865815 | -1.575921202 | Down | 5.53E-122 |
| 128977 | 9.79483278 | 3.285937422 | -1.575715858 | Down | 1.34E-13 |
| 7060 | 19.6887491 | 6.610373568 | -1.574567746 | Down | 5.53E-68 |
| 7042 | 3.7284031 | 1.252611415 | -1.573618917 | Down | 3.02E-21 |
| 143279 | 3.42612185 | 1.151661757 | -1.572859404 | Down | 3.71E-18 |
| 91663 | 182.037244 | 61.23050303 | -1.571911206 | Down | 0 |
| 51185 | 22.9233403 | 7.716001583 | -1.570891939 | Down | 3.25E-52 |
| 256302 | 6.6418206 | 2.238726655 | -1.568900368 | Down | 1.85E-33 |
| 729085 | 1.88939517 | 0.637319147 | -1.567836564 | Down | 5.95E-08 |
| 7544 | 1.23735729 | 0.417377743 | -1.567836564 | Down | 5.95E-08 |
| 1879 | 9.99606504 | 3.372144144 | -1.567694083 | Down | 2.52E-54 |
| 3611 | 325.325276 | 109.8088662 | -1.56688837 | Down | 0 |
| 55450 | 48.4030077 | 16.33858402 | -1.566813739 | Down | 4.57E-115 |
| 5095 | 12.4688448 | 4.210141023 | -1.566387348 | Down | 1.11E-32 |
| 2824 | 19.792789 | 6.685938931 | -1.565772833 | Down | 4.11E-65 |
| 284018 | 9.23847751 | 3.122442744 | -1.564979999 | Down | 5.38E-16 |
| 388566 | 1.47652281 | 0.500142173 | -1.561793481 | Down | 4.99E-12 |
| 275 | 19.5349317 | 6.619886207 | -1.56117789 | Down | 1.84E-42 |
| 84527 | 4.59578017 | 1.557437317 | -1.561135693 | Down | 1.79E-15 |
| 26227 | 29.1236563 | 9.880060646 | -1.559599685 | Down | 8.41E-60 |
| 26167 | 1.47870649 | 0.501811129 | -1.559119347 | Down | 1.17E-05 |
| 84449 | 4.04300629 | 1.373786227 | -1.557270923 | Down | 1.14E-16 |
| 23708 | 4.99511224 | 1.697748093 | -1.556894682 | Down | 4.32E-14 |
| 6322 | 4.86126839 | 1.654268358 | -1.555139496 | Down | 1.50E-15 |
| 84333 | 4.89853472 | 1.667006431 | -1.555090595 | Down | 3.89E-36 |
| 4958 | 7.15889184 | 2.437248485 | -1.554482937 | Down | 1.40E-19 |
| 100131434 | 4.92508865 | 1.677015589 | -1.554253592 | Down | 5.73E-13 |
| 5023 | 3.95421623 | 1.348836425 | -1.551676364 | Down | 1.05E-12 |
| 3201 | 19.5256471 | 6.664533854 | -1.550794483 | Down | 7.62E-35 |
| 202134 | 1.52849934 | 0.521871598 | -1.550349138 | Down | 0.00016953 |
| 79739 | 4.32475954 | 1.478540822 | -1.548445846 | Down | 4.38E-17 |
| 492311 | 8.24280189 | 2.818033917 | -1.548445846 | Down | 7.24E-25 |
| 55197 | 10.6894566 | 3.655506497 | -1.548045301 | Down | 2.13E-46 |
| 114789 | 13.9688288 | 4.777108929 | -1.548001383 | Down | 1.08E-48 |
| 8284 | 8.70761962 | 2.978755897 | -1.547568485 | Down | 2.77E-49 |
| 375484 | 3.41888762 | 1.169896008 | -1.547146706 | Down | 2.25E-08 |
| 222663 | 3.03859532 | 1.040240245 | -1.54648779 | Down | 7.97E-17 |
| 6508 | 11.4363846 | 3.918528449 | -1.545247173 | Down | 4.20E-49 |
| 8604 | 21.1727417 | 7.261439411 | -1.543880636 | Down | 3.31E-84 |
| 65012 | 5.64128284 | 1.936420196 | -1.542631224 | Down | 7.58E-15 |
| 84870 | 56.5067301 | 19.40504338 | -1.541991048 | Down | 2.02E-119 |
| 25994 | 63.4816136 | 21.83168771 | -1.539915136 | Down | 1.11E-182 |
| 64375 | 8.08106031 | 2.781272761 | -1.538799363 | Down | 1.73E-44 |
| 3991 | 2.11365386 | 0.727731615 | -1.538260742 | Down | 2.52E-09 |
| 7857 | 3.2490172 | 1.11863753 | -1.538260742 | Down | 2.52E-09 |
| 8555 | 4.16435342 | 1.434547738 | -1.537496535 | Down | 3.04E-24 |
| 285830 | 2.66491771 | 0.918274853 | -1.537093041 | Down | 2.34E-06 |
| 1408 | 18.3366448 | 6.328293115 | -1.534841349 | Down | 2.94E-76 |
| 56948 | 14.2120063 | 4.914467519 | -1.532003219 | Down | 1.63E-18 |
| 9811 | 17.6638212 | 6.109550493 | -1.531659327 | Down | 1.32E-100 |
| 9338 | 48.8950697 | 16.92260249 | -1.530737546 | Down | 1.58E-58 |
| 22933 | 77.0283295 | 26.66002634 | -1.530710932 | Down | 3.44E-147 |
| 4302 | 27.7732804 | 9.621224772 | -1.529405123 | Down | 9.26E-202 |
| 84448 | 10.902382 | 3.779094113 | -1.528531027 | Down | 3.88E-41 |
| 4601 | 43.2602809 | 14.99771742 | -1.528300085 | Down | 8.37E-146 |
| 23236 | 11.0577605 | 3.835089529 | -1.527727074 | Down | 3.56E-74 |
| 2947 | 32.58848 | 11.3032136 | -1.527629061 | Down | 9.46E-131 |
| 79366 | 25.2749674 | 8.776738011 | -1.525952483 | Down | 2.99E-53 |
| 8777 | 9.30342742 | 3.233807735 | -1.524528403 | Down | 5.62E-68 |
| 11096 | 3.64564969 | 1.267356154 | -1.524353929 | Down | 1.95E-35 |
| 266812 | 6.4484701 | 2.242709737 | -1.523714011 | Down | 1.27E-13 |
| 57599 | 10.3896968 | 3.613794679 | -1.523567105 | Down | 2.09E-38 |
| 27246 | 47.5711521 | 16.55556799 | -1.522770456 | Down | 8.86E-78 |
| 133308 | 6.0454519 | 2.1048355 | -1.522142699 | Down | 4.65E-21 |
| 10894 | 16.4952666 | 5.743579636 | -1.522030028 | Down | 4.09E-41 |
| 79632 | 2.58860916 | 0.901410666 | -1.521920729 | Down | 1.48E-11 |
| 57209 | 5.00637111 | 1.74644763 | -1.519341856 | Down | 5.68E-25 |
| 55260 | 11.7032335 | 4.083546631 | -1.519012586 | Down | 9.30E-27 |
| 23413 | 45.7254584 | 15.96206304 | -1.518350506 | Down | 1.62E-218 |
| 65982 | 29.2351218 | 10.20632078 | -1.518239712 | Down | 3.70E-83 |
| 4882 | 12.4781978 | 4.356283855 | -1.518239712 | Down | 1.86E-42 |
| 6253 | 14.272033 | 4.982707435 | -1.518189083 | Down | 1.06E-32 |
| 57521 | 6.84650751 | 2.392038354 | -1.517127718 | Down | 2.38E-46 |
| 154810 | 17.4274916 | 6.09087965 | -1.516642431 | Down | 1.52E-150 |
| 125170 | 7.80724444 | 2.728731164 | -1.516583174 | Down | 7.03E-21 |
| 1193 | 8.22897709 | 2.876446405 | -1.516425517 | Down | 1.14E-22 |
| 6722 | 50.3922674 | 17.62277475 | -1.515761275 | Down | 6.39E-208 |
| 352954 | 11.762554 | 4.114214251 | -1.51551251 | Down | 5.07E-47 |
| 80317 | 1.90577122 | 0.666949628 | -1.514725228 | Down | 1.48E-05 |
| 126069 | 1.327993 | 0.464748564 | -1.514725228 | Down | 6.19E-05 |
| 386724 | 1.05153266 | 0.367997643 | -1.514725228 | Down | 0.00026145 |
| 3032 | 119.438419 | 41.80736901 | -1.51443781 | Down | 5.58E-249 |
| 130271 | 2.75206876 | 0.964432923 | -1.512763711 | Down | 9.15E-20 |
| 25956 | 5.13119779 | 1.801656456 | -1.509971698 | Down | 5.11E-24 |
| 84935 | 15.4571231 | 5.42848361 | -1.509650671 | Down | 1.40E-36 |
| 51421 | 8.74423038 | 3.071126395 | -1.509563523 | Down | 7.52E-43 |
| 54839 | 4.75382742 | 1.670188419 | -1.509078665 | Down | 4.02E-14 |
| 146433 | 4.13369863 | 1.452315147 | -1.509078665 | Down | 9.61E-08 |
| 23529 | 23.193331 | 8.151434142 | -1.508584224 | Down | 5.33E-42 |
| 6415 | 682.510117 | 240.0336552 | -1.507613732 | Down | 0 |
| 163131 | 1.9110541 | 0.67245308 | -1.50686311 | Down | 6.61E-06 |
| 85004 | 6.16506673 | 2.169874779 | -1.506504721 | Down | 1.84E-14 |
| 64221 | 4.94017247 | 1.738848598 | -1.506429086 | Down | 6.61E-23 |
| 6578 | 11.3186796 | 3.987010029 | -1.50532653 | Down | 1.21E-46 |
| 115286 | 12.720581 | 4.482865135 | -1.504671563 | Down | 1.20E-22 |
| 1003 | 17.2173826 | 6.074005538 | -1.503145709 | Down | 2.32E-68 |
| 80833 | 16.6656128 | 5.880011969 | -1.502983374 | Down | 1.79E-58 |
| 219749 | 5.58174279 | 1.97047503 | -1.502172177 | Down | 3.38E-21 |
| 57544 | 1.86840243 | 0.65976267 | -1.501786172 | Down | 1.20E-09 |
| 8653 | 14.652637 | 5.174927265 | -1.501549839 | Down | 1.76E-65 |
| 83700 | 36.0358736 | 12.72806617 | -1.501420578 | Down | 3.17E-125 |
| 81609 | 12.1149957 | 4.279666287 | -1.501223681 | Down | 4.99E-82 |
| 847 | 33.535767 | 11.8514598 | -1.500635823 | Down | 1.15E-73 |
| 83692 | 23.5066042 | 8.309846134 | -1.500172472 | Down | 6.34E-81 |
| 8013 | 4.19827421 | 1.484947287 | -1.499384682 | Down | 1.02E-26 |
| 9923 | 4.85535441 | 1.718446407 | -1.498471747 | Down | 1.78E-42 |
| 150709 | 1.10244588 | 0.390250052 | -1.498237105 | Down | 1.22E-05 |
| 54477 | 18.0032485 | 6.376879794 | -1.497334655 | Down | 9.29E-76 |
| 8910 | 43.4734407 | 15.39888728 | -1.497308175 | Down | 1.20E-73 |
| 158586 | 1.89650028 | 0.671779414 | -1.497280093 | Down | 1.54E-11 |
| 2037 | 31.9143939 | 11.30796002 | -1.496868563 | Down | 4.94E-134 |
| 23396 | 44.7074257 | 15.84272021 | -1.496694407 | Down | 1.49E-211 |
| 152330 | 1.2234988 | 0.433599443 | -1.496577881 | Down | 3.24E-07 |
| 8828 | 23.8926861 | 8.468232278 | -1.496436309 | Down | 8.65E-150 |
| 8082 | 47.9410897 | 16.99487705 | -1.496162777 | Down | 1.53E-203 |
| 55349 | 1.0725431 | 0.380355194 | -1.495616405 | Down | 5.09E-05 |
| 132884 | 2.14587038 | 0.761319495 | -1.49498901 | Down | 2.48E-10 |
| 203 | 57.0776196 | 20.2645551 | -1.493966668 | Down | 8.79E-122 |
| 80210 | 19.5080405 | 6.933896313 | -1.492330706 | Down | 7.29E-42 |
| 2030 | 120.375418 | 42.79424103 | -1.492052237 | Down | 1.68E-278 |
| 26049 | 0.55205465 | 0.196265409 | -1.492005151 | Down | 0.00021469 |
| 145581 | 0.87849724 | 0.312321656 | -1.492005151 | Down | 0.00021469 |
| 3087 | 4.66217838 | 1.65748872 | -1.492005151 | Down | 4.02E-09 |
| 55786 | 6.50281461 | 2.311868187 | -1.492005151 | Down | 2.65E-15 |
| 83988 | 8.71451824 | 3.099991275 | -1.491156757 | Down | 1.82E-32 |
| 3700 | 3.33014103 | 1.184990037 | -1.490708347 | Down | 1.28E-11 |
| 11346 | 58.6639797 | 20.87544548 | -1.490667961 | Down | 1.53E-293 |
| 7730 | 2.34094951 | 0.83414136 | -1.488730019 | Down | 2.26E-05 |
| 27347 | 15.9831198 | 5.699250156 | -1.487705014 | Down | 4.77E-50 |
| 84892 | 8.61651879 | 3.072901147 | -1.487503759 | Down | 7.42E-22 |
| 115207 | 21.5762 | 7.701336507 | -1.48626006 | Down | 1.13E-125 |
| 201973 | 2.92672008 | 1.048063701 | -1.481558364 | Down | 1.21E-07 |
| 23140 | 6.56623778 | 2.353910351 | -1.480007618 | Down | 2.86E-70 |
| 126068 | 3.56448413 | 1.278163036 | -1.479621427 | Down | 3.82E-16 |
| 133522 | 1.24504432 | 0.446891885 | -1.478199352 | Down | 4.19E-05 |
| 155054 | 0.96453481 | 0.346206775 | -1.478199352 | Down | 0.00040085 |
| 127435 | 51.8954075 | 18.64578444 | -1.476757378 | Down | 3.40E-144 |
| 84224 | 2.53014226 | 0.909649263 | -1.475836214 | Down | 3.10E-10 |
| 284390 | 2.08432374 | 0.750277466 | -1.474083243 | Down | 2.01E-06 |
| 56953 | 3.4428093 | 1.239280727 | -1.474083243 | Down | 2.01E-06 |
| 862 | 4.14983431 | 1.494384267 | -1.473502565 | Down | 1.09E-14 |
| 5578 | 5.69961875 | 2.052833799 | -1.473248592 | Down | 3.24E-47 |
| 9625 | 2.97057481 | 1.070470567 | -1.472496992 | Down | 5.74E-16 |
| 3491 | 75.3753045 | 27.16997327 | -1.472078779 | Down | 1.87E-158 |
| 54922 | 9.98161485 | 3.598048102 | -1.472058763 | Down | 1.15E-31 |
| 8803 | 15.4111854 | 5.557546656 | -1.471457774 | Down | 5.29E-32 |
| 5236 | 57.2268054 | 20.66095212 | -1.469784335 | Down | 6.50E-130 |
| 2869 | 18.2280083 | 6.582151427 | -1.469525808 | Down | 5.35E-44 |
| 4774 | 46.3623422 | 16.75941197 | -1.467981922 | Down | 0 |
| 57396 | 12.8665213 | 4.651811182 | -1.467757605 | Down | 6.76E-31 |
| 400713 | 2.76469339 | 0.999792062 | -1.467419513 | Down | 4.05E-07 |
| 27303 | 7.16843392 | 2.592958607 | -1.467058794 | Down | 7.07E-20 |
| 91734 | 7.41371146 | 2.681680149 | -1.467058794 | Down | 1.16E-10 |
| 5787 | 1.81313273 | 0.656243515 | -1.466181376 | Down | 3.24E-20 |
| 221895 | 11.6458064 | 4.215158579 | -1.466151724 | Down | 2.25E-35 |
| 114928 | 5.07273249 | 1.836485437 | -1.465815627 | Down | 5.26E-18 |
| 7089 | 19.207922 | 6.954556636 | -1.465671004 | Down | 1.25E-48 |
| 117583 | 6.24563154 | 2.263355732 | -1.464384109 | Down | 3.63E-22 |
| 5193 | 2.99946554 | 1.087070342 | -1.464260161 | Down | 9.15E-09 |
| 4674 | 2.22180417 | 0.806347634 | -1.462257808 | Down | 1.66E-06 |
| 1806 | 2.56419561 | 0.934252257 | -1.456622273 | Down | 9.02E-12 |
| 284058 | 12.2433636 | 4.464632722 | -1.45538656 | Down | 5.08E-56 |
| 83992 | 1.1383691 | 0.41517746 | -1.455168383 | Down | 1.50E-07 |
| 22982 | 14.9596039 | 5.456909966 | -1.454915833 | Down | 4.18E-107 |
| 8462 | 14.3026576 | 5.226960597 | -1.452239052 | Down | 4.60E-53 |
| 10186 | 43.973422 | 16.07725655 | -1.451610564 | Down | 3.80E-86 |
| 107 | 0.63741911 | 0.233279137 | -1.450184976 | Down | 1.38E-08 |
| 81610 | 42.7972602 | 15.66388254 | -1.450076588 | Down | 4.79E-94 |
| 79891 | 6.70642602 | 2.455555238 | -1.449494856 | Down | 4.03E-16 |
| 57763 | 4.08254858 | 1.495505027 | -1.448837292 | Down | 6.22E-09 |
| 11159 | 4.03110133 | 1.477914711 | -1.447611032 | Down | 2.81E-09 |
| 5162 | 63.4712326 | 23.27033256 | -1.447611032 | Down | 5.69E-87 |
| 254102 | 48.2336254 | 17.68731651 | -1.447324071 | Down | 1.15E-222 |
| 7162 | 150.324355 | 55.15937009 | -1.446400886 | Down | 0 |
| 25859 | 2.78602419 | 1.022477886 | -1.446138143 | Down | 1.23E-07 |
| 56129 | 1.28243819 | 0.470878491 | -1.445462565 | Down | 1.25E-05 |
| 9240 | 29.5802841 | 10.86564441 | -1.444862168 | Down | 8.25E-71 |
| 654 | 1.66015163 | 0.610334856 | -1.44364213 | Down | 5.58E-06 |
| 4929 | 7.52049844 | 2.76607219 | -1.442989474 | Down | 4.13E-25 |
| 100271722 | 5.05141118 | 1.858464381 | -1.442575442 | Down | 1.13E-08 |
| 64651 | 31.9421445 | 11.7590917 | -1.441684548 | Down | 2.54E-91 |
| 10268 | 17.6267163 | 6.493368949 | -1.440724646 | Down | 1.44E-22 |
| 1066 | 76.7051264 | 28.25680509 | -1.440724646 | Down | 2.28E-137 |
| 26092 | 36.5586583 | 13.47864428 | -1.439537731 | Down | 6.39E-124 |
| 57605 | 9.44976387 | 3.484257333 | -1.4394271 | Down | 1.15E-57 |
| 221061 | 5.10138424 | 1.882172855 | -1.438489642 | Down | 2.41E-19 |
| 51705 | 14.3430032 | 5.292739398 | -1.438260606 | Down | 1.58E-52 |
| 7048 | 21.755133 | 8.028071108 | -1.438230537 | Down | 1.77E-91 |
| 339524 | 3.13158193 | 1.155914878 | -1.437856464 | Down | 9.80E-11 |
| 1407 | 7.57532777 | 2.79650347 | -1.437684192 | Down | 2.50E-23 |
| 607 | 6.25965613 | 2.311141686 | -1.437477698 | Down | 3.57E-36 |
| 55825 | 4.22515341 | 1.56160828 | -1.435971116 | Down | 2.06E-08 |
| 5244 | 1.03797321 | 0.38381379 | -1.43529076 | Down | 5.20E-05 |
| 6594 | 9.88229029 | 3.656890265 | -1.434228099 | Down | 5.17E-37 |
| 55709 | 1.84144314 | 0.681473792 | -1.434106768 | Down | 2.31E-05 |
| 3040 | 472.746575 | 175.0518356 | -1.433284817 | Down | 6.62E-251 |
| 5291 | 1.5314233 | 0.567134037 | -1.433111462 | Down | 1.03E-05 |
| 57664 | 28.5759713 | 10.5915841 | -1.431884159 | Down | 1.64E-78 |
| 780776 | 1.82449892 | 0.676708613 | -1.430893637 | Down | 9.17E-07 |
| 25891 | 6.67447612 | 2.486307473 | -1.424649884 | Down | 1.01E-17 |
| 79998 | 1.65783796 | 0.618860942 | -1.421615823 | Down | 0.00021785 |
| 93653 | 2.89575274 | 1.080967087 | -1.421615823 | Down | 3.75E-06 |
| 414328 | 6.04883738 | 2.257994624 | -1.421615823 | Down | 5.85E-10 |
| 9481 | 12.4056156 | 4.630941701 | -1.421615823 | Down | 2.26E-33 |
| 5994 | 1.65881603 | 0.619226051 | -1.421615823 | Down | 1.89E-05 |
| 80854 | 8.59885883 | 3.211981251 | -1.420681738 | Down | 1.12E-53 |
| 9149 | 14.8502752 | 5.549268965 | -1.420120028 | Down | 3.94E-34 |
| 50804 | 4.6546425 | 1.740248116 | -1.419377351 | Down | 2.57E-23 |
| 23126 | 18.6964636 | 6.994698836 | -1.418431567 | Down | 5.70E-108 |
| 770 | 28.6328908 | 10.71494262 | -1.418049208 | Down | 2.16E-42 |
| 27345 | 7.27872067 | 2.726040499 | -1.416877904 | Down | 9.29E-12 |
| 116224 | 6.29790481 | 2.358966495 | -1.416717024 | Down | 2.28E-21 |
| 9693 | 3.13784592 | 1.175613975 | -1.416360098 | Down | 5.18E-20 |
| 6622 | 3.20769808 | 1.201950069 | -1.416161393 | Down | 2.18E-10 |
| 25939 | 14.89183 | 5.5860162 | -1.414629393 | Down | 3.75E-36 |
| 4355 | 5.23988353 | 1.965929263 | -1.414323331 | Down | 1.88E-21 |
| 112 | 7.66038179 | 2.875115628 | -1.413796319 | Down | 9.54E-45 |
| 8660 | 12.0870396 | 4.541182763 | -1.412320931 | Down | 6.18E-74 |
| 3791 | 3.97350465 | 1.492917119 | -1.412277959 | Down | 3.26E-22 |
| 9730 | 2.06485813 | 0.775804312 | -1.412277959 | Down | 7.64E-12 |
| 91947 | 17.4564182 | 6.559388326 | -1.412124374 | Down | 2.47E-62 |
| 29 | 20.9965187 | 7.897725158 | -1.410641077 | Down | 6.58E-102 |
| 3590 | 16.8625437 | 6.345449778 | -1.410027849 | Down | 1.92E-26 |
| 727 | 0.8147598 | 0.306812871 | -1.409015787 | Down | 3.47E-05 |
| 27013 | 27.3396717 | 10.29710871 | -1.408756612 | Down | 5.80E-50 |
| 6339 | 3.17182642 | 1.194886335 | -1.408440435 | Down | 9.39E-09 |
| 2948 | 64.8936593 | 24.44947071 | -1.408274286 | Down | 2.57E-80 |
| 8611 | 40.9497878 | 15.42984299 | -1.408132594 | Down | 2.72E-58 |
| 2805 | 35.1204489 | 13.23367528 | -1.408097499 | Down | 3.45E-65 |
| 1427 | 4.82343775 | 1.817873391 | -1.407810024 | Down | 7.83E-05 |
| 58155 | 11.5582853 | 4.35652811 | -1.407676632 | Down | 6.78E-36 |
| 51660 | 75.4937335 | 28.45525768 | -1.407661652 | Down | 3.60E-63 |
| 3249 | 1.55345817 | 0.586065706 | -1.406349067 | Down | 0.00017735 |
| 10014 | 19.8518687 | 7.498152885 | -1.404667669 | Down | 3.11E-91 |
| 11278 | 2.89958275 | 1.09575973 | -1.403913822 | Down | 1.81E-28 |
| 284119 | 426.992912 | 161.378958 | -1.40375964 | Down | 0 |
| 26137 | 16.6815937 | 6.305963167 | -1.403468477 | Down | 1.77E-43 |
| 9927 | 53.3709369 | 20.18175028 | -1.403003039 | Down | 2.54E-212 |
| 51133 | 11.8614208 | 4.487131031 | -1.402411613 | Down | 4.05E-41 |
| 3777 | 3.53405377 | 1.337563814 | -1.401716266 | Down | 1.83E-13 |
| 56999 | 6.34476715 | 2.402355582 | -1.401117513 | Down | 7.26E-41 |
| 7592 | 1.23178939 | 0.466483625 | -1.400857263 | Down | 5.61E-06 |
| 8503 | 7.08772386 | 2.684335839 | -1.400757219 | Down | 1.22E-35 |
| 84909 | 34.2875901 | 13.00095653 | -1.399068738 | Down | 4.41E-83 |
| 8678 | 16.8377955 | 6.394286744 | -1.396847918 | Down | 5.17E-32 |
| 51566 | 19.7844553 | 7.514233655 | -1.396669466 | Down | 4.05E-58 |
| 152007 | 53.381854 | 20.28324313 | -1.396061065 | Down | 5.07E-86 |
| 54897 | 4.93248414 | 1.87434332 | -1.39592918 | Down | 1.52E-34 |
| 8727 | 17.6968646 | 6.737301848 | -1.393250932 | Down | 6.47E-38 |
| 1960 | 9.2267017 | 3.514906758 | -1.392328596 | Down | 3.92E-35 |
| 5144 | 5.74353542 | 2.188325321 | -1.392111829 | Down | 3.35E-41 |
| 3570 | 14.3823971 | 5.480568215 | -1.391906768 | Down | 6.81E-52 |
| 79898 | 3.16908408 | 1.208440892 | -1.390919026 | Down | 1.52E-07 |
| 252983 | 1.35639626 | 0.518390152 | -1.387668492 | Down | 2.53E-08 |
| 144100 | 1.54481488 | 0.591845636 | -1.384141118 | Down | 1.24E-07 |
| 284370 | 4.3379568 | 1.662708186 | -1.383480695 | Down | 4.18E-16 |
| 400657 | 2.90185308 | 1.112634655 | -1.382994529 | Down | 7.13E-10 |
| 7812 | 237.716417 | 91.19652527 | -1.382190777 | Down | 0 |
| 10882 | 4.68615529 | 1.797905639 | -1.382087459 | Down | 2.76E-07 |
| 9677 | 5.63556246 | 2.162584837 | -1.381802876 | Down | 3.24E-28 |
| 133584 | 3.84667504 | 1.478174211 | -1.379795648 | Down | 9.59E-15 |
| 55228 | 1.79619048 | 0.690227902 | -1.379795648 | Down | 6.14E-07 |
| 79026 | 101.469023 | 39.03375804 | -1.378245087 | Down | 0 |
| 27445 | 0.30797551 | 0.118558073 | -1.377221704 | Down | 1.37E-06 |
| 80303 | 10.3665577 | 3.994368649 | -1.375897523 | Down | 1.43E-18 |
| 2059 | 5.93281116 | 2.286124113 | -1.375812134 | Down | 1.55E-21 |
| 48 | 18.2268723 | 7.025850825 | -1.375322171 | Down | 9.22E-55 |
| 10903 | 15.5221379 | 5.993245492 | -1.372917901 | Down | 5.24E-38 |
| 343990 | 1.65531461 | 0.640525784 | -1.369776892 | Down | 1.12E-06 |
| 79776 | 3.74607654 | 1.450062814 | -1.36926498 | Down | 6.58E-44 |
| 84872 | 4.97116898 | 1.924437398 | -1.369148404 | Down | 3.92E-10 |
| 3945 | 189.831847 | 73.50339165 | -1.36883932 | Down | 1.02E-202 |
| 51616 | 19.8088379 | 7.671001415 | -1.368657415 | Down | 4.35E-45 |
| 55778 | 6.23597044 | 2.415693676 | -1.368176564 | Down | 1.73E-18 |
| 81563 | 13.2595585 | 5.140630996 | -1.367015381 | Down | 1.13E-112 |
| 1998 | 9.51405052 | 3.691968562 | -1.365669418 | Down | 1.56E-26 |
| 375387 | 3.20144538 | 1.242410224 | -1.365581789 | Down | 6.79E-08 |
| 185 | 3.65600351 | 1.419113141 | -1.365277839 | Down | 1.13E-08 |
| 113026 | 25.0632764 | 9.735266465 | -1.364282648 | Down | 6.21E-72 |
| 92703 | 1.68870461 | 0.656649247 | -1.362722134 | Down | 0.00021565 |
| 55857 | 13.479454 | 5.24422336 | -1.361961019 | Down | 1.32E-25 |
| 9159 | 57.904416 | 22.55262217 | -1.360378194 | Down | 3.69E-164 |
| 283070 | 22.5694461 | 8.79370951 | -1.359827232 | Down | 1.32E-45 |
| 9941 | 7.37332127 | 2.872892638 | -1.359810539 | Down | 2.33E-20 |
| 91369 | 25.4470525 | 9.915435144 | -1.359750568 | Down | 3.71E-87 |
| 57194 | 9.43080546 | 3.676432851 | -1.359074357 | Down | 1.78E-41 |
| 4299 | 8.15338386 | 3.178846148 | -1.358895745 | Down | 2.78E-63 |
| 9459 | 6.23931492 | 2.434212466 | -1.357932533 | Down | 3.80E-28 |
| 57758 | 3.19767086 | 1.248283718 | -1.357075571 | Down | 3.63E-11 |
| 3910 | 60.5142326 | 23.62585604 | -1.356907893 | Down | 0 |
| 10098 | 2.35347673 | 0.919400978 | -1.356027482 | Down | 0.00049068 |
| 283507 | 0.80248776 | 0.31349706 | -1.356027482 | Down | 0.00049068 |
| 2053 | 13.7639807 | 5.376988509 | -1.356027482 | Down | 5.97E-26 |
| 644353 | 1.10292653 | 0.430865417 | -1.356027482 | Down | 0.00049068 |
| 55268 | 77.6372962 | 30.33384212 | -1.355821632 | Down | 6.77E-75 |
| 6263 | 1.23486321 | 0.482516283 | -1.355701707 | Down | 4.73E-17 |
| 389114 | 3.8828621 | 1.519583709 | -1.353444321 | Down | 4.32E-19 |
| 113612 | 2.35030937 | 0.920303869 | -1.35266847 | Down | 1.75E-10 |
| 2054 | 12.2748786 | 4.809979494 | -1.351606102 | Down | 1.39E-35 |
| 498 | 254.144617 | 99.60744505 | -1.351324192 | Down | 0 |
| 26269 | 6.09462257 | 2.388840258 | -1.351226496 | Down | 4.48E-14 |
| 5253 | 10.4983662 | 4.115198368 | -1.35113095 | Down | 1.51E-46 |
| 80298 | 18.7446212 | 7.350402194 | -1.350581572 | Down | 3.07E-28 |
| 122525 | 5.5633984 | 2.182127248 | -1.35023119 | Down | 1.68E-14 |
| 64283 | 1.02610026 | 0.402466475 | -1.35023119 | Down | 1.01E-05 |
| 23067 | 12.0194488 | 4.714507593 | -1.350191739 | Down | 3.28E-80 |
| 84276 | 6.38955169 | 2.507150721 | -1.349665982 | Down | 4.48E-18 |
| 7846 | 461.063267 | 180.9725326 | -1.349193984 | Down | 0 |
| 8491 | 6.66520994 | 2.622765338 | -1.345561583 | Down | 2.02E-23 |
| 80263 | 5.58342993 | 2.198243836 | -1.344800226 | Down | 1.75E-17 |
| 9604 | 12.8374078 | 5.054196582 | -1.344800226 | Down | 2.12E-33 |
| 55810 | 8.9471772 | 3.523410728 | -1.344459928 | Down | 1.42E-40 |
| 54492 | 12.742733 | 5.018522608 | -1.34434011 | Down | 2.38E-66 |
| 9185 | 0.69725685 | 0.274945554 | -1.342544252 | Down | 8.22E-06 |
| 6595 | 13.8267091 | 5.457740904 | -1.341082006 | Down | 1.65E-64 |
| 199777 | 2.42466971 | 0.957793252 | -1.340002058 | Down | 3.15E-13 |
| 375341 | 4.98203139 | 1.96939901 | -1.338978674 | Down | 1.06E-15 |
| 1845 | 63.7023051 | 25.19783957 | -1.338045534 | Down | 9.51E-208 |
| 728855 | 8.97165844 | 3.555515437 | -1.335315977 | Down | 1.72E-10 |
| 3384 | 18.311693 | 7.261507744 | -1.33442414 | Down | 8.30E-21 |
| 85453 | 6.87750777 | 2.727382276 | -1.334368938 | Down | 1.01E-25 |
| 56890 | 2.104482 | 0.834689882 | -1.334152982 | Down | 2.43E-06 |
| 84190 | 3.16364914 | 1.254781903 | -1.334152982 | Down | 2.43E-06 |
| 9260 | 375.385992 | 149.1880962 | -1.331242387 | Down | 0 |
| 3214 | 17.748842 | 7.0629361 | -1.329384951 | Down | 8.56E-30 |
| 4213 | 13.3999539 | 5.333895619 | -1.328966535 | Down | 1.74E-31 |
| 65123 | 28.0555928 | 11.17226721 | -1.328366413 | Down | 2.07E-99 |
| 196403 | 50.8494426 | 20.26065524 | -1.327551127 | Down | 7.96E-82 |
| 219333 | 3.89182403 | 1.552130887 | -1.326196258 | Down | 1.33E-08 |
| 84858 | 45.1319454 | 18.02139674 | -1.324438137 | Down | 1.19E-112 |
| 11188 | 18.2920167 | 7.304110496 | -1.32443365 | Down | 7.28E-76 |
| 5310 | 21.169127 | 8.465285409 | -1.322331163 | Down | 2.93E-232 |
| 79178 | 11.4313788 | 4.572069623 | -1.32208015 | Down | 4.44E-21 |
| 4134 | 63.7831632 | 25.51715935 | -1.321707914 | Down | 0 |
| 9209 | 34.5461632 | 13.83611959 | -1.3200861 | Down | 4.32E-103 |
| 285464 | 3.23449836 | 1.296006583 | -1.319468936 | Down | 1.20E-12 |
| 255031 | 6.1870643 | 2.479416986 | -1.319254106 | Down | 8.62E-12 |
| 160760 | 3.10493214 | 1.244620132 | -1.318856249 | Down | 1.67E-10 |
| 55218 | 7.83508833 | 3.141061147 | -1.31869751 | Down | 1.05E-18 |
| 5565 | 13.8116139 | 5.53750385 | -1.318574206 | Down | 2.72E-59 |
| 55187 | 4.96512044 | 1.990940571 | -1.318378557 | Down | 5.14E-64 |
| 7373 | 12.2613446 | 4.920141968 | -1.317345341 | Down | 1.67E-62 |
| 100130581 | 2.04105341 | 0.819779775 | -1.316005635 | Down | 3.58E-06 |
| 87 | 339.790553 | 136.5473616 | -1.315244305 | Down | 0 |
| 540 | 0.68201598 | 0.274345327 | -1.313812534 | Down | 7.51E-05 |
| 283232 | 15.4953995 | 6.242416042 | -1.311663534 | Down | 7.67E-21 |
| 23303 | 4.65978253 | 1.879158455 | -1.310175902 | Down | 8.40E-33 |
| 2736 | 2.29611686 | 0.926041323 | -1.310047594 | Down | 1.99E-13 |
| 27020 | 61.7370235 | 24.9002037 | -1.309978383 | Down | 7.94E-117 |
| 10052 | 5.43571127 | 2.193126018 | -1.309480113 | Down | 2.59E-33 |
| 29767 | 3.41943373 | 1.381237591 | -1.307795924 | Down | 1.61E-25 |
| 94134 | 4.75504732 | 1.921785069 | -1.307012702 | Down | 1.12E-16 |
| 7561 | 3.34763034 | 1.352967821 | -1.307012702 | Down | 4.77E-09 |
| 23291 | 7.01642924 | 2.835863569 | -1.306948881 | Down | 4.95E-26 |
| 3212 | 36.5648821 | 14.78690487 | -1.306138606 | Down | 4.27E-46 |
| 4190 | 136.864315 | 55.41332497 | -1.304441494 | Down | 9.90E-133 |
| 114907 | 77.2261895 | 31.27210772 | -1.304213729 | Down | 4.41E-90 |
| 147808 | 11.2693113 | 4.56463342 | -1.303828445 | Down | 1.21E-18 |
| 8527 | 5.85707366 | 2.37401551 | -1.302850678 | Down | 1.61E-29 |
| 54538 | 9.65084119 | 3.914218376 | -1.30193045 | Down | 1.32E-29 |
| 51619 | 14.5255171 | 5.891524685 | -1.301876579 | Down | 6.09E-17 |
| 10788 | 5.07687131 | 2.059413214 | -1.30170636 | Down | 9.44E-24 |
| 57623 | 2.62769724 | 1.066199546 | -1.30132159 | Down | 2.07E-12 |
| 58528 | 7.07427992 | 2.871706924 | -1.300674786 | Down | 6.49E-28 |
| 196527 | 20.5492721 | 8.346782184 | -1.299795261 | Down | 6.07E-96 |
| 51719 | 24.0437335 | 9.779917777 | -1.297766693 | Down | 6.38E-73 |
| 55793 | 5.36456664 | 2.182277439 | -1.297627106 | Down | 5.15E-13 |
| 57659 | 46.1622837 | 18.78014637 | -1.297506291 | Down | 6.40E-207 |
| 55628 | 1.29715837 | 0.527910571 | -1.296989169 | Down | 2.58E-09 |
| 342926 | 1.22978596 | 0.500805495 | -1.296084941 | Down | 0.00013659 |
| 57665 | 24.1629882 | 9.839888971 | -1.296084941 | Down | 1.21E-30 |
| 51315 | 17.8660227 | 7.276332932 | -1.295935043 | Down | 3.99E-25 |
| 5664 | 9.89027553 | 4.028191662 | -1.295878385 | Down | 1.17E-18 |
| 4637 | 3781.03538 | 1540.219579 | -1.295645307 | Down | 0 |
| 7259 | 28.2583339 | 11.52600542 | -1.293783805 | Down | 3.01E-76 |
| 65251 | 3.54899738 | 1.449021201 | -1.292332806 | Down | 6.33E-10 |
| 147685 | 5.46291327 | 2.230454493 | -1.292332806 | Down | 3.98E-05 |
| 7507 | 20.686383 | 8.451412061 | -1.2914171 | Down | 7.40E-28 |
| 7450 | 22.6889212 | 9.275012645 | -1.290566863 | Down | 1.76E-150 |
| 6584 | 5.00047059 | 2.044421376 | -1.29037129 | Down | 8.50E-14 |
| 152137 | 14.9344578 | 6.109303737 | -1.289564988 | Down | 7.24E-101 |
| 202181 | 3.79988091 | 1.556238031 | -1.287891464 | Down | 1.39E-09 |
| 2631 | 86.6092024 | 35.48400803 | -1.287351347 | Down | 3.60E-128 |
| 79993 | 1.6501964 | 0.676106775 | -1.287314732 | Down | 3.44E-06 |
| 9215 | 7.19488256 | 2.949528992 | -1.286486549 | Down | 4.78E-24 |
| 23710 | 78.6464198 | 32.24721097 | -1.286206704 | Down | 3.36E-110 |
| 25827 | 3.00903282 | 1.234344902 | -1.285554274 | Down | 1.02E-06 |
| 10171 | 5.07720836 | 2.083476312 | -1.285042771 | Down | 1.13E-09 |
| 80315 | 3.17678181 | 1.304085627 | -1.284527404 | Down | 7.07E-20 |
| 140733 | 0.80319404 | 0.329810255 | -1.2841123 | Down | 0.00030809 |
| 55900 | 14.1436254 | 5.807703351 | -1.2841123 | Down | 4.52E-29 |
| 132720 | 3.38081636 | 1.390381312 | -1.28189106 | Down | 2.73E-08 |
| 1384 | 42.3930919 | 17.43916365 | -1.281498339 | Down | 6.57E-91 |
| 55893 | 34.1231216 | 14.04028769 | -1.281177136 | Down | 5.11E-122 |
| 801 | 247.898098 | 102.0190028 | -1.280909301 | Down | 0 |
| 11080 | 7.99530295 | 3.29087828 | -1.280680034 | Down | 9.34E-15 |
| 4338 | 41.5209412 | 17.09364404 | -1.280379161 | Down | 5.20E-48 |
| 3705 | 53.4508072 | 22.01128794 | -1.279968171 | Down | 8.74E-134 |
| 23122 | 4.99168057 | 2.058134108 | -1.278188624 | Down | 3.04E-28 |
| 23258 | 17.6847923 | 7.297616645 | -1.277012007 | Down | 6.55E-66 |
| 127396 | 2.60963302 | 1.078534635 | -1.274774435 | Down | 2.10E-05 |
| 9778 | 8.01205118 | 3.311828907 | -1.274543493 | Down | 1.27E-47 |
| 10181 | 40.223211 | 16.62854565 | -1.27436626 | Down | 1.61E-92 |
| 1195 | 38.2543289 | 15.81574791 | -1.274261234 | Down | 1.49E-94 |
| 100129482 | 4.35104384 | 1.799082609 | -1.274100121 | Down | 3.40E-29 |
| 51665 | 6.44663541 | 2.666766664 | -1.273454796 | Down | 8.63E-34 |
| 138311 | 9.80885484 | 4.061486772 | -1.27207677 | Down | 2.76E-18 |
| 155066 | 12.5731706 | 5.212017953 | -1.270434544 | Down | 1.43E-26 |
| 9946 | 20.4619646 | 8.482295453 | -1.27041803 | Down | 4.42E-27 |
| 10150 | 37.7894326 | 15.66535612 | -1.270405289 | Down | 1.70E-130 |
| 140461 | 32.9686777 | 13.66825586 | -1.270266865 | Down | 3.41E-64 |
| 254827 | 0.85861966 | 0.356130618 | -1.26961273 | Down | 0.00020012 |
| 347733 | 3.13494036 | 1.300282652 | -1.26961273 | Down | 5.01E-06 |
| 7436 | 8.59214392 | 3.568227676 | -1.267810487 | Down | 2.81E-24 |
| 4719 | 20.0227882 | 8.319094503 | -1.267144474 | Down | 3.71E-51 |
| 5990 | 7.97740444 | 3.314705357 | -1.267038786 | Down | 2.17E-25 |
| 10087 | 7.6908446 | 3.197310523 | -1.266283176 | Down | 3.29E-32 |
| 100272228 | 1.81633926 | 0.75591018 | -1.264746975 | Down | 1.38E-05 |
| 80345 | 4.53301296 | 1.886514661 | -1.264746975 | Down | 1.38E-05 |
| 7182 | 3.67541592 | 1.5312619 | -1.263186461 | Down | 1.07E-23 |
| 54462 | 13.8785735 | 5.782807774 | -1.26301724 | Down | 2.90E-78 |
| 23567 | 6.4301838 | 2.680231536 | -1.262502341 | Down | 7.98E-16 |
| 5178 | 1.75661718 | 0.732292482 | -1.262307929 | Down | 1.21E-12 |
| 6314 | 3.89870298 | 1.629286181 | -1.258754215 | Down | 5.28E-22 |
| 5098 | 1.95008578 | 0.817407432 | -1.254410323 | Down | 4.64E-08 |
| 11252 | 14.6650664 | 6.151766633 | -1.253310918 | Down | 1.19E-23 |
| 10180 | 49.6310565 | 20.83378867 | -1.252317944 | Down | 5.11E-129 |
| 26051 | 1.9991064 | 0.8395362 | -1.251690822 | Down | 2.05E-10 |
| 7915 | 2.41710138 | 1.015075587 | -1.251690822 | Down | 2.05E-10 |
| 57007 | 20.6413983 | 8.677150768 | -1.250247405 | Down | 2.22E-32 |
| 9915 | 1.92934115 | 0.811349322 | -1.249713173 | Down | 1.67E-10 |
| 2550 | 32.9831516 | 13.88038118 | -1.248682072 | Down | 2.85E-107 |
| 9828 | 20.8253573 | 8.770453465 | -1.247617906 | Down | 2.67E-116 |
| 285195 | 4.47044545 | 1.883464901 | -1.247029445 | Down | 6.21E-13 |
| 79772 | 1.23845536 | 0.521960578 | -1.246529117 | Down | 8.45E-05 |
| 388507 | 1.928715 | 0.812878065 | -1.246529117 | Down | 1.41E-06 |
| 80311 | 1.15790542 | 0.488011923 | -1.246529117 | Down | 1.41E-06 |
| 259217 | 8.18971565 | 3.452056276 | -1.246357378 | Down | 1.04E-34 |
| 79582 | 8.19107181 | 3.453298457 | -1.246077216 | Down | 3.82E-14 |
| 55178 | 10.8415586 | 4.573210166 | -1.245293051 | Down | 2.36E-15 |
| 373 | 2.30302504 | 0.971840886 | -1.244738061 | Down | 8.25E-08 |
| 57337 | 2.65988948 | 1.122946088 | -1.244077638 | Down | 8.95E-11 |
| 79742 | 10.2996111 | 4.351993658 | -1.242841501 | Down | 2.49E-35 |
| 3767 | 2.42711063 | 1.025689589 | -1.242645682 | Down | 2.76E-07 |
| 127002 | 3.47851863 | 1.470011424 | -1.242645682 | Down | 2.76E-07 |
| 64777 | 16.9769453 | 7.175926896 | -1.2423398 | Down | 3.50E-23 |
| 9580 | 7.15253263 | 3.024326843 | -1.241840113 | Down | 4.54E-22 |
| 51780 | 9.79004995 | 4.14027326 | -1.241590232 | Down | 2.12E-48 |
| 1822 | 76.5947469 | 32.39841887 | -1.241322044 | Down | 2.75E-234 |
| 124637 | 6.06723256 | 2.568399565 | -1.240168952 | Down | 1.60E-17 |
| 5322 | 8.45355364 | 3.578981088 | -1.240009017 | Down | 8.91E-13 |
| 726 | 4.23426332 | 1.793146054 | -1.23961799 | Down | 1.36E-14 |
| 91526 | 1.63155163 | 0.692100468 | -1.237191252 | Down | 1.30E-05 |
| 25841 | 2.52247453 | 1.071624983 | -1.235039581 | Down | 8.69E-09 |
| 23417 | 7.57897553 | 3.221021615 | -1.234484507 | Down | 3.89E-13 |
| 10444 | 24.6179066 | 10.46606604 | -1.23398882 | Down | 9.17E-75 |
| 64081 | 2.71713681 | 1.155164563 | -1.23398882 | Down | 2.51E-06 |
| 90736 | 12.5947876 | 5.355325493 | -1.233780624 | Down | 1.04E-10 |
| 26999 | 3.40426537 | 1.448960237 | -1.232325497 | Down | 1.24E-17 |
| 151473 | 1.03676002 | 0.441334357 | -1.232138025 | Down | 0.00018889 |
| 100132215 | 2.20497649 | 0.938627899 | -1.232138025 | Down | 0.00018889 |
| 51166 | 3.45854109 | 1.472802719 | -1.231599404 | Down | 4.90E-07 |
| 57092 | 66.1726708 | 28.19163267 | -1.230968477 | Down | 6.63E-104 |
| 6935 | 27.3222901 | 11.64175991 | -1.230769244 | Down | 3.64E-115 |
| 57494 | 2.78650744 | 1.187712918 | -1.230271838 | Down | 8.43E-13 |
| 55731 | 14.6302751 | 6.236133426 | -1.230233197 | Down | 4.58E-44 |
| 23430 | 7.39919257 | 3.15422042 | -1.230084366 | Down | 8.49E-06 |
| 84645 | 4.0553267 | 1.728755422 | -1.230084366 | Down | 8.49E-06 |
| 342035 | 1.07558158 | 0.458866442 | -1.228970745 | Down | 3.57E-05 |
| 54536 | 3.53932702 | 1.510529147 | -1.228421043 | Down | 2.27E-10 |
| 23301 | 19.8509484 | 8.472072815 | -1.228421043 | Down | 3.31E-72 |
| 26058 | 8.88645239 | 3.793771626 | -1.227974753 | Down | 2.47E-50 |
| 55742 | 64.1970083 | 27.43275907 | -1.226606336 | Down | 2.13E-138 |
| 81539 | 32.4230688 | 13.86625875 | -1.225442059 | Down | 6.94E-190 |
| 55529 | 5.87554799 | 2.514426233 | -1.224494184 | Down | 2.99E-11 |
| 7716 | 20.802632 | 8.904520961 | -1.224156165 | Down | 1.17E-67 |
| 56478 | 10.2480533 | 4.388602695 | -1.223516315 | Down | 2.27E-26 |
| 79791 | 16.2947846 | 6.978215549 | -1.223480214 | Down | 5.45E-42 |
| 94015 | 6.07590527 | 2.6023896 | -1.223262414 | Down | 6.12E-16 |
| 23637 | 19.6076712 | 8.398384932 | -1.223234376 | Down | 1.06E-68 |
| 81704 | 3.33288017 | 1.428027067 | -1.222746125 | Down | 1.28E-18 |
| 22869 | 1.87906887 | 0.806096195 | -1.220994025 | Down | 4.14E-08 |
| 5799 | 1.40769115 | 0.604003037 | -1.22070313 | Down | 4.48E-06 |
| 634 | 1.94307268 | 0.833721092 | -1.22070313 | Down | 4.48E-06 |
| 10811 | 7.17415079 | 3.079777456 | -1.219981962 | Down | 1.63E-09 |
| 8493 | 5.25951669 | 2.25830873 | -1.219687505 | Down | 6.45E-13 |
| 7565 | 2.21650362 | 0.952607291 | -1.218332225 | Down | 1.88E-05 |
| 221091 | 26.692061 | 11.47368072 | -1.218082429 | Down | 1.78E-45 |
| 34 | 14.7842375 | 6.355058949 | -1.218082429 | Down | 5.95E-28 |
| 11067 | 86.6711246 | 37.25783203 | -1.218007696 | Down | 8.90E-123 |
| 7775 | 3.83371703 | 1.648575132 | -1.217524219 | Down | 7.04E-07 |
| 4698 | 79.8158063 | 34.32792807 | -1.217291695 | Down | 1.40E-85 |
| 114804 | 4.50315475 | 1.937323377 | -1.216871268 | Down | 1.44E-16 |
| 35 | 18.2931988 | 7.870542387 | -1.216772406 | Down | 1.83E-25 |
| 28968 | 1.38470323 | 0.596424683 | -1.215164946 | Down | 0.00042317 |
| 1435 | 4.78125498 | 2.059400465 | -1.215164946 | Down | 3.46E-11 |
| 23095 | 17.9072752 | 7.715209756 | -1.214768547 | Down | 1.23E-129 |
| 79970 | 5.99447719 | 2.583529009 | -1.21429085 | Down | 3.81E-16 |
| 132789 | 7.4926362 | 3.230180213 | -1.213858748 | Down | 2.81E-11 |
| 26224 | 11.5789068 | 4.992051902 | -1.213794214 | Down | 1.91E-29 |
| 5797 | 10.8227872 | 4.672645689 | -1.211760537 | Down | 1.54E-42 |
| 56204 | 8.44552531 | 3.646995974 | -1.211478554 | Down | 1.14E-25 |
| 84134 | 9.39097588 | 4.055385325 | -1.211436086 | Down | 1.39E-18 |
| 2944 | 53.1678092 | 22.96220337 | -1.211291937 | Down | 4.32E-44 |
| 645644 | 2.64495191 | 1.142382234 | -1.211196044 | Down | 2.47E-14 |
| 22898 | 8.3655721 | 3.615612336 | -1.210224205 | Down | 2.58E-32 |
| 115294 | 21.5873613 | 9.333015676 | -1.209771687 | Down | 2.09E-62 |
| 285203 | 12.5892765 | 5.442825649 | -1.209767652 | Down | 1.04E-36 |
| 51268 | 2.65522895 | 1.148319455 | -1.20931222 | Down | 9.86E-06 |
| 11043 | 4.22262393 | 1.826420295 | -1.20912097 | Down | 1.73E-22 |
| 788 | 10.9962423 | 4.760355518 | -1.20786938 | Down | 2.17E-15 |
| 1891 | 206.913112 | 89.62404371 | -1.207067344 | Down | 2.81E-176 |
| 51074 | 20.8604687 | 9.038583757 | -1.206602932 | Down | 3.26E-19 |
| 3726 | 384.749865 | 166.968258 | -1.204346959 | Down | 0 |
| 7035 | 9.69549371 | 4.207586978 | -1.20432127 | Down | 5.18E-27 |
| 3098 | 65.1150521 | 28.26872524 | -1.20378425 | Down | 2.39E-173 |
| 1471 | 1456.43691 | 632.3862695 | -1.203565259 | Down | 0 |
| 56975 | 9.49510442 | 4.130767934 | -1.200773854 | Down | 2.06E-08 |
| 1605 | 23.4788657 | 10.21647291 | -1.200465499 | Down | 1.11E-87 |
| 339483 | 6.99325801 | 3.043539581 | -1.200214605 | Down | 2.29E-12 |
| 26140 | 23.233339 | 10.11263003 | -1.200038254 | Down | 3.92E-54 |
| 3930 | 26.5285163 | 11.55214344 | -1.199383426 | Down | 2.38E-68 |
| 1027 | 21.8480096 | 9.515019035 | -1.199223402 | Down | 1.22E-36 |
| 5884 | 8.19534112 | 3.570685936 | -1.198602755 | Down | 8.08E-19 |
| 5295 | 16.5742016 | 7.224263849 | -1.198016883 | Down | 2.99E-72 |
| 7005 | 73.5851199 | 32.0941436 | -1.197103995 | Down | 7.41E-147 |
| 51439 | 13.2036838 | 5.761598845 | -1.19639937 | Down | 4.63E-43 |
| 27306 | 5.99673123 | 2.617651922 | -1.195903044 | Down | 6.84E-08 |
| 51435 | 21.466919 | 9.374477288 | -1.195304994 | Down | 4.38E-53 |
| 142679 | 1.36894337 | 0.598000172 | -1.194844962 | Down | 2.73E-06 |
| 51310 | 34.6530255 | 15.14355306 | -1.194277579 | Down | 3.59E-55 |
| 84437 | 3.91755091 | 1.712075943 | -1.194205327 | Down | 4.01E-12 |
| 5824 | 24.8634023 | 10.86665913 | -1.194115265 | Down | 9.93E-62 |
| 22808 | 10.854401 | 4.745742989 | -1.193574238 | Down | 1.68E-33 |
| 84981 | 39.1369956 | 17.11563228 | -1.193218419 | Down | 7.06E-37 |
| 643641 | 2.17423473 | 0.951127444 | -1.192797133 | Down | 2.00E-11 |
| 54820 | 21.7900659 | 9.546631288 | -1.190606917 | Down | 8.01E-58 |
| 2151 | 4.14562125 | 1.816857961 | -1.190142679 | Down | 8.09E-11 |
| 1808 | 31.1034096 | 13.64644416 | -1.18854766 | Down | 7.37E-94 |
| 55283 | 1.8994907 | 0.834198185 | -1.18715057 | Down | 7.39E-05 |
| 54332 | 1.70705066 | 0.749684407 | -1.18715057 | Down | 9.18E-06 |
| 114876 | 25.7274696 | 11.30387181 | -1.186492556 | Down | 2.23E-71 |
| 3177 | 13.8503993 | 6.086081095 | -1.186342111 | Down | 2.27E-24 |
| 169981 | 1.5238675 | 0.670197516 | -1.18507922 | Down | 7.41E-06 |
| 5869 | 83.461175 | 36.70956352 | -1.184949271 | Down | 5.05E-180 |
| 4037 | 16.4159286 | 7.225631723 | -1.183900732 | Down | 4.67E-41 |
| 255104 | 9.4017238 | 4.141924422 | -1.18262407 | Down | 8.85E-20 |
| 29997 | 195.461734 | 86.15350299 | -1.18190483 | Down | 7.56E-194 |
| 23002 | 18.4429279 | 8.134828845 | -1.180883811 | Down | 1.87E-52 |
| 57577 | 4.32157178 | 1.906528901 | -1.180607724 | Down | 1.07E-12 |
| 140831 | 1.54797923 | 0.682915217 | -1.180607724 | Down | 0.00039289 |
| 55333 | 11.9808928 | 5.285933431 | -1.180505256 | Down | 2.32E-56 |
| 284391 | 1.57087069 | 0.694417006 | -1.177690241 | Down | 0.00031536 |
| 2034 | 53.7354319 | 23.77586597 | -1.176375795 | Down | 1.01E-178 |
| 5064 | 28.3712951 | 12.55379015 | -1.17630901 | Down | 4.26E-54 |
| 23098 | 1.29427146 | 0.572691951 | -1.17630901 | Down | 2.62E-07 |
| 51267 | 5.09269266 | 2.253425326 | -1.17630901 | Down | 2.62E-07 |
| 91392 | 4.72770176 | 2.094612732 | -1.174455497 | Down | 1.73E-11 |
| 23335 | 1.91985214 | 0.850794514 | -1.17411257 | Down | 1.62E-10 |
| 10982 | 16.8114267 | 7.453954132 | -1.173364315 | Down | 7.54E-37 |
| 84167 | 2.35754973 | 1.045614821 | -1.172936711 | Down | 1.65E-06 |
| 7571 | 4.91908861 | 2.182816439 | -1.172200228 | Down | 9.24E-12 |
| 652 | 11.7495908 | 5.219701822 | -1.170571215 | Down | 3.59E-16 |
| 54621 | 3.31088756 | 1.472044533 | -1.1693967 | Down | 3.99E-12 |
| 51205 | 6.52294846 | 2.902622115 | -1.168167466 | Down | 5.03E-09 |
| 4052 | 26.6583035 | 11.86273489 | -1.168148319 | Down | 1.88E-105 |
| 22794 | 41.9661275 | 18.67538736 | -1.168087176 | Down | 5.19E-113 |
| 133746 | 4.10343341 | 1.826607952 | -1.167664522 | Down | 2.15E-25 |
| 114819 | 2.86628351 | 1.276355858 | -1.167150696 | Down | 2.45E-11 |
| 83989 | 9.25182174 | 4.123991045 | -1.16569627 | Down | 2.39E-29 |
| 64400 | 14.2004706 | 6.330961668 | -1.165442174 | Down | 1.20E-21 |
| 152273 | 4.82098391 | 2.150673828 | -1.164538873 | Down | 1.23E-19 |
| 64921 | 4.12761809 | 1.841461849 | -1.164457984 | Down | 1.06E-11 |
| 3782 | 4.17537615 | 1.864634038 | -1.163013664 | Down | 1.42E-09 |
| 654466 | 1.94136453 | 0.86716204 | -1.162697526 | Down | 3.00E-07 |
| 201134 | 5.19499876 | 2.320484208 | -1.162697526 | Down | 3.98E-13 |
| 253512 | 2.53524495 | 1.133284176 | -1.161615478 | Down | 2.42E-07 |
| 130916 | 7.3910883 | 3.305110571 | -1.161088273 | Down | 6.55E-23 |
| 1024 | 4.2384672 | 1.895336946 | -1.161088273 | Down | 3.60E-06 |
| 90203 | 22.4201525 | 10.02668753 | -1.160951024 | Down | 1.26E-46 |
| 1979 | 20.5811575 | 9.206607942 | -1.160582508 | Down | 4.81E-99 |
| 3199 | 5.49090187 | 2.459664785 | -1.158581418 | Down | 1.28E-07 |
| 353088 | 6.68222059 | 2.994554191 | -1.157986369 | Down | 1.84E-11 |
| 5931 | 62.3426708 | 27.96993725 | -1.156342945 | Down | 2.89E-80 |
| 283 | 13.3158511 | 5.974431676 | -1.156271257 | Down | 9.78E-12 |
| 57704 | 27.9880225 | 12.57526043 | -1.154221278 | Down | 1.10E-64 |
| 5243 | 0.86566908 | 0.388975724 | -1.154135513 | Down | 0.00056237 |
| 7088 | 11.061945 | 4.972174616 | -1.153656212 | Down | 3.31E-24 |
| 399687 | 5.94292409 | 2.672145915 | -1.153174164 | Down | 9.45E-30 |
| 113235 | 0.99915457 | 0.449371556 | -1.152799065 | Down | 1.86E-05 |
| 25961 | 2.07877611 | 0.935350663 | -1.152155148 | Down | 0.00045087 |
| 25932 | 75.549065 | 34.00347452 | -1.151731731 | Down | 2.63E-209 |
| 23510 | 15.840168 | 7.135067619 | -1.150588627 | Down | 4.84E-38 |
| 29761 | 9.11825708 | 4.108023069 | -1.150313802 | Down | 8.75E-30 |
| 256435 | 2.08497244 | 0.939336849 | -1.150313802 | Down | 1.21E-05 |
| 57523 | 4.63227773 | 2.087287126 | -1.150092682 | Down | 3.06E-24 |
| 467 | 34.0540522 | 15.34973264 | -1.149612954 | Down | 1.75E-51 |
| 8187 | 3.85934062 | 1.739790531 | -1.149440764 | Down | 3.42E-07 |
| 8214 | 17.1774226 | 7.745107555 | -1.149156405 | Down | 1.59E-14 |
| 81558 | 13.1440134 | 5.932390317 | -1.147720425 | Down | 8.06E-21 |
| 50717 | 45.6329729 | 20.61357461 | -1.14648194 | Down | 3.08E-119 |
| 27146 | 1.3017098 | 0.588419286 | -1.145491418 | Down | 0.00018703 |
| 55799 | 4.20797618 | 1.904014797 | -1.144081848 | Down | 4.48E-11 |
| 2983 | 16.014878 | 7.248413012 | -1.143675741 | Down | 1.17E-33 |
| 55852 | 7.24251899 | 3.279246998 | -1.14312699 | Down | 2.61E-24 |
| 1743 | 37.1550666 | 16.82426868 | -1.14301516 | Down | 1.75E-66 |
| 2252 | 8.37593195 | 3.797218201 | -1.141306818 | Down | 7.53E-22 |
| 613037 | 4.9732473 | 2.257314938 | -1.139580456 | Down | 4.43E-12 |
| 23741 | 143.356161 | 65.07987569 | -1.139320513 | Down | 2.88E-184 |
| 9727 | 13.5958399 | 6.172595606 | -1.139216093 | Down | 5.47E-37 |
| 285512 | 1.8004958 | 0.817436259 | -1.139216093 | Down | 6.27E-05 |
| 4144 | 94.1996452 | 42.79168144 | -1.138391258 | Down | 2.95E-163 |
| 10641 | 10.7954299 | 4.90592755 | -1.137822857 | Down | 1.55E-12 |
| 57711 | 5.33536624 | 2.426812775 | -1.136524497 | Down | 1.32E-18 |
| 10603 | 3.30069047 | 1.501655886 | -1.136213605 | Down | 3.27E-05 |
| 84162 | 2.85961429 | 1.301572615 | -1.135564765 | Down | 9.88E-29 |
| 7975 | 18.3016513 | 8.333172971 | -1.13503599 | Down | 6.49E-39 |
| 51097 | 13.1328523 | 5.980710384 | -1.13479153 | Down | 2.23E-21 |
| 64328 | 4.70906855 | 2.145718496 | -1.133980906 | Down | 6.65E-30 |
| 57179 | 47.6019666 | 21.70914466 | -1.132718292 | Down | 4.97E-83 |
| 463 | 7.13338917 | 3.253688435 | -1.132511576 | Down | 6.01E-71 |
| 26240 | 10.8609903 | 4.955307361 | -1.132109206 | Down | 3.31E-12 |
| 3913 | 81.1013618 | 37.00876515 | -1.131859141 | Down | 2.38E-285 |
| 54726 | 5.03887666 | 2.299665934 | -1.131677843 | Down | 3.47E-23 |
| 6764 | 46.7859254 | 21.3659561 | -1.130760713 | Down | 1.05E-129 |
| 4724 | 83.9949432 | 38.38483973 | -1.129765852 | Down | 6.61E-36 |
| 7360 | 101.374854 | 46.32830054 | -1.129734169 | Down | 7.87E-134 |
| 118 | 82.230429 | 37.59074814 | -1.129294728 | Down | 1.62E-202 |
| 154791 | 35.3627911 | 16.17306422 | -1.128639102 | Down | 8.20E-16 |
| 84340 | 5.10078332 | 2.332983698 | -1.128542591 | Down | 4.68E-11 |
| 91404 | 5.32245816 | 2.434718146 | -1.128337933 | Down | 3.93E-35 |
| 199704 | 1.92752577 | 0.881781593 | -1.128256881 | Down | 3.04E-06 |
| 4122 | 12.1470766 | 5.557584266 | -1.128079329 | Down | 1.84E-47 |
| 6487 | 19.7009794 | 9.014320055 | -1.127976773 | Down | 1.07E-30 |
| 56957 | 8.81882 | 4.03544348 | -1.127858401 | Down | 1.13E-35 |
| 8325 | 3.89777512 | 1.784453604 | -1.127168465 | Down | 5.54E-09 |
| 222255 | 2.29188758 | 1.050223414 | -1.125840016 | Down | 2.37E-09 |
| 145567 | 13.9420171 | 6.388849156 | -1.125811315 | Down | 2.25E-30 |
| 5566 | 65.6844812 | 30.10416213 | -1.125589592 | Down | 1.11E-106 |
| 8418 | 5.79123729 | 2.657992699 | -1.123534471 | Down | 4.37E-10 |
| 728118 | 1.4964912 | 0.687205006 | -1.122771342 | Down | 0.00026516 |
| 10395 | 7.37489852 | 3.387410476 | -1.12244038 | Down | 1.98E-34 |
| 84620 | 1.59509298 | 0.733665439 | -1.120446289 | Down | 5.24E-08 |
| 83931 | 35.5990216 | 16.37423022 | -1.120410507 | Down | 5.33E-83 |
| 55827 | 30.4536056 | 14.01097664 | -1.120055526 | Down | 2.98E-61 |
| 9917 | 16.0146434 | 7.368499233 | -1.119948958 | Down | 3.11E-58 |
| 55203 | 3.64700522 | 1.678759694 | -1.119316535 | Down | 2.42E-15 |
| 126272 | 3.14085475 | 1.446036707 | -1.119053053 | Down | 7.13E-05 |
| 374868 | 4.06915022 | 1.873643205 | -1.118881294 | Down | 4.24E-12 |
| 284309 | 2.24415754 | 1.033384685 | -1.118796551 | Down | 1.46E-08 |
| 4154 | 39.0512941 | 17.99438368 | -1.117823669 | Down | 1.83E-149 |
| 26046 | 2.50741935 | 1.155909254 | -1.117175155 | Down | 4.20E-13 |
| 166379 | 2.04646116 | 0.943680268 | -1.116761242 | Down | 2.40E-05 |
| 219931 | 5.34566063 | 2.465374313 | -1.116561547 | Down | 1.60E-17 |
| 7769 | 4.33763163 | 2.000479706 | -1.116561547 | Down | 1.76E-09 |
| 55602 | 5.72445255 | 2.640427007 | -1.11636647 | Down | 1.42E-09 |
| 11095 | 9.27508214 | 4.278169339 | -1.11636647 | Down | 9.62E-22 |
| 1028 | 39.6987767 | 18.31643121 | -1.115956118 | Down | 6.02E-47 |
| 5376 | 148.728178 | 68.62164243 | -1.115942445 | Down | 7.10E-162 |
| 38 | 65.5006697 | 30.22188121 | -1.11591619 | Down | 4.40E-84 |
| 54910 | 24.5829655 | 11.34771981 | -1.115256531 | Down | 3.31E-53 |
| 10494 | 52.2021503 | 24.10310651 | -1.114890135 | Down | 1.45E-67 |
| 283820 | 1.85477578 | 0.856901792 | -1.114043022 | Down | 4.27E-06 |
| 9755 | 7.8631841 | 3.633819383 | -1.11362692 | Down | 8.73E-21 |
| 51652 | 128.800328 | 59.5604881 | -1.11270878 | Down | 4.26E-241 |
| 79567 | 28.5352699 | 13.19864987 | -1.112355852 | Down | 6.92E-70 |
| 648 | 12.8564833 | 5.947960555 | -1.112029079 | Down | 1.30E-28 |
| 11112 | 30.0090834 | 13.89221489 | -1.111122624 | Down | 1.64E-36 |
| 178 | 5.36830785 | 2.48537641 | -1.111003042 | Down | 3.97E-25 |
| 3398 | 68.1966148 | 31.58126633 | -1.110631106 | Down | 3.38E-56 |
| 5311 | 15.0458704 | 6.977417754 | -1.108602453 | Down | 3.80E-46 |
| 5167 | 4.32387347 | 2.007800809 | -1.106708156 | Down | 2.07E-20 |
| 6430 | 214.675679 | 99.68579894 | -1.106698851 | Down | 1.27E-207 |
| 288 | 2.24192657 | 1.041187599 | -1.106508994 | Down | 5.93E-21 |
| 90952 | 28.4579254 | 13.22046791 | -1.106057253 | Down | 1.17E-32 |
| 25 | 22.8557311 | 10.62150535 | -1.105567716 | Down | 8.12E-80 |
| 51004 | 15.2001908 | 7.069868774 | -1.104334093 | Down | 9.91E-16 |
| 1911 | 5.77922938 | 2.689648511 | -1.103459482 | Down | 5.34E-19 |
| 140467 | 61.5062881 | 28.66258834 | -1.101565016 | Down | 4.07E-71 |
| 81532 | 67.0146169 | 31.24495369 | -1.100852601 | Down | 1.39E-57 |
| 57017 | 74.2844543 | 34.63987317 | -1.100626678 | Down | 5.62E-71 |
| 22836 | 19.027508 | 8.878567922 | -1.099687729 | Down | 9.52E-63 |
| 23107 | 21.6249525 | 10.09058094 | -1.099687729 | Down | 2.21E-36 |
| 85315 | 1.94514677 | 0.907639494 | -1.099687729 | Down | 8.66E-07 |
| 285331 | 6.62900079 | 3.09320767 | -1.099687729 | Down | 1.38E-13 |
| 152559 | 3.17507179 | 1.481544009 | -1.099687729 | Down | 2.31E-08 |
| 132001 | 3.8646133 | 1.803296137 | -1.099687729 | Down | 0.00037419 |
| 2313 | 6.79152941 | 3.169046367 | -1.099687729 | Down | 3.18E-17 |
| 23270 | 5.67193814 | 2.646625507 | -1.099687729 | Down | 3.92E-15 |
| 114900 | 3.20364223 | 1.494875479 | -1.099687729 | Down | 0.00058221 |
| 55325 | 22.2587452 | 10.38631968 | -1.099687729 | Down | 3.10E-32 |
| 1305 | 2.04157228 | 0.95263332 | -1.099687729 | Down | 4.19E-05 |
| 8228 | 3.89552776 | 1.817721362 | -1.099687729 | Down | 6.68E-08 |
| 79963 | 4.53365947 | 2.115484778 | -1.099687729 | Down | 2.04E-06 |
| 84836 | 26.5471852 | 12.38738075 | -1.099687729 | Down | 5.38E-39 |
| 2040 | 126.732502 | 59.15931223 | -1.099109378 | Down | 1.71E-225 |
| 23331 | 6.63603777 | 3.10267187 | -1.096810968 | Down | 1.23E-46 |
| 84532 | 8.53837819 | 3.992304109 | -1.096740444 | Down | 1.32E-23 |
| 23208 | 6.38331308 | 2.985508533 | -1.096328716 | Down | 6.86E-21 |
| 10427 | 6.94282586 | 3.247488109 | -1.096198742 | Down | 3.64E-20 |
| 7741 | 8.12644117 | 3.80488131 | -1.094772238 | Down | 1.02E-14 |
| 80778 | 8.11298203 | 3.804493391 | -1.092527937 | Down | 1.61E-10 |
| 644128 | 2.60521941 | 1.221939586 | -1.092231924 | Down | 3.75E-10 |
| 651250 | 7.55831499 | 3.549637764 | -1.090392836 | Down | 8.32E-50 |
| 863 | 2.80180445 | 1.315915854 | -1.090289031 | Down | 2.60E-08 |
| 51673 | 157.102114 | 73.78950897 | -1.090214976 | Down | 1.11E-102 |
| 63826 | 4.76119936 | 2.236371841 | -1.090164954 | Down | 3.21E-08 |
| 23092 | 4.54446476 | 2.13665239 | -1.088758168 | Down | 5.43E-25 |
| 84247 | 7.04155566 | 3.31309393 | -1.087715087 | Down | 6.53E-23 |
| 10868 | 10.197833 | 4.798881786 | -1.087492432 | Down | 9.64E-28 |
| 51809 | 2.08539532 | 0.9815435 | -1.087196784 | Down | 1.50E-06 |
| 2036 | 11.4366282 | 5.388792676 | -1.085627785 | Down | 3.08E-42 |
| 51422 | 13.4335535 | 6.33251179 | -1.084991217 | Down | 1.64E-27 |
| 2006 | 56.9123123 | 26.82919036 | -1.084937277 | Down | 5.40E-113 |
| 197131 | 4.74968613 | 2.23981405 | -1.084453215 | Down | 2.07E-22 |
| 9767 | 1.47273737 | 0.694594307 | -1.084257688 | Down | 1.60E-05 |
| 23641 | 26.2039843 | 12.35928083 | -1.084191392 | Down | 4.77E-22 |
| 84079 | 9.68170418 | 4.566757364 | -1.084090873 | Down | 5.62E-26 |
| 54793 | 14.8621946 | 7.01140825 | -1.083871023 | Down | 1.25E-29 |
| 90233 | 1.83597006 | 0.866542806 | -1.083199606 | Down | 3.06E-05 |
| 163050 | 4.61141851 | 2.176501475 | -1.083199606 | Down | 3.49E-09 |
| 10938 | 57.6337005 | 27.21969736 | -1.082261627 | Down | 1.82E-113 |
| 23466 | 15.3432869 | 7.248660219 | -1.081821308 | Down | 7.52E-30 |
| 7177 | 190.808417 | 90.1638812 | -1.081503289 | Down | 2.55E-129 |
| 8572 | 69.6213216 | 32.89975961 | -1.081452157 | Down | 3.47E-91 |
| 730101 | 1.58929685 | 0.751224786 | -1.08107205 | Down | 9.08E-05 |
| 7318 | 12.6124011 | 5.962169034 | -1.080933768 | Down | 4.09E-25 |
| 27309 | 15.74449 | 7.443067103 | -1.080877879 | Down | 2.47E-18 |
| 5333 | 7.75887554 | 3.66837966 | -1.080704625 | Down | 7.67E-15 |
| 9855 | 2.85288413 | 1.34944131 | -1.080058922 | Down | 6.82E-08 |
| 56131 | 1.48828461 | 0.704524255 | -1.078929168 | Down | 0.00021777 |
| 137886 | 7.22772571 | 3.422920248 | -1.078314078 | Down | 3.94E-22 |
| 22862 | 5.75270416 | 2.724895898 | -1.078039165 | Down | 7.37E-22 |
| 4329 | 7.05789444 | 3.343489287 | -1.077883358 | Down | 3.82E-10 |
| 84765 | 4.93839322 | 2.339792016 | -1.077661422 | Down | 4.72E-10 |
| 3660 | 15.4086909 | 7.301560463 | -1.077467564 | Down | 2.57E-21 |
| 9658 | 2.25192817 | 1.06746825 | -1.076967652 | Down | 8.90E-10 |
| 5175 | 49.9226342 | 23.71487811 | -1.073901608 | Down | 1.10E-97 |
| 2323 | 16.5854917 | 7.879790968 | -1.07369252 | Down | 4.21E-11 |
| 8939 | 7.76820168 | 3.694042012 | -1.072380383 | Down | 3.37E-15 |
| 309 | 128.554449 | 61.16096417 | -1.071696482 | Down | 2.53E-208 |
| 10241 | 45.0244049 | 21.43113471 | -1.07099897 | Down | 3.03E-83 |
| 23387 | 14.2203592 | 6.773582053 | -1.06996703 | Down | 2.70E-49 |
| 5071 | 4.46793593 | 2.129555587 | -1.06905611 | Down | 1.33E-11 |
| 7767 | 2.91380297 | 1.389188614 | -1.068660833 | Down | 2.22E-05 |
| 201283 | 3.10867362 | 1.482325582 | -1.068436795 | Down | 2.22E-07 |
| 25981 | 0.81623729 | 0.389210821 | -1.068436795 | Down | 2.22E-07 |
| 83714 | 2.53214443 | 1.207799372 | -1.067978869 | Down | 2.76E-05 |
| 147912 | 15.3156126 | 7.310694953 | -1.066922618 | Down | 1.08E-29 |
| 220929 | 4.86713028 | 2.324400013 | -1.066213024 | Down | 1.09E-10 |
| 10233 | 3.64681346 | 1.7421828 | -1.065740397 | Down | 5.30E-05 |
| 10404 | 27.5171566 | 13.14753097 | -1.065539506 | Down | 2.82E-30 |
| 5128 | 5.62431407 | 2.687533259 | -1.065394551 | Down | 4.65E-14 |
| 119710 | 23.4307765 | 11.19987363 | -1.06492231 | Down | 4.21E-12 |
| 10079 | 11.9420712 | 5.711808901 | -1.064033459 | Down | 3.63E-51 |
| 1959 | 4.09546345 | 1.960015232 | -1.063161853 | Down | 3.65E-08 |
| 23091 | 63.588595 | 30.43352564 | -1.063106557 | Down | 1.91E-223 |
| 4548 | 6.50238445 | 3.113517519 | -1.06242346 | Down | 3.94E-39 |
| 23248 | 9.13094981 | 4.372538138 | -1.062293971 | Down | 1.30E-40 |
| 7694 | 2.91851603 | 1.397985402 | -1.061885696 | Down | 2.88E-06 |
[truncated: 9,768 more chars]
